# Diverse, Novel Mycoviruses From the Virome of a Hypovirulent *Sclerotium rolfsii* Strain

**DOI:** 10.3389/fpls.2018.01738

**Published:** 2018-11-27

**Authors:** Jun Zi Zhu, Hong Jian Zhu, Bi Da Gao, Qian Zhou, Jie Zhong

**Affiliations:** Hunan Provincial Key Laboratory for Biology and Control of Plant Diseases and Insect Pests, Hunan Agricultural University, Changsha, China

**Keywords:** Mycovirus, *Sclerotium rolfsii*, virome, deep sequencing, biocontrol

## Abstract

*Sclerotium rolfsii*, which causes southern blight in a wide variety of crops, is a devastating plant pathogen worldwide. Mycoviruses that induce hypovirulence in phytopathogenic fungi are potential biological control resources against fungal plant diseases. However, in *S. rolfsii*, mycoviruses are rarely reported. In a previous study, we found a hypovirulent strain carrying a diverse pattern of dsRNAs. Here, we utilized the RNA_Seq technique to detect viral sequences. Deep sequencing, RT-PCR and Sanger sequencing validation analyses revealed that this strain harbors various new viral species that show affinity to the distinctly established and proposed families *Benyviridae, Endornaviridae*, Fusariviridae, *Hypoviridae*, and Fusagraviridae. Moreover, some viral sequences that could not be assigned to any of the existing families or orders were also identified and showed similarities to the *Alphavirus, Ourmiavirus*, phlegivirus-like and Curvularia thermal tolerance virus-like groups. In addition, we also conducted deep sequencing analysis of small RNAs in the virus-infecting fugal strain. The results indicated that the Dicer-mediated gene silencing mechanism was present in *S. rolfsii*. This is the first report of viral diversity in a single *S. rolfsii* fungal strain, and the results presented herein might provide insight into the taxonomy and evolution of mycoviruses and be useful for the exploration of mycoviruses as biocontrol agents.

## Introduction

Mycoviruses are a type of virus that infect and replicate in fungi, infecting all major taxonomic fungal groups (Ghabrial and Suzuki, 2009). Most mycoviruses are associated with latent infections. However, several mycoviruses can obviously cause abnormal symptoms in the host, such as hypovirulence and debilitation, and thus hold great promise for exploitation as biological agents to control fungal diseases. Since Cryphonectria hypovirus 1 (CHV1) was successfully used to control chest blight disease in Europe (Nuss, 1992; Xie and Jiang, 2014; Ghabrial et al., 2015), phytopathologists have been inspired to study mycovirus-mediated hypovirulence in plant pathogenic fungi. Recently, the DNA virus *Sclerotinia sclerotiorum* hypovirulence-associated DNA virus 1 (SsHADV-1) was proven to possess the potential for biologically controlling sclerotinia disease under field conditions (Yu et al., 2010, 2013). Rosellinia necatrix megabirnavirus 1 has the ability to control apple white root rot disease caused by *Rosellinia necatrix* (Chiba et al., 2009; Liu et al., 2016). With the rare exceptions of DNA and negative-sense RNA (-ssRNA) (Liu et al., 2014), mycoviral genomes consist of double-stranded (ds) or positive-sense single-stranded (+ss) RNA. With the increasing number of mycoviruses identified, some of which have unique molecular and biological properties unlike those of any other known mycoviruses and cannot be assigned into any of the established virus families, mycovirus classification is being refined. Thus, the discovery of novel mycoviruses that have diverse molecular and biological properties will facilitate our understanding of viral ecology and evolution.

The development and application of high-throughput next generation sequencing (NGS) technologies and bioinformatics have greatly enhanced the discovery of new viruses in many organisms, including fungi (Marzano et al., 2016; Zhang et al., 2018). NGS techniques can detect the presence of viral sequences regardless of the sample viral titer and does not require prior knowledge of the genomic sequences of candidate viruses (Mokili et al., 2012; Roossinck, 2015). To date, some examples in deep sequencing of the transcriptome (RNA-seq) (Schoebel et al., 2014; Li et al., 2015; Liu et al., 2016), ribosomal RNA (rRNA)-depleted total RNAs, small interfering RNAs (Marzano et al., 2015; Vainio et al., 2015; Shi et al., 2016; Remnant et al., 2017; Mu et al., 2018), and viral dsRNA (Coetzee et al., 2010; Al Rwahnih et al., 2011; Bartholomäus et al., 2016) have proven the methods to be useful for facilitating the discovery of mycoviruses or other RNA and DNA viruses infecting plants and insects. The NGS data and analysis tools have advanced the virology research in areas of viral evolutionary, ecology, epidemiology, genomic diversity, and interactions between viruses and hosts (Zhang et al., 2018). Because some material needed for deep sequencing library construction can be infected by viruses, we can use transcriptomic data for virus identification and genome assembly *in silico*, which has recently been demonstrated in several studies (Jo et al., 2015, 2016a,b). Usually, RNA viruses with poly (A) tails and polyadenylated RNA viruses that can be collected together with mRNA using oligo d(T) are easily sequenced in transcriptome libraries (Gu et al., 2014). Recently, several recent studies also showed that rRNA depleted RNA libraries were suitable for virus identification lacking poly (A) tails (Jo et al., 2015; Mu et al., 2018). In addition, sequencing of rRNA depleted RNA could reveal an entire micro-organisms in samples collected from environment materials. However, with the increasing of enormous and complicated NGS data, we would be more dependent on the accuracy of sequence assembly and homology searching for discover of new viruses (Zhang et al., 2018).

*Sclerotium rolfsii* Sacc. [*Athelia rolfsii* (Curzi) Tu Kimbrough] is a soil-borne pathogen that causes southern blight disease in a wide variety of crops (Rivard et al., 2010). *S. rolfsii* infects more than 600 plant species and is thus a serious problem. Moreover, *S. rolfsii* produces sclerotia, which plays a key role in the disease cycle, and has the ability to survive in soil for long periods (Punja, 1985; Xu et al., 2008), thus serving as an important barrier to controlling this disease. Because hypovirulence-associated mycoviruses reportedly have potential for development as biocontrol agents for fungal diseases, screening for mycoviruses possessing biological control potential is significant for the control of southern blight disease.

Previously, we isolated a hypovirulent *S. rolfsii* strain, BLH-1, that contains a series of dsRNAs. Virus-curing and horizontal transmission experiments confirmed that dsRNAs were associated with the hypovirulent traits of BLH-1 (Zhong et al., 2016a). This study aimed to further clarify the species and genomes of all the mycoviruses infecting BLH-1, and to determine the possibility of interactions between the RNA silencing machinery and mycoviruses in *S. rolfsii*. To do this, the following procedures were utilized in this study: transcriptome deep sequencing of the RNA from BLH-1, *de novo* sequence assembly, homology searches against reference viruses in the database, and Sanger sequencing of the RT-PCR amplicons used for virus confirmation.

## Results

### Transcriptomic identification of mycoviruses infecting the *S. rolfsii* strain BLH-1

Two high-throughput sequencing libraries were constructed and sequenced on the Illumina MiSeq 2000/2500 platform, producing approximately 3.6 to 4.7 × 10^7^ paired-end reads with lengths of 300 nt. Raw reads were cleaned and *de novo* assembled into contigs using Trinity. All resulting contigs were compared to the *S. rolfsii* reference genome. BLAST searches of the remaining contigs against the NCBI viral database revealed the presence of viral sequences representing partial genomic segments of several distinct mycoviruses. The predicted amino acid sequences of the putative viral genomes showed significant sequence identity with previously described viruses from several distinct lineages, including the families Fusariviridae and *Hypoviridae*, the “alphavirus-like” and “benyvirus-like” viruses and some other unassigned dsRNA mycoviruses. A list of all viruses in the viral database that showed the most BLASTx matches to the assembled provisional viruses generated in this study is shown in Table 1. Because most of these assembled viral sequences shared less than 50% amino acid identity with the previously described mycovirus, we suggest that they are novel viruses.

**Table 1:** Assembled viral sequences in the *Sclerotium rolfsii* strain BLH-1.

**Name**	**Accession**	**Sequence length**	**Closest relative virus (BLASTx)**	**Maximum Identity (%)**	**Genome type**	**Tentative virusclassification**	**References**
*Sclerotium rolfsiii* beny-like virus 1(SrBenV1)	MH766487	7,865	Agaricus bisporus virus 8	30	+ssRNA	Benyvirus-like	NP_612601.1
*Sclerotium rolfsiii* alphavirus-like virus 1 (SraLV1)	MH766488	7,550	*Sclerotinia sclerotiorum* RNA virus L	26	+ssRNA	“Alphavirus-like” supergroup	ACE88957.1
*Sclerotium rolfsiii* alphavirus-like virus 2 (SraLV2)	MH766489	7,591	*Sclerotinia sclerotiorum* RNA virus L	26	+ssRNA	“Alphavirus-like” supergroup	ACE88957.1
*Sclerotium rolfsiii* alphavirus-like virus 3 (SraLV3)	MH766490	7,888	*Sclerotinia sclerotiorum* RNA virus L	24	+ssRNA	“Alphavirus-like” supergroup	ACE88957.1
*Sclerotium rolfsiii* fusarivirus 1 (SrFV1)	MH766491	7,301	Agaricus bisporus virus 10	42	+ssRNA	Fusariviridae	YP_009182158.1
*Sclerotium rolfsiii* fusarivirus 2 (SrFV2)	MH766492	7,281	Agaricus bisporus virus 10	40	+ssRNA	Fusariviridae	YP_009052456.1
*Sclerotium rolfsiii* hypovirus 1 (SrHV1)	KU885931.1	16,050	*Sclerotinia sclerotiorum* hypovirus 2	57	+ssRNA	Hypoviridae	AHE13861.1
*Sclerotium rolfsiii* hypovirus 2 (SrHV2)	MH766497	11,010	Ceratobasidium hypovirus A	26	+ssRNA	Hypoviridae	AOX47536.1
*Sclerotium rolfsiii* hypovirus 3 (SrHV3)	MH766498	10,739	Ceratobasidium hypovirus A	24	+ssRNA	Hypoviridae	AOX47536.1
*Sclerotium rolfsiii* hypovirus 4 (SrHV4)	MH766499	11,035	Ceratobasidium hypovirus A	25	+ssRNA	Hypoviridae	AOX47536.1
*Sclerotium rolfsiii* hypovirus 5(SrHV5)	MH766500	10,758	Ceratobasidium hypovirus A	25	+ssRNA	Hypoviridae	AOX47536.1
*Sclerotium rolfsiii* hypovirus 6 (SrHV6)	MH766501	7,382	Beihai sipunculid worm virus 6	24	+ssRNA	Hypoviridae	YP_009333562.1
*Sclerotium rolfsiii* hypovirus 7 (SrHV7)	MH766502	10,961	Ceratobasidium hypovirus A	25	+ssRNA	Hypoviridae	AOX47536.1
*Sclerotium rolfsiii* hypovirus 8 (SrHV8)	MH766503	12,791	Cryphonectria hypovirus 1	26	+ssRNA	Hypoviridae	ATZ76097.1
*Sclerotium rolfsiii* ourmia-like virus 1 (SrOLV1)	MH766504	443	Magnaporthe oryzae ourmia-like virus	42	+ssRNA	Ourmiavirus	SBQ28480.1
*Sclerotium rolfsiii* endornavirus 1 (SrEV1)	MH766493	12,081	*Sclerotinia sclerotiorum* endornavirus 2	25	+ssRNA	Endornaviridae	AND83000.1
*Sclerotium rolfsii* RNA virus 1 (SrRV1)	MH766494	6,720	Rosellinia necatrix fusagravirus 2	34	dsRNA	“Fusagraviridae”	BBB86783.1
*Sclerotium rolfsii* RNA virus 2 (SrRV2)	MH766495	6,182	Phlebiopsis gigantea mycovirus dsRNA 2	37	dsRNA	“Fusagraviridae”	CAJ34335.2
*Sclerotium rolfsii* mycovirus dsRNA 1 (SrMYV1)	KU885933.1	10,173	Phlebiopsis gigantea mycovirus dsRNA 1	51	dsRNA	Unclassified	YP_003541123.1
*Sclerotium rolfsiii* unassigned dsRNA virus 1 (SrURV1)	MH766505	1,910	Heterobasidion RNA virus 6	46	dsRNA	Unclassified	AHA82552.1
*Sclerotium rolfsiii* unassigned dsRNA virus 1 (SrURV2)	MH766506	1,958	Heterobasidion RNA virus 6	57	dsRNA	Unclassified	AHA82552.1

### Benyvirus-like viral sequences

**Figure 1:**
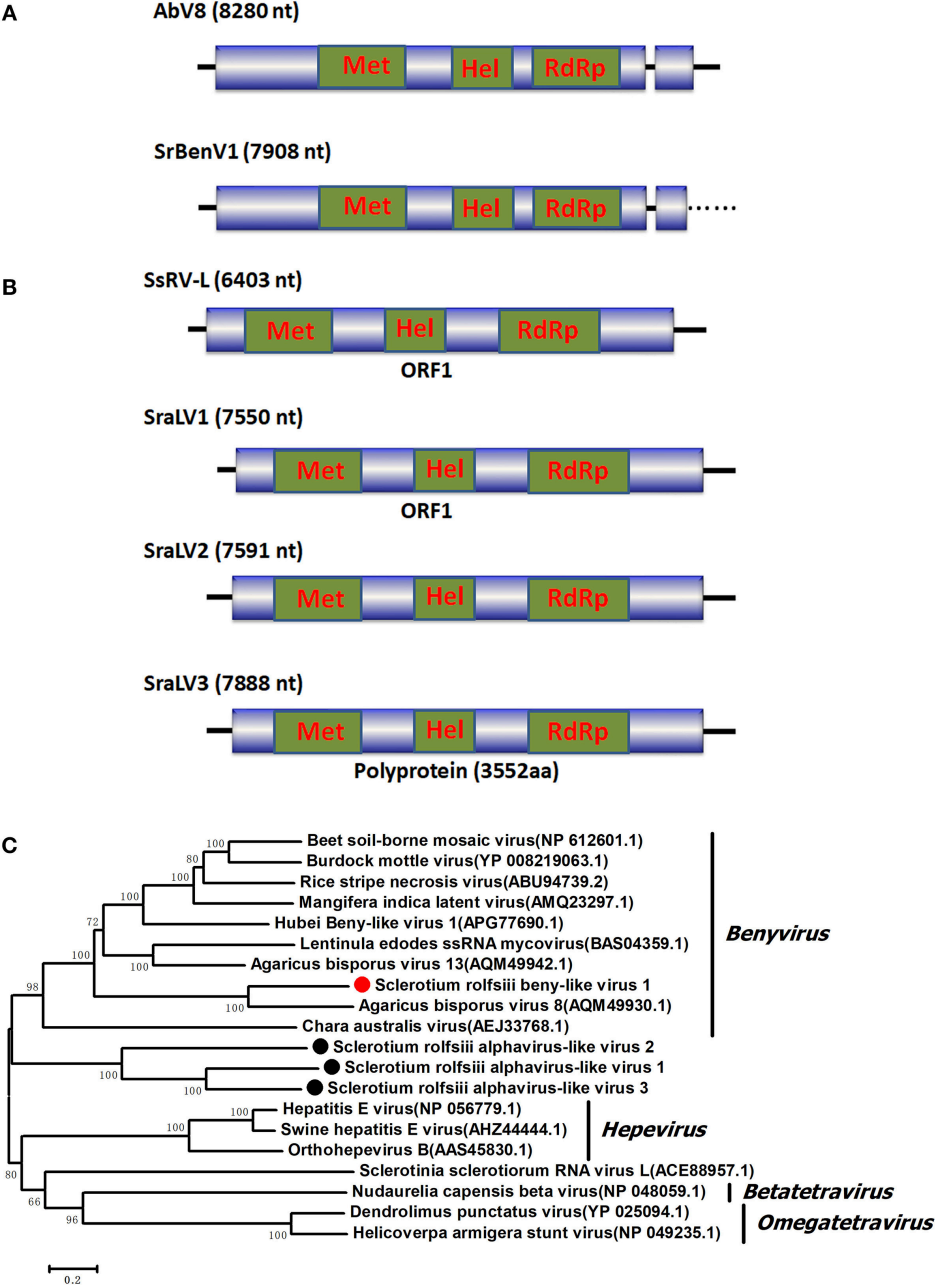
Genomic organization and phylogenetic analysis of the *Benyvirus*-like and Alphavirus-like viral sequences and **(A)** Diagrammatic representations of the predicted genomic organization of SrBenV1 showing the presence of a large 5′ open reading frames (ORF) and a 3′ incomplete ORF, comparisons with those of related AbV8. **(B)** Genome organizations of the *Alphavirus*-like viral sequences, SraLV1, SraLV2, SraLV3, and the AbV8 which related to these containing a single large ORF. **(C)** Phylogenetic analysis of the SrBenV1, SraLV1, SraLV2, and SraLV3 based on amino acid alignments of the replicase encoded protein of the four viruses and other *Benyvirus* and *Alphavirus* viruses related to these. Phylogenetic tree was constructed by Neighbor-Joining algorithm using MEGA6, with a 1000 bootstrap replications. The percentage of bootstrap values supporting the branches in phylogenetic trees were indicated on the nodes. The genetic distance was represented by the scale bar of 0.2 amino acid substitutions per site. The novel virus SrBenV1, SraLV1, SraLV2, and SraLV3 were indicated by red dots. Names and database accession numbers of other related viruses analyzed were indicated in the tree.

A sequence named *Sclerotium rolfsii* beny-like virus 1 (SrBenV1), 7908 nt in length, was predicted to encode proteins showing similarity to the replicases of Benyvirus-like viruses, such as Agaricus bisporus virus 8 (AbV8), beet soil-borne mosaic virus (BsBMV), and Mangifera indica latent virus (MiLV) (Figure 1). BsBMV was identified as a member of the genus *Benyvirus* in the family *Benyviridae* that consists of two + ssRNA genomic segments with sizes of 6,883 nt and 4,616 nt, each of which contains one ORF encoding a polyprotein. SrBenV1 was shown to contain a larger ORF1 and a 3′ incomplete ORF2. The predicted amino acid sequence of ORF1 was similar to that of the replicases from viruses in the family *Benyviridae* and most closely related to Agaricus bisporus virus 8 (AQM49930.1) with a 45% identity. ORF2 was incomplete and had no sequence identity to any other sequences in the database. Although benyviruses often have four to five linear positive-sense ssRNAs that are 6.7, 4.6, 1.8, 1.4, and 1.3 kb in size, with the first RNA encoding the RdRp (Gilmer et al., 2017), in this study, only the RdRp-encoded RNA1 was identified. Phylogenetic analysis of the SrBenV1 replicase was conducted and revealed that SrBenV1 was clustered in the family *Benyviridae*. These results showed that SrBenV1 belongs to the genome of a new viral species in the family *Benyviridae*. Based on the genome sizes of *Benyviridae* family members, we estimate that the SrBenV1 viral sequence covers approximately more than 90% of RNA1.

### Alphavirus-like viral sequences

BLASTx searches of the predicted amino acid sequences of three viral sequences (7,550, 7,591, and 7,888 nt in length) showed similarity to products encoded by alphavirus-like +ssRNA viruses, such as *Sclerotinia sclerotiorum* RNA virus L (SsRV-L), bat hepevirus (BHV), and hepatitis E virus (HEV), with identities ranging from 25 to 26%. SsRv-L is a +ssRNA virus with a 6,403 nt genome containing a single ORF that encodes a polyprotein with three domains: viral methyltransferase (pfam0l660), viral_helicase l (pfam0 l443), and RdRP (RdRp_2, pfam00978) (Liu et al., 2009). SsRv-L was most closely related to an HEV belonging to the alphavirus-like supergroup. A conserved domain search revealed that the three viral sequences also contained these three conserved domains, indicating that they might represent novel +ssRNA mycoviruses belonging to a virus taxonomic unit that is closely related to *Alphavirus*. We named the three virus sequences *Sclerotium rolfsii* alphavirus-like virus 1 (SraLV1), *Sclerotium rolfsii* alphavirus-like virus 2 (SraLV2) and *Sclerotium rolfsii* alphavirus-like virus 3 (SraLV3) (Figure 1).

### Sequences related to fusariviridae

**Figure 2:**
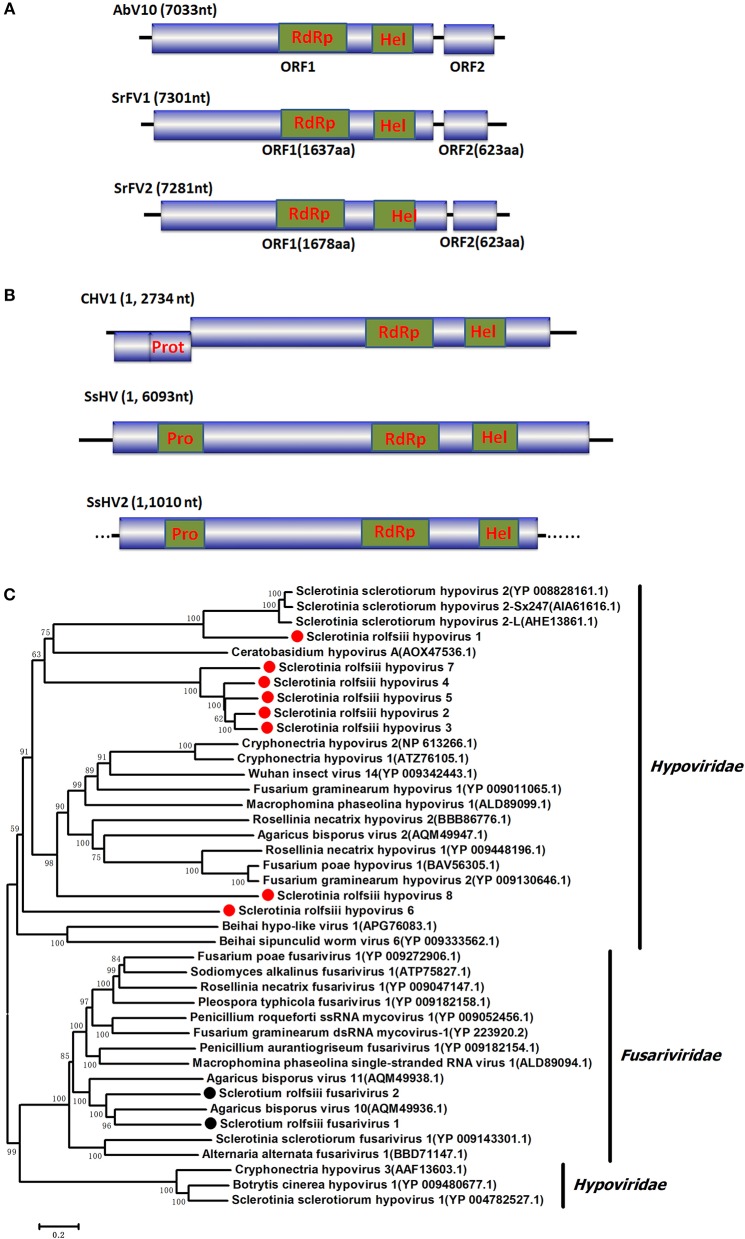
Genome organization and phylogeny of the viruses that similar to members of the *Fusariviriade* and *Hypoviridae* family. **(A)** Comparisons of the genome organizations of SrFV11 and SrFV2 and comparison with that of related virus AbV8 in the *Fusariviriade* family. **(B)** Genome organization of the hypoviruses, exampled as the SrHV1, SrHV2, and CHV1. **(C)** The predicted amino acid sequences of the RdRps were aligned and subjected for phylogenetic tree construction using the method described in Figure 1.

Two RNA sequences showed similarity to members of the family Fusariviridae, the two viral sequences of *Sclerotium rolfsii* fusarivirus 1 (SrFV1, 7301 nt) and *Sclerotium rolfsii* fusarivirus 2 (SrFV2, 7281 nt) (Figure 2). Fusariviridae is a new, recently proposed + ssRNA family comprising members that typically have 6 to 8 kbp genomes, with one larger ORF encoding putative polyproteins of replicases and one to three smaller ORFs encoding hypothetical proteins (Zhang et al., 2014). Both SrFV1 and SrFV2 contained two ORFs (ORF1 and ORF2). The ORF1 of each virus encoded polyproteins possessing conserved domains of RdRp (RdRp_1, cd01699) and Hel (helicase, cd00079), with aa identities ranging from 31 to 42%, and corresponded to proteins encoded by members of the Fusariviridae family, including Agaricus bisporus virus 10 (AbV10), Agaricus bisporus virus 11 (AbV11), *Fusarium graminearum* dsRNA mycovirus-1 (FgV1), Pleospora typhicola fusarivirus 1 (PtFV1), and Rosellinia necatrix fusarivirus 1 (RnFV1). Phylogenetic analysis based on ORF1 indicated that consistent with the homology search, SrFV1 and SrFV2 were clustered in the fusarivirus clade. Considering the genome organization and genome sizes, the two viruses likely represent the nearly complete genomes of novel members of the Fusariviridae family.

### Sequences related to *Hypoviridae*

A viral sequence 16,050 bp in length was identified. The predicted amino acid sequences of the viral genome were similar to those of viruses in the family *Hypoviridae*, with *Sclerotinia sclerotiorum* hypovirus 2 (SsHV2) being the best match at 57% identity (Figure 2). Viruses in the *Hypoviridae* family consist of +ssRNA genomes that range in size from 9 to 13 kb and contain one or two ORFs (Suzuki et al., 2018). Members of this family are currently grouped into two genera, *Alphahypovirus* and *Betahypovirus* (Yaegashi et al., 2012; Hu et al., 2014; Khalifa and Pearson, 2014). The CHV1 and CHV2 from *C. parasitica* were assigned to the genus *Alphahypovirus*, and their genomes consist of two ORFs. In addition to *Alphahypovirus* and *Betahypovirus*, a third distinct lineage and genus, named *Gamahypovirus*, was recently proposed with SsHV2/5472 and its conspecific SsHV2/SX247 as a prototype (Hu et al., 2014; Khalifa and Pearson, 2014). Based on the genome size and sequence similarity, we suggest that SrHV1 is a new member of the *Gamahypovirus* genus in the family *Hypoviridae*. Seven possible hypovirus-like viral sequences were obtained and showed sequence identities to viruses in the family *Hypoviridae*. Less sequence similarity was observed between these sequences, and these viral sequences might thus be different hypoviruses, designated as Sclerotinia rolfsii hypovirus 2 to Sclerotinia rolfsii hypovirus 8. These viruses were predicted to encode proteins similar to those encoded by Macrophomina phaseolina hypovirus 1 (MpHV1) and CHV1, with identities ranging from 25 to 26%. Hence, diverse infectious hypoviruses belonging to *Gamahypovirus* and *Alphahypovirus* might exist.

### Ourmiavirus-like viral sequences

One ourmiavirus-like sequence was identified, which was 443 bp in length and named *Sclerotium rolfsii* ourmia-like virus 1 (SrOLV1). A BLASTx search showed that this virus was similar to ourmiavirus-like mycoviruses, including Magnaporthe oryzae ourmia-like virus (MoOLV) and Rhizoctonia solani ourmia-like virus 1 RNA 1, with identities of 42 and 39%, respectively. Ourmiavirus genomes have three ssRNA segments of 2.8, 1.1, and 0.9 kb, with the largest RNA segment encoding the viral replicase, and the smallest segment encompassing the coat protein (Turina et al., 2017). Although the replicases of mitoviruses and ourmiaviruses are all phylogenetically related, ourmiaviruses are considered to replicate in the cytoplasm instead of mitochondria (Crivelli et al., 2011). A few ourmiavirus-like mycoviruses have previously been discovered from *Sclerotinia sclerotiorum, Rhizoctonia solani* (Marzano and Domier, 2016), *Magnaporthe oryzae* (Illana et al., 2017), and *Botrytis* (Donaire et al., 2016). Sequence similarity revealed that the oourmiavirus-like mycovirus SrOLV1 could also infect *S. rolfsii*.

### *Endornaviridae*-related sequences

**Figure 3:**
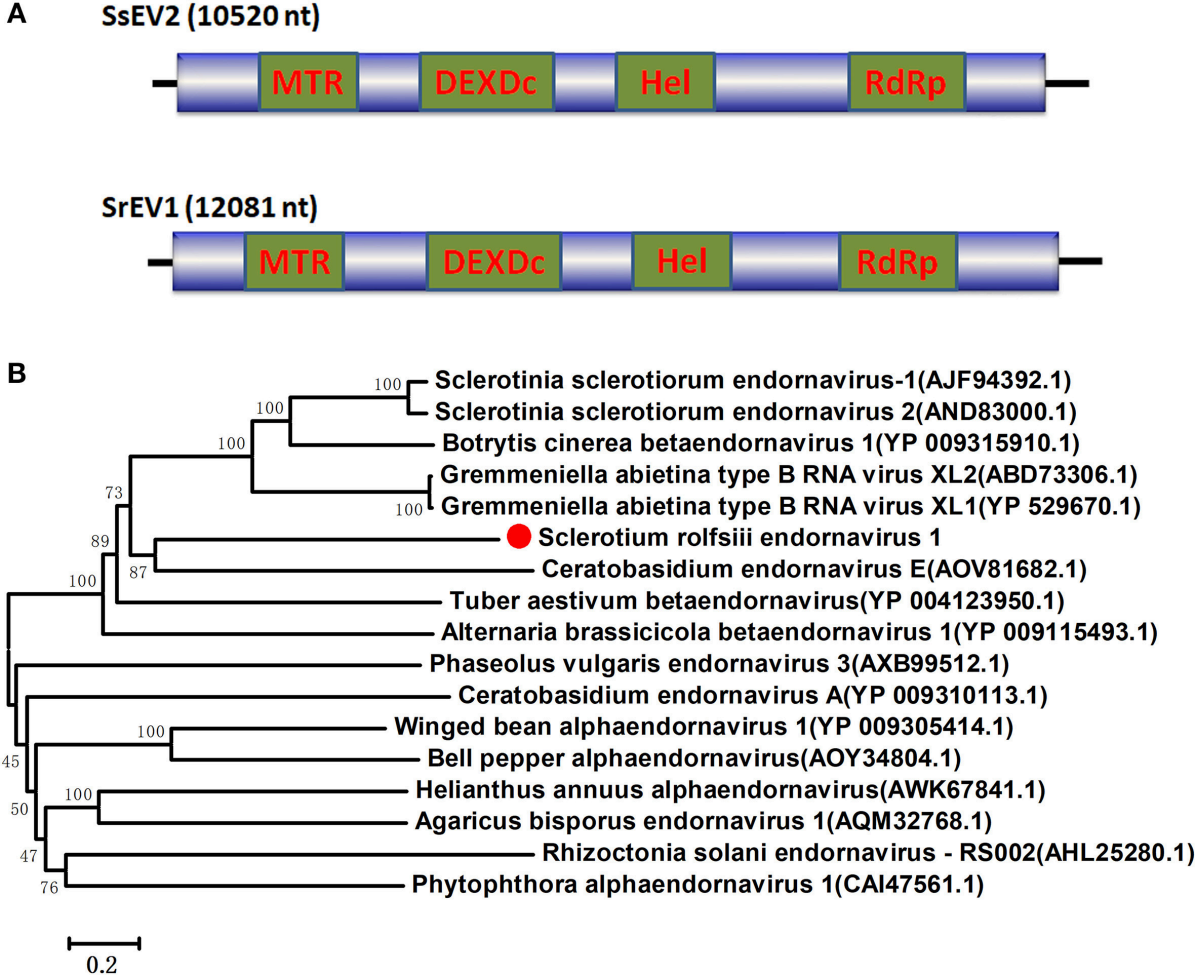
Genome organizations and Neighbor-Joining tree of the novel virus relating to confirmed and proposed members of the *Endornaviridae*. **(A)** Comparisons of the genomic organizations of the novel *Sclerotium rolfsiii* infecting SrEV1 and the identified member SsEV1 of the *Endornaviridae* family. The SrEV1 was completely sequenced and predicted to harbor a single larger ORF containing domains of viral methyltransferase (MTR), putative DEXDc, viral helicase (Hel) and RdRp, which were indicated in the ORF box. **(B)** Phylogenetic tree depicting the relationships of SrEV1 with other *Endornaviuses* were generated using the method described in Figure 1.

Several RNA sequences showed similarity to members of the family *Endornaviridae*. Viruses in the *Endornaviridae* family have linear ssRNA genomes that range in length from approximately 10 kb to more than 17 kb and contain an ORF encoding a single long polyprotein. The polyprotein encoded by endornavirus often includes conserved domains, such as viral RNA helicases and RdRps (Ghabrial et al., 2015). The gaps between the endornavirus-like sequences were filled by RT-PCR and assembled as an RNA sequence. In addition, the terminal genomic sequences were determined by RLM-RACE. We designated this virus as *Sclerotium rolfsii* endornavirus 1 (SrEV1) (Figure 3). The full-length SrEV1 genome was 12081 base, containing a single ORF encoding a 3997 aa polyprotein. Blastp analysis showed that this putative protein showed low sequence identity to the polyprotein of endornavirus, with *Sclerotinia sclerotiorum* endornavirus 2 being the best match at a 25% aa identity. Conserved domain searches revealed that this polyprotein contained the conserved domains of viral methyltransferase (MTR), putative DEXDc, viral helicase (Hel) and RdRp. Consistent with the homology search, phylogenetic analysis based on the conserved RdRp domain suggested that SrEV1 is a new putative species in the family *Endornaviridae*.

### Sequences related to “fusagraviridae”

**Figure 4:**
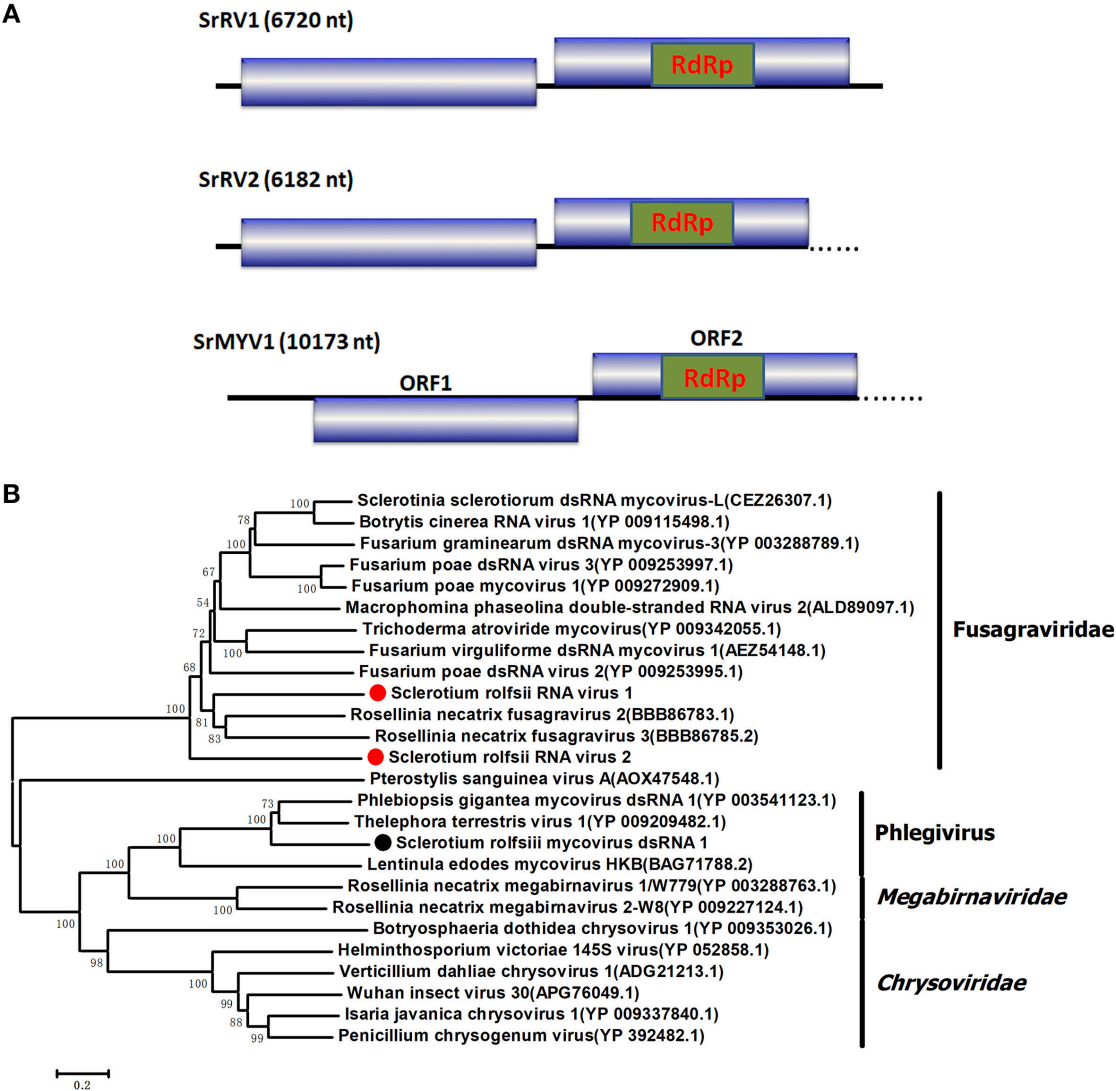
Schematic representation of the genome and phylogenetic analysis of two novel members of the family Fusagraviridae, SrRV1 and SrRV2, and a *Phlegivirus*-related virus, SrMYV1. **(A)** Genome organization of SrRV1, SrRV2, and SrMYV1. The three viruses were all predicted to contain two ORFs, ORF1, and ORF2, with their ORF1 encoded unknown proteins and ORF2 encoded the RdRp. **(B)** phylogenetic analysis illustrating the evolutionary classification of these three viruses was conducted using the Figure 1 described method.

Four viral sequences were associated with SsNLV1-like group mycoviruses, such as *Sclerotinia sclerotiorum* dsRNA mycovirus-L (SsNsV-L), Phlebiopsis gigantea mycovirus dsRNA 2 (PgV2), Macrophomina phaseolina dsRNA virus 2, Botrytis cinerea RNA virus 1 and other viruses that could be grouped into the recently proposed family Fusagraviridae (Figure 4). The gaps between the contigs were filled by RT-PCR, resulting in the assembly of two RNA sequences, *Sclerotium rolfsii* RNA virus 1 (SrRV1) and *Sclerotium rolfsii* RNA virus 2 (SrRV2), with lengths of 6,720 bp and 6,182 bp, respectively (dsRNA1 and dsRNA2). BLASTx searches showed that the partially predicted amino acid sequences of the two dsRNAs were similar to those of viruses in the proposed family Fusagraviridae. The sequence of SrRV1 contained two ORFs. The predicted amino acid sequence of SrRV1 ORF2 was similar to that of the RdRps of Rosellinia necatrix fusagravirus 2 at a 35% identity. SrRV2 also contained two ORFs, ORF1 and an incomplete ORF2. The predicted amino acid sequence of SrRV2 ORF2 showed 37% identity to that of the Phlebiopsis gigantea mycovirus dsRNA 2 encoding RdRp (CAJ34335.2). Hence, SrRV1 and SrRV2 likely represent new members of the Fusagraviridae family.

### Phlegivirus-related sequence

Several RNA sequences showed similarity to unassigned dsRNA mycoviruses, such as Phlebiopsis gigantea mycovirus dsRNA 1 (PgV1), Thelephora terrestris virus 1 (TtV1), and Lentinula edodes mycovirus HKB (Lev-HKB). The gap between the contigs was filled by RT-PCR, and the 5′ terminal sequence was also determined by RACE. All the sequences were assembled as a 10,713 bp RNA sequence, named *Sclerotium rolfsii* mycovirus dsRNA 1 (SrMYV1), as previously described (Figure 4). SrMYV1 contained a complete ORF (ORF1) encoding a putative protein with 1634 aa at the 5′-proximal end and an incomplete ORF (ORF2) that encoded 1243 aa at the 3′-proximal end. A BLASTp search showed that ORF1 was 38% identical to the hypothetical protein, and ORF2 was 51% identical to the RpRd of PgV1. PgV1 is an unassigned dsRNA virus with a 11,563 bp genome that contains two major ORFs encoding the hypothetical proteins and RdRp. The organization of the predicted SrRV1 ORFs was similar to that of a Lev-HKB clade including PgV1, TtV1, and Lev-HKB, and the SrRV1 sequence (10,713 bp) might represent a nearly complete genome.

### Putative double-stranded RNA viruses

Two viral sequences that showed similarity to Heterobasidion RNA virus 6 (HetRV6) were identified and designated as *Sclerotium rolfsii* unassigned dsRNA virus 1 (SrURV1) and *Sclerotium rolfsii* unassigned dsRNA virus 2 (SrURV2). HetRV6 is an unassigned dsRNA virus composed of a single genomic band of approximately 2 kbp encoding a putative viral RNA polymerase that is distantly related to Curvularia thermal tolerance virus (CThTV) and Fusarium graminearum virus 4 (FgV4) (Vainio et al., 2012). However, the genomes of CThTV and FgV4 contain two dsRNA segments (Márquez et al., 2007; Yu et al., 2009). Both SrURV1 and SrURV2 had incomplete ORFs encoding proteins that were closely related to the RdRp of HeRV6 (46 and 57% identity, respectively). Hence, the two viruses might represent unassigned viruses related to CThTV-like viruses.

### Viral genome sequence validation

**Figure 5:**
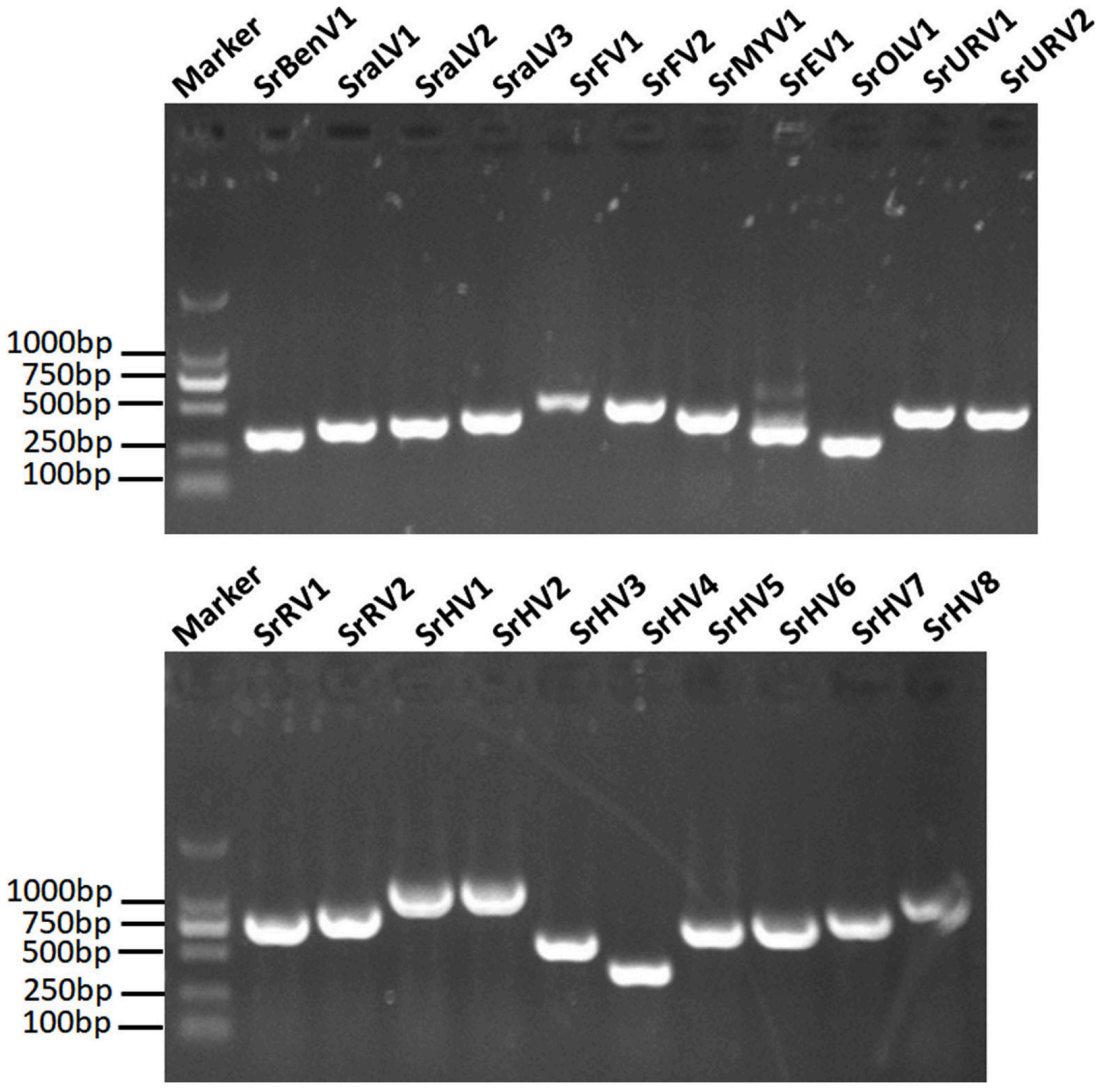
RT-PCR validation of the identified mycoviral sequences. The final assembled viral sequences were confirmed by RT-PCR using the primers that designed based on the obtained viral contigs and the total RNA template extracted from the BLH-1 strain of *S. rolfsii*. The marker was in 2,000 bp Ladder, and the other lanes indicated the abbreviates of these detected viruses which were detailed in the Table 1.

We used RT-PCR and Sanger sequencing to confirm the origins of these assembled mycoviral sequences. When PCR amplification was performed using reverse transcription products synthesized from the total RNA of the fungal strain BLH-1 as templates, products of corresponding size were obtained and matched to these viruses (Figure 5). However, when we performed PCR using DNA extracted from the fungal strain BLH-1 for all the assembled viral sequences, no products were obtained, indicating that the viral sequences identified were indeed derived from exogenous mycoviruses infecting BLH-1.

### Full-length genomic organization of SrHV1

In our previous study, we found that the host fungus BLH-1 was a hypovirulent strain deficient in sclerotium production. As mentioned above, the nearly complete genome of SrHV1 was most closely related to the *S. sclerotiorum-*infecting hypoviruses SsHV2/SX247 and SsHV2/5472 (Hu et al., 2014; Khalifa and Pearson, 2014), which are responsible for the hypovirulence and deficient sclerotium production in the host fungus. Thus, in this study, we further verified and obtained the full-length genomic sequence of SrHV1 by RT-PCR using primers based on the assembled sequences and ligase-mediated terminal amplification method. The sequence was deposited in GenBank under accession number KU885931.1.

The complete genomic sequence of SrHV1 was determined to be 16093 nt long excluding the poly (A) tail at the 3' terminus. In comparison, the nearly complete genome of the high-throughput sequencing-assembled contig of SrHV1 was 16050 nt, indicating 99.61% coverage of the SrHV1 genome. SrHV1 was determined to contain a 13,815 nt long ORF (nt positions 1185 to 14,999) encoding a 513.37 kDa polyprotein of 4604 aa residues that was predicted to contain the domains of a papain-like cysteine protease (Pro), RdRp and helicase (Hel), like reported for SsHV2/5472 and SsHV2/sx247. The 5′-UTR (untranslated region) and 3′-UTR of SrHV1 were 1,184 and 1,094 nt, respectively. The genomic organization of SrHV1 is depicted in Figure 2B.

### Molecular characterization and phylogeny of SrHV1

A homology search using the polyprotein aa sequences of SrHV1 showed significant identity with polyproteins encoded by members of the family *Hypoviridae*, with SsHV2 being the best match at 57% (coverage: 63%; E value: 0), followed by CHV2 (accession no. NP_613266.1; coverage: 63%; E value: 4e-52; identity: 23%), CHV1 (accession no. NP_613266.1; coverage: 49%; E value: 1e-53; identity: 23%) and Fusarium graminearum hypovirus 1 (FgHV1) (accession no. YP_009011065.1; coverage: 45%; E value: 1e-34; identity: 23%). Based on its sequence similarity to hypoviruses, we suggest that SrHV1 is a ssRNA mycovirus in the family *Hypoviridae*.

At the N-terminal end of the polyprotein, a putative Pro domain was detected. In this domain, two aa residues, Cys and His, which are necessary for the autoproteolytic activity of the polyprotein and strictly conserved among other reported hypoviruses, and Gly, which is relevant to the potential cleavage site, were observed (Smart et al., 1999; Yuan and Hillman, 2001). An RdRp domain containing the highly conserved aa sequence motifs that are characteristic of hypovirus RdRp domains was detected at aa positions 3101 to 3418. The RdRp of SrHV1 had the highest aa identity (74%) to that of SsHV2 (coverage: 99%; E value: 3e-147). Based on multiple alignments of the RdRp aa sequences of *Hypoviridae* members, a phylogenetic tree was constructed, and SrHV1 clearly clustered with SsHV2-SX-247, forming a branch distinct from members of *Alphahypovirus*. At aa positions 3872 to 4189, downstream from the RdRp domain, a Hel domain was identified. As previously described in other hypoviruses, the Hel domain of SrHV1 also contained three characteristic motifs of the Hel superfamily 2 (Hall and Matson, 1999): GKST, DExH, and QRxGR. The Hel domain of SrHV1 was most closely related to SsHV2, with a 74% aa identity. In addition, the SrHV1 Hel domain also showed aa identities of 27 to 30% to the Hel domains of other *Alphahypovirus* members. Phylogenetic analysis based on the Hel domains revealed that SrHV1 was grouped with SsHV2-SX-247 and SsHV1L, a clade related to but distinctly branched from members in the genera *Alphahypovirus* (Figure S1).

### Small RNA profiles of SrHV1 and SrEV1

To survey the presence and mechanism of antiviral immune response in *S. rolfsii* and to confirm that these putative viruses genuinely infect the host fungus, we assessed the presence of antiviral small RNAs in this virus-infected fungal strain. Because SrHV1 might be the candidate causal agent of BLH-1 hypovirulence and its genome has been completely sequenced, we focused on SrHV1. A small RNA library was generated from the *S. rolfsii* strain BLH-1 and subjected to deep-sequencing of small RNAs (sRNA) on the Illumina platform, resulting in 33 million reads.

**Figure 6:**
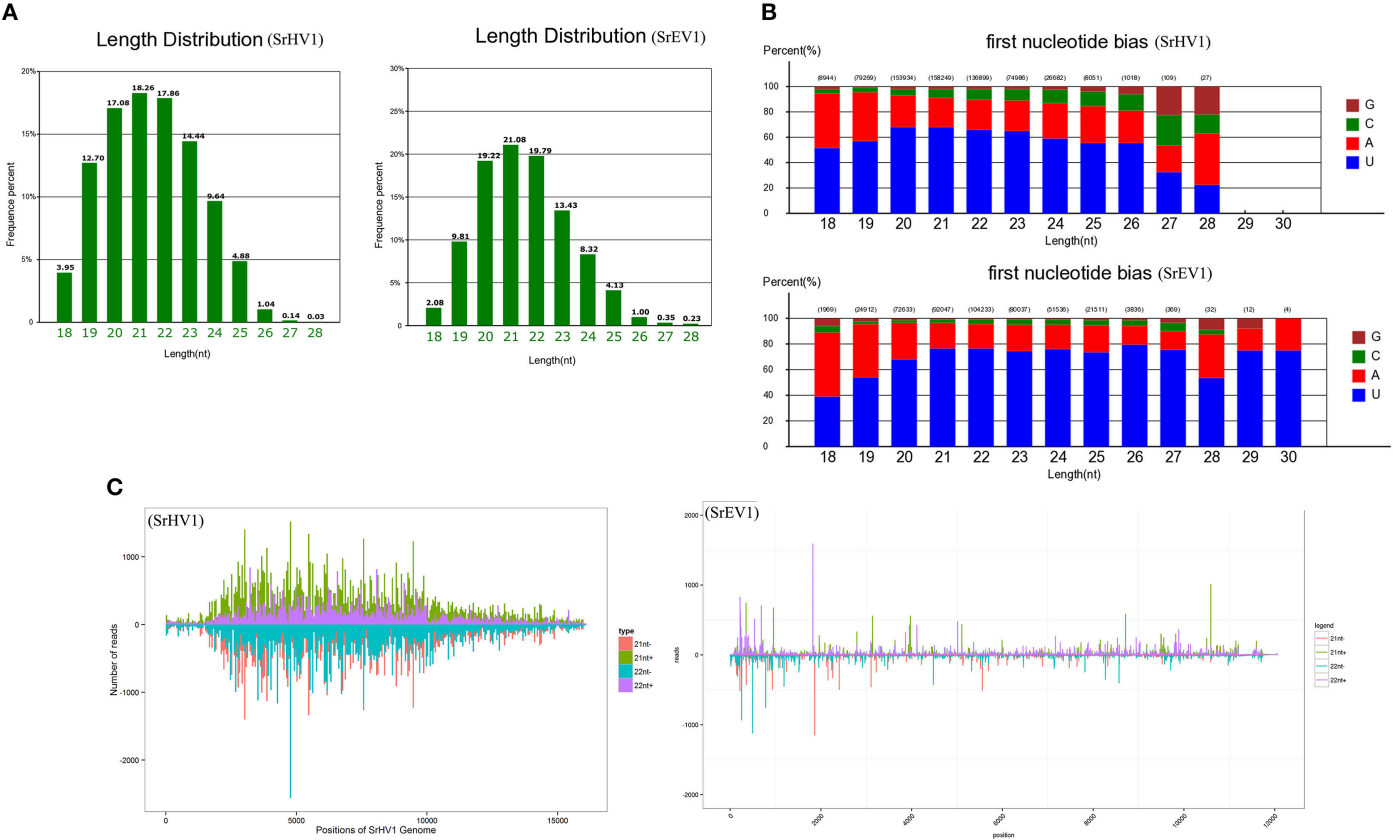
SrHV1 and SrEV1 derived small RNAs analysis. **(A)** Size distribution and abundance of small RNAs matching the SrHV1 and SrEV1 genomes. **(B)** 5′-terminal nucleotide composition among the SrHV1 and SrEV1-derived vsiRNAs. The x-axis represents the length distribution, and the y-axis shows the percentages of nucleotides constitute of G/C/A/U in the 5′-terminal of the vsiRNAs. **(C)** The vsiRNA profiles distribution among the SrHV1 and SrEV1. The vsiRNAs derived from the positive and negative strand of the viral genome were indicated above and below the line, respectively. For SrHV1, the 21-nt vsiRNAs arising from the genomic and antigenomic strands were shown in green and red bars, respectively. The 22-nt vsiRNAs matching the genomic and antigenomic strand were represented by purple and blue, respectively. For SrEV1, the 21-nt and 22-nt vsiRNAs arising from the genomic and antigenomic strands were indicated by green and purple bars, red and blue bars, respectively. The x-axis indicated the length of the SrHV1 and SrEV1 genomes, respectively. While the y-axis in SrHV1 and SrEV1 separately represented numbers of reads matching to the genomic and antigenomic sequences of the two viruses.

After filtering according to length, the remaining sRNA reads were mapped to the *S. rolfsii* genome, and the unmapped reads were then aligned against the SrHV1 and SrEV1 genomes. Abundant small RNAs were matched to the target genome. These small RNAs were mainly distributed from 19 to 24 nt, with a peak at 21 nt, and exhibited characteristics typical of Dicer-produced virus derived small interfering RNAs (vsiRNAs), which are produced from dsRNA intermediates upon binding to Dicer (Li et al., 2013) (Figure 6A).

Because gene silencing is mediated by argonaute proteins, which bind to small RNAs to induce the degradation of RNAs that complement small RNAs, the composition of the small RNA 5′ nucleotide is preferred by special argonautes (Wilson and Doudna, 2013). We found a clear preference for the U base at the 5′ nucleotide residues of both the SrHV1 and SrEV1 vsiRNAs of all sizes, especially for vsiRNAs of sizes 20-, 21- and 22-nt (Figure 6B). The 5′ nucleotide preference of these vsiRNAs was consistent with the high abundance of U bases in the SrHV1 and SrEV1 genome.

When exploring the polar distribution of SrHV1 and SrEV1-derived vsiRNAs, we found that the vsiRNAs in sizes of 21 and 22 nts originated from SrHV1 and SrEV1 were heterogeneously distributed along the sense and antisense strands of the virus genomes. Some regions of hotspots in, which produced more vsiRNAs, were located in the 5′ and middle genome regions of SrHV1 and in the 5′ region for SrEV1 (Figure 6C).

Small RNA data indicated that the vsiRNAs were generated from the genomes of SrHV1 and SrEV1 by Dicer, acting on dsRNA replication intermediates, and were bound by argonaute proteins; thus, we can conclude that the two viruses were targeted by the host gene silencing mechanism, an antiviral immune response exhibited by many fungi.

## Discussion

In this study, we characterized a complex virome from a hypovirulent *S. rolfsii* strain and identified diverse viral segments using high-throughput transcriptome sequencing. Some viruses discovered in our analysis were nearly full-length. These detected viruses might belong to viral species within the families of *Benyviridae, Endornaviridae*, Fusariviridae, *Hypoviridae*, and the proposed family “Fusagraviridae” and show similarities to other unclassified LeV-HKB-like and CThTV-like dsRNA viruses and unclassified alphavirus-like and ourmiavirus-like +ssRNA viruses. We conducted RT-PCR, genomic PCR and Sanger sequencing analyses using specific primers based on the obtained fragments to confirm the origins of our viral sequences. Positive amplicons were produced from only RNAs extracted from the *S. rolfsii* strain by RT-PCR but not by genomic PCR, confirming that the viral sequences represented non-integrated RNA virus sequences. To the best of our knowledge, this is the first report of a comprehensive analysis of viral diversity in a hypovirulent *S. rolfsii* strain. Thus, our results represent a step forward in exploring *S. rolfsii* mycoviruses and provide insight into screening the mycovirus potential of *S. rolfsii* controls and understanding the mechanism of *S. rolfsii* hypovirulence.

Among the identified viruses, SrHV1 was the fully cloned putative hypovirus relating to members of the family *Hypoviridae*. SrHV1 shared the highest sequence identity to the previously reported virus SsHV2, which was shown to induce hypovirulence of the plant pathogenic fungus *S. sclerotiorum*. SrHV1 shared the same genomic organization, genome size and phylogenetic grouping as two SsHV2 strains but was distinct from other members of the *Hypoviridae* family. Currently, two genera, *Alphahypovirus* and *Betahypovirus*, which range in size from 9 to 13 kb, are classified within the family *Hypoviridae*. These two genera exhibit several differences in terms of their genome size, organization and gene function, as alphahypoviruses range in genome size from 12.5 to 13 kb and contain two ORFs, whereas betahypoviruses have a smaller genome size of 9.1–10.4 kb and consist of a single large ORF that encodes a polyprotein. A UDP-glucosyltransferase (UGT) domain is present in the proteins encoded by betahypoviruses, while alphahypoviruses lack this domain. Hu et al. (2014) proposed that in addition to *Alphahypovirus* and *Betahypovirus*, a third genus, Gammahypovirus, should be established to accommodate SsHV2. Here, the identification of SrHV1, which should be classified into the same genus as SsHV2, further validated the rationality to establish a Gammahypovirus genus in the *Hypoviridae* family and indicated the diversity of viruses in the *Hypoviridae* family. It is worth noting that the SsHV2/SX247 alone could likely leads to hypovirulence and complete loss of sclerotia production of the *S. sclerotiorum* host (Hu et al., 2014); thus, we hypothesize that SrHV1 induces hypovirulence in this *S. rolfsii* strain. However, as described in this study, the *S. rolfsii* strain BLH-1 was coinfected by a diverse range of mycoviruses, and we could not exclude the possibility that other mycoviruses also induced host hypovirulence or acted together with SrHV1. To elucidate the cause-and-effect relationships between individual mycoviruses and their hosts and the possible interactions between these coinfected viruses, further research with respect to the construction of full-length infectious cDNA clones and associated experiments is needed.

Studies on mycoviruses using high-throughput sequencing have recently been efficiently applied to the discovery of fungal viruses (Bartholomäus et al., 2016; Marzano and Domier, 2016; Marzano et al., 2016; Deakin et al., 2017; Mu et al., 2018). In contrast to other dsRNA extraction and cloning methods, this approach can provide viral information for a diverse set of viruses belonging to different taxa regardless of their genome types and viral titers. For example, by deep sequencing, 18 phylogenetically distinct RNA viruses and 8 ORFans were identified from the mushroom fruitbodies of *Agaricus bisporus* Deakin et al. (2017). Marzano and Domier (2016) obtained virus contigs from a collection of fungal species from *Colletotrichum truncatum, Macrophomina phaseolina, Diaporthe longicolla, Rhizoctonia solani*, and Sclerotinia sclerotiorum using RNA_Seq analysis. Their analysis also revealed novel types of mycoviruses in the families *Benyviridae, Ophioviridae*, and *Virgaviridae*. Recently, deep sequencing was also used to identify the diversity of mycoviruses within a fungal species collected from diverse geographic regions, potentially providing overall knowledge of viral diversity, evolution and ecology on a species scale (Arjona-Lopez et al., 2018; Mu et al., 2018). In addition, when high-throughput sequencing is used for virus detection, the enrichment step, which might bias detection, is not necessary. High-throughput sequencing was used to detect mycoviruses on the phyllosphere and the arbuscular mycorrhizal fungi colonized in the roots (Ezawa et al., 2015; Marzano and Domier, 2016). By deep sequencing extracted dsRNAs, different mycoviral species were shown to coinfect a *Fusarium poae* fungal strain (Osaki et al., 2016), as illustrated in other fungi, such as *Rhizoctonia solani* (Bartholomäus et al., 2016). These studies indicated that the known mycoviruses characterized thus far might represent only the tip of the iceberg of all mycoviruses in nature, and some mycoviruses might have been overlooked by traditional PCR-based detection approaches. Because screening viral sequences principally depends on their homology to known viruses in the database, the recent explosion of viral databases that contain enormous novel viral genome information (Li et al., 2015; Shi et al., 2016) makes isolating and identifying new viruses in a larger host range and geographic distribution more feasible.

Like other eukaryotes, fungi can also use RNA interference (RNAi) as a primary defense against virus infections, as described commonly in *Cryphonectria parasitica* infected by the CHV1 (Nuss, 2011). When the viral genome is targeted by the host RNAi mechanism, an overlapping vsiRNA population derived from the virus can be produced. Like in plant and animal hosts, 21 nt sRNAs represent the primary class size of vsiRNA populations in mycovirus-infected fungi (Yaegashi et al., 2016; Donaire and Ayllón, 2017). For example, the Magnaporthe oryzae virus 2 (MoV2) infection in Magnaporthe oryzae results in the production of 21 nt vsiRNAs (Himeno et al., 2010). In our study, the sizes of most vsiRNAs that mapped to the SrHV1 and SrEV1 genomes ranged from 19 to 24 nt. The 21 nt vsiRNA with 5′ U bias was the most abundant vsiRNA species, which was in accordance with previous reports in fungi and suggested that SrHV1 and SrEV1 are targeted and processed by the host RNAi. The similarities in pattern of vsiRNA composition between our tested SrHV1 and SrEV1 with some other mycoviruses and plant viruses indicated that the biogenesis of vsiRNAs in some RNA silencing pathway, in some case, is conserved among different kingdoms. Of course, different plants might have various siRNA biosynthetic pathways. It has been suggested that DCL4-dependent 21-nt vsiRNA synthesis, is the first antiviral defense in Arabidopsis (Moissiard et al., 2007), whereas DCL2 often acts as a DCL4 surrogate for 22-nt vsiRNA generation (Deleris et al., 2006). The percentage of 21-nt and 22-nt vsiRNAs relies on different virus-plant host associations. Therefore, DCL4 and DCL2 should work redundantly or synergistically for systemic antiviral silencing. In fungi, RNA silencing against mycovirus infection has been fully characterized in some mycovirus/fungus systems such as in the fungus *C. parasitica, Colletotrichum higginsianum*, and *Fusarium graminearum* (Segers et al., 2007; Campo et al., 2016; Yu et al., 2018). In the CHV1/*C. parasitica* system, the dicer-like (dcl-2) and argonaute-like genes (agl-2) have been demonstrated to be necessary for antiviral silencing (Segers et al., 2007; Zhang and Nuss, 2008; Sun et al., 2009). However, the presence and contribution of each *S. rolfsii* Dicer protein in the biogenesis of each size class of mycovirus-derived sRNAs is unknown. The virus-derived small RNA from SrHV1 and SrEV1 showed Dicer-mediated siRNA profiles, indicating a gene silencing response of the host against these viruses. In the present study, the 5' nucleotide residues of vsiRNAs derived from SrHV1 and SrEV1 all tended to be U residues instead of A, G or C, indicating that the vsiRNAs with 5′ U might be enriched by sorting into special AGO-containing complexes after dicing. In *Arabidopsis*, different AGO proteins specially recognize the 5′ preference base of vsiRNAs (Mi et al., 2008). As reported in *N. crassa*, argonaute-like proteins (QDE-2) are biased toward associating with sRNAs with 5′ U terminal nucleotides (Lee et al., 2010). We speculate that argonaute-like proteins that are conserved in fungi and plants play a dominant role in the posttranscriptional gene silencing (PTGS) of *S. rolfsii* and other fungi.

The p29 of CHV1, a papain-like protease that shares sequence and functional similarities with the helper-component proteases (HC-Pro) of potyviruses, a symptom determinant, has been shown to act as an RNA silencing suppressor (RSS) in the fungal host (Segers et al., 2007). Marzano et al. (2015) speculated that SsHV2 also contains a similar symptom determinant because deletions in the SsHV2L and SsHV2/5472 genome regions lead to altered sclerotia production. In our study, SrHV1 was identified as a novel member of the family *Hypoviridae*, like CHV1. In addition, the *S. rolfsii* strain BLH-1 was defective in sclerotia production, and SrHV1 showed an intimate affinity with SsHV2, both of which contain the papain-like proteinases that are closely related to the CHV1-encoded p29. We presume that SrHV1 might also have an RSS that functions to resist host antiviral defenses and determines or participates in host hypovirulence actions, such as growth, virulence, and sclerotia production. However, to validate this hypothesis, additional gene function characterization studies are needed.

## Materials and methods

### Fungal isolates, growth conditions and RNA preparation

The fungal strain BLH-1, originally isolated from a southern blight disease-infected *M. cordata* plant in the Hunan province of China, was maintained on potato dextrose agar (PDA) at 27°C. Total RNA was extracted by mycelium harvest using the RNeasy mini kit (Qiagen, Valentia, CA) and quantitatively evaluated using the NanoDrop ND-1000 spectrophotometer (NanoDrop Technologies, USA). High-quality RNAs were subjected to RNA-seq. Sequencing cDNA library was constructed from poly(A) selected total RNA, using the TruSeq™ RNA Sample Prep Kit (Illumina, RS-122-2001).

### Bioinformatic analyses and virus genome identification

Bioinformatics analysis of RNA-seq data was carried out using the CLC Genomic Workbench software package (CLC Bio-Qiagen, Boston, MA). Reads were *de novo* assembled with Trinity (Grabherr et al., 2011). The resulting contigs were compared to the *S. rolfsii* reference genome to subtract the host-derived sequences and then compared against the non-redundant viral reference amino acid sequence database available in NCBI using the BLASTx and tBLASTx programs. Multiple alignments of amino acid sequences were conducted using the ClustalX program (Larkin et al., 2007), and phylogenetic trees were constructed by the neighbor-joining (NJ) method using 1,000 resampling bootstraps in MEGA 6 (Tamura et al., 2013).

### Viral genome sequence confirmation

To confirm that the viral sequences were determined from mycoviruses infecting the fungal host rather than from sequences integrated into the host genome, RT-PCR and PCR assays were separately performed using templates of total RNA and DNA extracted from the fungal strain BLH-1 and primers designed based on the *de novo* assembled viral contigs. The amplicons were analyzed by agarose gel electrophoresis and sequenced by Sanger sequencing.

### Amplification of cDNA ends

To completely sequence the genomes of some of the mycoviruses, sequence gaps not covered by the transcriptome sequencing assembly were filled by RT-PCR amplification using designed primers based on the obtained viral sequences flanking the gaps. The 5′ and 3′ terminal sequences were elucidated using a ligase-mediated terminal amplification method as described previously (Zhong et al., 2016b). All of the PCR products were cloned into the pMD18-T vector (TaKaRa) and sequenced, and every base was determined at least three independent times.

### Small RNA deep sequencing and analysis

The pooled total RNA from three *S. rolfsii* strain BLH-1 replicates was extracted. The small RNAs were purified from 17% denaturing polyacrylamide gels, ligated with 5′ and 3′ adaptors and then subjected to small RNA library generation and Illumina sequencing.

After the adapter sequences and low-quality reads were removed, the data were subjected to bioinformatics analysis, and sequences 18–30 nt in length were extracted. To identify SrHV1 and SrEV1-derived vsiRNAs, the clean reads were respectively mapped to the SrHV1 and SrEV1 genomes using the BLAST search function. vsiRNA analyses, including the nucleotide size, composition of the 5′ nucleotide and polar distribution, were performed using Perl scripts and Microsoft Excel as previously described (Wang et al., 2016). The data from the raw sRNA reads were deposited in the NCBI Sequence Read Archive under accession number SRR7754484.

## Author contributions

JZ and QZ: conceived and designed the experiments; JZZ and JZ: performed the experiments; JZ, BDG, and HJZ: analyzed the data; JZZ: Wrote the paper.

### Conflict of interest statement

The authors declare that the research was conducted in the absence of any commercial or financial relationships that could be construed as a potential conflict of interest.
