# Supplementary material for: Diverse, Novel Mycoviruses From the Virome of a Hypovirulent Sclerotium rolfsii Strain
Source: Front Plant Sci. 2018 Nov 27;9:1738. doi: 10.3389/fpls.2018.01738 (PMC6277794; doi:10.3389/fpls.2018.01738)
Supplement: Supplementary file 1 [file Data_Sheet_1.ZIP › Electronic Supplementary Material.docx]

**Fig.S1**


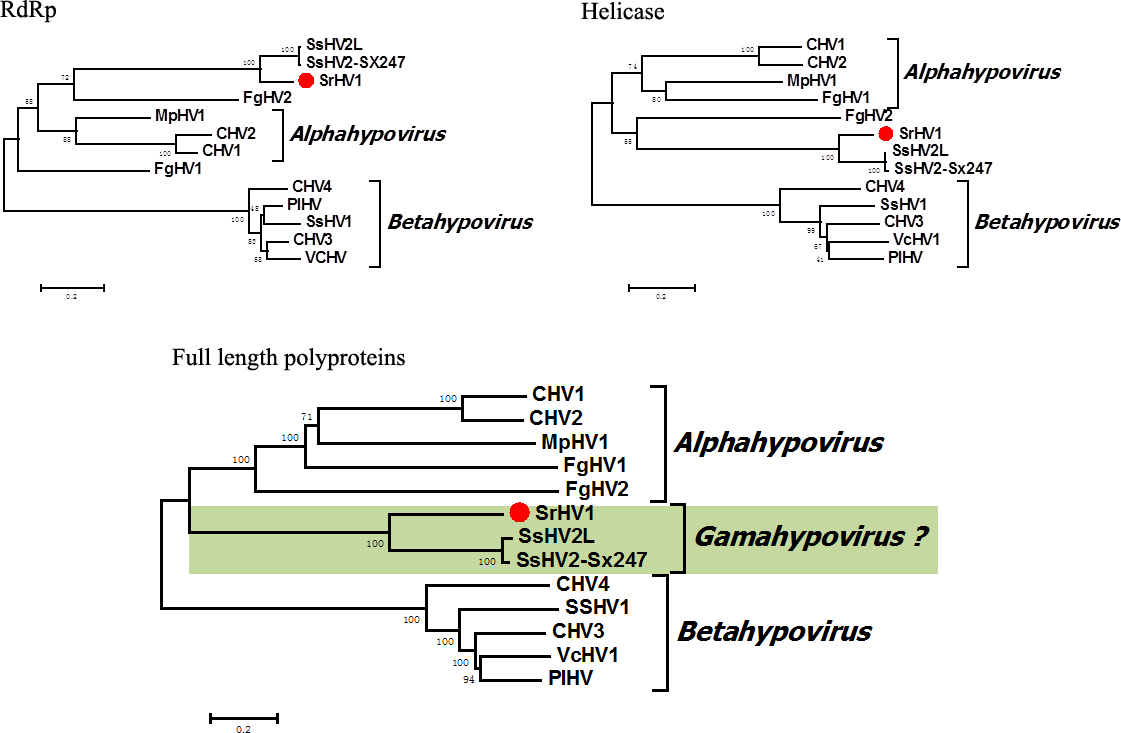


**FIGURE S1 Phylogenetic trees constructed based on multiple alignments of (A) RdRp, (B) Hel domains and (C) the full length polyproteins of virus SrHV1. The aa sequences were multi**-**aligned and subjected to phylogenetic analysis in MEGA 7 using the** **Neighbor-Joining algorithm, with a 1000 bootstrap replications.**

**Nucleotide sequences of the obtained viruses**

>SraLV1

CGCCGTGTTTACTTCAACCCTTACGCCTCTGCTCACGTGGTTGGTCCGCGAAACATGGGTCGCAACCGCCACCGCTCCCGTCCTCGAGGAAAGATTCCTCGGCGGACACCGTCAACATTCCCGGGATACGAGAAAGACTTTTCCGACGCCCGCGTGCCCTTGGCTAGTCCCGTCAGTGAGCACGTGTTCACTCGCATCGGAGCGACTTTCTTCGATTTCCCCCGCCTCTCGTGCCCTGCGCGCAAGATTCCTGGCCGGGTTGTTCGACAGCACAAGAGAGACGCAAGGACCGCCGCCAACTTCGATGCCTTTGCCGACGGCATCGCAGTGATGTTCGCTGCTGCTGACGCCTTCCTCCCCGTCTTGCCTGCCCCCATCAAGGTACGCAGCACGAAACGTCGGGCCGCCATTTCTTTCCCTTCGCTGGAAGTGGAACCGACTACGCGCGCAACACGCATTCGTGCGCCTGGTGATTGTTGGCGCCGACTCGGGTATCCCCTTGCCGAGGACATCGAAGCCGCTTGCGATGACCCGGAGAACGTTACCGTGTCCACCGTGCTTGATGTCGTCACAGAAGATGCTTGTCACGAACTGAGTTGGCAACCACTCATCTTCTGCGAGCGTCAAGATGATGGCGACTTTCACATTGAGCGTGTCCATTGGGTCAATTCGCGCCGCTTCCGCATGATGACCGACGAAGACTTCGACACCATCGGAGGGATAACGATCTGGGAGTTCCTTGCCCTTATCCGCGATCACCGTGAATGCCTCGTCGCGAGTGGACCGCCGCGCAATTCCGACGGCGAGCAACCTAGCGCTGCCTTGCTTGAGCCGACCATCTCTACTGCCTTCGCTGCAGAGCAAGGTGTCGAATATGGCATCCCGGCCGTTTGGAACTGGCTCAAAGCGCACGTGCCCGACCAGCCGGTGTACGCCCTGGCAACTTCGCAGGAACCCTACCCGCAACCTGGTCCTGTACCGCCGCCCGTTGCCGACCCCCTTCCGCAGAATCCGTGGCCTGGTGGACCGCCACCAGATTGGAATGCGCCTGGCCCGGTTGTCCCGCCAATCGACGGGCAGATGCCGCCTCTTCCGCATCCTGCCCCGCCGGTCGTCACGCCACCGGTCGTCGTGCCTGGTCCGGAGCCACCCGTTGTCATACCGCCGCCCCACGACCCTCTACCACCGGTTGTGCCTCCGGTCATCCACCCAGGGGAACCACTCCCCCCGCTGCCGCACAACCCGTGGCCTGGAGGTCCTCCACCGCCCATCCACGGGAACCCGGGACCTCTAATCCCGCCAGGTAGTGGTGCTCCGCCGGTTGACAGCTTGCCGCCGATCCATCCTGACCCGCCAGTGATCCCGCCGCCGCATGACCTGCCGCCGCCCGATTTGCCGCCTGTTGACCCTTCGTTGCCGGTACCGCAACTCCCTGGGCCTCAATTCCCGCAGCCGATTCTTGATCACCCAGCCCCGCCATTCGGACACATCCCGTTCGCCGATGGCCCGCTCGGGGACTGGGTTGCACCTGCAGTCGAGCTGCTCAATCGTGCTGGTCACACCGTGCTGTCCGTTGTGCACGATTTGTACCATGGAATCGCCGTCCCTGCCCCGCTCATTATGCCTGCTCTACGTCTCTTCGAATACTATCGACAAGACTTTTACCAGCAGCACAACCCTGGGCGCATTTACCGCGGTTCACGTCGAGGTGCCGCAAATGCGAGGTACAACGAGGACTTTGATCTGGTCATGCATGGCGCTGCGCGTGCCCTACCAAGCATCGCTGGTAACATTGTACTCAGCATCGATCCATGGCTGGGTGCCGCACGTGATCAGCTCGGGCAGTTTTCCGATTCAGACCGCGGACAGAACAGCGTGATGACACTCGCCATCGGTGAAGTCAACACCGCGCATCAAGCGAGGCTGGCTGCGCCGCAATTGCGAGTCGCGGCCAATGCACCCGAGTCGTCTGTGCGTGAATTGGCCAATGCTTTCCCGGAGTTTCGAGTCGTAAAAGTCTCGACGGTACACCCGCACGGCGTTTCGACGGCCGTTCGTTGGGCGCTGAGCGTGCTATGTGTCTCACACCTTCAACGGCGATTTCCTGGCGTCCGCGTCCGAGGCATTGGTTTGTCACCGCAACAGGCAGCACAGTACCCGCAACTCGTCCACAATTGCGGGCCTGAGCTCACAGGACGCGACGTGTATCGACACCGCCATGCATATGACGCGATCGCGAATGCAGCGTTTGCGCAAATCTCGCAGAACCACAGGTTCCAGGACTGCCACTCGAACTGTGCTCCAGTCGTCGTGGCGCCTTTTTCTTGTGGCGACATCTCATTGCTTGATTTCGCAGTCGAGTGTATTCAGTCCGGTGTTCACACGGCTTACGTGATGCTCAATTTGCCGGTGGTGTTCTTGGACAGCCGCATCCGCCGCTTCCGCGACTTGGAAAACGGGTTCCTCTACGAGCGCGAGGGTGATTACGTCGATATGTACTTTACTGGTGGCGCATCTGCCGGATATCGCAACAGGTACGACACGCTTCTCAGTTGGTGCAGCCCTGTCCCGCGCATCGACGGATGGCACATTTCAGTCGAGGAGTTGCGCCGGTTCGGATCGACTTACTTGCTGGAGGTTCGACTCGCACCTGGCCCGCAGGAGGTGATGCCCACATTGTTCAAGTTGGCGATGGAGGAGTTCATCGTGCTGCCGAAGCTCATGCCGTATTGGCGACGCGACGATTCAAATACCGGGGATTTCGCCATTCCCGCTCGGCGTTTCCGTGCACTCGTATCCTTCGTGTCCACGATCGAGCCAGGCAAGGTGCATTTCGAGCCAGTCGCCAATAAACTTCGCGGTCAGCTTGGCGAAGTGCGAATCGGCAAGACCGTCATCGAAAATCGTTGGGACCTGGAAGTTGACGAGTTCTACTCCGTCGTTGGCCATGCGATAATCGCTCATGCTGTGCATTCGCGCGACTATCAGGCAACTGTTCCTCGTGTGCTCGCCGCTGTCACGAAGGGCTTCGATCGGCACTCGACTAACCCGTTCTTGCGGTACGCCACGTACCTTGGTGATCTGTTCACCCTGCAGCTGGCGAAGCATCGCAATGCGATGGACCGGTCACCGTTCGAATGGCTCCTAGACGCGATTACTGGAACTGGGATCAATCACGGGCAGTTTTACTCGCCTTACGTGCCGCGCTCAGAGCACCGCGTGATCAACGATCGGGACTTCGTCGCTAACATGACACTCGCCCAATTGCCGAAGGATGTTCGTCGAGTTGGCCAGCTTACTGCGTACGCCGTTTCCGGAGCGGTCGACTACGTGCAGGAACAATTGTCCCTTCTTCGGCGTCAGCGGCGCATTCCTGTTGCCGTCGCGCCTGCGATCGTGCAGCCCGCACCCGCTCCAGAAAGCGTTGCGCCGTCACCCGCAGTTGCCGCAGTCGATGCTCCGACCCTAGTGAGGGTCCGCAATCCCGAGCTCGTGCCGTTGCCGCCACCCGACGAGGAAGAGATCAACTACTTCTTCGATGATGACGATTCTGCGCAGGGCGCTCCGTCTGTGTACTTGTGGGGTTCCGAAACGTCCGACGATGACGCGCCTGCCGGCCCGGAAGAGCTTCCTCCAGGACCCTCTGTCCTGGGGGCTCCTGAAGCGGTCGTCGAAGAGCAAAGAGCCGCGCCTGGGACTGCCGTATCACCTACGCAAGTCGCATTGCCTCCTGAGACTCCACAGGAACACGAACTGTTCGCTGACTTCTCAGGCATGTCGGCATCGTCCGAGTCGTCTGTGATTCCGATCGCACCTGGCAAACCATCTGGTGCCATTGACGACGGTGTCCGTGAGCTGTTGGATCGGCGTGGTCAGCCTGTCACACAGGCGAAGCTCCCCACCATCAGTGAAGGGGACCTCTACGACCGATTCACGGCGGCGCCGTTCCGGCCTAGTGCAGCACTTGCTCAGCGGTTCCAGACGTACTCGGAAGCCGACCGCATCATGCGTAAGGATTATCCTCGTCCACTCGACATCACACGGCAGATCAGCACGGGACGATTCGATATGCTTTGTCCTGATGCCGGGTTGTTCGACATGATCTCCGAGGCGATGAACTTGCCTGTCACCGTGCGTCAACAGGAATTGCCCTCTGAGGATGCCATATTCCGCACCATGGGGATCTCCGCTTGGGACGGTGGCAACGCGCTCCACAACAAAGTGCGCTCGTTCTATGGCAATCCGGCGACCCGCCACTCTGCTGTGACTGTACCATCGCTCCATCTTGATGGCGTCGCCGGCAGTGGAAAATCGACTGCATTCCGAAATTGGGCCGTCAAGTACGGGTACAAAGGAGTCGTCGTCGTGCCCTCCCACGCCTTAAAGGAGGAGTGGCGCAATGCACTCGAGGGAAGCCAGATCCGTGTGACCATGCAGCACGTTGTCTGCACATACAGGAACCCTCACTTCGTCGCGATCGATGAGTGCTACACGTTTGCTCGCGTACACCTTGAAGCGTGGGCGCGGTATGCCACATCCGTCGGAGCGAAATTGGTGCTGCTCGGTGATTCGTTGCAACGGGCCAAAGATGACACGGGGTCCCTTCCGTCAATTACCGACCCGTGGTTCACACGCCGACGCATTCGTCTCACCATCTCTAACACGATGGGGTGGGACGCCATGCATCTCATGCATGTGACACACGGGCTGCCTTTGACTAAGGGTTCGCCGTTCCAGACGCGCAATGGGTACCGCCGGACGATCATGATGTACCAGCCGACGAAGATCTCGCTACCGCAAATCGACTTCCGCGGATGGATGTTGACCAAGCCGCGAACATCGGCCGCTGGTGGTGAATTGGACATCTCGCGCAGCGTCACGCAGATGCAGGGGACACGTTGCGCTCAACACCTCCACGATTTCACTCTCTCGTTGAGTGGAGGACTCTGGCAACGTGGCATGTTGGGAGCGACGACTGTCTTGATCTCCAGACATACGACACGCCTGGTCATCGTCGCTGACATTGCGATCATGGAGATGGTCTTCCCGGGCGTCCATTGGGACGATTTCATGCCGGTCAACGGCCTCCGCGAAACACGTCTACTCGGGCAACGTCAAATTTTCCCGTTCGATCTGGACATCTTGCCTGTCATTCGCCACCTCGATAGCGCTGCCCACCCAGAAATCGAGTTTGCGGGTCTCCCACTCGTCTCGTCCCGGCGCACCGGTGAAACACTGGCTTCCTTTGCGTCTACGTACGAACCACCGGTTCGTTCCGACTCCGAGTACTGCGTCGCCCCCGAGCGCTTTGAACTGCAGGGCTTCGTTTACGGTCGCGTCAACTTCGATCTCGCCAAGGACCATGAGCACGCCATCGACTTCGTGACTTCGGGATCACGCGGACTCACCAGTGTGGGCGAGATCTCCTACCCGGTCACGCGTACAGACGTCCGCAATGATCTCGATGAGGCGTGGAAACTGGCAGACATTCAGGTCTCGCAATCGCGGTTCGAAGATTTGCGGAACATCTGCGAGAGGCAATTCCAGCAGAACAAGTCCTGGACGACCGCTGGAACGATTTGGACAGATGCGGCTGCCATTTTCCGCCGCTTCTGCGCCTGCTTCTTGGCCGACGAGCGGATGCAGCGAGTCGATCTGGAAGACTTTTCTGTCCGATGGCTCTCTACTCGCGCCCCTGCCTTTTTGCAGCGACTCGCCGACGAACCGTTCTCGGAAACAGCTAGATCGGTCCGGTTCCACGCGTTCCTCAAGCAACAGGTGAAGTACAAGAGTACGCCTGGGTTCCCTGCGAGCGTCAATTACGGCCAGCAAGTCATCGCCAACGAGGCACCCTACAGTGCGCTCTTCGGACCGCCTGCACTCGCGGCGTACACGGCGACGCTCGCTGCGATACGCGATGACTATGTGCCGGACATTGGGTTCTCCGACGACGAACTGTCCGAGTACTGTCGACGTCGAGGGATGTTGGAGCGTCTCTCAGGGCCAAACATGCAAGTCGATGTTGAGAAGCAAGACTCATCGCACACGGCCGAGCACGTCCTCGCTTTCTGCATGTGGCTGGATTTCCTCGGATTCGATCCACAGCTTTCGGAGCTTTACTTCTCTCACTGCTCCTCTTACGCGGTGGTGAGTCAAGCTGAGCCGCAGTTGTACCGCGGGCGCATCTCGTTCAATCTGGCGTCCGGTGACCCGTTTACGCTTCTTCGGAACTTCTTCCAGATGTCCTCGACGTTGGCTTGCCGGTATCGCGGCGTGCGACGAGCATGTGGTGTGCAAAAGGGCGACGACCTGATCCTGGACGTCCATTTGCGTGATCAACATCGTCTGGCGACCCTTCCGGCAATCGCACAAGTTCGGTTCAAAGTGGACTACGATAAGCCTGCATACCATGCGGGTCGTTTCGTCACTCCACTTGGCTTCTTTGTCGACCCCGTGCGCGCCTTCGCCAAGCATTTCGCGCGTGTCTCCGACGTAAATGTGCCAACCGCCGAGCTTTACCAGAGCTACATTTCTCGGGCTACAATGTACCCAGCCGAGATCCAGCGGTATTTCCGGGCCATTCTGCGCGTCACATACGAGGATTACAACGACGAAGAGATCGACCTCATCCTTCGGACCGTAGCCCACCTGCGCGGCAAGCGATTTTTTGAGGAGTACTCCACACCGCGATCCCAACCGCGGATGCGGGTCGAGGCCCCGACGGACTGCGCGGTCTTCGTCGCAAAGTTCTTGATCCCCGGTCGTCCTGCGCGCTTCTATCACCAATTCCGCGGCCGCACTGCCCTCGGGCTCCAGAATGTGTTCCTTCGGTACGGAATTACTTCCGAGATCTTCCCTGATTCGACGTTCGTCCCTCCGCATGCGGATGGTCTCATTATCACTCCTTCACACACGTTCTTTATACGACCTCCCAAAAGAGTCTAGCTATGACCTTTAAGAAGTTAACGCCTCTTAAAGTTATGTCTGTCTCTCGCACTTTCCTTGTTTCCTCCCCCGCCGTTACAACCGCCGCCCGCGGCGTTGACGTTTCCTTGCGTAATGTGGCGATGGTGGCTAACCTTCGCACCTTGTTCTCTAGTGTGAAAATCGCCGAAGTTGCTGTTTCCCTCGTAACATTCATGAATGATGATGTTGAGTTGGTTTTCGGTCTCGTTCCGTCTACATTGTCCATGCCGACGGTCGTGGGTGCTGTGTACGAAGTACCTCACCAAAATTTGGTTGTTTCTAATTCGACGACTGCAATTGTGCATACTGCAGTCTTCTCTCAAGCAACAACGCCTGGTCTCGAATGGGACATGGCGCAAACAGCCATAGCCGGTGGACATCCTGTCTTCCGAATCATGCACGCGTCCGACCTTGCCGAAGGTGAGGACGTTAAAGTTGTCGCTCGTGCGACAGTCCTTGTGACTGTCACTTGCGACGGCACGGCATTCGGTGCGTGACTGTTGCAGGCCTTCTTGATGTTTCTTTTTTTTCATATCGCCAATTTATGCAAGCCCCACGTCGCGCCCCACCAAAAGTCTGGTTAA

>SraLV2

CATGGTCTGCTGGGCCTCGCGACCCCTCCTAGGGACGCGCGACCAGTATGTAGTTTATAATCTTTGGTGGCCTGATGATTGGTTCCACATCATCAGTCACGAGCCGGCCGATCTATCGCGTTACGACTTTCCCCTTCGCCCTGTGCCTCGCACATCGCGAACCCATCGCAAGAAGTACGCGTGTGCTCCCTGGACCGCTCCGCCACCCCCCCCTCCTGCCCCCAAACGCCGCCCGCCGCATCGATCTCGTCGTCGATCCACGCAACCGCGGCGCACTCCTCTAGCCGATTTGCCCGCAGAACATGGGCTGCAGCAAATCACTTACCCGGTTATACCACCCGTCCGACGCCTCAACCAGCGGCCTCCCTATCGTCGTGTGCTGCCTACTCGTCCTCTTCCCGGTCCGCGTGAGTCTGGTTCTTCACTCCGCAAACGCTTGCGCTCTGTGCGCACGTTGATTAACCTGTCCGTGTACTGTGACGGTACCGCCGAATTATTCCATTTGCCCGAGCCTGTGCTCACTCGCGAACAGATCATCGAGAAGCTAATGGCCGAGGCTATGAGCGAGTCCATTCTCGTTGGCGGCACCTCAGACTGCTGGAAGGTCCTCATCTATGGTGACACTGACGAAATGCTGCCTCTCTCCGAGGTGCAGCGTCGTTGGGAGCTCGGCCACACGCAATCTGCCACTTATTACGAAGGCAAACTGGAAGAGTTCCGCCGACTCACTTTGGTCAAATATTCTTGGCAGCGAACCAACTTGCATTTGGAGAAGGCTATCGTCGCATCCGAGACTTTGCTGCCGCGTCTCCTGAAATTCGGGTTCCGCCCCATCACGGACCTATTTAGCGAGACGTGGTACACTGCCGATGACGGCACCATGTCAGCTATCCGCGAGAAAATCGATGCCCAAGCTGAGATCATGGTTGGCGACTCTAGTACCGTCGAGACGGCTGTCAATGAGGCCAACGCCACTCCCGGTTTGACGCTTGACGCCCTGCTCGCAGCGCTCCCCACAACGTGTGCGGCCACCCTGCATTATCTGGCCACCCACCTCGTTGATTTGGTCCCTGAGAGCATTGCTGCACCCGCCGCTAGCATTGTGCAATGGTTACTGAGTTCCTGGACGTCCATATTCGATCATCTTCACTCCCTGTTGGTCGATCTTGCCGCCCATAGCGCGTTCGTCGCTGGAGCTCTGCAAGCTTTGGCCGACCTTGGTCACGCCTCCTGGAGTTTCGGTGAACTCGTTGCCAACGTCCTTTCTGGCACGGCGACTGCGGTCTACTTCGCCCCTACAGTGCTGCGTCTCCTCGCCTTTTTCTTCCAACGCTTCGATGAGGTGCACGGCGCTGAGTTCGTTTACACCCACACAAAGCTCTGCGCCGCTGCGTGCGTTGACAAGCTGCCCGAGCGCTTGGTGGCGATTGATACTAGCGGCATACCCGAACCTGCGGCTCCAGTGGATTTGGGTCTGCCCGAGCATTACCACGTCTTGCTGGAGAGCGTTGAAGCGTTTGTTTCCGGACCATCTGGGCCGAGCAACTCAGTCGCTATCGCAGCCAAAGTAATGACGGATAAGGTCATCAATCACATTGACCGCCTGCCCAAGCTTGGCGTCCCAGTGGGCCTCGATTCCGACACACGTAGCGCGCTGCAACTGGCCTTCCCTAATCATCGCGTCGTCCCGACGAGCGTTGTCGACGAGCACGCCGTGCAACAAGCCATCCGGGTTGCATTCCGCCGAATAGCTACTGAGGCCGTGGTCCGGCGTGCCCGCCCTGTACTCGCTGTGGGAGCTTCGTTGACTGAGATTCACAGCGCTGTTCCGTCGCTGCCGCAACTTGTGCACAACTGTTCACCGGAGCTCTCTGGTCGTGATGCTTACCGCAGTCGTTACGCACATCCTTCCCTCGCCGCCACTTCTCTGCACTGTCGAAATCACAAGTTCGAGGATTGCAACGTCGACCCCCGCATTGCCCCCGACGTCTGGTCTTTCTACTCGGCGCATGACATACCGATCGACCACTTTGTGCGTGCAATGCTGCGCGTCGGTGCCACAACTGCCTTCGTCGCGCTCAATTTGCCCATCCCCATGCTAGATGAACGCGTGACAGAGTACACCGACTCATTGGCACAGCTGCACTACGAGCGTGTTGGTGACAAACTCCACGTCTATCACATGACTGCTGCGAGCGCCGGTTACACGCATGATTTCGACTCTGTCATGTCCTGGGTGCGACCTACACCGCTTATCCCAGGTTTACACCTGCAGACTGAGAGCATGGCGCAATTCGGAACCTCGGTGTTGCTGCGGCTGACCCTTGCCCCCGGTGCCCAAGAGACCACCCCGAAACAATGGCACGCTGCTGCGGACGATTTCTACATCCTCCCATCGTTGCTCGGCCCCGATCTCACCGACCCCGGTACTCGCCACATCGTCATCCCGACCTCCCGTTTTGAACAGTTGACGGCTTTCGTCGCATCCATTCCTACGTCACAGCGCTCCTATGTCGACGTTGCCGCGAAATTACGTGGCCAGATGGCCGAAGTGCGCGTGGGCAAATTCCGCATCGAACCGAGGTGGCGTGTGTCCGTGGCTGATTTCCACTCCGTTGTGCGGCACGCCATCCTTGCCGTGCATCTCTACGAGTCGTCAGTTGAGCAGACTGCTGATCGTCTCCGTGCATACTTCGGGAGGCAGCGTCGTCGACATGGCACCTTCTTGCCGCGCCTTTTGCAGCACGCCTTTGATTTGGCCACGTTCCGACTGAATGGTGAGCGCGCCCCGATCGGGCAGCGCGCCGATGACTGGCTGTTCGATTTGGCTTTCTTGCGCCGCTTGGACAATACGCGCACGTACAATCCGTACCAGCGCGCCGGCACGTACCGTTTGCGCAACTCCATCGTCGAGGGTCCCAGGCTCACATCCATCGCCGATTTTGTGCCCCCTCTGCCGAGTGCCAGCCCTGACATCGTCCCCCCCCCGCCGAATGCGCCGTCTCAAGCCCCGCGCGAGCCTGTCGACCCTGCTGCAGTACCGTTGCCGTCGCCCGCCGGCACGGACTATGAAGGATCTGTTCACGTCGCCACGCCTGAGCCCGCTCGCGCCGACGATGATCGTATCTCGACTGACGATGACGACGCATCAAGCAACAGTACGCACGGCCTCTTCTCCGGTTCTGACGTTGACCCTATGGCCGGCCCCGCGCCATCATCTGCTGCCACGTCGGTGTCCCCTGATGATTCGGTTTCCGCCACCGGTGACAACGTCGACCCGCATGAGAACGTCCCTCCAGCCGGCGCCGACCCGCGCGATGATCCCCGCTTTCGTGCTCACTCGCCGGAATTTCCTGATCGCCCGCATTGCTCCGTCAAATCAGACTGTTCATTGCTCGGTCTGAGCGGTGAGCATTATTGCACCTCCTGCCGACAGCGTGTCGTGTATTCCCACGGGGTCGAGTGTCGTGTTTGCGCGCACATCGCTCACCCACCATCACCTGAGCCCTCGCCCGCACCTTCCGCTGAGCCTTCTATCGCCGCTGAATCTGAGACTGACGTCGACACCGCCACGAATGTGCACTCACGCGCGTCCGACGACTCGTCTGCAACTCGTGCTGACTCCATCGAAGTCGAAGACGACCCGACGCGCGACCCGGCACTGCCCCACGACGCCAGCGGTGAAATTTACCCGGGTACGCAGATCCTCGCCAACAAGTACCGCAGCACTCCCTCCGAACGCCCAATGGACTTCGGCTCTGCGTTCACCGACGCCGCTTACCACCCCGACGACATCGAGTTCAAGAGACTAACGGGCATCCTGTCTGGGTGCGGCTTCGCTTGGCCCCCGGATGTCCGTGAGCTCCCTTGTCCGACCAAAGGCGCTGTGCACATCCTGCGGTGGTTCCACCAAGCCAAGCGCTTGCGCAAGGCTGTGCTGCGACCCAACATACGGCTTGCCGAGTCTGCCTTCGACTTTTCCAACCCTGGCGACGTTGCCCTTTACTTGACCGAGGGCGACTTCAGCATTGCCCCCATCGTTGCAAAAGTCCGTCGCTTCGCTGAGTCTGTCGTCAGTCGTATACGCGATGAGTTGGAGGTCCCTGTGCTCCTTGTGGACGGCCCGCCCATGTCTGCCAAGTCTTCGCTGGTCCGCGCGCTGATCAACAGTGGTTGCCCCGCCACCGGTTACGTCCCTTCGCGACGCTTGCGCGACGATTGGATTGAGCATGTGCCGAATCGGCGCCTTCTGCGCATCCACACACGTCACGGTGTTTTCTCTAACAACGGTTTCTTTTCACTTGGTTTCATAGATGAGTATGGTGCCTTTGATGCCGTTGAGTTGGAAATGCACATGCGAGCGCTAGTCGCGGCCGGTTGCACGCGCATCGTCCTTCTAGGTGATCGTTTCCAGTCGTGCGGATCTCCACTTCCTGTTGATAGCCCGCTCGTCAATTACCACCTGCAATTGCACACTTCGTTGGGCATGCCTGCGGACGCACATGGACTCTTCGTCTACCTCAACAATGTTGATCCGGCTGTGTATACAACCACTGGCGACTCCCGACCATCCGTGTACTTCATCCCGGACCTACCTGACGAGCATCAGCGGCTTACTGATTACCGCATGCGCCTTCATCAGTTCACATTGCCCGATGGCTTGCCATCGATCGGTCAATCGCAGGGTTCCCGCGCCGAGGTAGCCGTTCTCCATTTGCGCGGCTCCCAGCTTGGCAATTCCTGGCTGCTCGACCGGCACAAGGAACGGGCGAACATCGCATTCACCAGGCATAAGCGAGCGCTGTTCCTCACGGGTGCTGATAACGCGCAGTTGGCCCTCCCCGGTATCATGTTCGAGCCCTACATCGAAGTGCGCGGCCGCCGCGCTGAGGCGGAGTCTAACACGCGTCTGCCGGGTTCGACGTTCTCGCTCGTTTACGGGGCCGGGTACGGTGCCGCCAAGGATCACCTAATTCGTCTCGGCCAACACGCCGGTGTTAACTTCGTCCCTGAGTTCTTCAAACGTACCGATGCCATCAAGCCTCGCGTTGTCCCAGATCGACCAGTTGTCGCTCTACAGTCCGCTGCCGACGTCGCCGCCCTGATCACCTCACTTGGACAATCAGAACCGCTGGCCCCGGAGTTGCGTGACTGCCATTTGAGCGCGCCCCGTTACTCTGCGACCTTGCGCACGCCAGACGCCCCGCTGCAGCGAACCGCTTTGCGTAATGAATTCAGCGATGCCAAGAACATAGCTGCCATTCACACCGCTTCGTCTGATTGGGATGCTCTGTTTAATGCATTTGACCGGCAATTTTCAACATCCAAAAAGGTCCATTGTACGGACGCTGAGTTTGCTGAAGGGCGCGCTATGTATGAGCGGTTCCGCGAGTGCTTTTATCGACCATCTGGGACCGTGGACGTCGGCTTTGATGCGACGGCTACGTGGCTCGCTCGCCAGACAGCTGCCACTGTTCGGGCCCTACTTGATCAATCGCCACTTGGTTCTGACGGGTTCGGCTTCGGTCTAGAGGCCTCATTCAAAACGCAGACCAAGGCAAAAGCCCAGCCCGGGTTTGCAGCCACGTTACCCTACGGCCAGTCAATCTTTGCGAACACTAAGGCCTTCAACGCTCGATTCGCCAATGCGCAGTACTTAGCTTACACCAACATGCAGCGACTCCTGCGCGACGACGTGATCTTAGACATTGGTTACAGCGATGATGATCTCTCTGCCCGTTTGCGCGAGCTAGGGATTGCCTGGCGGCTCGCCGGACCATCCAACCTTCAGGTCGATGTGTCCAAACAAGACTCCTCGCATACGGCTGCGACCTTGTGGTGTTTTTGCCTCATCTTGCGTGATTGTGGGGCTGACCCTGACGACGTTGCTCTGTACTTCAAGCATTGTCAGAGAAAACGCGTGCGCGATATGAGGGGCGCTTACACTGGTGCTACGGCATTCAATTTGGATAGCGGTGATCCCTTCACTCTCATTCGCAATGACGTCCACGAGCTCACGATGATTGCTTGTCGTTTCCTGCATTGTCCGACTGCTCACATCGTTGAGAAGGGTGATGACGTCCACGGGTGGATCCTCAACACAGAACCGCACCCTCTCGCTCGTCTGCCCTCCGTCATGGCCACCACTTTGAAGGTCGATGTTGGCAACCCGGCGTACCATGCTGGCCGGTTCCATAACGGTGAGCGCTACATTGTGGACCCGATACGTGCGTTCCTCAAACACTTCACTCGACTGCGCGACCCATCCGTCTCCGTCGTTGAACTTTGGCGCTCTTATCATTCGCGGGCCACTGATTACTCGCCGGCTGAGATCGACTTTTTGACTACAGCCTGCCAGAGTATGTACCCGTTCGTCGAGGGTCAATACGTCCAGTGCATTATCGAATTCATGGTTCAGTTGCGCGACTTCCGTACTTTCCGCAAGTTCGATATTAACGATCCACTGCAATCGATGCGCCTTGTCGACACTGAGGACTGCGCCGTTCAAGTCGCGAAGCACGTGCTACCTGGTCGCCCGCCTTCATTCTATCGCCGCTTCCGTCATCGCACTCGCGCCGACCTCGTTCAGCTCTTTAGCGATCATTCTGTCCCTACGCTTTGTATTGAAGACGGTGATGTACCTCGCCCCATTTCCGGCGTCGTGCAGTTATCACCGCACCACGCCGTTCTTTATTTATGAGCGCTCGCTCACCACTGTCGTTACAGTCATAATGACCTTTAATTTATCCATGCCCGTCTTGCTGATTGGCCTTGATCTTTTGACTCTTCCACCTGCTCTTAACTTGTTCTTTGTTTCACTTTCTATCACTAGTTTGCTTTTTGGCTGCGGCTACATCATCAGATGTCTCAACCCACCCCACCTGCCTCCCCCGGTGTTGCAGCAATTTCAACAACCAATGCACTCGTACCAGGCGGCACCAACCAGGCTGTCACCTCATCCTCTGTCCCAAGCGGGCAGCCCGACGCGCATTCACTCCCCGTCTACTGCGCAGCCTGGTGCTTTGCACTCGCCGGCGCCAACTCCTGTGCTTCCTACAACATACGAGACCACAAGGACGTCCAGTGGGCCCTCACCCACTTCGCCTCAGTCCGACTTACCTCCCTCACTGTCACAGTACATCAGCTCCGCTCCTGGGGAGCCGCTGCGTCTGGAAGTGGAACCGTCAATGTTCCTTTCCGTTTTGGAATCGCTCCCCGCAATCTATCCCTCGGGTCCCCCTCCGGATCTGACGATAGTGCCGCGACCCGCATCTTCGCGATCCCACACCTCCGCGGCTTCCATTTCAACTCCACTGTTGGCTCGTCCGTGAGTTTTACGTGGGGTGACGCACACCCCTTTCCCCCGGGTATTCAGCTCGATTTACGTGCCGGTGAGTTACGGTTCGAGTATCCGTCCATTATCTGCGGTCGCCACTCCGCAGGTACTGAAGTTCATTCGCTCATTGGTGTTTCTTGTGAATTCCGTATTGAATGTAGTGGCGCCTCCTTTGGTGCTTGATTGTATATTTGTTTTGTTTTGTCGCAATCATCCCCTCATACACGCAATGGGGCAGTTTTGATTATTTCTTGA

>SraLV3

CCTAAGCCCTTCCTTTCCTCTCCGTACCATCATCCCCCGACTAACGGTTAGTCGACGGGACCGTCCCGACGATGCTTTATCGCTCCGCCGGTCTTTGGGCCGCGCGTGACGTTCAGCGTCTGTTCGCCGAGCAGCAACCCTCCCCTGGCGTTGATGCTTGGGACTCGCTCACCGTGTGCTTCCTCCCGGCACGCGCTGTCGAGCCCTTGCGCCCCGTCACGCCGCGGGAGGCTGCAGCCGCCCTCTTCCCACCGGTCGCTTCTTCGCGCTCTCATCGCAAAAAGTATGCCGTGTGTTCTTGGCACCCTCGTGTCGAGACCATCGTCACACCTCCGCCCAGTGACGACGTGGAACCGACGGCGCGCAGCTCCTGGCGCTCACGCGTTCATCGCCCGCGCTCCCCACCCAAACTGAAAGGAAAGAAACCCACCCCTAAAACACCCTCAACTAGTACACCCGCGGAACATGGGCCGCAGTGTACTACCCCGGTCATCCCTCCGTCCCGACCGCTCTCCGAGCGCCCGCGTCTCCGCCGCCCCCGAATCTGCGAACCGCTGCGCGTTCGTCCTGAGTCCAACGGCTCCGTTCGCCGACGCCTGCGTGCATTCCGCAGTCAATTCGCCTTCCTCGACGTCACCGGCATCCGCGAGACATTCAACCTGCACTACGTGGAATCGATCGAACGTCTGCTCGCCGACGCAATCGGCACACCTCTCATCGACCAGCTGGTCGACGAGATGATTGCCGAGCGCTACTCTTCCGGCGAACCTATTAAGGTCGGTGGTGACGCAGACTGCTGGCGCGCGATGTTCCCGCATGCCCACGCTGCCTGGGGCGATTCGTTCGCAGAGCTTGCATCCCTCCAGATGTCGGTCCAGTTCGGCATGTATCCGCCGCACAACATGTTTTGCTTCTTCGACGACCGTTTCTCAAAGGACTGGCTCATCGAAAATTGGCTGACGGTTAACTGGATCAGGATGTCTCTCGACGGAGTCATCGCGCACATCTCTGAAGTCTGCGTATCCGAGACGCGCCCCGCCGGCGATCACTGGTACAACTTCGGCGAATTTATCGCGCTCCCGACTGACGCGACCGATAACGAGTTCACGCGCCGCGTGCTGACCATCCATGCATCCGAACGAGTACTCCGCAACGACCCTGCCTTGTACGCGCGTCTGGTCGGCGCACTGCCCCCTCGCCATGTCACGTCCGTCGAGCCCGTCGCCACGTATGCAGCCGCAGAACCCACCGTTGCAGCGCATGCAGCTGCCAGTGTCGCGCGCGAGATCGTCGCGAACTACTGGCAACACGCGCTAGACAAATTCAACGGCGGCCTGCCTCTCGGTGATAATGTGCCCGTCCACACTCTTGACGAACGGCCTGGGAACGGTCCCAAACCTCCGCCTGGACCTCTGCCTGAGCCAGAGGTGCCCGGCCGTACACCGGACAACGACGAGTGGATGCCATCCTTCCCGGGCGAAGATTCGCCGTACGACGTCGTGCCAACGCAGCCCGGGCCGTTCATTCCCCGTGAGCCGCTGCCGGATTTGCCGCCGCCTGACCACACGCCGTTCCCGGTGCCGCACCCGCCCGACCCACCCGTTCCAACGCCGTATCCCCCGGGTCCTTTGCCAGCGCCTCAGCCGGATTTGCCTACGCCTGCCCCGCACCCTGTGCCACCTGATGGTCACGGGCCGCCAATCCCTATCGACGTCGGCCAGCCGCAGTTCGAATTCCCACCGCTGGACATCCACGCACCGACTTGGGTCACACACTACATGCAGGTGTTGCACGACGCCGGCATGACGCTTGCGGGTGCAGCCAGCGAGGTCGCCTCCGGCGCCGCACACGTAGCCCCGTTCGTCATGCCTGTTTTGCGCTTGTTGCAGTATTACATGCAAGATTACAATCAAGCGCACAACAACCACCGCATGTACCGCGGCTCGGATCTCGGCGTCGCTGCTGCACGTTATGCAGATTCCCTCGGCGACATGGAGTTACACGCAGCCATTTGCGCGGTCGCCGAACCTGGCCGTGACATCATCGTCGGCACCGACCCGTACTACCTTGCTTTGTCACACGAGCAGGGCATGTTCGGCGAGGGGGACAACGGCTCGCGTGCGCTCGCAACTCGCGTCATGACGGAACTGAACGCCGCCGCCACCGCCCGCGGGAACTTGCCCCTCCTGAAGGTGCACCAGAACACGCCGCGCCAAGCTCATCTCGCCCTTTCGCGCGCGTGGCCTGAATTCCGGTGTCTGACTGTGAATTCTACGCATCCGCACGGTGCCGCGCAGTCCAACCGGACGGCCATGTTGGGGTTCACGTCTGCACTGATCAACCGGCGCTTCCCCGGCGAGCGCGTTCGCGGCATCGGGTTGTCACCGCGCCAGTGTGCTGCGCTCCCGAACCTCGTGCACAACTGCGCGCCCGTCCTCACCGGCCGAGACGAGTACAGACACAATCACGCGTACGACCGTGCTTGCACCAACGCGTTCCGTGTCGTCAGTCGTGACCACAAGTTTGAGGACTGTCACATGAGCTGTGCGCCCGTCGCCATCGCGATTCACTCTACCGGTGACATCCCGCTCGTGCATTTCGCCGAGGAGTGCATCAAATCAGGCGTCCACACGGCGTTCGTGGTCATGAACCTGCCAGTCGTCATGCTTGACAAGCGGATCACGCGGTATACAGACACGGACAACGGGTTCGTCTATCAACGCGATGGCGACCAGCTTCAGATGTTCTTCCGCGGCGCTGCTAGTCCAGGTTACGTGAACAGCTTCGGCGCGACGATCTCCTGGGCGCGACCGATGCCGCTCATCCCAGGCTACCACGTGCAACTCGAGGAACTTAAGCGCTTTGGCACCACCTTCTTGTTCGAACTACGTTTGTCACGCGGCGCACAAGAGACTCTTCCGACGATGTTCAGCACCGAGGAGGATGACTTCATGGTTCTCCCAATGCTCCGACCAGGCTGGCGCCAAGAGGACGGGCATCGTCGCGACTTTGCGGTGCCTGCACGCCGTTTCCGCGCTCTCGTGTCGTTTGTGTCCACGATTGAACCTGGCAAGATCGTCTTCCAACCCGTCGCCAACAAGTTGCGCGGTCAGCTCGGCGAAGTGCGTATCGGCAAAACCGTCGTCGAGAACCGCTGGGACTTGGAAGCGGACGAGTTCTATTCAGTCGTCGGTCACGCCATCACGGCGAACCAGCTCTTCGCGCGCGACTACGCCGAGGTGATGCCGGCGCTTTCGGACAACATCACGCGTGCTTATCAGCGTCACTCTCGCAACATTCTCGTGCGTGGTCAGCGGTACTTGCGCGACCTGTTCACGTTCCAGCTCGCCAAAAAGGCGGAGATCGACGCCACTGACGGCTGGGATTGGCTCCTCGACGCCCTCACTGCAACCAAGCTCGATCACGGCCAGCTGTCTAATCCGTACGTGTCGCGCAAGCATTATCAGATCATCAACGATGAGCTGATACACGACGATCCTTCTGTGCTCGGAGGCTTGGCCAACACCGCTCGTGAGGCCGTCGAGTTTGGGCAATACGTGTTTGCGGCTAACAATTCGATGTTCGAGACCATGGCCAAGATTTTGCGTCCGTACGATCCCGACGTCTGGCTGCAAAAGCAACTGGCTCCGCGCACTCCGACCTGGCGCGACATAGAAAATGCCAACACTGAGGAGCTGGAACGTTTCGTGGGCATTCTCGACCCGCACCACGACGTTGATACGCCTCCGCCAACGGAAATCTCGCACGAGCCGTCGACGATCGCACCCAACGACTCCGTCTCCGTTCGCGGCATCGAAGCCGTCGTCCCTGCGCAACCGGCAGCCCCTGTCACGCCGGCACCTGAGCCCGCGCCGACGCCGCCGCCGGCCAGTACGATCACGAGCCATCACGTCGCGCACGTCACTCACGCCAGCGCAGACGATGGTGGACCAGTGGTCGAGCAGCCGCCACCCGACGTGGCCGTCGTGGCCGCCCCGAGTCCGGAACCCATCGGCGTCACTTCGCCCGTTGAGTTTGGCAGCGACCCCGACGTGGACCCGCCGGCGTCCGTGCCTTCCTCCGTCAGCACGCAGGCTTCCACAGTCAGTTTCGAAGCGGAAGTCACACGCGATCGCGGACACGTCGGCAATGCACCGCTCATCGTCGACGAAAACGGCCACACGCTCACCGACGCCTTTGAGCTTGACGTCGACATGGACGCGCTCGCCGAGTGCCAGTCCCAAGCGCCGTTCGAGCCGCAGTACTCGTTGTACCCGCAACCGGTGCTGTCTGAGGCTTCGGCCGCCTTCGTTCGCGCCGCGCCCGACTCCGACCGCCTTGATGAGCTCATACGCCTTGGTCGCTTCGATCTCCCCGCGCCTGACCGTGGTATGAAAGAGCTCGTGAACACCATCGTCACGCGTGTGGCCGTACAGCGCTTCCACCACCAACCGCTGGATGACGCGGCCATAGCGCTCGAGGCTGAGCACTTCTGGACGGGCGACGACGTCAACCAACTGCAGGCCAAGATCATGTCGTTCTACAAGGACCCGCGCACTCGCGAGCCGTATTGTCGACTACCGCAGTTGTGTATCGATGGCATTGCCGGCTCGGCCAAAAGTTCGGTCGTGTGCGAATGGCTGCGCGAGCACGGTGAGTTCAGCTTAGTTGTTGTGCCTGGCCGGAATCTGCGCAAACAATGGGCCGACAAACTCGCACCGTGTCGTACAGCCCTCGTGGTCACCCAGCATGAGCTGCCAAAGAGCCCAAGCTTCAAGTACCTAGTGATCGACGAGGCGTTCACGTTCGGAGCTCACCACCTTCGCGCCTGGCTTGCGTTCGCTCAATCCGTCAAGGCGCGCGTCGTTCTACTTGGCGATCGTTTCCAGCGGCATCCGACTGCGCAGTTGTACCCCGTCGATCACGAGGTGTACACTGCTCGCGTGCTCCGACTCACGGTCTCGAACACGATTCCCCTGGATGCTATGCTCGTCTTCCGCCGCCAGAACAACTTGGGCGGCGTCTACGCGTCGCGCAGCGAGGTTTTCCGGTCGATCCGCTTCTACGACAGTCGCTTCAGTGACCGCATTGATCACTACGTAGAAGGTCTCAGGCTGAGCGCGACACGCAGCTTCATGGCTGGCCTGAACGGTGCGATCTCCATCGGGCAATCACAAGGGCTTCGCAGTGCCGACGTGACTATCGGACTCCCGCCGGAAGGTGGCGCCGCTGACTGGCTTCGGCAGCACAATGGTGCCCGCAACGTCGCTTTCACGCGCCACAGCGTGTGTCTGAACGTGCTCGTCCACCCTTCCAAAATGCGTGATCTCGTGTTCGGCATCGACGTCCGCCCCTGCACCTACGTGAACGGTATACGACTCAACCGTCTGCTGGGCCAACGCCAAGTGTACCCGTTTGATCTTGATTTGTTGCCTACGTATCGTGAACTTGGCGATCAACACGTCGAGATCAACGCACCGGGCTTGCCGCTCGACGCCAGTCGACGCCATGGCGAACACTTGACGAGTTTCAATTCGACATACGAGCCGCCCGTTCACGGCGACCCCGAGTATATGCTCGAGCCTGGCATGCATGAGATTCAGGGATTGGTCTACGAGCGGACCAACGTCGACAGCGTCAAGGATCATGAACATGCCGTCGGCTTCAACACGGGCCACTCACCAGGGTTGCACAGTGTCGGTGAAGTTTCTGCTCTTGTCACGCGTAACGACATGCGCAACATTTTCGACGACGCGCACAAGTTCGCCGACGTTCAAGTCTCGAACTCTACGTTCGAGGATCTGCGTAATATTCTTGAACGGCAATTGCAGAAAAACAAGTCGCCGCACACGTCTGGCGGCACCTGGCGCGACGCCGCTGCAATCTTCAAACGGTGGTGCGAGTGTTTCCTGTCCAGCGAGTTCGTAACTCACGAGGGGATCGATGATTTCGCCGTGCGCTGGCTCTCGACGCGCACGCCTGCCTTCCTGCAGCGCCTCTTCGACTCGCCTTTCAGTGAGAACGGGGCCTCCGTGCGGTTCCACGCCTTCCTGAAGCAGCAGACGAAATTTAAGGCCACGCCTGGCTTTGCCGCGAGCGTCAATTACGGTCAGGAAATCATCGCCAATGAAGCACCGTACTCTGCGGTTCTCGCACCGGGCGCGCTCATGGTTTACCGCCGTGTCAGCGAATGTATGCGCAAGGACTTCATTGCGGACATCGGCATGAGCGATGAAGAGCTGGCTGAGAAAATGCGGCGTCAAGGGCTTCTCGACGACCTGTACCAGTGCAATATGCAGATCGACGTCGAAAAGCAAGACAGCAGTCACAGTGCGGAACACGTCCTTGGCTTCTGCATGCTGATTGAATTCTTGGGCTTCCCGCCTGAATTGGCCGAGCTTTACTTCGAACATTGCAGTCAGTACGCGGTGGTCTCGCAGCATGAGCACCTGTTCAAGTGCACGATATCCTTCAACCTTGCGTCGGGCGATCCGTTCACGCTGATCAGAAACTTCTTCCAGATGACGTCCACGCTCGCCTGCCGCTACAGCGACACCGACGTTGCGCGCGGAGTCCAGAAGGGCGACGATCTGATCGTTGATCGGCACCTACGCAAGCGCCACGTCCTCTCGCTGTTGCCCTCGGTCGCAGCCGTGGTGTTCAAGGAAGATTTCGACATGCCGCCGTACCACGCTGGTCGTTTCGTGCACCCGCTCGGACTTTTCGTCGACCCGGTCCGCGCGGTCGCCAAGCATCTCACGCGTGTCACCAACGAAAACGTGCCGACCGCAGAGTTGCACCGCTCGTACGTTTCGCGTGCAACTGTTTGGCCCGCTGAAATTCAGGAATATTTGCGCCACGTTCTCCCTGTGCACTATCCTGACTTCACCGGCGAACAGGTCGAGCTTGTCTTGCGTATCGCGCATTTTCTGCGAGACTGGCGTTTTTTCCGCGCGACCCACGACTTTGTACCCGTCGTGAAGCTTACTGTCGAGTCTGAAACTGACTGCGCAGTGACCGTGGCGCGCGCTGTGTTCCCGGGGCGCGACCGGCAATTCTACCGCCGTTTCCGAGGTCGCGATGCTGCGTCTCTGGTTGCATTGTTTGCTGAGCACGGCGTGTCCGCTCGTCTAGTTCCTTCCCTGTCGCTGGCCGACCCCACACTTACAGGCGTTCTGGTATCGCCTACACACTGTCTCTACCGCCGCTCTTTAACGGCACAACGCTCACGTTTCTACGCGGGCGCTAGCTATGGCCTTTAAAGCTAGTGCCTTGCAATAACATGTCTATCCAGTTGTCAATCACCTTCCAAACCGGCTACCTCAACGCCAACGTCCTTTCTGTCGTTGGGGCTATCGCCGAGGATACGCGTGTTAAGAGCTATTGTTCCGATTTCGCTTCTGTTGTTATTTCTGAAATTCGTGTTACCGTGCTGGTGAACAATATTGATGACCGTTTCTTACGCGTTGGGCTCATACCATCCAATTACGCCCAACCCACCACCAAGGCCGCAGCTGGTTCCTTGCCGCAGCAAGATTCCTTCACGTCGAATGCGCAGACCCCCACTTCCCGCGCTTTCGTATATCGTCAGGCAACTGACGGTTCACCCTCCGTAGCGAACGGCACCCGGTTCGTTCACGGTTTGGAGTGGGATCTTAATGCTCGAGGCTCTCGTTTCGGCTACCCGCGGGTTTGCATCTGTCAATTAGGAGCGGATGCAAAGGATGACAGTAAAGTCGCCACTCTCATTGTGGAGGCTGATCTTGTCGTCTCCGGGCAATCCTTTGGCTCAAACTTGTGATTCTCACATACAAGTTTGTCGCCCCGCGTTTGAAGTTCATTCACGGTTTTTTAAATTTTGTTCATCGCTGTGTGAGGCGAAAAAAAA

> SrBenV1

CACGCTGCAAGTGATAAAGTTGTGACTTGGAAGACATACCACCCGAACTTCACAGCTCCTATAGCAACCCAAAGGGGTGGGGCCTCTTCCGATAGCCACTTGAAACCTTGCGGTATTTCCGCTACCCGGCGACATGGGTCTTTCTTCTCCACCGGAGTCTCCAGGGAAACCTGTCGAGCGTGCCGGTTGCTGGTCTGACAACCCAGCAGCTTATTTTTGCAAAACACACAAGTTGCGCGCGAATGTTGGAACGTTGTACACCGAGTTAGCACTTTGGAGGCTGCACCGCTTCCGGGCTCCCGGCTTCGCTGTCCGTACGGACGGCGAGGTCTGTGAGCCGGAGGAGTGGGTGCGGTCGATTCTGAAGGATCGGTTTTGTCCCAGGGCCGTGCAGGAGTGGATGCGCCTCCTTTCGGTCCTTCGTGGACGTCACTTCACCAAGACCAATGGTCCTCACACACATATTTGGACTAAGACGTCTTGGTCGAAGAAAGCAGATCCTAGTCAGGTCTTCCGCCCGACTATGCCGGGAAGGGTCCGTTGGTCTCAGTATGAGCACGTTGAGCCGTGGATGTTTGCACAGGACGACGAGTGGGACTGTTATGTCAGCCACGAGCCGTTTTGCATTAGGTGCAGACACACTCGTGGTTCTGCTGCTCGTGCGGAGGCTGAACGTGCTGAGGCGGTTCGAGACGCGGCTGGCGACGAAAGGCCTGACGGCCCGACGGTCGACAACGTCTTCGTCACCGCAGACCGACCCAACGAGTTTGGGGTCACCCCGGAGAATTGGCGGGAGACCGCTGAGGAGACGGAGGTTGGCACCATCCCTGAACGCGTTGATGCGGTTGCAGCCGTTCTTACAGAGACAGCCTTCAACAGAGCCATGGTGAGACGACGAGATGAGTTGATGTCTGAAATTTCCTTTTGCGATTTTGTCTCTGATTCTATTCATAATACTAAGGTTTCCTTTCTCTCTGCCATCTTTTCTTCGCTTGCTCTTCCTGTCGACTGTAACAAATTTTATGTATTTTCTTCTCGTACTGTTCGCATTCCTTTTGTTATTGATCCTTCATCCATTCCTTTGCCTGAGGACGATGTTGACTGGGACGTTGGTAAGGAGCCGTGGGAGTTTCCTCTTCCCTCCTGGGATCTGCCCGACGATTATGTCGGGGACGGGGTGAGGAGGAAAAGGCGCAAGCGTTCGTTGGCTGTTAGGAGAAAGAGATCGAGGCTCTACAAGAAGCGCAATTATCGTTGGACTAAGACGAAGAACCGTCCTTGCAAGCTTCGTCGTAAGGAGGCCAGGGCGGGGCTTAGGGAAGCTTGGAAGCTCAAAATCGCTGCGGACGAGTTTGCTCGTGCAGTCGACCAAGCGGCAGCAGAACTTTTCTCCGTGCCTGATCCTTCATTTTTGACCACTGATAGTTGCATTGAGGTGCCCGAACCAGAAGCCAAGTTCGAGTATGAGATGGTCGACACGCAGGTCTGGGGAAGTGGCGATTGTTGGACTAAGTTATCATCCATCTTGGTCGGCGATATGTTCGAACAAGTCATCGACATGTTCTACGATGGCGAGATTGACCTTATCCACGACGGTAACGATTTCGCTACTGCTCGACGTGTCCTGGAGAGCCTCAAGAAAGCGCGTGCCGATTATCCACCCACCTCTCCTGAGGCGGATTGGAATCACAGCGCTGTCTTTGTTAAATGGTCCACACAGGAAGACGGAGATTTGCATGTCGACGATGTCGAGTTCATTTACGATCGATCCGTCCAGATGATCGAGCTGATGGACGAGGACTTTTGGACCACCGACACACGCCGTGCGAGAGCCTACATGAAGACGCATGGTCGGCTTACAGAGTTCATGGACTTCCTTGAGGCCAATCTGGTCAAACGCAGGATTCTGCCGACAGTTGAAGGGTGCAAACCCATAGACGTGGAGGTTCAGGTTGGGTCTGACGCGGCTGGCATCGTCAACAGTGCTATGCAAGTCCTTGGCACGGATCTAGCCAAGCCTGCTGCCGCTCGTCAATTCGACGATGACCTCAAGTCGACTCCGACCGTGCCGTACCGAATGACCAGCTCACAATTGGCTCAATTCTCTGCTTATGTCGGGACGCCCGTGGTGTCATCTCAACGACTGTCATCTCTCAATGACCATCTTATGTTGCAGGCTGCGCGCTCTCTGCTCCGAGACGAGCTTCGCAAAATCTTTCCCCACAATGCTGATGGGGTGCCTGTGCTACATGTAGGCGCCACCATGCATGAGGTGCGCGCCTATTGGAAACATGAGGCGCACGATTTCGCCCTTGCAATGGTCGAGGATAAGGACATCTCGAGGACCCACCATGACGCCGCTGTCTGGCTTGCTCGCATAATGAAAGAAGCCAATTTACCATCGCTTAACTCTGCCAACACGAATGCCAAAAGCGTTTGTGTGGACTACCCTTCTTTGGAGGATGTTCTCAAAGCGGCAGAAAGTGGTGGTCGTCGGCGGTCTTTCGTCAAGTCTTATCCCAAGCGCAAGTATGGGGCTCTCGTGTTCGAGGATTGTCTTTACAAAATGTCGGAAGCCGAGTTTGGCTCGATTTGGGAGAAAACCGATGCGAGTGTCGGGTACGCGACTTTGTTCATTCCAGATCCTATCATAACGGAGCAGTGTGCCCCAAGCGACATCTACAACTATGAGCGGTACTTGCCTTCCATGCCCGAGATTGCCGAGTTCGTCAAGTATGCGTGGCCGTCGTGTCTTGCGTTCACTCCTCTGATTCCTTTCCAGGTCGTTGCCGATGCTGTCGAGAAGGTGTGGCGATGGGTCTTGGACACTGGTTGGGAGACGCTCGGTCATATTTGGGAGAGTTGGACCCAGTCCGGTTTCCCGATCGAGTCCATTCTCGGCACGTCCTTCGACGTCATGAAAATATCTCCGATCGTGCGGCAGATTCTCGACACTTACCTTCCAATCATCTACGACCGTTTTTGTCGAGTCAGTGTCACTTGGAAAGACGGCTTTTCGCATGGCTACGACGAGCAATTCAGACTTTGGAAAGCATGGACCAAGCCCAGGCATCGTTACAAGAATTTCTGTGTCGACATGGAGGTGCAGGCTCGGTTTGGTGAGATGTATCTTCTCCGCTTTACTCGCACCAGCGGCGCTTCTGACATCGTTACGTCACTAGCCCTGCCAAAGAACCGGCATGTGGTTCTCGTTGCGGATATCGAGAAATCCTACGACCGGAAAGTTGGATCTCTCGGGAGCATGAAGTACTTCCCTGTTCTGGCTTCGCACTGGTACAAAATTCTCAATTGGTGTTTGGATCAGCCGCCGGAGAGTTTGTCTTTTGCCGTGGTTATGACCACTCTAAATCGTATTCGTGGAGGCCTGTCTCTTGGATCTAGGAGATTGGTCGAGGAGATGAACATCGAGGACGGAGACGTGGCGAAGATTGCTCTCTCAGCTCTCATGGAGGCTTACAATAGACATGCTGTCATAGACATCATCGAAAATGATCCGGAGATGAAAGCTTCGTACGAAACTCACCTAGTCACTTTCCTCAAGAAAGCGGCGAGGTTTGGTCTCACGTTGGCTACTGGTGGTCTCGCAGTGCCTGCCTACTTCCTTTTCAGGTGGCTTTGGCAACAAGACAGACAGATCGAGTTTGTCAAGTATCCAGCTCCACCCAAGGTCATTCGAGAGGTCGGGAAACCTGTTGAGAATTTCGAGGAGCGTCCTATCCGCGTGATATTGCCGGTCACCGAGGAGAAAGTTGCCGTGAGTGGCTGTCAGTTCTGCGACATGCAGGCGGCGGGGTATTTCTCTGCGGATGGCAATGCCAACTCTGGTCAGGCTTTGCATGTTCGACAGCACAACTCTGAACCGTTCAACTTTAAGTTCTCTGAGTCTGAGATGGCTGAGATCATCAACGAGATACGCAATGCCGAGACCTTCCATGGCACATCAAAGGTCACTGCACACATTCGCAAGCTCAAACATTTTGCCGAGACACATATTGGTGGTGCGGACTATTCATTCTCGTTGGATTATATCATGGGCGGGCCTGGCACTGGCAAGTCTGTCATTCTGCGGAAGCTGGCGTACGATTTCGAAGCCCGGGGCAAGTCCGTCGTGATAGAGGTGCCGTTCAATGCACTTCAGCCAGACTACATCGCTTGTGATGTCATTGGAGCACCTGGCAAGCATACCTTCAAGGTCAACACCACTTGGTACACTCCTCAGCATGGAACCGCAGACGTCTATATTGTGGATGAGATCACGGCCAGCAATTGGAGGCATGTTTGTATGAAGGCGTATTTCCTGGGAGCAACACATGTCATCGTGGCTGGGGATCATTCTCAGACAGGTCTACGTCCTGAAGCAAACGAAGGCGCCAACATTTTCGACACGCTGGGCTCGAAGGCCGGCGACATCCGCACTCATGAGTTGGTCTACAATTACCGAATGGATGCTTGGAGAGTCCGGCTTCACAACATGCTTTATGGCTACAAGATGATTCCCAAGAGGAAAGACATGATCCCGCCGAAGATCATCACGCTGGCGGAGTACAGGACCCTGAATCTCGACAAGAACAAGCATAAAGAGATGGTCTACCGACATGACTCTGCCGAGAGAATCTTTGGTGTTGCATCAGCTCCCGGTTCCCAGGAGATCGCCAATCTTTCCATTCGATCGGCACAAGGCAGAACTTCTACGACGGCAGCAATTGCGTTGTTAGATGCTGACTCGTCGATGACCGGGGACAAGGCTCTTGTCAATGTTGGCAATTCTCGTGCCACCCACCAGACGTACTATGTGGTGGCCGAGTCTGTGGACGATCCTGTCGCCAAGGACATGGCTCAAAGGCTTCATATAGATACTCCTGAAAACATTGAGCGGGTCGCCAACCATCCTTTGCCTACTCTCAAGGAGACCTCTGTCCAGATTCTGAATAAGAGTCAAGGGGCTCTTGAACAGCATTTGCGAAATAAAAAGATCGAGGGAAGTGTCTGGGCTGACAATGAAAGGGTCTATCTCGAGCCGGGCGATGACACGCCTTTCGATAGTGGTCCTCAGCAGGCACAATGTTGGGTGTGGGATCAGTTTGAAACATGTCTCTTCGACCACATTCGCAAAGTCCGACCAGATTTAGAGCCTGAGATGCGTGCGTATCTTTTGGCTCTCTGGGATGACGACGATGCGATGGGTAGGACAGGCTTGGTCGGAACCATGCTCAATTATGTCTCGACCGGGTCTCGGCCGCTCACCAAAAGCGGAGAGAAGTGGCTTCTGCCTGTTGCGCGAACCTTAGCTTGGATGGACTCCAAAGATTTCCGTGTCGCTTACTTCGAGGACGGAGAACCTGTATTTGTCACTAGAGCCGGCCGCACAAATGGGCCTGAGTTCGTCTTGGCGAATGTGGACAACCACATCACTCCGATACGTGGCAAAATGGTTCGTGTCAAGTCCTACGGAATGGTCAAAGAGATGAACATGGTCGATGTCGTTCGTCCTGGTACTCTCGTCATGGACCTTCCGGTGGAGGGCAAGGTGTACGTTCTGAATCGCTACGCTATGAGCCCTGTGGAACATCCAATGGATGTCGCAGGTCCGGCACAAGTGGCCGCAGCTCTCAAGAACCGCGCCGACCCTGATGAGGTCGAGTGGAAAGCACTTTGTGCCGTCGAAAATTCGAAGCATGCCTTTGTTCCAAGGCCTCCTCCGACGCCACCTCTTTCTGATTCGGAAGACGTCAAGAAACCTTTTGTTAGGCCCAGGACCTTCGACGACAAGCTTGCAAGCATAACGGCTAAACCACGTTCTCGCGAGAACACTTGGATGGCTCCTATGGGACATGAAGAGTTTGTTCGAATACTCAACAAGTCCGGGGTTTCTCTCGCCGAATTCAGGAAAGAGATCAGTCGGTTGGACACTCCCGCCAAGAAAACTGTGTCTGAGGCTGAGGCCAAAGAAGCGAAGCGTGTTAAGGTTGAGCAGCTCATGGCGTTTGCGGAGACGAATCTTGCTCTAAACGACGTCACAAGACCTAGGATGCGAGCCTACATCGAGGGCACACTTGTGCCGGCAAAAGTTCCGACTGTCAAGTTTGCCGACGCAGGTGGCTTCGGGTGGAAACCGTTCGGCTTCAAGGTTCTTCGATCTGAACCGGTTGAGTTTGAGTCAGCGCTGCATGCAGACGAATCGACGGGCCGTGCTCCAATGGATGGCCTGTTCATGGCCGATCCTACTCCAGATCAGATAGAGGAACCAGTCGTCAAGACGAAGTTCCGGGCGGGCACTGATGCGTTCCGAATGGCTCGCTTCCTTGACCCTTCTCAGGCTTTCCCGGAAGATAGGCTTAATTTCGCGACACAGTATGCAGGTGTTGAAGAAACTCTCGACCTTGATGTCAATGTGGATGGTATTCTCTGGGATCGAACGCCAGCCGGTAAACTTAAGCGGCGCGCCAGGCGCACTTGGCGACAAATTTCGCCCGGTCTTGGGAATCACTACACTGATCAGGCTACTGAAAACATTGCTGCAACGAAGCGTTATTCCGATATCAAGAAGCCGGCGAAGCGGCGAACTGCTGAGTCGGATGCCTGGTGTAGGAAAGTCGTCAACGAGGCCTGGGAACAGCACTACCGAAAAGATTACCTTTTGGGACGCGAAGAGCGCAACTGGGTGGTCAACGGAGCACTTCGTGATGCTTGTCGGCGGAGGTATTGGAAACGCGGGTTGCAAGCGCGTGCCAAATGGGCTGGACACTCACTTACCTATTCCGGAAAGGCACAGTTCAAGCCTGAGAAGAATGGTAAGCTGAATCTCGAAAAGAGTGGTCAGGGTCTCATGCAATCTCCTGCGGATTTCAACAACGAGTGGATCTCATGGGCGCGGGTACACAATTTCATGCTAAAGGACGCCGCGAAGGACACTTGGTTGACAGACAATAGGGAGTCTGAGGGCACTTTCCGGACTAAGGTCACCGCAGGCATACAAGCGTTGCCGGGTAGACCCATGATTGCCATGGTCGACGCTACGACATTCGACGCACAACAGAGTCAGGTGACGGTGTTCATTGAGAGAGAGTCCTGGCATAAGTTTGGCACGCCATATTCGGAGATTGACGATTTCCAGGAAATAAGGAAGCCGAACAAGTTCATTGCCCACGGCCAGTTCAAAGGTAAACACACCGGGCAGAAGGGCTCGGGCTTCTTAGACACTCTGTCCGGAAACACGTTGCTGGAGTCGGTTCTTGTCGCTAAGATGTTCAACGGGAAAGGTCCCATGATCGAAGCTGGTAAAGGAGATGATTACATACGCGTTCAAAATGGCATAACGGTCAACGAGGCTCGGGTGAAGGAGGTCGAGGAGTATACGGGTATCAAATTTGTGACAGATGTCGGCTACGGGGGAGAGTTTATAGGTTGTACGGTTTCGGCTGAGGGGATGTTCCCTTCAGTGACAAGGACCGCAATGAAGGCGATGGCTGCAAGAATACGGGATGCTGACCACTTTTATGAGTACCAGAAGTCTTTGAGGGACAAGGTTCTCGAATGGCGTCGCACCGGGATAACGGAAACGATCGCTGTCTCTGCTGACGCGGAGAATAAGTCTCAGTCATATGTTGAGGCTTGTTTAGCTTTTGTCAATTCGATGTCGCACATGTCTTACGATCAGTACGTCTCAGTTTCGAAGAAGGTTAAAGAAGTTCCTTTCAATTTGCCCCCTGTTTCTCAGGTTAGGGCTGCCACATTCGTTTAAGTTTTCCTTATTGATGTGATTTACGCACGTTTGACTCGTGGGCACCGCCGATGACGGGACACATTCTATTTTCTTTCCTTAGCTTTCACGTGAGTTGTGTTAGTGTTAAAGGTCAAGGTGTAACTGTCACCTTGTAAAAAAAAAAA

> SrEV1

TATAATTAAAGCATCAACCGACCATGTCCAGCAACACCAATAACATGCAACCAAGCAACCACCACCAAAGTGAGGGACCACTAAAAACGCTCCCAAGCCGCGATAAACTTTATACAGCTATCAGGGATAACAACGGAGAGTCCCTGATAGCTAGTCCTTTCTCCGTAGAGGATAGTGTAGCTAACACTATCCTAGAAAAAATTCCCCAACTAAGTCAATATGACTTGTCAGTTGACTGTGTTGTTGATGACAACACAGACGAAATGCAAGCAATATGCGATGTTCTATTGAAGCGCATGTATGATAATGTTGATATGGATGGGGATTTAATTATTTCTGTAACTGCCAAGTTACAGAAAAATGTTGCTAAGGCATTCTCACCAGATAGTGTTAAGTTGGGTGGAGACACAGATTGTTGGAAGAAGATTTTGTGTCCAAAGAAGGCTGATCTCGGCTATGGGTTTTGGATGGCAAATGCTGCTAGGGCTCGAGAACATCGTAACAATGAGATCCAAAATGAGCAACCTATCACTTTGACGGAATTGTCAGAGATGATGTGGGATGCCAATAGATTGTACCCACTCATACGAGTTAGTAAAGCTAAATACACTGAAAAAATGACCCAAAGAATTAAAAGTTGGTATGTCAAATTTGTTCTATGGAATGAGAGCGATTTGCATCTAGAAGACTGTTCTTACGAAGAATGTCCTGATGACAGTTACATTAATCTTTGGGATGTGGATCAAGAAATTAAAAATGGAACATTTATGAATCGGTTCTTCAAACCAGATTCAGATTCTGCCAAGACGATTAATTTTGAGCTATTGAAATTGGGATCTGACGACATTTTAGTGGGTGGTGTCACACTAATCAATGCTTTGTCTAATGCTCTTGGTATAGATGCTAAAACAATTGCTAACGATCCTGAAATTCAAAAGAAAATTTCGACGTATGCTGAATGGGCTGCTAGTTTGAGTATTAATAAGAAAAATCGTGCTATATTATTACCTAGAGACACTGATGTCGCCATTATAAACACACTAAATAGCAATTTCGGGTCAATGGCATTTTTACCTTCAGATTTGGATGCACATCCACACCCCTATTTGTCAGCTTGTAGGAAAGCTATTACTCATAAAATCGTAAATGAGTTCCCTAGATCAAGAATCATTCATGACCTCGGTGGTGCTTTCGATTTTCACTTTAAAGAGGGTTATTATAATGTACATAGCTGCTTTTTGCGAAGTAATCCAATGGATGAAGGTAGGTTTATTGACAAAACCGTTAACACTCTTAAAAACATATCTAACAAAGATAAAAATAGGTTGCAACTAAGTGATAGTAACTTCCATGAATTAGTATATTCCAATCCAGAAAAATTCGTTTGTGGTTTGCCCGGTCAAGATTGCCATTATGTCACAACACATGCCGTTAGTTTCGGTATGTCTGTGGATACCTTGCTTCATGTACAAATTGAGGACTTGTTAACATTTTATGCAAAGAAAAAAATTATTAAAGCAACTCATGCTATTACCGTACCAAGTGATTACTTATATAAAACCAGTGGTCAAATGGCTTTCGGCGAAGCAAGTTGGAAAATTGAAAATGGCTATTACTGGTTTGTTACTAGTGGCGATAGTGGGGTTTATAAGGTGGAGGATAGCTTACTACGTTATTATTTGCAAACACCTATTGTCGTTGCTGAAAACTTTGTTGTTTATATGCGCAATAGGGGAATGCTTGGTAGCCATTATATTTTAGATCACTACATAATGGACAAGACTAAATTCATTACAGGTCCCTTTTACAATGCAATTTGGCTGTCGGGTCATACTGAAGATGTGCTAGTGGAAATACCAATCATCGATACTACTCGACCAGTCAGCTTGATGAAAACTCCTTACATCAAGAAGGATCAAATTAAAATTAACACTAAGTTTATGGAAAAGTTAATGATCCGAATGCTAGCTCCCACAAGTTGGGAATCTCTGCAAGTTTCTGCCTCAAGTTTGGTTAACACAACCTACATCACAAGTGATGGGTTCACTCGTTTGTTCAAAATGGATTGGAAAGAAGTGTACTACCATTGCGTCGTTGCTTGGTGGCAAAGCAACAAATTTGCTGAGAGTCTAATGCCACTTGTTAATGCTGCTAACACGCCCAATCTCACTGAAAATTGGTGGTCAGCTGCCTGGAACAGTATTAAAAACTTTTTGAAAGAGACGGTTGATGAACTTGATCCAACGCAGATTGAAGTATTATCAGAATGGTTGGGCAGTCACACGGAAGGTAAAATGAAAGTTGAGGATTATACTATGAGTATGCTCACAGCTGTTAAAAACATAGAAAGCAGTGTAGAGTTAGGCAGAAACGTGAATATATTGGAGGCTATAGTTGCTGATATAGACGTGTCTATTGAGGAAATACCAACAGCAGAGGATATTATCAAGACTGGAAAATTGGCAAACACTAATGTCTACAAGGCTTTAAAACCTGTTGAGATGGAGACCAAAAACTGTGGTGGCAAGTGCCCACACAACCATGAAGTGCCTCATCATCATGTTATGTCACAATATGATATGCCTGTTGATGCTTGTAGTTGTTGCAAAGTCAAATCATCCTTGATCAATAACTTGTGTATGCTATGTAATGAGAAAGATTTGTGTATGAATAAAAGCAAAAAATGCAAGCATCATCATACAACAATTAATGATGAGTGTTGTAACAACAAAATTTGTAAGTGTGTTAAACCTTACCGTTGTGCATGTTGTGAACTTTCGTCTCAGAACAAATTTTGTAGAGTGTGTATGGTGGATTGGGAACATATAAATGCTATGTTAGAGTTGAATAATGAAGAACAATTTCGTGCTGAAAGAACCTATGATATTATTCAGCACCCGGACATAACTTTAATTAAGGACAGTCCTACCGTTGAAAATAGGCAATTAGATAATGGAACAATTGTAGTGCCAGCCATCAATCCAATAATACAATTGGACGATGTTGACACATATTGGCATGCTCATTTATGTAGTAAATGTAATAATGCTTACGCAATATCACATATATTCAATGATGTCAATCATCCAGTAGTAGTTGGTGAATGTCCTTATTGTGGATCTGGAAGAGATGATAAAGGTAATGTGTCCATCAAAATGGAAGCATTTAATTATGATAGTTTGATTAATGTTTTCCAGACTTACCCTAGCTATGTTAAATCCGAGATTGAAATTCAGGAAGAGACAAGCAAAATTAATAAGGAACTGAGTGAAGCTGATGAAATTGAAATAGCGAAATATCCGGTCCAAGATGATGTCAAAGAAATGTTGGAGGAGAACGAGGCTGTGGCAGAGTCTGAAGACATTGAAAAATTACAAGAGGTTTCGGAGCAATTAGGTGTTGTCAAACTCGATGATCTAAGTGTGCGTAACGATTGTTTGTACTTTAACATGAATGAAAATGACCCTATTAATCCCATCAATCTTTATCATTTGTTCGTACTGCCCAACATTTATAAATTGAGTGTCACCGTAACAAACTATGAAGACAATGATGAGGTGGGTGAGTGTGGTTTAGCAGCTATGGACCAAGCTTTCCATTATTCAAAAGATGACATGCGAGAGGTGTGCAACAAGATGTTTGGTCATTCAGACTGGTTGTCTGCTATGGAATTAGGTGCATTAGCCATGCATTACAAACAAAACCTAATTGTTTCTGATGGTACAGATACTTTGCTAATTAAAGTTGACAATGAGAACTTTGCTGAATTAATTGTGTCTACCAATCAATATAATGGGCAGGAACATTGGGTACATGCCACTGGTAGAATAGAGTTAATAAATAATAATGAGTTAAGGAGAGCAATTCGGAACAACTTGATGATGCTATATTTAATATCACAAATAGAACAAAATAAGAAACCAACTATGGTGGAAATAGTCAAAAACTACAAACAGTTGGACAAATACTCAGTTTCTGAGGAAAACATTGGTATGAGGTTCACTGACAGTAACTGGATCAAAATTTATCAAAGATTTGGTGGTACATTTACTAAAGATGGTTTGGTGCATGAATCAAACACATTCAAGAATGACGATTTTAAGAAATATGTTACTAGACACAGAAACCTGATGGTTTTAGAGCATCCAACAGGCTTTGGTAAGACAACAAAAATTAACAACTTTGACATGCCAAATGAAAAGAAAATAATCATCTCACCCAATATCATTACAGTAAAAGGTTCAATTCATTACATACACCAAGTAACGGGTCAGCCTGTTGATGCTAGAGCTGAAAGTCAATGGTTCACAGCTGGTAATTTGTCAACTCATGAGTTAATGGAAAATGAGAAAACCAAAATTCATAACGCACACATTATTTTGATGACTGTTAATGCTGCATTTGAATATTTGAGTAGGGGTATACTTTACCCATTCGAAAATAGGTTGGTTTATTTGGATGAGGTCCATGATTTGACCCCTACTTACTTGGGAGTTGCAAGATTATTGGGCAACAATGTAAGAGCTGTCATGTCAGCCACTTTCGGTAATAATTGCTACACTGAGACTAGTTGTAAAGTGCAGACCAAAATATTGAACTATTTGGATGTCAACGATTATTTAGGTACAAAGGTTATGGCAATTTATGACACTATAGATGCTTGCAAAACTTTTGCACAAAACATTGATTCCACATATTTAGATCCCAAATCAGCCAATTCTGTTGTAACTTCTAAAACTAGGGACTCTTATGACCCTAATATTACTAAGTACGGTGCGGCTACTAATGCATTTACCACGGGCGCCACATTCCCCAATGTGAATTACATTGTTGATTCTGGTCGACGACTTACCATGGGCATCTACATTCAGAGTGAGTACGTGATCAATAGAAAAACCAAACATCGAGATTTGTTTGAGACAATCAAATATAATTTCACCTTTAGTGAAATGATGCAGACCAGGGGTAGAGTTGGGCGCGTTACAGAAGGCACTTATGTTTGCCCCATGCCTGTGTTTGACAGACCGTATGTGGAAGATAGTGCTTTCTATTATGCTATTTATGGCGGTACAAACATGCCAATTAAATATAATGAAGTCGTCGCTAATTGGTCCACACAAGAGGATTATCGAAACTTAGTCTCAATAGAGGACCCTGAAATGTTAGAAGTGGATTATGTGAAAGATTTCTTAAGTGAATTACAACAGATATTTACTGCTTTCAAGCGCGTGCCTCAAAGGTCAATTAACTTTGGTGACTTTGACGTAAATGAAGATAATTTGAAGGTCATTTTTGAACAGTACGTTACGACAAAAGCACAAAAGATATTTACTACTAGACCAACGGAAGCGTCCACTTCTCAAAGTAGAGCTGAACACTTAGGTGAATTACACAAATGGTCATATATCAGTAGTAGAAAGTGTGTCGTTTGTGGACGCGCCTGTCCAGACTTCTTAGAAAAATGTGGCTTGCACCAGTATAATTTACATGAATCAGATTTAAAATTCCTGTCTTTCCCTAAGTTGGTTGAAGGCAATGTCTCATACCCTGTACCACCCGTTAAGCTGATAGACACAAGCTCATCAAGCTATGATGTATCAATTCACGTAATAGATGAAATGCGATCCACTTTAATAAATAGCATCAATACAATGGCACCTGTTGCAAAATATGCTAGAATGAACAAAGTAAGCACATTCCACAATGCCTGCATAGTCGTTGAATTGGGTAAAAATAAAAAATTAAAAGAAGAATTGCCTGGCACTCGCAACATTAGAGCTGGTGAAATTGTTTTCCTGGTTTTACTAGAAACACATCGTGTGATGTTACAAATGATTAATGAAGATTTTACAGCTTCACAAAAAATGAAAGTCATTTTGAACATGCATAGTAATAACCACTTAATGGACACAATGGTAAATCTTATACGTAGAGACCCCGTTGACATGCTACAATTGTTTAGTAGTATGAGAAAGTTTGTCAACTACACTGGTGCTCCCGGTACTGGTAAGACTTATAATGCTGTTGCGGAAATGCGGAAATTGACAAAAGATTTTTGTATTATTACAAAACAGACTCTCAATTCAGTTAGGTTAAGAAATACCTACAAGTGTAATGTTTACACACCAAACAGCTATGTTAAACAAAGCCGAGTGTATGATGGTGTCATTATTGATGAGGTAGCTGGGTTTGATTTTGTTGATTTCTTCACCATCAATCTAAATAACAAGTATGTTATCTTAACTGGTGATAGGAATCAAATTTCCACCTCAACTAGATCATTACAATTTCATGCAGAGGTCAATGATTTAGTAGCTATAGATCCTGTTATTCCTAATAAAGAATTCCATGAGTCATATAGATTTGGCGAAAATACTGCTAAATATCTCAGAGTATTTAATCCCAAATTAATTGGAAAGAATGATAATGACTTAGGTATCACATTTATTGGTGGTGAAATTACAGATAACTCGTCCTTCCAACAGATGTTGAATAATGTTAAAGTTAGCATGATTTGCACTGCTACAAACGCTAGGGCTAATCTTTTAGCTCGTTATGGCAGGCAAATGGGCATTCCTATTAACACTACCAGTCGCTTGCAAGGCTACGAGGCTGACCATGTGGTCTATTATTTGGGTGGTATAAGTGCTAGCAAAATCACTGATTCAGAAGTTTATACTGGTCTTTCAAGACACAGAAAGAGCTGTACTGTTATAGTTGAGCCTGAATTGTTTAGTTATGTGTACGACAAATTTAAGATCAGTGTTCGTACATTGAAGCAACGTGCTGGTGCTATTGTGTTGCAAGATTTGGATGAAGTGACCAGGTTTCTATTAGAGGAAGATTTGTCTGGCGATTTAAAAAGTTTGATGGACAAGCTAAACGACAATTTGGCTGACATAGAGGGAAACAGATTTATGAAGTCACTGAAACTATATTTGGCTAATGTGTGTAGGGTTGCTAATGAAAATGTAGATAAAATTGTTAGTATTGTCTTGAAAACTTTGAACATGTATAACAATGAGAACAAAGAATATAAGGAACATTACATGAAAGCCATGCAAGAAATTAGAGATCTTAATATAGTTGGTAATAGTTTGTATGTCAAATTATTATACCTTAAAAGACGTTTTGTTAAACCAGATGAATTAAAAGGCTTTATACCAATAAAGAATATTAAAGAATGGGAGCATTATACAATACCATTTGATTTCATTAGAGATCATTTGATGATAGGTGCAAGTGCAGACGATATGAAAAGTCTATTACACAGTATGGACGTAATTGTTGATGAGAAATACGTGGAATATGTAAAACAAAGATCGATATTAAATAATGAGATCGGTGAAGAGTCTGTCATCTATAATTTGCTTGGTAAACTAAAGTCATTGTGTAAATATTTAACTAACAAAATGAACTTTAAATATGACAGTGTAACTTGGCAAGCGTTGCCACAAATTTCATCTGTGAAACGCATAGACGACATCAAGAAACTGGTTAATTCTGTTGTAAACGATAGCTACAATGGTGCTAAAGAGTTGAAAAACTTAATTTTGGCTAGAATAAACAATTATGTTGAAGATTTCTCCAACAAATATGTGATCAGGGAACACAGAAACTTATTTACAGATGAAGTCATCATAATCCCTAACTACATTGTGGAGAGAGATCTAGATTTGATTTTAGCTCGTTCACCATGTATTTATGAGTTCTTGCCTAACTTTAAGTACGATGGTCTGACAGACAATGTGAGTGTAATTGTTAATCATTATTACAAATGCTTGTTAGAAAAGTATCATAATATTCCTATTTACATAACCACGACTATTTATAGGCACAATAGTCAGTTGTATATCATTGGTGGTAATAGAATGGGTTCTATAAAAGTTGGTGACAGTGATAAGGCTTACGGGCGTTTCGTAAAATACTTGTATATGAATCACAAATATAACGTGTCGCTGTATAATTATTTAGATTTCTTGACTGAATTGAACTTCAATGAGTTTAATATTGGAAAGTTAGTTGCTAGCTGCATATTGTCAAATATCATGTATTACTCTATTAAATCAGTTGCTACTATATTTTTGGGTGTTCGTAAGGCGATATATGAAATTGCTAAGGTTTTGCTCAAGTTTGGATTGATCAATAGATTGCCTAAGTTAGATAAGTGGCAGTTTAAATATACTAAATTAGATGATGCCAATGACAAAACATCTTTGTTTGAATTGGATCCAGAAGAAATCAAAATGATTATAAATGACCATTTTAATACCAATGATCAAGAGAGCCCAGAGTGGTTCAAGACTTGTTATAGTTTGGTAAGGAAAGTTCTCTCAATGAAAGACAAAGTGGGTGATTATTTGTCACCTAAATTGAACATGTTATATGAAAAAGCTTTGAAATGGGACATTACGAGTAAGCTGTCAAGTTTCTTTAGGGCTATTTTAAAACATTGGGCAAAGGAACAAGACATATCATTTGATGATGATGTTGATGAGGGTAGTGCAATTGAAGAAGAAATTGTAGACAAGATGGTTGAAGAAGCTCGTGAAGAAGCTGAAGAAATTCAAAAGAATGAATTCAATCTGTCCATTAAGGTGGAAATGAATGAAAATGTTAAAACACCAGAACCATTTAAAAAATCAATTGACTGGTTAACATACCCTGAGTTTGAAAAACAAATGTTTGCACCTGAAGTTGACAAACGATTGATGGCAGTTGAGCTGATTGAGGATTATAGTTTAATTGGTTTTGAACATGTTATTGATAGAATTAGTTTCAAGAACTTGACAACAAAATCTAGTCTGTTTAAAGGTAGAAAAGGTGTTGACACGTTAGAGGACGGTTATGCTAGATTGAATATTAGTGTCTTAGGGGCAAATCATTCTATTGAATTTTATGAAGCGTTAGAAAACAAAATGATCATAGTGTTTGACAAAAATGCAATTATAGCTGTAACTGATGATGGTAACAAATCTAAGATTAAAATGTTGTCAGAGCCTACAAAGATTGCTATGGATCAGATAAAGGAGAATGCCAGCAAATTAAAATGGGTAGCAGGAAATAACAAGTTGCAAAAAGTCTTCAAGTTTATGAAACAATTATTGAAAGCAGTTGAAAAATGGCTGAAAGACCACGCTTTCTTGATGTTTATGCGCTCTTACACATCTAAAGATAAGCGCAGAATAGACGTTAGGATACCATTAACTAAGTCCGAAATTAGAAATGAATTGTATAACTCGGACAGCATGGCTCAGTTTGTTGACTTTAGATCCCGACTAAATGATTATAAGAAAGTATTTTACGAATCAAGGGGTGTTTTGTACATTGTATCTGGTGCTGATAATTGGAGCACAAATTGGTGTTGTGTGATAAGAGATGTTGAAGTAGGTGTCTCAGAAATTGTAATGTACTCCAAGCACAGAAGACAATATATGGTTGATTACTTGCAAATGTTGCCTGCTGGTTTGAGTGATATTATATTGAACACTGGTGGTGTCTCTTACAATCGGGACTTATTTGCCAATAGTTCTTATGATTTAGAATTTGAAGAATTGAGAGGGTTGGATCGACCTAACAAACATTGGCGTCCAAACTGGCGAGGTGGTTCCAAAATTGATCAATACTTTTATAAGTTAGGTTTGCGATCAAAGAATTTGTACACTGAATATGATAAAAGATTGATCAATGATGCACTAAATTATCATCCAGTGATTGGTTTGGAATCATTCCAAGAAGAACGACAATACACGCTACACAAGAGATCAGGTAATTTGAAACGTGATGGTGTTGTGGATCAAGACAGTAGTGGTAAGATGGTGAATGAACGATTAAGAGCTTTGAAAGAGAGTTATTATTTTTTGGCTCCTTCTGGTGCAGGTAAAACCATGACTATCAGCATTTTGAAGGAAGAGTTTCCAGATTATGACCTTATTGATTCAGACAGGGCTGCGGAAATTAATTATCTTACCAATCTTGAATCGTTGAGACTCTCTTATAAAGCAATGTTGACTAGATATAAAAAATCGAACAAAATTGTGGAGGATAAAATTGTCAAACCCACAATAATATTCGCTCACAGTGTGGCTGAAATTCCAGATGATAGCTTATTAAAAGGTATAGGGCATTATATTTTGTTTGATAATTGTAAGATTGCCAAAGAAAAACTAACGTCAAAAGTTGCATTTCAAGATATGCACAATTTGTGCATGTCGTCTGTACACCCTTTTATTGATGCAAAAGGGTCATATTTTACAGATGCAGCTAGAAAAGCAATTGTGTCTATTAGAACGAAAACTATTTTTCATGATGTTAGCAAAATAACACCCAAATTCCAAACGCCAGATGTGATTTCAACAACACTAGGTTTGGAAGACCCCACAGGCATGGAATTTTATACAAAAAATGACATGGTGGTTGACAGTTTAATTCCAAACACAAATGATGTCAGAGTGAAAAAGAAAGGTGGGTTCGAATTATTTAAAAAGAGAAAAATATACGTTACTGGTGAACTTGATGATGCAGCAAGATTAAGCTTGCCTGATGAAAAATGGTCCATTTTAAAGAGTTTAAATAACAGAATATTTAACTCGGTACCTTATAGATCTGAAAGAGTCACCAGTGATGATTACCTGCACTCATTGAGCAAGCTGTTTGTCCCAAATTATGAAAAGTATTTGGAAGTGTTTTCCAAAAACTTAATTGTTCCAAGTAAAGATACAATGATGAGTTGGCTGGAAGACAAAGGTCATAAAGCGAACATCATCATTGGATATGATTGTAATATGCAGAACCATATGGAATGGTTAAGGAATTCAGGACACATTCACTACAAGAGGGAGGTAATTTATAAACAGGCTATAAAAGACTTTCAAAATGAATTAGGACGTATAATTTTGTGGTATGATCATGTTCTAATTATGTGGCGTGCTCCAATGGTTAACGATGCTAAAGAAAGGTTAAAATTGTTGTTAGACCCAAACAAGATTTATTGGGCAGATGGTAGCTCACCTGCCGACCTAAATAAAAGTGTTAGATCAAGAAAGCATTCAAAATACTTGTTGGAATTAGATTTGTCAAAACAAGATAGGCAAACCGATGCTCCCATAATTGAAGCTGAGATGGATTTTCTGTGTGCTCTAGGTATGCACCCAGAAGTGATGGATTCTTTTAGAAATATATTGAAACCAAGTTTTGTGAAGACGGCGGACAACTTGTCATTCCATTTGCCTGTAATGCAATGGACAGGGGGTGAATTGACAGCATTTGGTAATACGTTTAGAAATTTACTATTCATTGCTGATTTGTGGGATGAGTGTGATTTCGACTATGCAATGGTATTAGGTGATGATAGTCTAATCTTCACAGACAAGTATCAAAGCGAATCTTATGTGGCTGGAGTTGCCAGAGCTCGACACAATGTTGACTGCACGTTTAGCATTAGAGAGGATACTGGTTTGTTCTTACAATATATAGTTGCTAAAAGGGAAGCAGATGAAGGTTACATATTTACCCCAAATGTCAAACGAATTTTAGGAAAATTGGGCACAAACTTTAACAAGTATAACAGTATTGAATGGATTGCAAAATGTCAATCAACTTTGATGATGTTAGGCTATACATCTTTATTAACTAAATCAGCTCTGCTTGTTGAATTACCATGTTATGTGCCAACTGGTTTCACAGAAAGTGATTTGCTGGCCAACAATATGAAGTATCATAAGGCCGAAATTAATGAAATCATTGGTGACATAATGAAAATTGATAATGCTATTCAATTAAATAATGTTCGTGAAATGACAGTGTATTATATAAAACAAGTATTTAGCACTAAGGAAGTATTCAAAATTAATAGAAAGAATGCCGAGAAGATTGAAAATCCAAATGACATGTATGTTGCCAAAAACATAACCAGCATCAATTATCATGAAATAGTCAAAGAAATTAAAAAAGAAGACAATTATTCCAATATAATAAAACATTTGCAGGATGTTTACGGCATAGAGTTAGAATTGTAAGCTTGTAATTGACATGTAAATGAAATAATTAAATTAAGAAGAGAAAGGACGCCCCCCCCCCCC

> SrFV1

TATTTAAGTTATTAATTGAGCTGGATCATTTTGTCGGAGATTTGATGGATCTCCCTGGCTGTCCTGGCCAGTCTTGGATGTGCTAAGTTGTGGGTAGTTTCCCTAGGTTTCATGCCTGCACTCTTTCGGAAAATTCCGAGGTGGATTAAGGTGCATCTGGCTGAAAATAGTTTTAAATCACTCGTCTCCATCGCGTTTTGTTCATTTAAATCATGCTTGGTATCAAGCCTGATAGTGCCTTAAAGACCCTGATGAGCCCTGGCACGAAACGCACTTTTGTGCGTTGGTTGATTTTAGTTGTTTTGTTAGTATGTTTTTGGAAGCTTATGCTTTGTTTTTCATACTAAGTGGTTATATAATAACCAGCTCAGAATCTCATAACTTTCCGCCCGCCTTTCCTGGCTCTTGGGTGGAAGGTGATGACTCTATGTCTCCATTAGATTTCTCTTTCAACCTTATCACTTTTGGTGGTAGGCGTGTCAGGGAATACTATTATAGGGGTAAATTTTTGGCAATGGTTATGCCATTAATTGTTATGGCAGGTGCCATTTTGACATTCCTTTCGGTGTGTTGGCCATTCATGGGTGTCGCCATTCTTATTCAGTTTTTCGGGGTTGTGCCCGGAGTCTCAATGTTTTATAGCTTCATGCTTTTGATTGCGTCTGGATGTTCTTTTGCTCTGGCTGTCTTCTCATTTTATTGGATGGACATAGTCTACCCAATCCTAAGTGCGGTCTTTGCATCGAGTGTTCCGGCGCGTGTCTCTGGTGTGTCAGTTGGAAACAGAGCTGCTGAAAGGCTCAGGGCAGAGTTTAGGTTATTCAAGGCTGAACCACTTTTCTTCAGAGGAAGAAGGTGGAACCCTGAAACCATCTCTCTGGCTGAAGATGACGACTTAGCGGAGTTCTGGACGGTTATGTCATACTGGCTATATGCACCTTCGGTGGTGCCTGCGGCCTTTACTGGTGTAAGTTCCATAACTATGCTTGTTTTAATTTTCAACCAAAGGATTTGGAGTGCTGTTCGCTACTCTACACGGTCAGTTTGGTGGACCATGAAATTCTTGTGGGCAGCTGCATGGCTTTCGGTCACTCTCCGGCCAGGTCTATTGATGTGGCTGACCGATTTTGTGATCGGGTTTTGGCGCACCTTCTTTAGGCTTGTCTGGTTTTACAGGTCTAACCCGCGAAGAGAAGCCCGCGCTGCTGTCCGCTTGGCCATTGCCATAGTCGTGATGCGCACTGTCCGTCTTATGCAGATTGTGTCACTCATGGCTTTTAGGCATAAGCCGAGTGGCCAGGAAAGGTTGTCTCAAAAGACTAGGCTTGCCTCATATTGGAACAACACCATTATTGACCTTGGAAGGATTGTGGACAATATCGCTTTGCCACACTTCATACGAGGTATTCCTACATTGTTTGATGCTGATGCCATTAATAAAACCAATGAAATCATGGCTGAATTGGGATGGCCAACAGCACCTCCTGTTGGATCCATACCAAATACGGACCCCCTTAATTTTGAAAAGTTTAGGGAGTATTTTATTGGTGGAACGGAAATACGGCAAGGTATTAGACAATTGCGCCTTCAGGTGTCGAAGGAGCTTGAAGGGCTTAAAAGTTTGGCTGAAGAGCCCGGGTGGAAACATACTAAACAGTATGCGACCATCGAGAATGAATTGGAATCAGTGTCTCGCTATTTCACTGTTGATCCCATTGATGTGCCTGATGTGGGGGTTGATGAGTTGTGGGTTCTCATTGGTAAGATCTTTGAAAACTCAACTCTGACTCCACTTCAGGCCATTATTAAGAAGTGGGAGAAGAAATATGGCCTTGGGCCATTTTACGGTGACCCTGATTCCAAAAGGTGGCGTAAACTTTCTAGACGCAAGTTTATACGCACGATTGGAGGCATGGATGAATTCTTGAAGCTTGTGACCAGGACTTTTGAGATTGCCACATCTCTGGTTCCAGTTTCACCGGTGTCAGTCAAGGACGAATCGTTGCCTCCAAAAAAGTGGATGAATGATGTTGTGCGCACAGTGGTAGGATCACCACTTGCACACTACTTCTCGACCACAGTCTTCAACTTTCAAGTCAATCACAACTTCCAGTATTGGTCTACAAACGTCAAGATCGGTATGCCCCTCAATGGCTTCAATTTAGCACGTCTCTTTGCGGAACATGACGGCTATGACAAGCATTTTGAAGGAGATTTTACCGGCTTTGACGCAACGGTACATTCTCAAGTTGTGGAAATGATAAAGAAAGTGCGAAAGAAGGGCTTTGAAAGGCACAGGGATTATGCTAAAATTTGTTATCTCATAGATGCGAATTATGCAAATCTCGAGAAGATGCCCTTGATGACCACTTCTACTGGAAATATCTATAGGAAAAGCACTGGCCTTAGTACTGGCCACAGTTCTACCTCAATGGATAATTCTCTTGCTGTTACCATTTATTATCTTGCTGCTTGGAAACAGCTCACAGGACTATCAGCACATGAATTCCGCCATTATTGCAAGCTCAGCAATTATGGGGACGACCATATTTTATCATGGTATTCAACTGCTCCCGCCACTTGGAACCCTCACAATATCATCAAGGCCATGAAAGGCTTTGGTGTGATTCTGAGGGACGAGAATCCTGGTGCTAATCTCATTGATATGCGATTTTTGGCAAAGAGATTCAGACGCATAACTTCAAGTGATGTTTCAAAGCTGCAGTCTATGGGTATTCCCATCCCTTATGTTGCAGTTTTGCATGACCCTACACGGTTGGTCGGCAAAGCATATGCTCCTTCCAAGGACCATAAGAAAGACCGATTTTACCGAGCTAAGCGTTTGACATCTTATTTGTCATTAACTGCACATCAACCGGAGTTGTATGAAAAGATTCGTGCTGATATTGACCGTATATTAATTTCCAATGCTGGCAAGAAGTTGAACCCTCCAGTGCCCATTCCAACATATGAGGATGTTCTTAAGGCGTGGTACAGGCCTGATTCCCATATTCAGGAAGAGGATGAAGATGACCCATCTGGTCAAATCATGGATTATTCTGTGAAGGGACCCTTTGAGCAGCTAGTTAACCTTGTTGCTGTTGTGCCTGACCTTCTTAATCCATCAATTTATAACATGGGCTATGTCGCATGGTTTATCAAGCAGTGTGGTGTGCACGTCCATTGGCCCACTGAATTGCTCCGAAGGTCAAATGGTTGTATTACTGATTCACACCTCGTTGCCACCATGCGTAAGACGCCCTATGACTTCTTGTCTAATTCACCCTTTGTGACATCAGCACATACTGCTCATTCTGATGATGCTTTGATCACTAAGCATTGGCTTTTCCTGCTACTCCGTGGATATTTACCATCCACGAATGTTGCAGATGGAATTGCCTTTATAGACAAGAAAATTGGTAGTGCGAACTTTGCCTTAAATGCGTACGTTCAGTCCATTGTCAAAACATTGGACATTCCGTTTGCAGACCTGGCTTTGGTCGCTGCTCTTGACTTTATTCCGGATCTCCCTTTTGTTGGGCTTGTCAACATTATCCGGATACCATCTTTTTCGTCACTAGTTTCTTATTTGATTGGGGTTGCCTACACTGAAATGTGGGCACTTGTCCCTGCAAACATGAAACAGGTGAAGCCTGCATTGGATAGTCTGGGTGACTCCAACCCGTGTGTGGTCGTCCAGGCTCCCACTGGTGTGGGGAAATCCACGGCCTTAGTGGCCTATATTTGGAAATTCTATGGTCACAAATATGACCGTATGATCATGGTGCTTCCACGAGCTGTCTTGCCCGTTTCGTTGGCACCATACCTTAGAAATTCATTTGGATTACCCGCGCAGGTGGTCGGAGACCAAATGGCATTTGACTCAAGTTACAGGTTGATTCTCACAACACCTCAGGAGGTACTTCTTCATCCTGATTGGTTGACTGAAAACAACTTGTTCTTCGTTGATGAATGCCATGTTGCAGAGCCTGCCATGCTGGCAGTGCAGAAAATTCTGCGCCACTTGGTGAAACCTAGGGTGTTGGCCAGTGCCACACCTTCAGAACACAATAAGGACGATGGCCAGGTCATACCTATGACATTGGCCAATGTGCGATCTGTTGAGGATCTCAACTGGCGTCGTGAACCTTTGAGCCCTGAACACACATTTAACGACGTTATTCGTCATTATAGAAATTTAGTGGTTGACATTGTTCGCAGTCAGCCGCTATCCAAATTCCTCGTTTTTGTCGTTGATTTAAAGGAGGCTGAGGCCATGGCGTATTCCTTGCCATTGTCAACAACTATCCTTTCATCGAGGAATAAAGTGATAGACCCTAAAGCCTCGGTTGTTATTGCAACATCTGTTGCAGATGTTGGTTTAACTATTCCGTCGGTCGATTGGGTCATCACACACAACATCCTTCGTAAAGCACGCTATAAGGGTGGGAGGACTGAAACATACCTTACTAAGGCAGATCAGTCCACAATTTTGCAACGCAAGGGGCGTACTGGCCGTACAAACAATGGGGTTTTCACACTCATACGTTATGAGGGTGAAGTCCCATGGATTGAGGAGGCCAGTTATCCCTCCGAAAAACAAGTTGGTTTAGAACTGCTCGCATCTGGCATTCCGGCCCAGATTATTTCGCAGTTTTATCCCGAAGTGATAGCACACTTCTTTGGAAGTGACTATACGCGTGAAATGGACAAACCTATAGAAGATTTTGTTCGATTTTACGCCATGTTTGACAGTGCATTGCACAAACACAATTATAGATCCTTTAAGCCTGATATTGATGAGCCTGGATCAGCGTCACTGTACACCGTTCAAGGCAACACTCTGCCGTCGACGTGGGTTGACCGCGGCAAGATGCCAGATCGTGACGGGATTGCTTATCCGACCAGACCGGCAACTGCTGAAGCAGTATTTAAGGTGATGGTTGCTGCATCCAAATGGCTTGCCAGTAGTGATGAATCTGTCACTAAAGGCATGAAGGAAGAAGACCTTGTTGCTTATTTGCGCCTCCACACCTTTGATCTACGTACATTCCAGAGGTATGTAGAACGTAATAAGGGATGGCGGTTCAATTTGAGCGCAAGGTCACTTGAGGGTATTGACCCTACAGGTAGGTTCGGGACACGTAGCGGTTTGACCCGAACCCAAGGCTACTTCTCTGAAGAGGAGTTGTGGCAAGGTGTCGACATCAACTGGGATGTTTAGCATACCAGTTGAAAGTGCCTCAAAGGGGACTGATGAGTCTTGGCACGAAACTCTTCGGAGTTTCCCCGTGTGGGTGTTTTGATTCAATAATGTCTACTGCTGCATCTATGCTAGCTGACGTTAAGGCGCTTACAAAGCTTGGTCGTGAGACTAATGTTTTCCAAGATTTGCCAAAGGCACAAGAAGACATCGAATGGAGCAACGCTTGCAAGGTTGCTGCCTACACAATTGACACTTTCCTTGCGGCTGAGGAGTTTCCTCCCCTTACCAAGTACGCTATTTCAGAGTACAAGGGTAAGGTTGCCGAGGCCATGAGGGATTTCTTTGAGATGTGGATTTCTAACGAGAAGCCGCATTCTCCTGAACTTCTCCCTAAGAGGGCAAAAGCTTATATCGACTGGAAGGCTATCGCGGCCCAGCACGTGCCCGCGAAGCCTCATGTCGAGGAGGCCCCATCCGTCGGTTCTGCATCACCTTCTGATCCGGAAGATGAGCCCTGGATTGACGTGGAAGTTAAGAAGAAGGCTAAGGCTAAGAAGGCTAAGGGTAACACCTTTGCCAACAAGGTCAAGGCTGCTTTCGTTATTGGCCACCCTGTTGCTGATGATGTTGCATCAGTAGCAGATACCGTCCACTCTGTCGTCAATGTGAATCAAGGCGTCGAATTGTGGACTAGGAATGGCAGGCCATCTTCCCTTATTTGGGTACCTCCAGTCAAAGCTTTGTTCTTTGACGGCGAACGTGTGAAGAACACAGAGTTCAAGAAGGTCCTTGCATCTGGAAAGGGTGTGTGGGAGACAGTCGAAACTGAGGGTTGGTACAATGGCAGTCGTTTGACTGTTGAACAGCTCCAAGTTTTGGCTGGCCGTGGCCTCATCCCCGAAGATCGCCTTATTGGGCTTGCTTCGCAGTCAAAGGCTAATACTGGTGACGCCAGCACTCAGGCACAAGCTGAGGTCGCTGATGTCACGAAAGAGCCATTACAAGCACAGGCTGCTAAAGTTGCCTATTACGCTGCACAATCTGAAAAGTTGATCAAAGCTCGCATTCCAGCAAAGATTAACTTTATCAAAGTGTGGTTTAAAAGGCTGTTAACAGTGCCTGAGTCACTCGACCCTGTTCCTGTGGCCGAGAAATTCCATTGGTTGCTCAAGAAAGAGTGTAAGAAAGAAGGCCAGATTTTGCCTTCAATCAATAAGCTCTTTTATTCGTTCCTCAATGGAAACCTTGCCAGCTGGAAGGCTGGTGAGACAAAGAAAGCGTTTTTGGAACGCACTGATAAAGCCCGCAAGTTCTGGTGGGGTAACCAGGCCACCCTATACCTTGACGAGTTCAATTTGTTGCCCTCATCTGCGGCACTTGTCCGGCCCAAGACTGGCCTCTTGATTTCAGCAAAAGCTACCTCGTCCAAAGTTGCGGAAGAGGCCCCCAAGTCCCTTAAGAAAGGCTTGGAAGCCGCCGTGGCCAAGGGCAAAGCTGCAATTGCTGCCGTCAAGGAGAAAGCCTTGGAGAAGAACCTTGACACTGTGGAAGGTGCAAAATTCCACGCAAAGATTCTTCGAGAGCACTTAAAGAACAAGTCACGTGGTCTTGTCACCAAGATGCGCGGCTTCTTTATCGGTGGTGATAAAACGCCATCAACTATTAGTAAAGTCAGCTCCGCAATGTGGGGATGGTTTAAACGCCCATTCCAGATTGTAGAGCAGACGCCTGGTGGTCCGAAAACTTCCTACTCAGTTTTCGCTCCATTCAAGAATGTCTATCGCTTTGCTAAACAGTCGACTTCCTACTTGAAGTCAAAATTCGTTAATGCCATTAAATGGCTAGGCTGGCACTTCAATGATATTGATGAGCCAGAGTGGGTGCAGCCAACACCCACATCCGAAACTACCGTAACAACGTAGTTTTGGTTTTCTTTTCTTTCTTTTCTTACATTACATCAACTAATATATCAAGGGGAACGTTAAGAAAACGGTCATATGAAATACGGTTTAGAAAATTTTCCCTGTTAAAGGTTTTATACTTCCCGTATGAGTAGGTTCATATGGTACGCCCAGTGATCTTTTCCTCATATACA

> SrFV2

TTGGGGCGTTTTGGAGACTATTCAGTACGTCTGGAGTTTTCCAAACATTCCTTTTACGATGCGATCAACCTGTTTCCGCTGACGCAACTGTTTTTGAACCAATGCCTTAAATTAACCCTGACGAGATATGGCGCGAAAGCAATCAGCCTCTGCAAGTTTTACTTGCAGTGGCACAGGTTGCTCGGTTCGAATTCTTTATTCAAGATGTTGCAATTTGCATTATTGCTCATCTCTATTTGGGGTACATCCCTACCTGTGACCATACCGCCAGAAAATCTGGTAGCCAAGTTCCCAGGTGAGATACCTGATACTGCTGGCCCTGACTTCCCCATGTTTGTGGCAGGGATTGTTAATTGGGGCATGCAGCAGCATGTAGCAGAACGTTTTTCCCTAATCGGGTTTTTGATTTATGCTACTTTTGTTGTGATGGTGACGCTGTCATTGCTGTCGGCGTTTTTGCCTTTCGCCACCATTGTTTTAATGTTTTGGCTTATGGGCCCAATATTCGTGACCACTTGGGCAGCGACGTTGTCCATCGCGGCGTGCATCCTTGTGTCTGCAGCGGTGTCTTCGCTCCAGCCTATTTGGATATTTGTCGTTAATTGGATAACAACTGAATACCTCAAGTTGCACCCGGAAAACACTGACCGATTCAAGTCTTACGTGGAGTCCGTGGCCCGCAATGTGAAGGCGGCCGCAGATGAGAACCGCGTTGAGTTCATACTCTACGGAGGCCGCATTTATGACCCCAAGGCCATAATGATGCTTAACGAGGAAGATGTGAATTTGACGTTCGCCATCTGCACAGCCTGGATTTGGTGCCCGTTGCCCAGGGCAGGACTGCTGCCTTTTGCTATTTGCTGCGCATTTTGGGTGTTTTGGTTCAACAAGAAGATCTATGCCATGGTCAGAGCCTTCCTTACAGGATGGATGATCTGGACCAAAATAGCCATTAGTTTCCTTGTGCTCATATTCGCAGTTAGCCCTTCGACTCTGGCATTGCTTTCAGACTTGATACTGTTTGTGATCTCATTGCCGTTCTGGCTCTTGTCAATATTCAGGCATGGGGGTCTTCATTCGTCGTACCAAGCTGCTCGTTTGTTGGTGCTCGTTGCAGTCCTCCGGCTCCTAAACGTGGCTTTTAAAACCAGCATTTTGTTTGACAAACAAGGTGCTGGTCCCGTCAAAGGGATAAGCCCTGCTACGATGAAGTTCAGGGCAATTTGGAATAATGCCATAATGGATGTCAACCGGGCAATAGACCAAATTGCCTTGCCACATTTCATTCGCACATTGCCTGAACGGTTTGACGCGGAAGCCATCAATGAAACGCAGCAAATCCTGGCCTCACTTGGGTGGCCTGCATCAGACCCAGTTGTTCTGGATGAGCCTGTCAAGCCAGACAACATAGGCACATATCGTCATGCATACCTGGGCACTGTGAGGTCCATTAAGCAGGGGGTCCACAGGCTTCAACTCCAAGTTGCAGAGGAACTTGATAACTTGAAAGGGCTTGCGCCTGAGTACAAGCGATCTGATGAATATGTGACTATAGAGAATGAACTGGAGTCATTGGCACGGTACTTTGATGACCCTGCCATCACGCTTCCTGACCTGCCAATCTCCGAGGTTTTTGAGTTGGTAGGGGATATCTTCCGCAATTCGAGACTCACGAAATTCACTGTCATCGTCAAGAACCTAGAGAAGGCATACGGGTTGGGCCCGTTCTGGCGTGACTACCGCGTCAAGCGCTGGAAGAAGCTGTCAAGGCGCCAGTTTATAAAGGATATTGGCGGCATAGGAAATTTCATACGCCTGTGGGCTCGCACCTTCGAGAGGGCACCTGGCCTTGTGCCTGTGGCCCCTGTCTCCGTCAAGTCGGAGGCCCTTCCACCTAAGAAGTGGATGGCTGACAAGGTCCGCACAATCATTGGTGCTCCCATAGCGCACTATATAATGAGCACAGTGTGGAATTATTGGCCTAATCACAACTTCAGGTACTGGTCCACCAACATTAAAGTCGGGATGCCACTTAATGGGAAAGTCCTTGGCATGTTGGTTACCGAGCACTCTGCTTTTGATGAGCATTTTGCTGGTGACTTTACAAACTTCGACTCTACTGTTGTTGGCAAAATGAGCCAGTTGATAGCTGAGGTTCGGAAAAAGGGCTTTGAGTATCACCGTGACTATGCCCGCATTTGTTACCTTATTGATGCGAATTACAAAATATTGCAGAAGATGCCTATGATGACCACATCAACAGGCAACGTTTACGATAAACGTACAGGCCTGTCGACTGGCCATTCTTCGACCAGCATGGACAATTCATTGGCCGTGACAATATATTATATAATCGCATGGCGTGAACTCACTGGGCTCAGTGCTCATGAATTCAGACATTACTGCAAATTGAGTAATTACGGTGACGACCATATTCTTTCTTGGCGGGCCAGTGCACCAGCCACTTGGACTAGGGAAAATATCATCAAATGCCTGGCCAAGTATGGCGTTGGCCTTCGAGATGAGGAACCTTCTCATAAACTGGAACGCTTTGAGTTCCTGAGCAAGAAGTGGCGACGCCCAACCACTGCTGACATAGTCGAGCTCACAGAGGCAGGCGTCGCCGTGCCTGCTTGGATTGTGTACCACAACCCAATTAAGCTCATTGGCAAGGCTTATGCGCCCTCCAAAGACGTGAAGGTCGACAGACGGTATAGGGTGAAAAGGTTGGTTAGTTACCTTTACCTCACAGCAGGGCATCGCGACTTGTATGACAAACTTCGCGTTGACATTGAGAAGATCCTCAACAAGAAGGGTTCTGTCATGCGTTCTCCTGTACCTATTCCGTCTTACACCGAGGTTGTAGCTAGGTGGCATGATGAGAATAGTGTGGTCGGAGAACCAGAAGCGGACCGTCCTGAAGACATTAATGACAAAGCAATCGTGCTGGACTATACCATGAGCCTCGGATTGGATTCGCTGCTACACGTGGCCTCAGTCATTCCTGACTTTGTCAATCCAGCAGTGTACAACATAGGTTACACGAACTATCTCATTGGGCTCTTCGGTGAAAGGGTTGCCTGGCCTATTGAACTCATACGCCGGTCGAATTCGGCAGTGACGGCGACCCTCATCGTTTCACTTCTTAAACGGTCGCCTTATGACTTTCTGGCAGATCACAACCGGTTATCGACTATGTCAATTGTTGCCGGCAATGGCACCTTGCTGTTGCGCCATTGGCTCTTCATCGCGCTAGGGGGTCCACAACTCACCCCGAACACTTTTCAGTTGCTAGCCTGGCTTGACAAAAAGATAGCTGCGTTGAACTTTTTGATCAATGGTCATATTCAGACTTTCGTCAGGAGACTTGATTTGCCCATTTTACAAATCATTCTCATTGCAGCACTGTCTTATGTACCCGACGTTCCAGTTCCCCGCTGGATTTTGCTTATACGCATACCTAGCGTGAGCTTCCTAGTCGAGTCATTTTATGGGTATTTACTCAACACATTCTGGTCGAAAGTGCCGGCTAATATGAAGCAGGCATCTGCGGCTCTGCAGCTTCAAGGCCCTGAAATGCCTGCTGTCCTCATTGAGGCACCTACAGGGACAGGGAAATCGACTACTTTCGTGAATTTTGTATGGCGCAATCACAGGTTTAATTATAAGCGCGTCATACTGGTAGTGCCCCGACAACTGCTCGTGTTGACGTTAACGCCGTACTTGCGTGATGCCTTTGCCTTGCCTGCACATGAAATAACTGAGGGCCATTCATATGATGAGACTTATTCTTTGATTGTGACTACACCACAAGAAGTGTTGCTCCACGAATTGTGGCTGACTGAAGGCAACCTCTTCTTGATCGATGAGGCTCATGTTCTTGAACCCCCTCTAATGGCCTGCATGACTGCAATACAGCGTATGCACGCCTGTTATATTATGCTTACTGCCACTCCCTCTCAGTCAAACATGGACGTGGCAAACATACATGTGCCATTGCAAATAGCCCAAACTTGGACAATCGTTGACCAGGAGATAACTGGCATTTTCGCTGACACACCAATATCAATGGCACTGTATTGGGAAGATTATCGTGCCCGTGTGCTGTCACTTGCCCGCGCCCATATGTTGTCCAAATTCCTGATCTTTGTGGTTGGCATTGGCCACGCCCAAGATTTGGCACATCGGCTTGGTCGTAGATGTTGTGTGTTGTCAAGTCAGTCTAAGGTCATTGATCCAGAGGCTGAAGTCTTCATAGCCACTTCTGTGGCGGACGTTGGCCTCACCATACCTGACGTAGACTGGGTCATATCAAGCAATGTGACCAAAACCATGGTAGCTGAAGGCACTTATGGTAAAGTCAGCTTGGCAAAAGCTGATGCTGCACTACTGCGGCAAAGGCGCGGCAGAACAGGCCGAACAAAATCAGGCATCTCAACAGTCATCAAGTACGGTGACATTATAGGTCAATCGCCTGTGGGCACTTGGTCCGAAAACCAAATCGGTGTTACCATGCTCAAGTCCGGTGTTCCTCCCGCAGTAGTCGCCAGGTTCATGCCAGGGGCAATAACTTCATTATGGGACACTGAATACACACGCAATGAGGACTCATTAATTGACTCCTTCGTCGCGAATTCGACCGTCATGTTCAAGGAATTGGAGCGCGAGCACCAACGGACATTTGCGTCTGCTCTTGATGATGACTTCGATATGGGCGATTTGTGGACTGTTCAAGGCAACACAATCCCTGTGCGCCAGAATTTAGTTCCGAACCGCAATGACTGGGATGCGAATATCGCACCTCCTGCCACAGCACATCAAATGTTTGACTTTGTTGTCGGAGCTTCATTGTACCTGGTCAAAAACCATATGACTATGAACCCCGAAAAGTTCCGCAATTTCTTGCGCACCAATATGTTGTCAAGCAACCGTTTCATGGGTGCATTCTATGCCCAATCTGCAATGGATGAGCCGATAGGCGATGTGTCACATCTGGGAGATGAGACCGGGTCATTTGGGCGTTATGTGGTGGCTCGCAGCCGCAGGCGCCCTGAGGCGGACAATGCAATCATGGACGGTTTCCATACGTTTGGAAAGCCCATTGACAGAAACGAACTTGTGGGCATTCCTGTCCCTGAACCGTCTGCCTCCACCAATAGTGACCTGCCTCCCTTGACAGGTGACTATAGTTACAAGCCGGTGAACTGGTGGGAAAAACCTGGCGCTGCTCCGCCATCTTAACAATTTCGAATTCAATGCCTTAAACAGACCTGATGAGATCTGGCACGAAACCTCATTGAGGTTGTCTGGTGACGGACGTTCCCAACTCAATGTCTGGTGGTAAATACATCAACATCGAGGAAGCCTTAAAGGGCAACATTGAATTTATGGAGAAATATTCTCGCTTTGCAACAGCACCCACTGAGATGCCGTTTGATAACAACTTGCTGGACTTAGTGGCGAAGTTTGTCCTGGAGTCACCTGATTCACACCACATGCTGTTCACTGCCATGCCTGGCGGTCATCAGACCAATGAGCTCGTTGCGGCCCGTATGACTGCGTTCATTTCGTCCGCTGAGATCCTCATCCAGTCCGCAGGCACATCCGGCATTACTGAGGCTCAGCTTGGGGTTGTCAAATCAATTCAGCGGAATGCCTTGAACATTAAGAAGTCGAGGGTTTACGTTCCGGAGGTAGTCGAAGAAGCCCCAGCTTTTCCTCCTCTCCCTGCAGTCATCCCAGACCACATAAGTGAGCGTTCATCAGTTGACATTGATGACCGTAACAAGCGTGCAAAGGGCTGGACCAAGGTCAACAGGAAGAACAAGAAAACTGCTGCACCTCGGCCAGTTAAAGCACCCTCACCACCTCCGTCAGTAGCTGACTTGCCCGATGACAGGTCAACTGCAGGCGAGGTCAATGTTGGGACATCAATCTTCCCTGTATCTCAACGCCGTGTGTTGGCTTTGGTCTATGAACCCCCGCGCCGCCGGCCTTTTGTGGACGGCAAGCGTATCAAAATGGGCGACGTGGCCAAGACCGGCACCGACAAGGTTGAGTGGAAAGAAGTGTCTGGTGAACAGTTTTACGACACTTCCGGCATGTCCACTGCTTTCCTGATGACTGCCAGGGCAGACAAGCTTATCCCCTCGTCGATATACACTGGCCTTGTTGGTAAGGCAAGTGATGACTGGCGTGAGGTCAAAGTTTACACTGAGCCCGAGGCAATCGAGGCCTACTCCAAAGGGGCAGTTAACGCTGCAAAGATCGCTTACTACTGGGCTCAAACCGAGAGTGAGCTTAACCGTGCCAATTTGACATACAAGCAGGTGTTCTTTGACACTTGGTTCCCCAGGTTGATGGACTTGCCCGCCAAACTTGATGGGTCCACTCCTGCAAGGCGATTGTATGTGTTCACAAAGATGGCATGTAAATCGAAGAACCCCCGAACGTCATTGAATAAAGCTTTAATGGGCTTTAAAATGTCTCAATTGATTGTCTGGAAGGGTGGCGAAACCCGTGCAGCAATGATTGAAAAGTCCAGGAAAAGCTTGACGGCTTACTGGGGTGACAAAGCTGATGACTATATCAAAGCTTTTGAGGCATCCCCGGCCGGCGAGTTCGACATTAGGCCCAAATACGGCAACTTGGCTGTAAAAGCCAAAAAGCTTATGATTGACGCTGAGCGCAAGGCCCGTGCCGACGCCACTGCCCTCAAGAACATGTCCGCCCAGGCCATCGCTGACTTCACTCAAGCAATGAAAGAAGAGGACGAGGAGGTCACTCAACTTACTGAGGGGCTGTCCTGGTTTTCACGCTTGAATGTTAGGTCACGACGGCTTTGGTCCATTGTTACCGGCAAGGTCCTTGGCAAAAGCGAAGCTGAAGATCGGCCTGGCTGGCGTCAGGTCCATGAATCAGTTTATGGCCACATCGGTAGGATCTTCTCCAAGGCAACCGCTGGATTGTCTAGGTCAAGCCGATGGCTCACGTCAAAACGGGTCATTAGTGCTCAAGTGTCAACCACTGAAGAGGGTGAGTCTCAAGATGCTGACGCGGCTGTCAGCATCACTTATGAAGACCGCCCTTGGTTTGCTTGGGCAAAGTATTTGACAATAACTCCAGTCAAGACTGCAGGTGGTGCAGTCAAAGAGGCTGGTGCGGGATTCATCAGATGGGTCTCGCGTACTTGGTATGAATGATGAGGTTTCTTTTTCTTCTTTCTTTTCCTTATTTATAGTATCTTAAAATCCACACCCGGAATGTTTATTTCCCTTCTAT

> SrHV1

TTGGTGGGTGGTTCCCCTGGGTTTGATCTCTTGAGATCCTTACCAGGGTAGCTACTCATCCCCACAACAGGCTTTTTCAAGCATCTGTTGGCTAGTCATCCTCCATCAAAAATGGATGACTTGCACGGGGACCTGACCGCGTAGCGGATAGGTCAGAATGTGAGAGGTGTGGTAAACCTCTCCTAGTTCGGAAAGCCGCTCTTCTTGCACAGCGGCTTCAACGGCGGAGTGAGCTGGGTGGATGGTGAATCCAGCGCTCTTTGTTTCAGATGTGCTGGGTGGATGGTGAATCCAGTGCTCTTCGGTGACGGACTTTGGTGTGGTGACGGCCTGTACAACCGTGTTCGAGGGTGTCCGCGTGGTGGTGGCGCGGGTAACGGAATCAATCGGCGGGGATAATAAACCTGTCGGTAGGGCGTGCCCTCCTCCTTGATACTGAGTTCTGCTCTCCTCCGAGCCTAGGAGCGATCCAGGCGAAATCTGATACAGGGCCTAGGAGCGATCCAGGCAACATTCCAACCCCACTGTCATTACAGATTCTTTGGCCATCCTTCCGGCCGTCCTGTCTTTACAACCTCTCCTTCCTATCGTGGTATATTTCCACTCGCTGAACGCTGCGACCACTGCAGAGGTAGGAATCGACCTGCGGGTCTCTGGCGTTGATCAGAGACCTTAGACGGTCGTGCACGATCCTATGAGTTAGGGGCTATTAAGCTATGGGGGGGTGAAAAACCCCTGCCGAGATGTGCCCGCCTCTCTATCTGAAAGCAAACCCTAAAAGTGAACAAAGCGACTTACTACCGGAGGATTACACTGCTGTAGGCCTTTCGAGGCCGTGTGTTGGGGCTAGGCATCCGCGAGCCGAACCCTATTAATCAATCAGCAAACGGACCTTTGTCAGGGATGCGTTAACCCTGACCCGAGACTATCCGCTTGAGGCCCATTTTAGAGAGGACCGGCGGACCGAGACGCCACACTGGACGTATTTCTTGCTGACCGGCCGGATTGCCTGCGAATCCCGCTTCAACAGTGAGGGGAACAGTCCTGCTCAGCCTCTGCTGACAATACCCTGCCGACGTGAAACATAGTCGCGACATGGTGCGCTGCATACTTTTGTGGGATCACGCCCGAGCTCTTCGATACCTCAACGTGAACCGGCCGCGGGTGTTTTAAAAACATTTCTTATGGCGTCATTAAACACAACCAAACCAAAAGCCGCTGTCAAAGTGGCACACCCCAAGTCTGGAACACCAGTGGCTTCATCACCACGAGGTGTTCTCCCCGTGCGTCTCTCCGAGACGAAAAATGCAAAGGTCAAGTTCCTGCCGCAAGGCGACAAGACCCGAGCAGGCAAGCCCGTGGCGGCATATGCCACAAGGACCCCTTCCGAGTCCACCTCCGCGGTTTCCCCTTCCGCCACGAAAAAGGGTCAAGCCGCGCCATCAGGCAAGGCCAAGCCGAACCCGGGCGGTTTACCCGGGTATGTGAAAGCGTGGGGTGCCAAGAAAGGCCTCACCAGTCCCGCCGCGATCCAGGCGGCTTGGGGCGAGGGCGGTACGACCGTCCGAGAATATCATAAGGGGGCTTCAGGCTTCCTTTCAACTTACGGCGTTCCGGGTGGCCTGCCCCGGTTCGCGTTTCAAGCCTTCTGCCGTGTCACTACTCTGACGGCAGGACAGGCGACTCATGTGGTCGAGCCTTACTGCGACCGCGTTAAGGCTTGGCTCGATCAAGAGCTCGCCAAACCTCGTGGCGCAAACTTCGAGGACGACATCAAGGCCTCGCTGTTCACGTGGCTCGAAACTCAACAGGACATAGTTGAGGAAGAGCCCATGCTGAAGCGAGCGCGCTTCGAGGAGACGGACCTCGTTGATCACCGTCACATCTTCCTCGCGGACTCCTTCCCGGGTCTGCGTCGCATCGAGGTGGCCGAAAGAATCACCGGAATCGCACCACAAGGGTGCCGAGGCAGCACTTCCGCTGCGTTCTCTAAGTGGTGGCTCGGGAGCGCCGCCATGCGGTGGGCCAACCAGGTCCACGCAGAGTTGGAGAGACGGGCGATAAAGATGCAGCAGGAAAAGGACGCGGAGTGGGAAGCTCCGCCAGTCGTGGCGGAACCCGTTCCTTCTGCCCCTTCTTCCTTCCCGGTTCTGGCACCGCCTGTGCACGGCTGCCAGACCCTGTCTCAGGCTCTCGCCGCGGTGGCTCCGAACGCCAACTTGCGCCGCTTCCAGGCCACGATCCGGATGCGGCTGACGCAGGCTCGCCTCGCGCCCCAGCTCGAGTCCAGGAGGATCCGAGATGGAATCTCTGGCTGGGCTCCTCATTATGGGGATTACCTGTTCAACCATGTCCAGGGTCACAAACCATCTGCGGAGCTCCGTCTCTTGCTAGCTCTCCACGAGCAGCACGAGCTGCGATCGGCGTATGAGGAGGAAGAAACCAAGGACGCAGCGGCAGCTGCTGTAAATACCCCTGCCGAAACGACGGCACCGGACGCCGCATGTTCGGATGTCGTCCACATCCCCGAGACGTTGGTGATCAGGAAAAGTCTGACGCCAACAAGCGCCCCCAACTGCGCGCCATGGAGGCCCGTGCTGAACGAAGCTCGGATCGACATCATCGTCGCACGTGGTTGGGCTCGCGCCCGCAAAACGGCTGCTCGCCGAGATAGAGCACTCCAGAAAGTCGCGGAAAAGGCCGCGCACACAAAAGCAGTGTCTGAGTCCACTGCCAGGAGCATGAGGGCTGCAAGTTTCCAACCGGTGAACCTCTTTGAGGGAGCACCGTCCCGACGGGAACAGCTCTGCAGGCGAGGCCCGGCTAACTGCCAGCCCTATCGCCCGCTTTCCGCTCCGGCAGCTGTCTCAGCGCCGAAACAAGAGGCAGAAGACTGGTATCAACCCAGGCCTCGGGTCGAGTCTGTCCGACCTAGAAAGGACAGCGCCAGCCACAAGGGTTTGGTCAAGCTCGCACGTACTCCGGGATATTGCTATGTGAAAGCAGCCCACCCGCGGCACTGGAGATCTCTCGCCGAGCAGTTCGGCGACCGCATGACCTTAGGTGGTCTCATCGTGCTGGGTTTCTCTCCGTGCGTCCAACGACTTAGCGTCCGAGGAGTCAGCTGGTGGCACTACAGCACGCTGGAGCCGCCTCTCATGGGGCCCCATACGGCGGCTGACTGGCACTCTCCTGTGAGCGGCGTCTTGCCTAGAGGAACTTTGTCGGTGCGTCCGGAGGCTTTTTACGCGGACCTACCGTCTCCGAAAGCAGGTGATCTGCTGTGCTCTGGTTCCCTGGCCTCATTGGCTTATGCCTGGGCACCCACCCGCGAATTGGGCGATGAGTTGGTACTGACGTTGGAACCCAATGCCATGCATGAGGTGGTCGAAGTGACCGAATCGGCTCTCGAATATAGAGCCCAAGGTTCCGAACAGATGGCTCGCCTCACGGCGCCAGTCTCCACCAACTTGCAACGGGCGCTAGACACTGCGGCCTTCTTCCGTCTGCGAGCGGAGTTACAGCTCGAGAAATCCGAGGCTCATCTTGCTTCTCTGTCTCATGATGACTTCAAGAGCTACAAGCTGTGCAAGGATCATCTTATCGACGGTGAGAGCTCCACGACGTCAGCGGAGATGGAGGAGATCCACGATGCCTTCGCAGACCCGTCTTTCTGTGCGACTCGCTGCGGTCACAGCCATGAGTCCTTCTCAGACAGAGAAAGCTGTAGGGTGTTCTGCCACCATACCCACGAAGACTTCGCTGACTTTCCTTCCTGCACGCAACGGATCCTCAGCGAGGGTGTGCGCGACATGAGCAGCACCATTGTGGATGTGGACGAGGAACCCGTCGAGCTCCCGGTATCCTCAACAGCAGACCAATTTTCGATGCTGGTGGAGCCCGTTCTCCAAGAGCCCCAACACACCGGTCCGGTCGTGCTTTCTTTGATGCTGAATCAGCTCATCGAGCACCTTAACACTCGGAGGGTCGTCACTGCGCAGACAGAAAGGTTCATCTCACAACAGACTGGACTTTATTTCTACTCTGTAGAAGATGTCTTCCTGGGAGAGCGACGAGTGGCTGTGAAGGTCCAAAGGACCGCGAAATTGAACATGAATTTCGCGCGTCATGATCTCCTCTCGTTGGCACCTGACGCCTGGGTCTCTGGAATCAGAAACGCGGCTCTCGCTGTTGTCCTGAGAGAACGACTGAGCTCTTCGCACATGCCCGGCCGCGAGTGCGATGCCCACTGCAGCCACCACAAGACAGCCACTGGCGGCGTCTACCCTGCGGCGAAACGACGGGCTTGCAAGCAGTACTGTGGCCATGAACCCCTAGACTTCCCGGAATACGTGAATCTACGGGCCAAGGTCAAGGACTTGGAAGACCAGCTTGCAGCACGTCCAGCTCAAACGGGAGTTGAGGCGAGGATCGTCGAACTCGAGAACGAGGTGGCCATACGGGACCGAGCCGGGTTGCTGATGGAGAAGCGCATACGTGAGCTCGAGAGTACTCCGAAACCCGCTGCGCCTCCTGTGGCAACTGCTCGAGTAGATCGGATCAAATCTGCTCTGAAAGCTTGGGACGCTGATTGGCGAGCTCCGGTAAGGGCAGCCCACTCGAGAGAAGCTATCACGCATCTCGGGTCGATCGTATCTGAAGCGCCAGCCAGTGCACAGCTCTTACCCCCTCCGTCAAGGGTGTTCAAGCCCAATGTGCTGTCCCGCTGGTGGAACAACGTGGATCAAGCCATCCAGATCAACGATCGGACATTCGCGTGGCACAGTGCAGGGCTCAAGCCCACGGTGCTAATAGGAGACCAGCTGGTCAAAGCCGACCCCTCTGCTCGCACGCAGTATCGCTCGTTCGTCGCAACCTGGGCGTTACTGGAGTACGCTTATGATCGCCTCAAAGGAAGAGGCAAACAACCGGAGTTCTTAGCTGAACTTCAAATTACAGAAAGCCAGCTCAGAGAGAGAGCTGTAATGTCACGTCGATTGGGCTATTTAGGAATGGCCCGATCAACATATCTGAAGAAGTGGGACAGAGCCACGACAAAGCCTTCTGACCCTGGCCCAGGGATCGCAGATGTTCTCAGGCTGTATGCCAATGACACCCCGTTCCTGTCTTCTCCCCAGACAGACAACTGGAGAGGTCGAGAACCAGCAGGTAGGGATCGTAACTCGCCCCTGATGCACGGAGTTGTCGACGAGCCTGTCCCAGAGTACGTGCCGGAGTGTGTCGTCGACTGGCTCGAGTCACTCCCCTGCCAGCCCCCTTTGACCATCACAGCCGGGTCTCGGCCACAAGACGAAGTGAAGGGTGACCTGCACCTCACAGAGGACAACTCTCCTTTCTCCGGAAGGGGTCTTGCAGCTGCTCGTGTGATCGCAGCAGCCTGGTACAACACACAGGTGCGGATCCTGAACGGCAGACCGAAGTGGGAGAATGAGTTGCCGGACATGGACGCAGAGTCCTTATACCCGACCCCATTCTGTCTCGCTGACTTCGTCTGGGAACCACAGAGCGACTACACTCGTGTGCGACTCATCACGTGGTGTTGGCACAAGCGACACGTCTACGGAGCGGATGAGGCGGCATTGGCGAAGTACTACTCCAGGAACCGACCCCACACCGTTGTGATCCCTTCTAGGCAGCTGACCTCGGTGGCGCTAGACGCCTTGAAGTACTGGCAGGAGCCCCATGTCCGCACAGGCTCATTCACGCTGTACGTCAAGCTTCGGGACGAGGTTCATGGACCCTTCTTCGTCAACGACATGTCCTTCGCAGGGCTCGTATGTCTTGTGACCTCTCTGGGGAAGAAGCGACTAGATCCTGGTCAAGGGCCAGCTCGGTACTTTGTTGTACCCAACGACGCAGGCAAAACTCTGCTGTGGAAGCGGCTGGGCAACCGGGTGGTGACCTGGCACGACGTAATCGACGCCTACCCCGATGGCGGAGACCTGGAAGAAAAGCTCCTGTACTGGTTCGCACGAGGTTCGACGGCTTGCGTCTTGCAACGGCATTGGTTTGAGCCTCCTGACGACGCAACCCCGCTTGGGACTCTTGCCCTCGTGAGCTCAATAGCCTTACGCTACCACCCCGTCGACACCGCGGTCGAAGTTCTTACGGCGCCAGATCCTGTTTACTACAGCAACGAGTTGCAGGATCTGGTTCGAGGCTTCGTCACTCAGACCCGTCACGGAATGGTGTTCCACCGTGCCACCTGCCGATGTGGAACTCAGCTGCCATGGGCCTTCGAGCTGATCGCGGCCGTGTCAGAGCCTCGATGCCCTGGTTGTCACATCCTGGTGGCAACCCGCGAACACGGTGAAGCAGGTCTGTCTAGTGCCAAATGGGTTGACCGAGTCTCTTGCGTCATGGGGTCACCTTGGTCTCTCGCCCCGGGGACATACAAGACGGTCAAGACAACACGTCAGGTGCCCGGTCGGCCAGGAGCAAACAACTATGCATTTGAGCTGCTCCGGCTACGAACCAATGCAGCTAGTGTAGAGGTAACCTTCCCCTCACACCATCCCGCTATCGGTGTCGCTTCCGGGCAGACAGCTGGCTTCCGCGGAGATGTGGCTGACGTAGGATTGTTCCCCGTCGAGTGCCTCGACCTCATTCAGTGCCAGGATCCTGCGCTCCTTGGACAGTTCACGCGTTCGACTTTCAGGCAGGGATGCACATGGCCAAAGAAGTGGCTTGAGATGCGCGTGGGGCTACCTTATGTCCCGTTCGAATTGGCCCTCCATAACGACGAGGTCACCATCTACCGTGGGCCTGGTAAACCGGGAATCCACCACACGAGCTTCAAACCGGCTCACATGGTTGTGCCCCAGTGGTTGTCGGACATGGTCGAAGGACTTGCTCGCTTGGTCAAGAACAAGAGAGTCATATCCACGGCCAATCCCAACTGCCCTTTGCCACGTCGTAGCCGTGTCGGCTGCAACTTCGGAGACCACGTTGCTTCATGGAATACAATCTCCGTGCCTCCCGAATTCCGGGGACGCTGCTATGCTCTATGGCGATTACTCCCCTTATACGCCGGTGCTCGCGAGGTTTGGATCATCAATGGCCGAGACGCTCAGGAACAGGTGGAACCATCTCTCGTGCCAGGGAGATTTCACTCGGCTAACACCTGGAATGCGATGCCTATCATACTGGCGTCAACGAAAACCGACGTCGAGCTCCCAAGATGTGGCCCAGGATACTGTGAAGATCTCTCTCGCCCTTGCCTCGGACGATTCGCGGCGGCGATGCACCTCTACGGAAACACCTACGGAGTCGATGAGTGGCTGCTTCAGCGGGCGCCCTTGACATCGACCCATTACCGGCAGTTGTCAGACGTAGACTTTCGCATCCTCCCCCTGGACGATGAGTTCCCTGATGTCGAGATGTCGGTGCGCGGAGAACCTTACTTCCGCGGGAACCTCAGCCGGATCAATTTGCTGAACCAACCAGCTCCACGACCTTCTGGTAGGCTACTGAGCTCTTTGGATCGAGCAGCGGCTGTCGCGGGGGACTTTGGTCTTGAGGGCATTCGAGTGCCCAGACTAACCAGTCCTTTCGACAAGCCCCCGACTCCGCGGAAGGACACGCTCCTCATCACGGTTCTCGGCTCATACGGAGACTTAGTCCCCATGGAGTACCTGGCGCGAGTCATCATCGCCTCTGGGGTTCCTGCGGTGTTATGGGTGACCCAAACACTAGAAGCAAGTGACTTCGACCAGCTGAAGGCAGGCAACGTGATCCGGGCTCTGCCCGGCTTCCTGGCGGCTATCGGCGCCCACAACCTCGGATATAAGGCAGTTCTAGCCCCTAACTTCCGGATGTCGCTCAACACCATCAAGTATACTTTGGCTAACTGGGAAACGACTGCCCGGCCTTCGTTGGTGGCGAATCCCCAAGCTTGGCTGGCAGCCTTCTTGATCCAATACGTCGACAAAGGGCTGGTCATCGGCAACACTCCAGGGTGCAACACCCCACGCTCAGCAAACGGACGGACCATGTTGCTTTCTCAGCAACCAGCCCAGACTCGTCGCCCGTGCGCCTGGGTCGAATGTTCTGACGGTGTCCAAGCGGTTCCGATGTCCATCAGGAACTCGATGCCTGGCATCACACGGCCTTATGATCCTTCATGTTTCTGCGAGTACGAGGTGATTTATTGCTCCGGATCCCAGGGTGTGGTCGACACGATCCTCGCTCATGGAGCGAAAGCAATGCTGTTCGACGCGTGGTTCGACCAGAAACGGACGATAGCTCTGGTCCCGGAGGTGTTCACTGAGCCCACCTGGCAGGCACTTGGGGAGGCACTCTATCATCACGGATTTGAGAGTACGCTTCCCAAGCCCAGCTGGTCCAGACGTTACACTCAGAGGATCCCAAATCTTCGACAGGGTCTGCGTTCGGTAATTACGCTCGTGACCACCAGCCAGTTGCTCCTCGCGTTGGCTCCATGGTTCGGCACTTTCCCGAGTCTTGCTCTTGAGTGGCCAGGACGACTTGCATTGCTCCATCCGGTCATGAGGACATTGATGAACGTTCCTCTGTTGTACTCCTGGTGTGGAACAAAGAGCGTTGCTTACCTCATCATCTTCTGGTATCTCTTTGAGCAGATCCCTCTGGTCGCTCTTGGGATGCAGGAGGGTGTGAAGCTCCGCCTCGAACTCAACCCAGGTTCATGGCTCTTCGTCCGACACGCGTCCTTGGTACACACCAAGAAGAAACAGACGATAGAATACGGCTGGTACGGCGCGAGAAACCTCCTCGCCCCATTCCAAGGACGGGTCTGGAATATCATAACGCCCGTGAGCCCTCAGACGATCGAGATCCCTGTCGCAATCGACTTCGAAGCTCTGAAGCGGGACGCCCTCAATGACGTCGGGAAATACGGTCCGTTCTTCAACTGCCAATCCCAGCTGCTGACCAAGCTAGGGAACAATGCCCCAGCAGTGACGATCCTGGTGTTGAGCCTCGTCTATGCAGGCGCATTGGTCTTTGCGCCGTGGTTGCTCATCGTCCTGAGTCATCACCTTGGTATCAAGATTTGTGGTCGCAAGCCCATCGATCTGATTCGCCTGGGAGACACCGAAGCGGACGAGATTCCGTGGTTGCTGGCTGAGACGTTCGACGACGAAGATGACTTGCCAGCTGACGTTTCTCCCGTGGACGATGTCCCTGCCGAAGCCAGTCGTGACTCATTCCTTGACCTCACCGACGAAGACCAAGCCCTCGCTGCGCTGCAATACGTGTTCTCGGAACTGCCAGAAGACGAAGTGACCCCTGACGCTCTCATCCTTGACGAGGCCGTTCAAGCCTGGAAAGCCATGGTCGATGTGCAAGAAGACCAGCCTCTAGCCGAGTACTACGAGCCTGTCGAAGGAGACTCACTGACCTCATGGGCAGAGGACTTCGTTCGATGGGCTCTCCAGGAGATCCGCACGATCGGGTCGGCGGCTCCCATCGCCCGTGATTTGGTTCGATTCCTCTTCGCGCTAAAGGACAACGTCGTTTGCTTCGCGGAACCAATGCTCCAAATCTTGAGAGTGGCTTTCAAAGCCATCGTTGAGCTCACCCGTCAGTTGGCTCCAGAGCTGGTTCGTCTCGCGTCACGATTGGTGGACCTTGCTTTCGGACACCAAGTCACGAGGAAACTGAAGGCTGCCTGGTTTGGGGCAGAGCTCCTCAAAGGGCACAAACTGAGCGTCCAACATCGGATTCGCGAACAGCTCGCCTTCTCACACTTCTTGGCCGAGGGAAACTTCGATGACCAATACAAGAAGGACACAGCTGAGCTACGGGCGGCCTACAGAGCCAGTGATGTTGACTGGGACAGGCTCCAAGCCATGTCCGAGGCGGAATTGGGTTCAAAGAAGCGTTTCGTCCATCGAGGTCCTCGTGGTTTCGGAGGTCCTGTCTGGAAGCCATTGCCGCTGCCACGATCTGCAGTCATGTCAGAGCAGGAGCTCGAACTCCTTATGGCCGAAGTTGAAAAAGAGAACAAGGCCGGAAATCCTGTCAACTTCAATGCAAGAGTTGACCAATACTTCACCGCGCGAGTAGCGCGATTGACTTCTGGCGGGACAGACCTCGCCACAGATGGCGCCCTTTTGGCAGCCATTCGCCCAGCGCAAGCCGAAGCTTCGCTGATCCGTTATACCTACCACGGGATCGAGCCAAATACAGCTCTAGGTACTAACACCCAACCGATGACACCCGAGCGGCGAGACCGACTCTTGGCGATTGCTGATGCCTACTACGAGACCAACAAGGATCTATTTGACGACCCCAAGTTGACGCCTCCTGAGGCCACCATCGAATGGTGGAAGCACCGCGGCATGACCAAATTCAACACGACCGCGCCTCTCAACATGGCCTCCAGGGCTCATGCCATCGTGGACGGGCAAATGACCGCCATTGTTCGGAACGTATACGAGAAGCTCAAAGCCGGTGAGTATCCTCACCAATACTACGCTGCGAAAGTCAAACAGCAAGCCGTCCCGGCTCGAAAACTGGTCACCCCTGACGCCAATGGAGACCTCAAGCCTGTCAGGACCTTCGTCGCGCAAGACCATCGTTCAACGGCGGCTGACTGGACCGTGGGTCTGGAACTGAAGAACCGTCTGCCTGGCGAGAACTCAGGCGAGTCATCCAAGATGGCCGCCGGCCAAGGTTACTCCCCTCTTTTCCGGAAAATCCGCGGAAAGGAGAACATTTTCATGGGAGACATGGCTCGCTATGACAGCCAACTCGAGAGAGATCATTTCTTCATGCTGGACCGTTGCTTAGAACGAGGCGTCTCTGACAAGGTTGTTCGGTCAATCCTCCAGGCCAAGCACGCTGCTATGCAATCATCTTACATTGCAGTGCTTTCTCTGCCTCGAGACAAGCCACTCGAACCTTTCCTCGCTCACGCGCGAGCATCGATGAGAGATTCCACAAAGGCTGCAGTGGGGTACGAAAATTGGATCCTCAAGATCCGATCCGGGGCCACCGGAGAGTCTTCGACCAACTGGACCGACTCGAAGACCTTCCGCTTGACGTTCGGACTGATCGTGGCTGACTACTGCGACTACTTCGGTCTCCCCTTCGACGCTCACGCATTCTTCGGCGAGTCAGACAAGTACAACCTGACCAATTCCGGTGACGACAACATGGGTCATCTAGACTTTCTTGCTAAGCAAGGGCATGTACTGAATCCGATTGTCATGGTGGCTATGGCCCGAAAGAACAACATGGTTCTTGACTTTGCGGTACTCGACACCTTCGACAAGTGTGAGTTCCTCGGCTGTATGGCACGAGTTCCCACTCAAGCAGACCTTCGGACTCTTGACCGGGTTAGGGCTCTGTTTAAAGCCGTAAATGTCAGATCGGACCTGGTCAAGTACGCCCGTCTCAACGAGGACCTGGTAGACCCTGCCACAAAAGAGAAGCCGGACATCATAGTCTATCGCAACTTGACGAACTCTCTGGTCAGGCAAACCGCTACTGTGCTACATGGCAACGTACACAAGAACGAAGTGTACCTCGAAAAATGGTTGGCCAAGTACGTGGGACATCTCCAGCTGTGTGCCTTCTCGCCTGTTCGACATCAGGAAACAATGATGGAATATGTTCAGACAGCGATGCGTTACTTGTACGCTGCTACTCCGAACCATCGACGAGACCAGCAAGGAATAGTCCTGCTACCTGAGGGCGAAGAGTATGAGAAGATTCTTGCACTCCTCAACCTCCAGTACGAGGACGTCAATAAGAGGCTATTCTACGCCCGTCCCAAGTCCTCGGTTGCCTCCAAGGATCCATGGTACACGGCTGAGTTCAGGAGACGGCTCAATCGCTTGTCCCAGCAGCCTCCCGCCGCATACTCCAAAGTCATCGGGATTCACATGGCCTACGGCAAGAAGCCCAAGGAGTATTACGAGGCGAAGATGGCCAAGATCCTCTCAGGGCTCTACCCCATCGACGAGACGGCGAAGCTATTCTTGGACACACTGCGATCAGCAGTCGGTGAAGCCTCTAGGAAGCTCGTCAAAGGTTTCCAACCCCAGGCAAGGGACCAGATGTACCTTGAGGACATCTACGACAGCGGCCACTACCGCGTGGAAGCAGCCATTTGGCTCACGTGGGAAGAGAAGGTCATGGCTGCCCAAGCGGACGCAGAAGAGGATGACATCGGCAGACCTGAGATGACCCTACCCCTATGGCAAGGCTTGTGTTCTAAGGCAGCTTTCGGCTCTATCACCGACCCGTTGAGGTTTTACGCCCTCATGCAGGACAAAGACTTCGCCGACAGCATTCGGTCAGAAAGGGCTTATGCGTATCGCAACTGCATGTGGCTCCTCGTCATGTTCTACGTCCTCCTCTGGCGACTAGAGGTGTGGATACACAGCGTGCCTATCTTTGGCTTGTCGTACTCACTGTTCATGTTCATGGTCATCGACATGTCCAAAGTCTACGGTCTTATCTCGATGCTGTATTGGCTGGAATACATGGATGTCCATCCGGTCATCAGTTCATGGATGCCCAGAGACCCGTATGTCTTAGCCAAACGTCTGGCTAGCTGGCAGCTGAAATTCGTCCCTATGAGCGTGGCACGTCTTCTGCCGTTTGCACTCACGTCGGGGCTTCTTGGAGATGGTTGTGCCTGGTTCGCCCAACTTTTCAACCGGGCGCAATCTCTAAAGCCTCTCGCCGGTCATGGCACTCCTATCGATAATCCATGGACTTCTGATGCCCATCGGATGTTGGATCTTTTGTCCGACCCTGTCAAGACACCGGACAACGCCATAGTCCTCCGCTCGGCCACTGGCACGGGAAAGTCAGCGCTCGGGACCGATGCTCTCACGAGACTCTTGCGCCATTCAGTCGCACCCCGTGGAAGAATGTGGGTGTTTGTTCCGACCCGCATACTTCTCAAGGACCCTATGCCGGCATTCCTCCAGCGCGGGCAAGACGGCGACCCTCAGCACAAGAAGACTTACCAAGTCCTCCGGCGGGGCGTCGCGATCGACCCAACCGCTGAGATACTCTTCATGACGTACGGGCATGGACGTAATCGCTTACTGTCTGGAGAGTTTCAGAAAGGGGTGGACACGGCATTCGTCGACGAGATGCATATGCTCTCAGCCGAACAACGCCTTGTTTGTGAGAGCCTCCGTGGAGAACGGATGATCATGTCGAGCGCAACTGATGTACCCCCGCCAGGATTCGTCGCACCGGTGTTCCAATCCTCTCAAAGAAAGAGATGGAAAGCCAATCGAGTGATCTTCCCGGCCTCGACCAATGTGGCCAGCATGTTCCAGCGAGCCCACAGTGACACTGCCCCTGTCCTTGGCATGAAGCACAGTCCGGCGGAATTGTCGAAGCGCACCCTCATTCTGTGCGCCTCATTCAATGAGCTCGATAACGTAGCTGAATCCTTGATCACGCTTCGGAAATCGATGTTCGGCGGAGGCATCGGAGTGTCCTTGCCCCCGGTCGTCGAAGTCTCGTCTCGCATCAAGCCAGGAACGGAAGCATGGATGGATCGCCAGCGAGCGTTCGAAGCTGGAGAGTATATAGCCCTAGGGACGAAACAGGCGGCCACCGGCATGGATATCAAGCCCAACCCTCCTTGGTTGTTGGTTGATGGCGGAGAGGACATCTACTCCCACGAAGGCACGATCGTCCGTCTTCCCACAACACCGCAGGACGACGAACAACGCGCAGGCCGAGTGACCCGTAACTCTTCGGACCGTGATGGCCTCATCTACTGTAGGGAACAAGCCGGATCCCGGCCATGGGCTACAGTTGAGTATCCAGCAGTCTCGTACTGTACGGAGTCCCTTATCGCTAAGGCATACAATCTCCCTCAACTGCTCCCTGTGGAAAACCCCGCTCTGGAACTATGGCCGTACTTCCGGCTACAAGAAGGGTTCGAGACACATGTCCGCGAAGCGCTTGTGTTTGCCGTGTTGGCTTCGGTGTCCGGCGTCACGCCCGCCAGGCTGGAGGAGTTCTACAGCCGGCACTGGCAACATCAGATCCCATTATCTGATGATTACGAGTGGATGGACACATTAATGCGTCGGCAAGGTCGCCGGTCAGCTGTCTCAGCCCCTGCTTGGGTCACGGTCCTCTCGGTCCTAGGCCAAAGACCAATAGGTTGGAACACTCATGGCATCCACTCTGGCGAAAGGAGGTCTGACGGGCTGGTCTATGGCAATATGCTCTACCCCGTCGCCGGGGTCTACCTGTCCTACGAGGAAATGCAACGCTCTCAAGGCAAACGTACATTTACCTCTCAGGAGAACAGAGCTGAAGACATCGCAGCCGACATCATCAACCGGCAGTCGGACGTCATTGACCGACTCCAGCGCCAGATCCGCGCCTTGGAGTCCAAGAGAGAACCGACACAGCCGCTGTCAGCAGAAGAAAAGTCATTCTTCCGTTCTCGAGCGGCCCTCCGTCACGCGGTGAAGATGGCAGTCAAGGGACACAAGGCCTCGCATCTCAACGACCTGGCAGACTCCATTATCATGAGGTACCGCCGAGCAGAACGACCTCCTGGAGGCGTCTACACTGGTCCTGATCTCTCGGCCGTAATCTCGAGCACCGAGGGCTGGGGTTATTGCACTCACGACGGCACGAGCGCGGTCATAGGAGAGTCGGGACCGGCCGAAGGCAGTACCGAGACCCTGCCATGCGGGCATAACGCTCACGCCACCGGAGACCACGTCATCGCCGACACATTCTGGGTGGTCAAGCGAGGTTCTAATTGGACCTCAGCGTACGCCGTAGCTGACACAGAAAACAGCCGCAACCGGGTATTGGCATCAATCACAGACGACGATATACGAGAAGGACAGCTGGCAATGCCTTTAAAGACGCCAGTCAGTACCGATTCTGAGCCCGAATCGGAAAAACCCAAACAAAGCGGAAAACACAAAACTACAAAAACAAACAAAATAGGAGGAAACGGCACCAGAGCACCTGGTGGTCGTCGCTCACAATGAAGCAAGAATCTGTAGAAGACCATCTATAGTTGATGGTAGCCATGGTTTTGGAGGTATTAATCGTGGACTGCTGGACTAGCTAGGAGACTAAGGTCGCCCATGAAAAACTAGCACCGAAAGGTTATTCCAGTCGGGTACCTCGCTCGCAGACGGTGAAATCTACGGGAGCATAACTAGTGTTCTCCTGAGTAGGTCTCCGAAACCAGAGCAACTCGCACACAGACGCCGCTTCAGTAAGCAATGAAAACCTACACTTGGACAACGTACTCGGCCGTCGTTAATTCGACGACTGAGGTGCCAGGAGCCATCCTTCATTGGATGACCCGACATGGCATGGGCCAGGAGCCCCCCTTAATTGGGTGACCCGACATGGCCCCCTCCTAATACAAAATGGCGAGACCCAGAGAACCGGACAGCTAAGCCAAGACGTGGTGACCGCTTGAGTAATCTCTGGTTAGAGTTTGAGAATCTGTCGAGGATACATCAGCAATGATGACACAGGAGACGAACGCCGAAGCCACCAAAAATACCAACTAACTCACATTCTTTTGAACATCCCTCTCTTTTTCTTTTTCTATTATGAGTTCGCTTCAGCAACACGGCCCAAGATGGGAACTGAACGACATCTAACAGACGCTATTCAAACATAAGAAACGAAGCCACGGAGATAATTTGTACCATAGGGTCGAAAGATCCGGAATCTCCCGAGGTAGCTCAGACGCAAACAAACGCGTACCTGTGCAAGGCCCAACGGGCCGACGGTCGGAAGCGTGGCTGAAACGCTACGTCACAATCAAGTCGATGTTTTCGCTGGCAAGCGGAGAAGGGCACAGCCCTTCCTTACACTTTTCATTCGACCTCTGAGGCGACCTGGGTTGGCTCATTTGTACCCACGATTGCATTCAGTGGCCCCGCGAAACTATCCCCCGCCAAGGGTGTGAAGTACCGGTTCATCACTAATCGACTGTTGTCAATTTCGTATCAAATGTTATTCTAGCTGACCTGTTAGGACTGTCCATTCCCGCCCCTCCGGTCTTCTCTCATCTTGAGGAAGGCTGGAATGTCGATATGGTGTGGCAGAACTAGTCAAAACCAAAAAAAAAAAAAAAAAAAA

> SrHV2

TATTATAGCGTTTTGGCGTAAAATCTCTTGTTAACCATGTTGTTTTCTATTACTCCTTGTGACGGTGTGTCCGTGTACATGTCTCGGTCTGGCGCATTGTGCGTCAAGACCGCGACTGGAGAAAAGAAGTCTGTTGGAGAACAGACTGTGCCTGGTTTTGCGGTGGTGAGACCGCTGGTTTTAAATTATGTGCCTGCATCCAGGTTTGCAGAGCACTGTGATGGTAGGCACGCTTGCCGACACCATGGTCGTGCGTGCCCCGAATTATTGGCGGCACGTCGTGCTGCCGAAGCGAAGCTGGAGGCTCGTCTCGCCCTCAAGCGACGTGCCAAGCAACAAGAAGAGGCTAGGTGGGCCCGGATCCGTGCCGACATGGCTGCCGCGAGGCAGGCGTTTGAGGCACGTCGTGACGAGCTGTGGGAGGAGTTTTCGCATGCCCCGTTCTCTTTTAGAGATGAGATAGTGTGGGGCATGCTGGCGTCTGAGGCGCGAGATGCTGGTTTTGTCTTTGGCAGGGGCATGTTCCTTTCTCACAAGGAAAGAAACAAATTGCAGCATGCGCTGAATGGAAATATAGAGCCGGGCAACAGCGCACGACGACACTTCCTCGCGAAGCCAGCAACCTGGGCCCAAGGTGTTGGGCGGCATTTCAAGATCAGCAGCGCGTCACGCCGCTGTTTCAGCGCGAAAGCTGATCGTCCTGTTGTCCTGGATGTGGAGCCATTGGTCGGTTCTCCCGAACCGCCCGCGCCTGCTGAGACGGTTGTCCCTCCGCCTTCAGTGCAGCCAGTTGTCGCTCCGCCCTCAGAGAGGCGTACCTTGACTGTGTCGACCTCCTCGAGTTCGTCGGTGTCTGCGTCGATGGTTTCGGTCATGTCCCGTGTCGAGGAGAGACACGTGCTTGCTGGGCCATCGAAGGCTGAGCCCATTGACCCCTTTACCGACGACCAAAGACGGTCGTACATCAACTACCGTCGCATCGCCAAGGAGAAGCTGGTCACACCTTACACGTTCGTACGCCCTAAGTGCTGCCCTCCTGATCCGAACTATCCTGGACGAATCGAGATAGTGATCGGGGAGGGGCAAGGCTATGTCGTACTTATGTCTAGCTTCCGCGACAAACCTTTAGTGACTGTGTGGGCAGAGCACCATGGTGTGGTGCCTTTGATACCGTACGACATATCGTACGGGGCGCCATGGCTAGCTACGTACCCTGACCATTTCCATGGCCATATAGTCGCAATGACTGGCAACAATGTCAAAGGCGTAGCGGTCATCCTTCCAGCCAACACTCCCTTTCCTGACTTCGCTGAAGCTGCGGCACGTGGGTTGGCTATCAACTACAGAGCAGCCGTCTACAAGACACCCAGGAAGGTTGTCGTTGCCGTCTGCCCTAAGTGTAAGAAGCTTTTGCCAATTCATCCTGATTTGACCCAATGCGAACATTGCTGGGCTATTGAAGGACCTGAACGCGATCGCAAAATACCTGCGTATGTTGCAGCTATTCGTGCGAAAGTACCGGAGGCAACCCTGGCACAGAAATTGACTATGGCCCCCCGGAACTTCCGGAAAAAGAAAGGCTTCGGTCGCCCAGCTCCAGTGGCTTCCCCTGTCACATCCATCTCTAGCATCCTGGAGTCGAAGCAGGCTCCTCTTCAAGAGAAGGTTGCGGCTGTCCTTGACTTGATACCCGAGGTGGTTGACCACGCGGGTAAAACCTTTGCAGAAGTCGCAGAACCTATTGTTGATCGAGTCTACAATGGGGTTTTTGGGCCCACTCCCGGCAAAATTATTGAAACGGTGGTGCGGCCTCAAAAGAAGGGCCGAGCACCGATAATGTCGGATGAGGAACTTAAAGCTTTGGTGATGCTAGTTGGCGCAAAGCAAGCGCTTGCATCGATACCCACGGCGAAAGTCCAGACCCAACTGCTAGATGAATACCTTCGTGAGTGTGTAGTGAATCCCAACCAGCACACGATCTTTGTCGATGCTATTATGGCATTGAATCCCCACCGTCGTCGCACTGCAATTGAGCTGGCGCGCTTCCCGTTGCCCGTCCGGGAGCAATTGACAGAGGAGCTTTTCAAACTCGCTAGGTCCTCTGCTGATGCGGATATAGAGGTGAATCCCGGTCCGTCTTTTTCGATTAGGCGACTTGCAGAGTTTGCAGGGGAGCACGTTACCCTTCCGATAGGCAGTGGTCACGACCTGAGGATCAAGGTTGTGCCCCACACCGCCTCGGCCTCTTGGGTCCCTTATGGCCCGGCTCAAGGGCTTCATGACGTTATGCCCCCAAGGTATGTGCCTGCCAGCCCTTTGCGCACCCCTGAGTGGGTTAAGGCTTTTCGCAAGGTCACAACAAAATGCCACGAGGTCGATTGCGGGAAGCGGAAGTTTAACACCACCAACATCGACGAAATCGCACGTGAATTCGGAGGAGTTGGTAGTTTATTTAATGTGCTCTTCTATGGGGCGTATGAACTGTGCTCGCACATGCCTGGCAAGTCCTGGCTATGGCAGAGCATAGAGCGCCGCGCTCAGCCAGAAACCTTCATGGATGGCTGGGCAGAGGTCTTCGACCGATGGGAAGGCGAAGGCCTGTGCAAAGTTAAAGCACATGATGCAGATGCCGTTATGGCGGCACTGAGAGAATTGCCCAGGTTCTCAGAGATGCCCTTAACTGACATGGACCTGGAGTTGGCCGAGAAAATGGGTTACGGGCACGTCAAATGCCCTTTGTGTTGGCCAACAGATCCATACTGGTGCCCGTTGTTCCACCGAATGGCTGGGCCAACACAAGCTGACGAGCATAAGCACATGATCGAGTTTCTTCGACTTGGCATGCTCTTTTGGCGAGGGGTGCCTGATGACCTACCAGTCTTAGTTGTGACTGACCTTGGCGAGTTGCAATCATCGCCTCGTGCCTTGGAAGGGTTCCGCACTATCCATATCACGACTGATAGGGACAGACGAGACACACCGGGCTATTATGTCGTGCCAGCTCTTCAGCGACAATATATGTCGCCAATGATACAGGCAGCTGCGACCTTGACCGGGCGCCGACGCCCTGTTTTGTATTGCAATGAGCTGCAGATGCAGCGACTGCCGACATCTGTTCTTTCCTTGCCCCCCGGGGCTTATGGCTTGTATGATGTTGATGGACAAAAGTCCATAGCCGCCTTTTCGTGGCCAGAGGTAGATTACACCTGGGAGAACGATGGCCTAGCCTTGGTGTGCCGAGCCTTTGGTAGCTACGAAGTGGCTGGCATACTAATGAGAAGTCGTGTTGAAGGCAACACTGTCTTTTGTGACAACGTGGAAGTCTTCAACGCTTGGAAAGTCAAGTCGGAGATGGCTGCGCCGCCTGTGGCTGTGCATACGCGCAACAATTTCCGGCTTCTTTCCAGGCCCAACTTTGTTGTGCGCACCGCGGGGCTGGGGCATGCCTTAGGTAGGCTAGCATACATTTCTCTTTCGGGTGCGACTCTATGGTCTGGGTTGGCGCCTGGGACTATGGCTTCTATGGTGAGCGGCTCAGCGTTCAACCCGTCTCATGTCGGGGCTGCTCTCGAAATGACGCTGGATGAGGCGCTGGTGTGGACGCCACCCCCACGTGACGCTTGCCCTTGCACAGGATGCCTCTTAGCAAGCTATTATGGCCCATTAGTGTTTTGCACTGCGGGCTCTGCTGGGGACATAGTACCTGTCCGGGCAGTGGCGAAAGCCCTGAGCCGGCGAGGGTTCGACACAGTCGTTATCGACTGCCTTGACGGGAAGGGCTATGATTTCTTGAAAGCCGTCCAGAGAGGGGAGGTCCTGGCGTTGGGCCCTTTTTACGCTTCTGCGGCCGTAGCTTGCAGAAACTTGCCCTTTATCACCATTGGGCCTCCTGAACTGGCCTGCAACATTACTTGGAGTCTCGCCCCTCCGCCGAGTGCCATTCGTCAGTTCAACTTTCGCCTCGGCCCGCTTTTCAACCCTTTGTTGTCTTGGTTTGCGGGTGTGACGCAACCAGTGATTAGAATTGCATCTTATCCTGGTGCCGCCCATTTGCCGAGGTCGGCCGACGGAGAAACTTTCCTCAAGAGGACCCCTCCCTCTGAGAAACCCCGTCAACATAAGTACTTAGTTGCGTTCGGTTCCTCGGGCTACCCAGTTCCCCCAGGTATGCCTGAGGTGCAGCCCGGCGACCACTACTCACAGTTTCTTGAGGCCGAGGCAGTTATTTGCCATGGTGGCGCCGGGACAGTGCAGACTGCAGCGATGGCTGGCTGTCGTGTCATTTCGGTGGACAACACATTGGATAGGGATTACCTCGACCCAACTAATTGCCAGGCTGGAGTAGGCGCAGAGCACCATGAAGACAAGGTCCTTGGGCTCCTGCTCGGAGTCGACTTTTTGCCGTTCTTTATTGCCGGAAAGCTGCCGGGCTTTGGACTACCTTCGATGCTCCACTGGTATGCATCGGCTTATCTTCTCTCCGATATCATGACATTGTGGGCTTTGTACCGTATCTGCGCGATAAGCCTAATTGTGGTACCCGGAGACTGGGTTTCCACTATAGTGGCGTCTTTGGTAGGTTCGTTTTTGCCCGGCGGTTTCGCCGCGCTAGCCGCAATAGTTATCAATGTCTGGGGAGTTTCGATCTTTGCTAGGGCTGGCATAACAGGGCCTGGGCTTATCATGAAGTTGCTGCGTTTCGGTCGTATGGTGCCCCAACGGCCCTGGCTGTGGATCACGTTTTTAGTTGCAGGCCTAAATGCAACTATGTTTGTTGCGTTCATCATGCTCTTCCTGGTGCCTGCAGCTGGTGTGACTCGTAGGGCGTTTGACAACCCAAAGTCCTACCTGTGCTTCAGCCTGGTGGTGGGGTTCCCTTTTCACTGTTTCCTCATGAGTGTGGACGGGACTTCTGTCTTGGAGGGGTCAGTCTTGGTGGATTCTGAAAAAGCTCCGTACAGACTTACAAGAGTTGACAACGTGAAACCGGATTATCTTTTCAGAGTGCCAACCTTGGTAGACTTCAATTCTATACAGGACAGCGATCAACCTTCAGCCTCTTACGGCTTTTTAACACACAATTGCAATACAGTCCTGTATAACGCGGTACGTAACAAGGGCGGATTTGGAATAGGGGCGTCCATACTTTATATGTTTGTCGGCATTGCGTCGATAATTGCAATATTTGGCATAGGATTTGCACTGTTTGTCGGCCTTTCTTCTTTGATCCTTGTTGCGACGTCTGCCATAACGCCGCAGACTTTTCAGAGTACGGTCAACGAGATCATATTTAAGTGGCGTCGTGCGGCCCTAATCGACCGTATTCGGGCGGATAGGACAGTTTTGTCTTCTCTACTGATTGTTGCTCTCCTTGTGACCGACTCGCAGAGTGGGTTGACACCGGAAGAACGTGTCGCTTACGAAGCGATCACAGCTTTCCGTCAGGATGTCGCAGCAATGCAAACTCCACCTTCGTTGACAGAGACCTCCGCCTTGATCAACAAGCATATCGTGCTTGGGCCTTTGTTTCAAGGAAAGTGGCGTACGCTGGGTATCATGTTTGGTATGATTCCCCACGACTTCAATGATGCTGAAGTCGCAGCTATCACAGGCGTCCATCCTTTGATCCGGGAGGCTCTTGAGATTCATGCCCGTTACTCAAAGAACCTTGCAGAAGCTAGGATGAATAAGATCACTTTCCGCCAATTTGTTGAGCTTCGCGAAGCTTTGCTAGAGGAGGACCCTTGTGCCTACCTGAATAGTGGAGTGCCCATTCTCGAGTCTTTTCATGAGATCAATGATCTTCAGACTCGGTTAGAAAACCTCACAGAGCCCTATGACGGTGAGCCAAATCAGGTCGAAGCCATAATCAATGGCATCATGCCTAATTTCCTTCTTGAAGAAGAAGTTCTCAAGGCGTACCAAGCGTTCCCCGTACACGGTGATGGTCCAGTATCGGACAGTGCCTTTGGATCTATCGGTCTCGAGCTCGATGAGGACGAACTCATAGTCTACAGATTCATTTTCTTCTCCGACCTACACACTCCAGAGCTCGATGGGTCTGGCGTCTTTTCGACTGTTAAAGAGCAGCCGTTGACCAAAGATGATATTTCGGCGCTCTCGGTGTTGGTCGCTGCCGCACGTGCTTATGGGGCAACAGAGCTTGAGGCCCTTCGCGAGGCAGTCCTTAATGCTAAGGACATACTGCATCCAAAGGGGCGCGCCGTGTTAATTGGTGCAGACGCGCGAATCAATGAGGCCATAGATGGCATGGTAAGTGCCACTAAGCCTATTCGGGCCTCTTTGCAATGGATCGGAAAGGCTGTATTCCATGCACGGCACTTCCTTCATAGCAATCCTGTTGCATCTGCCACGTTGAAGCCTTTTGATGTCGTCTTCACCCTTGTAGGGTTTTTGGCAGCAACACTGTTGTCCGTTGGTCTACGAGCTCTCAATGCTTTTCTGGGGGCGCTTGCATCAGCTGCCATTGTTGAGCAAACCACTGTAAAGGAAGTTCTGACTGCCCTTGGAGTTTTTCTCTCGACGATGGACCCACGTCACAGGTTCAAACCCAAAAGTGCTTGGGCTTTGCTTTGGGAACGATCCAGGGTTGTTCTCTCTAAGGGTGAGTCATTGCTATTTTCTCTCCGCAATTTCACATACGAGCACAACCCTTCATACCATGACTGGAGTGGCAAGATGGTTGAGTTGTTGAACATCAAAGGGGTGGACTCTGCATCCTTGCATCGTTCGCCACCTACTCGTGCAGTGTTCTTCCCTAGGAAGCCCGTCGGCTTGAAAGAGTACGAGGGTTTGGTTGCCGATTCTTCACTCCATTTCACCGAGATGGCGAATGCTAGACGTAACATGGAGCAAAGCTTGGCCGCTGGAAATGGTTTGGCTATTGATGGAGCATGGATGGCCCGACCAGAACACGTGACATCGTCCTTGCTTAGGTATACTGTGCCCAGGCCATCTATAGACTCTGATGCGAGAACTATGCTTAGCCTTGCAGCCGATGCGATATTCGAGCACCATCCCGAGTTGTACGTGAATCCCATGCCGATGACGGTGTCTCAGGTCTTGGCCAAAAGTAAATGGAAGTACTCTGCTGGCCTGCCGTTCCTCCCCGTCATTAAGAAGCGAGAGACATTGCGACACTCCAAGTGGTATGACGCAATTCAAGCTGCGGCGGAGCGCATTATTGAATCCGGCAAAATGCCCTCAGTTGGACTTCACGCCTTCCCTAAGGCCCAGGTTGTCTCGCTTCAGAAGTTGCTAGACAACCCGGCTGCAGTGCGCACTGTCACTGCGGGCGATAGAATCACAGGGACTGTTTTCAACACGTTGTTGTTGGAAAGGAACAAGCGTGTTCCTCCCGCTCATTACGGGCATGTCAACATGCTACGCCGCAGCGAGGGTGGTGTTGCCTTTCTGGAAGAGCACCTGTCGGCGCATCCTTATTTCTACACCGGGGATGGCCGAGCTTTTGATTCTACAGCTGCCTCTGAAGTTTCAACAGTAGGGTCAGTTCGACTTTACCAGCTTGGCTTGCAGGCTGGGTTCACTTTTAATGAAAAAGCTGCTGTGTCTCTGGTCAAGTCGTACTATGAGGGATTGACTCGTGGACTAATCATCAACCTTTTGAATGGAGACGAGATCATCAAAACTGGTGGCGGCGGTACTGGGTCTGTCGCCACAACCCCGGATAACCGTGACTGGGTCGAGCTTGTCTTCTTGGCCGCGTGGGGTTTGGTAACCAATACCCACCCGTCTACCTTCTACAAGCATTTTCGGCTCGCGCACGCCTCAGACGATGTCCTATTTTCTACGGACGAGTATGGGCGTGAGCACCTCCAGGAGTGGGTCGATTGTATCCGGACCAAGTATGGTCCGGACTTCTCTTTTCAGTTGGAAGAAGGTGTGGACAACCTCATTCACCTGAAGAGAGTCCCAATCTCTGAGCAGGATGCGGTGCTCTATGAGCGCCTAGGTCTGCCTGTTCCTTCAGTTGGTTTCCGGCACGACCCCCAGCGACTGTTATTCTCACGATCAGCCTACAGGTCTGACAGAGCCAAGGCGAATGCAGAAGTGATGGCTTCACACGTGGCTGAACGCAGTGCGGGGTTCCAGTTGCTGACCGCGCATGCACCACAGGAGTACGACCTCGTCACTAGGGATCTTGTCGATGCAAATCTAGAGTATGCTCTTCTCTTCTTTCGAGGTGCGAGAGCATCTCTCGAGCTTGACCCCAATGGCGTGGTCATCGATGGTCTCGTCGAGATCACGGATCGGCGTCCTGCCCGACACGTGAAACGGCTTGCGTCGAAATTTGGAGTCGGGTCCAATGCCGCTGCGTGGATAGCTAACCGGCAGAAAACAGCTGAGTCTCATCTACGTCAAAGACGTGGCGCTAGTTATGCAAAGGTTTTTGAGACCTGGGTCAAGCCACCCCCGCCGACTAACGAATCGAAAGCTTATAGGCGCTGGCTCTCTTACAGGAACGCAGCGAACACTTTGAACCCGCTGATCGATGTTATGAGGCTCACTGCCAACAAGTTGGACTTTGCTATATCTCTGGTGCCTAGTTCTCTTATCAAAAGTGCAGCGGAACCTCTGGCAGCCAGATTTTCTAAACCCTTCACGACTTTCGACTTTGTAATCGAACAGTACATTTATCGGCGTGAATTTCTTAGAACTGGGCAACCTGTAAGTTTCGAGCGTGCTGGCACTCTGGTCAGGGAGTCGCCTTATGCGGCAGCCACCGATCTGGCGTCGTTTTATGCAGTGCTTAGCTACCCGACGGCGTTGGCTGCCTTCCACAAGTCGATTCGCGAGAACCGAGCTGTTTACGACCCCGTGTTTTTCCCGTCTAACGACACAAATGCAGCACAAGTCGTGGTATACATGTGCTGGTATACTGTTATCGACCTAGTGATAAATGCCACAAAGCAGTTCCCGCTCCTGGGGACTATTGTGTTATTGACGTTTGCAGTCTACAGGTGGTTGGACATCTTTTACTCCGTCCAAAGTCTTCTCTTCTGGCTGGCAACAGCGTCAGCTTCTCTTCCAATCTCAAACAGTTCCCCTAAGGATAAGTATGCATTGCAAAAGGTTATGGCGGCAATACTTACTGCTGCAACCCCCTCTGCTTTGACTTGGTACACACCGGGTTTGTTCCGGGCTCTGGCCAAGTTGGGGCCAGCAATTGAATTGCAAGTCTGGGTTCCCAACCTTTTCCAAAGTGTGCATGCGACGGCACTTCAGGAGCTCAAAGACGTGCCGATATCTGCTTTTGGGGACTTCCACAGGGTTTGGTCTAGGGACACCCCTGTGGTTTTGTTGAACGCGCCGACTGGAACAGGTAAGAGCACGTCACTCCCTTCCAGCATTTGCACACGCTACCCTGGCAGTACCGTGTTTCTCCTCCTTCCTTTCAACCGATTGGTTGAAGATTATTCTAATCCGTTCACGCTGCGATTGCACGTGGTGAAAGTTAATAGACAAACGCAGCGAGACAGTATCATCGTAGGCGACATCTTAGTCATGACACCCGGACAGTTTTCGAGGCGCGTCCTACAGTTTAACGTGTCGAACGCCGTTGTCCTGCTGGACGAAGCTCACTTGCCGTCCAGCGAGGCTTTAACTGCTTTTGAGACGTGCAGGACGTTGAACTTACCGACAATCCTTATGACAGGCACGCCGGGGCATTTCCTGGCTGGGGGTCTTTTTGGAGACTTTCCTTCATTTGATGCGCCGCCCAGGGTCACGTATTCCCGTGACGTTATGGATGACTGTGCCAACTTTGAGGAGGCTGTCCATATGTTAGCAAGGACCCAAACACCACTATGGGATGTTTTGGTGTTCGACCCAAGCATAGCCACCCTGGATCTATGGCTAACACAGTTCCAAGGGCTCCGAGTCCCAGTTAAGAAAATAACTGCCTCAATTGGGGATTCAGGTGCCCGCGCAGCTGCGTTTGCGACTGGAGTGATCGCCGTTGGGGCAAATGTGTCCCCTAGCCCTACTGCCTTGATCTGTGCGCCGGAGCATCTTGTCGCTCTGCCTTCGAAAAAGCTCTCGCGAGACGCTGAAGGCAGGTACCTCGTTGAGCAAGACTCACTCATACATGCTGTTGTCCTATCACGCCAGGCAATGCCTGCACATGAACGACACCAGTTGTATTCGCGGGTAGGCCGTACAAAAGACGGCATTGTTATCCCGCTCGCAACAAACTTGCCTGAAGAGTATTCATCATACATCACCCTCGCGGGACTTTTGCGTAGTGGTGAGTCAGGCAAAGTCTGGCTACGATATTGCGGTATAACTGCATTGTCGATTGTTCCACATGCCGCGTGTCAGCTCTTCATCCCTCGCGAGACGAGTTTCCTCGACCAGAAACAAGCGGCAGTTGCATATGGGTTCGCGGCTGCAGTGGTGTCCGTTACCACGTTCTCACAGCTGCCTTTACTGTACTCGACTGCTGAGCGTGAGGAGTCGGAGCGTGAACTGCTTGACTCCTTGGTCGCTGATTTCGGGGTCACCGAGACAGCTTACGACATCCATTACAGAGTCGCTCTTGCAATGTTTTGTCTACATTATAGTCTAGATATAAATGGGAAAGTTTATCATTCCACGATTCCCACTATTCTAGCCAATAAGCTGGTACTTTTCAAGGTATCAGACTTGTTGTGATTTGTCAGCCAGGGAGGAACGATCCCCCTCTATGTGATATGTGTGCTATGAGCAGTTACACACGCCCCCTTCCCGAAGGAATTGCTCGCCGGTCCACGTTGTTTTTGGCTGTCCACCCATTAGGGTGCTAGTGCTTATAGTCTATGCCGAGAGTTGGCACTAGGAAATGCTCGGACGTCGAAGTGTTTTTCACAACAAGCTCTTATGAGTAGTTTATCTTGTTTGTGTGTTGCTGGGTCGACTACCGGCCGATGGGGTGATTTGATCGGCAGACTAGGTTTTAATTTTTCTTGTCCGCTGCCGTGATGTCCCCGC

> SrHV3

TATTATAGCGTTTTGGCGTAAAATCTCTTGTTAACCATGTTGTTTTCCATTGCTCCCTTCGACGGCGTTGCCGTGTCAGTTTCCCGTTCTGGCGCGTTGTGCGTCAAGAATCGGACTGGAGAAATGAGGTTTGTTGGAGAACAAACCATTGTTGGATTTGTGGTTGTGAAACCGATGACCCTGTCTTTTACTAGGGCCAGTAGAATGGTTGAGGAGTGCGACGGGAAGCATTCCTGCCGGCATCATGGTAGCGCTTGTCCCGAGATTCTTTCCCGGCAAGCAGCGCACTCCGCCCGGATTTCTCAGCTGCGTGAGCAGAAAAGACGTCGCCAAGCAGAAGAGGAGTTGTTTTGGCAGCAGGTGCGCGCGGACATGGCTGAAGCGCGCAATGCTTTCGAGGCTTCCCGAGATGCATTGTGGGAGGAGATGGCTCACGCTCCGTTCTGGTATCGAGATGACTACGTTTGGTCCACCTTGGCTTTAGATCTGACGAACGTCGGCCTGGAGCCAGCTGATGTCCATTTCCTGTCGCGAAAAGAAAGGAATAAAATGCAGCATGCATTGAATGGCAATATTGATCCCGGCAAGTCGGCCCGCCGAGGGTTTGTGCCCAAAGTCACTGGTTGGGCCAAGGCCACGACTATCAGGTTTCGGCTGAGCTCTGCAAGCAGACGATGCTTTATAGGTAAGGCTGAGGCCCCTATCGAGCTTGCCCCCGCACCTTTAGTTGGGGGTCCTGAACCCTCGCGTGACGTGCAGCAAACTGCACCTTCGCAGAATGCGGCTGTTGTACCTGCTTCGAATCCTGCGCCTGATGCGGTTTCTGCTGCCTCCACAGGTTCTTCGTCGTCGGTTTCTGCGTCTATGGTCTCTGTTATGTCAAAGGTTGAGCAGCGTCGCGAACCCCTGGCTTCGACTTCTGCGATGACACCCTTAGATCCCTTCACGGATGACCAGCGACGGTCGTATATTAATTATCGTAAGATAGCTAAGGAAAAATTGGTCACACCTTACACCTTTGTTCGACCACCGTGTTGTCCTCCTGATCCTAATTACCCGGGAAGAACCGAATTGGTCATTGGAGAAGGGGACGGCTATGTTGTGCTTATGAGTAGTTTCCACGACAAGCCGTTAGTCACAGTGTGGGCAAGCGAGCACGCTCAAGTGCCATTGATACCGTACGACATATCGTATGGTGCTCCGTGGTTGGCCACATACCCTGATCATTTTCACGGGCATATAGTCGCTATGACGGGCAACAATGTCAAAGGTGTCGCCTGCATTTTGCCAGCGAACACTACCTTTCCTGACTTCGCTGCCACTGCGGAGGCTGGGCTGGCTATTAACTACCGAGCCGCAAAATTCGGCATTTCTCGCAAGGTTGTCGTCGCAGTCTGCCCCAAGTGCAAGAAGCTTTTACCAATTCACCCTGATTTGACACAGTGTGAGCATTGCTGGGCCAAGTCTGGTCCAGAAGTTGGTCGTGTGATTCCTTCTTACATTTCGGCAATCCGGAGCCGTGTCCCTGAAGCGACAATTGCCCAGAAACTGACTATGGCGCCGAAGCGATTCCGTAAGAAGAGAGGGTTTGGAAAGCCTGCTCCAATCGCGTCGCCTGTTGCGGCGGTGTCGGACATTTTACAGTCCAAAACTGCCCCACTACAAGAGAAGGTTGCTGCGATACTGGACCTGATTCCTGATGTTGCGGATCACGCAGGTCAGACCTTTGCCCAGGTTGCCGAACCAATTGTCGACAGGGTTTACAATGGGGTGTTTGGCCCTACTCCTTCGAAAATTGTGTCCGAAATCACTAAAACTGATAAGAAAAATAGGGCGCCCGTAATGTCTGATCAGGAGCTTAAGGCATTGGTGATGCTGGTAGGGGCCGAACAGGCCCTCAGGCAGATACCAACTGCCAAGGTCCAGACAGTTATGTTAGAAGAGTACCTTCGCGAATGTGTCGTTAATCCTAACGAACACACTATCTTTGTAGACGCAGTTATGGCTCTGAACCCGAAAAGACGGCGTACTGCCATCGAACTTGCGCGTTTTCCCTTGGCGGTTAGAGAACAGCTAACCGAAGAGTTGTTTAAACTCGCCAGATCATCTGCAGACGCTGACATTGAGGTCAACCCTGGTCCGTCTTTTTCCTTTAGGAAGTTGGCGGGGTTTGCAGGCGAACATGTGGTGCTGCCTATAGGCGGAGGCCACGACCTTAGGATGAAAGTCGTGCCTCGGTCGTCGGAAGCGGCCTGGGTGCCATTCGGCCCAGCACAGGGTCTCCATGATGTCATGCCGCCCAGGTATATCCCTGGGAGCGCTATGCGCACTCCAGAGTTCTTAAAGGCATTCAAAAAGATGACCACAAAGTGTAATGAGCCTGGCTGCTCCAAGCGAAAGTTCGCCACAACAGATATCGGCAATCTTGCCTCTGAGCTTGGTGGACTTAGCAACCTTCTTAATGTGCTGTTCTTCTGCTCGTATGATGTTTGCTCCCATATGCCTGGCAAAAACTGGCTGTGGCGAAACATCGAGCAGAAAGCAGATCCCGAGACATTTTTCGATGGATGGGCAGAAGTGTTTGACCGATGGGAGGGCGAGGGATTGTGCAAAGTTCGCGCCCATGATTCAGAGGCTGTGATGACTGCCTTGCGTGAAATGCCTCGCTTCTCTGAAATGCCGCTAACAGATATGGACCACGAGTTGGCTGAAAAGATGGGGTACGGCCATGTCAAGTGCCCTGTCTGCTGGCCGAACGAACCTTATTGGTGTCCGCAGTATTCCAAGATGATACACCCCACGCAGCTGGATGAGCACAAGCACATGTTGGAGTTTCTGCAGCTGGGCCTTGATTTCTGGAGGGATGTGCCGGATGACTTACCCGTCCTGGTGGTTACTGATCTTGGCGAGCTCCAGTCCAATCCTGCATCGCTCGATGGCTTTAGAGCTATACACATCACTACCGACAAGGACAGGCGTGGCACGCCTGGCTACTATTTCGTTACAGCCCTTATGCGTCAATATATGACACCCATGATACAGGCTGCGGCCGTTCTTTTGGAAAGACGCAAACCAGTTTTATACTGCAATGAACTGCAAATGCAGCGCATACCTAGAACTGTGTTGGCGTTGCCTCCAGGGGCCTATGGTTTGTTTGGTGTAGATGGCCAGAGAGCTATTGGTGCCTTCACCTGGCCCGAATACGACTACAATTGGGAGAATGACGGGCTGGCGCTCATCTGCCGTGCCTTTGGCAACTACGAGATGTCACATCTGCTCGAGCGTAGCCGCATAGAAGGCAACTTCGTGTTCTGTGACAACGTCGAGGTTTTCAATGCCTGGAAGGTGAAGGCTGAGATGGCTGTGCCACCAGTTGGGTTGACAAATAGGACGAACTATAGGCTGTTGTCGCGACCCAACTTTGTCATCCGATCCGCAGGGCTGGGTCATGCCCTGGGCCGATTGGCTTATGTGTCCCTTTCTGGAGCCACTTTGTGGTCGGGGTTATCCCCGATGACCATGGCCTCAATGGTGTCTGGTTCGTCCTTCAACACAAGCCATGTAGGGGCCGCATTAGAAATGACTTTGGATGAAGTCCTGGTATGGACTCCTCCGCCACGAGATGCTTGCCCATGCACAGGATGTCTCCTGGCTAGTTATTACGGGCCACTTGTGTTTTGTACGGCAGGTTCGGCTGGAGACATTGTCCCTGTGCGTGCTATAGCAAAGGCGCTTTCTCGTCGAGGCTTTGACACCGTAGTCGTTGACTGTCTTGATGGGAAGGGGTACGACTTCCTTCGTGCAGTGCAAAGAGGTGAGGTGCTGTCCCTTGGGCCCTTATATGCCTCTGCCGCTGTCGCCTGCAGAAATTTGCCCTTTATAACTATAGGACCTCCAGAGTTGGCGTGCTCTATCACCTGGAGCCTGGCACCCCCTCCGTCTGCTATACGGCAATTCAATTTCCGCCTTGGCCCGCTTTTTAATCCTTTATTGTCATGGTTTGCCGGCGTGACTCAACCGGTTGTACGTATTAGCTCGTACCCTGGGGCAGCGCATTTGCCACGCTCCGCCGATGGAGAAACGTTCTTGAAACGCACGTCACCGGATGACAAACCTCGCAAGCACAAATACTTGGTGGCCTTCGGTTCGTCGGGATATCCAACGCCCTCTGGGATACCCGTTGTTCAGCCAGGCGATCATTATAGTCAATTCGTCGAGGCTGAAACTGTCATTACCCATGGAGGGGCTGGAACCGTCCAAACGGCCGCTATGGCCGGGTGCAGAGTTATCTCCGTTGACAATACACTTGACCGTGACTATCTAGACCCCACCAATTGTCAGTCTGGTATTGGGGCTGAGCACCATGAGGATAAGGTGTTAGGGCTGTTGCTGGGCATTGACTTCTTGCCATTCTTTTTGGCCGGAAAATTGCCTGGCTTCGGGCTACCTTCTATGATACATTGGTATGTGTCCGCATATCTTCTGTCCGACATTATGACTATATGGGCACTTTACCGCATTTGTTCCATATCTTTGGTAGTAGTGCCTGGTGATTGGGTTTCCACCGTCATTGCTTCCCTTGTCGGGTCGGTTTTGCCAGGCGGCTTTGCCGCACTGGCTGCCATAGTCATCAACGTTTGGGGTGTTTCCATATTTGCTAGGGCGGGCGTCACGGGCCCCGGTCTAGTAATGAAACTTCTACGTTTCGGACGTATGGTGCCACAGCGGCCTTGGCTATGGATTACTTTCTTGGTTGGTGGCGTCAATGCAACTTTGTTCGTTGCATTTATAATGTTGCTACTTGTCCCAGCTGCCGGTGTGACGCGTCGCGCGTACGACCATCCCAAGTCGTATTTGTGTTTTTCACTGGTCGTTGGGCTCCCTTTCCATTGCTTCCTGATGAGCGTCGACGGGACTTCTGTATTAGAAGGATCAGTTTTGGTCGATTCTGAGAAGGCTCCTTATCGGTTGACAAGGGTTGACAATGTCAAGCCTGACTACTTATTTAAGGTGCCTACCTTGGTCGATTTTGAGTCCATAACCGATACTGACCAGCCCTCTGCAAGCTATGGGTTCTTGACCCACAACTGCAACACTGTATTATACAATGCGATTTCCAATAAAGGTGGTTTTGGCCTGGGCGGGTCAATTCTCTACATGTTTGTTGGTATCGCCTCTATTATCGCGGTATTTGGTATTGGCTTTGCCCTTTTCGTTGGCCTTTCGTCACTGATGTTAGTTGCGACTTCTGCTATTACGCCAGCAACGTTCCAAAGCACAGTCAATGAGATCATATTCAAATGGCGACGCGCAGCACTGATCGACCGTATTCGAGCCGACCGGACTGTGCTTTCTTCGCTACTTGTTATCGCACTCTTGGTTACAGATTCTCAGAGTGGCTTGACGGCAGAGGAACGCCTGGCATACGAGGCGGTAACATCATTTAGGCAAGATGTGGCCAAGATGCCTGAACCTCCTTCCTTGGCCGAAACCTCTGCTCTTATTAATAAACACATTGTCCTTGGGCCATTGTTCAGGGGCAAATGGCGTACTTTGGGCATTATGTTTGGTATGATTCCACATGATTTTTCGGATGCTGAGATGGCGGCCATTACGGGCGTGCACCCCTTGATTAGGGAAGCACTAGAAATACATTCTCGTTATGTTGTTGCACTCCAGGCGTCAAAAACCCACAAGATAACCTTTCGTCAATTTGTGGAATTGCGTGAAGGCCTGCTAAATGAGGACTTCGAAATCTACAAAACCATAGGGATTCCCATTTTCGATTCTTTTTATGAAATCAATGATTTACAGACGAGAATGGAGTCTCTGACGGAACCTTATGATGGGACTCCCAACGAGGTGACTGCGATTATCGACGGGATATTGCCCAATTTCTTACTTGAGAATGAGCTTCTCACAGCCTATGAGGCTTACCCAGTCCATGGTGATGGACCTGCGTCAAGTGAGGACTTTGCACGTCTGGGCCTTGATCTTGACGAGGACGAAAAAGCTGTGTACTCTTTCATCTTTTACTCTGACTTGCATTGCCCTGAGACCGATGGGTCTTCGGTCTTCACTACAGTGCGAGAAGAACCTTTGTCGCAGTCCGACATGGACGCTTTGTCCATTCTTGTCGCTGCCGCGCGATCTTACGGGGCAACGGAAGTTGAGGCCTTCACGGAAGCAGTCCTGAATGCCAAGGACATCTTGCATCCCAGGGGCCGGGCTGTTTTAGTCGGGGCAGATGCACGCATCAATCAGGCGATCGAGGGCATGGTGTCTGCCACACGCCCGATTCGCGCATCATTACAATGGATAGGCCTTTCCGTGTTTAAAGCCCGACATTTTCTCCATTCCGATCCCGTAGCTTCGGCTCTGCTGAAACCTTTCGACATCTTTTTCACGGTGATTGGGTTTTTGTGCGCCACGTTGCTTTCTGTCACTTTACGTGCATTTAACGCCGTACTCGGAGCAGCTGCTTCAACTACTCTTGTTGAACAAACAACAGTTAAAGAAGTGTTAACCGCTGCAGGTGTCTTTTTATCGACCCTGGATCCCAGGCATAGATTCCGGCCCAAGAGTGCATGGGCGCTATTGTGGGAGAGGTCGAGAACAATACTTTCGAAAGGCGAAAGCTTGTTATTTTCTCTTCGTAACTTTACTTATGAGCACAGCCCCTCTTACCACGATTGGAGCGGCAAGATGATTGAGTTACTATCTATAAAGGGACTAGATGCCTCTGTCTTGCATAGGTCTCCGCCTCGCCGGGCTGTCTTCTTTCCATCCCGTCCGGTTGGTCTCAAGGAGTACGAGGGGCTGGTGGCTGATGCCGACCTGCATTTCATTGAGATGGCAAATGCCAGACGCAATATGGAGCAGAGCATTGCCGCTGGGAACGGCTTAGCGATTGACGGTGCATGGATGGCCAAGCCCGAACATGTCACGTCTTCTCTCTTGAGATATACCGTGCCACGCCCTTCGATCGACTCCGACGCTAGAGCGATGTTGTCTCTTGCTGCTGATGCGATATTTGAGCATCATCCCCAGTTATATGTGAACCCAATGCCAATGACCGTGTCCCAGGTGCTGCTTAAAAGCAAGTGGAAGTATTCGGCAGGCTTGCCGTTCTTGCCGGTTATTAAGAAAAGGGAAACCTTGAGACACTCCAAATGGTTTGAGGCGATCCAGGCAGCCGCTGAACGTATTATTGACGCAGGAGTCATGCCCTCAGTTGGCTTGCATGCCTTTCCTAAAGCCCAAGTGGTTTCCATGCAGAAACTACTCGATAACCCAGCTGCTGTCCGTACCGTCACTGCTGGCGATCGTATAACTGGCACTGTTTTCAATACATTGTTGCTGGAACGCAACAAGCGTGTCCCTCCAGCTCATTACGGGCATGTCAATATGCTGCGGCGCTCTGAAGGGGGAGTTGCATTTTTAGAAGACCACCTTTCTGAATTCCCTTATTTCTATACGGGCGATGGTCGGGCTTTCGACTCGACTGCAGCGTCCGAAGTCTCCACCGTCGGGTCTGTTCGCTTATATGAGCTAGGTCTCAAGGCAGGTTTTGTGTTCAATGAGAAGGCCGCGGTCTCACTTGTCAAGTCTTACTACGAGGGCTTGACACGCGGCTTAATTATCAATCTTCTCAATGGCGACGAAATAATTAAGACTGGGGGAGGTGGCACCGGTTCGGTGGCAACAACGCCGGACAATCGCGACTGGGTGGAATTGGTCTTCCTGGCTGCTTGGGGCCTTGTCACCAATTCACATCCATCTACGTTTTATAAGCACTTCCGACTTGCGCATGCTTCGGATGACGTGCTGTTTTCTACAGACGAGTATGGTCGAGAGCACCTACAGGAATGGGTTGACTGTATTCGGACTAAATATGGCCCGGATTTTTCTTTCGTTCTAGAAGATGGGGTGGACAATCTCATACACCTAAAACGTGTGCCGATATCCACACTAGATGAAGCACTTTATACGAGACTGAATTTGCCGGTGCCATCTGTTGGATTTCGACATGATCCTCAGAGGCTTTTGTTTTCTCGATCTGCCTACAGGTCTGACCGCGCGAAAGCCAACGCCGAAGTGATGGCCGCCCATGTCGCTGAGCGCAGCGCGGGGTTTCAACTATTGACCGCACATGCGCCGCAAGAATATGACCTCGTTACACGCGACCTTGTCGACGCCAATCTTGAATACGCGCTTTTATTCTTCCGCGGAGCCCGTGCCTCCTTGGAGCTGGATGGCTCCGGTAATGTTATCGACGGTATTGTCGAGATCACTGATAGACGACCTGCCAGACACGTTAAACGCCTGGCCCGCAAGTTTGGCGTTGGAAGCCGCGCCAGCGACTGGATACTAGCCCGTCAAGTGGCGGCCGAACGCGCCTTGAAGCAACGTCGGGGTGCAAGCTACCTGAAGGTCTTTGAAACATGGGTTAAGCCACCACCACCAACCAATGAGTCGAAAGCTTATCGACGTTGGTTGTCGTATCGTGGCGCGGCCAATAGTCTCAACCCTATTATTGATGTGATGAGACTGACGGCAAATAAGCTCGAATTTGCCATCTCTTTAGTGCCATCGTCACTCATTAAGAGTGCTGCGGAACCCATCGCTTCGCGGTTTTCTAAACCGTTTACGACCTTCGATTTTGTCATCGAACAATACATCTACAGAAGGGAATTTAAACGTACAGGCCAGCCGGTCTCGTTTGAACGGGCTGGAACGCTAGTCCGCGAGTCCCCATATGCCGCAGCAACTGATTTAGCAGCTTTTTATGCGGTGTTGTCGTACCCTACCGCACTTGCTGCGTTCCATAAGTCTATTCGTGAAAATCGCCCTGTCTACGATCCGGTGTTTTTCCCGAGCAATGACACTAATGCAGCTCAAGTGCTTGTTTACATGTGTTGGTACACGATGATTGACATAGCTATAAACGCCACTAAGGCTTTCCCATTGCTTGGGACAGTGGTTCTGTTCACTTTTGCAGCCTATCGGTGGCTTGACATCTTTTATTCGGTTCAAAGCCTTTTGTTCTGGTTGGCAACTGCTTCAGCATCTCTGCCAATATCCAACTCATCTCCTAAGGACAAATATGCCCTGCAGAAAGTGTTGGCGGCGATCCTGGCTGCGGCCACACCGTCAGCGTTAACATGGTATGTCCCCGGGGTCTTCTCGGCTTTGGGTAAGCTCGGGCCTGCAATTGAGCTACAAGTCTGGATACCAAACTTATTCCAGAGCGTGCACCAAAGCGCTATGCAGGAGTTACGCGATGTCCCGGCTGAGGCTTTCGCTTCTTTCCACACTGCATGGAGTCGCGACACACCCGTGGTGCTGCTAAATGCGCCCACAGGCACAGGTAAAAGCACGAGCTTGCCAGCAAGCATATGTGCCAGGTACCGCGGCGTTTCCGTTTTTCTACTGTTGCCCTTCAACAGGTTGGTTGAAGACTACCAAAATAACTTTTCGCTGAGGCTCAATATAGTCAAGGTCCAACGAGACACGCAACGAGATGAGACCATCGCGGGAGATATACTCGTGATGACACCCGGGCAATTCTCCCGCCGGGTCCTCCAATTTAACGTGTCGAATGCAGTTGTTCTAATCGACGAGGCCCACTTGCCTTCGAGTGAGGCTTTAGTGGCATTCAATACCTGTTCTCAGCTGAACATACCGACCATACTGATGACAGGCACCCCTGGGCATTTCTTGTCCGGGGGCCTTTTTGGCAGTTTTCCCACCTTTGATGCCCCTCCCCGACTGTCGTATGCTAGAGACATATTGCCCGAAGCCGCGACCTTCGAAGAGGCTGTTCATCTTTTGACGAGGACGAACACCCCACTTTGGGATGTGCTTGTGTTCGATCCGAGCTTGCAGACGTTGGAAGTGTGGTTGACACAGTTCCAAGGCCTGAAAGTCCCGGTTAAGCGAATTACCGCTCATATTGGCGACACAGGAGCTCGTGCAGCTGCGTTTGCTACGGGCGTTATTGCTGTAGGTGCGAATGTGACACCTTCACCCGGTGCGCTTATTGCTAGCCCGGAGCACCTAGTCGCTGTGCCTTCCAGGAAGTTAGCACAAGATGCACAAGGAAGGTATCTTGTTGATCAGGGTAACCTTCTACATGCTGTGGTTCTTTCTCGCCATGCTATGCCTGCGCACGAACGCCACCAACTCTATTCACGTGTCGGTAGGACACGTGATGGGTTGGTGATACCCCTAAAGACAACTCTTGATGAAGAGTTCTCATCGTATGTCACTTTAGGGGGCCTTCTTAAGGCGGGACAACCAGGTGTCCAATGGTTAAAATACTGCGGTGTACGCGCTTTGTCGTTGGATCCGTCTGCAGCATGCAGGCTTTTTATCGTCAAAGAATCTTCTTTTCTTGACAGTAATCAGGCAGCTGTGGCATATGGTTTTGCCGCTGCTGCTGTCTCTGTATCTTCTTTTTCTTCTTTATCGACATTATACACCTCTTCCGACAGGGAAGAGTCGGATATGCAACTCTTAGACGGCCTGGTTGCAGATTTTGGTGTGAATGCCGAAGCATACTCTGTACATTGGCGGGTTGCCCTTGCAATGTTTTGTATGCATTATGCGCTTGATATCAACGGTGTAGTGTATAATAACACCATACCTATGATACTTGCGTCTAAACTGGCCCTATTTAAGGTGTCAGAACTCTTGTGATTTGTCAGCCAGGGAGGAACGATCCCCCTCTATGTGAT

> SrHV4

GCGTTTTGGCGTGAAATCTCTTATAAACCATGTTGTTTTCCATCTTTCCTGCTAACGGTGTTACCGTCACCATGTCTCGACTTGGTGCGTTGTGCATCAAGAATAAAACTGGGGAGATGAAGACTGTTGGAGAACAGTCTGTGCCTGGGTTTGCCTATGTGGCGCCTTTGGCAATTTCGTTTAGGCGTGCCAGCTCTTTTTCAGAAGAGTGCAATGGCAAGGTGCCGTGTAAGCATCATGGCCGCGCATGTCCTGAGTTGGCGCGCACTAGGGCCTTGAAGCGCGACTCTTTGAGAGCGCGCGAGGCAGCTAGGGAAGCCCGTAAGCGTGCAGAGGCTGAGAGGTGGGCTAGCATACGCCAGTCCATGCAGCAAGTGCGCCTAGAGTTTGAGGCCAGGCGTGACGCTCTTTGGGAGGGATTCATTCACGCTCCCTTTTGGTGGCGCGATGAGTTTGTGTGGTCTACTTTGGCCTCTGACTTGGTTGAGGCCGGCGTGTCGGTGCGTGAGTGCCCTTTCCTATCCCATAAGGAGAGAAATAAGCTCCAGCACATGTTAAACGGCAACATCGAGCCAGGCCCGAGTGCACGTCGCAATTTTGTGCCTAAGATTAGGAATGCCTGGCAGACATCACTAACTAAGGCTTGGACCTCTTGTGGGTCGCGGCGTGCATTCTCCGCTAAGAGAACGAGCACCGAGACGACTGCGACTCCGGTTGTCCAGCCCCTTGCTCCCGAGGGTCCTATGCCTAGGCCTGAGGCGGTTGATGCTGTGCCGCCCCTAGTGGGTGGTCCTCAGCCAGCAGCCGCCGCTCCTTCTGTGTCTTCCTCCTCTTCGTCCATTTCTGCAGCCATGCAGAGTGTTATGTCCAAAGTAGAGTCCAAGTATACCACAGTGGAGGAACTGCCCGGCCCTTCATTGATCGATCCGTTTTCTGACGATCAGCGACGGGCCTATATTAACTATCGCAAGATCTCGAAGGAGAAGCTTGTGACGCCTTATACCTTCGTTCGGCCTTCTTGCTGTCCCCCTGACCCGAACTATCCCGGCAGGACGGAATGGGTGATTGGGGAAGGCGTCGGCTATGTCGTTTTGATGTCGAGCTTTAAGGAGAAGCCCTTGGTCACCGTGTGGGCCGATGAGCACGGCATTGTGCCTCTGATCCCGTACGATATTTCTTATGGGGCTCCGTGGCTGGCGACCTATCCTGACCATTTCCATGGTCATATTGTTTCCATGACCGGCAACAATGTGAAGGGTGTTGCTTGCATTCTGCCGGCCAACACTCCTTTTCCTGACTTCCGTGAGGCTGCAGAGGCTGGTCTTGCCCTCAACTATCGTGCAGCCAAGTTCAAGGTTCCACGAAAGGTTGTTGTGGCCGTTTGCCCGCGTTGCCGCAAGCTATTGCCGATCCACCCTGACTTGAGTAAGTGTGAGCATTGCTGGGCAGCTGAAGTCCCTGAGCGTAATCGTAACATTCCTCAGTACATTCGAGCGATCAGGGATCGGGTGCCCGAGGCCAGCCTCGCCCAAAAACTCGCTATGCAGCCGTCCAAATTCAAGAAACGCAAGCATATGGTGCGTGCTGCTCCGAGTGTGTCGCCGGTCTCTGAGATCACGAATGTTCTCGAGTCGAGGGACACGCCACTGCAGGAGAAGGTCGCTGCTGTTCTCGACCTCGTTCCCGACGTGGCCGACCACGCAGGGAAAACCTTCGCCGATATAGCTGAGCCTTTTGTAGACCGAGTCTACGCGGGAGTCTTCGGACCGACCCCGTCTAAGATAGTGTCAACTATAACCAAACCTTCGCAGAAGAAGCGGCATCCGGTCATGACTGACGAGGAGTTGAAGTCATTGGTCATGCTAGTTGGTGCAAAGCAAGCGTTGCAGTCAATTCCGACCGCACAAGTCCAAAACGACTTGCTAGACTCTTACCTTCGCCAATGTGTCGTCAACCATGACGACAACACCTTGTTCGTTGACGCTTTGCTCGCCCTCAATCCACGCCGGCGCGAGCACGCGATCAAGTTTGCGCGCTTTCCAAATGACATTAGGGCTCAACTTACCGAGGAGCTGTTCAAGCTGGCCAGGTCCTCGGCAGATGCTGATGTTGAGCTGAACCCGGGCCCGTCTTTCTCATTCAAGAAGTTGGTGGAGTTTGCTGGCGAACATGTTTCTGTGCCATTTGGGTCGGGACATGACCTGCGCTTGAAGGTTGTCCCTAGGTCCGCAGATTCCCACTGGGTCCCCTTTGGTCCGTCGGTGTCCCTTCACGAAGTGATGCCTCCTAGGTATTGCCCGCCCAGCCCGCTTAGAACTGAAGCTTTCCTTAAGGCTTTTAAGCGACACACCTCGAAATGCCCTGAGCTGGGGTGTGGGTTGCGACAGTTTAGCTCTCCTAACGTGGAGTCCTTTATTGGAGAACTCGGCGGCAACGAAAACATAGTTAATCTTGCATTTTACTGTGCTACTACCATGTGTGCGCACCGCTATGGCAGGAGTTGGTTCTGGAATTATATTTCCTCAACCTTTGACGAGCTACCGCTCAATGAGGCCATTGCGGATATATTTATCACATGGCAGGGGCGTGGGAAGTGCCGCGTGCGCGAGCACGACTCTGACACTGTTATGGCTGCACTGCGGGACCTTCCCCGCTTTTCTGAGATGCCGTTGACTGATATGGACCTTGAATTGGCTCATAAGATGGGCTTTGGCCATGTCAAGTGCCCGATATGCTTCAGATCTGAGCCGCACTGGTCGTCTCTCTACACAGAGATGGCTGGCCCCTGCGAGGTAGAAGATATCGACAAGATGACTGCGCTGGTTCGCCTTGGCATCAACTTTTGGAGAGGCGTGCCGGACGACTTGCCCCTGATTGTCTGCGAGGACATTGGTGAGCTGCAGTCGGTGCCTGACGGGCTGTCGCCTTTCCGTATTATACACGTGACCAGTGACGTGGATAGGAAAGGCGAGCCAGGCTACTACTATTGCTCGGCTAGTGCCCGTCCCTTTATGAACATTATGATTAGGCCTGCTGCAACGCTTCTTGGGCGCAAGCAGCCGGTCCTTTATTGTAATGAGTTGCAGATGCAGCGTATTCCCCCAACAGTGCTGGCGTTGCCCCCTAGTGCGTATGGCTTGTTTGGTGTCGATGGCCAGTCTGCCATAGCGACTTTTGCATGGCCGCAACATGATTTTGTGTGGGAGAACGACGGGCTGTGTTTGATGACGAGAGCAGTAGGTTGCTTTCCTTTAACCCGTGATCTTATGAGGTCGCGCATTGAAGGCAACGTTGTCTTTTGTGACAACGTCGAGGTCTTCAATGCATGGAAGGTCAAGTCTGAAATGGCATTGCCACCCGCTGCCCTCTACACCAAGAACAATTTTCGCCTGCTGTCTCGTCCACATTATGTGGTCCGCACGGCCGGTCTCGGTTCTGCGTTGGGTAGGTTGGCCTATGTTTCTTTGTCTGGGTCTTCGCTATGGACTGCGCTGTCGGCAGGAACACTAGCCTCACTCTTTGCTGGGTCCGTCTTCAATATGAACCACCAAGGTGTTTTTCATGAGATGACGCTGGACCAGGTGCTTATATGGACGCCACCTGCCCGAGATGCGTGTCCCTGCACAGGGTGTTTGTTGGCGGCTTACTATGGCCCTATTGTGTTCTGCACGGCAGGGTCTGCCGGCGACATTGTGCCTGTGAGGGCGGTCGCTCGTGCCCTAGCCCGGAAAGGGTTGGACACCATAGTAGTGGACTGCCTTGACGGGAAAGGTTACGATTTCCTGCTAGCAGTACAACGTGGGGACGTGTTGAAGTTAGGCCCCTTCTACGCCTCTGCTGCTGTTGCCTGCCGCAACCTTCCTTTCCTAACGGTCGGCCCTCCTGAGTTGGCGTGTGCAATAACTTGGAGTCTTGCGCCGCCACCTACGGCTATTAGGCAATTCAACTTCAGGCTTGGGCCCCTGTTCAATCCTTTGCTTTCATGGTTTGCTGGTGTGACGCAGCCAGCTATTCGCATCTCTTCTTACCCCGGGGCTGCGCATCTTCCTCGTTCGGCTGATGGTGAGACGTTCTTGAAGCGAACTCTCCCCACCGATAAGCCTCGCAAGCATAAGTACTTAGTTGCGTTTGGGTCTTCGGGGTACCCAGTGCCACCTGGGTTACCCGAGGTTCAGCCAGGCGATCATTATAGTCAGTTCCTTGAGGCAGACACTGTTATCTGCCATGGAGGGGCTGGCACTGTGCAGACGGCTGCAATGGCTGGGTGCCGTGTCGTTTCAGTCGACAACACGCTGGACAGGGACTATCTTGACCCCACGAACTGTCAGTCGGGCATAGGGGCGGAACACCATGAGGACAAGGTGATCGGACTCCTATTGGGGCGAGACTTCATGCCGTTTTTCATCGCCGGTCAGCTGCCCGGGTTCGGGCTTCCGAAGATGCTTCACTGGTATGCGTCTGTCTACCTTCTAGCAGATATTATGACTTTGTGGGCTTTGTTTCGCGTGTGCAGTGCCACCTTGCTTGTGGTACCCGGTGACTGGGTGTCCACGCTGATTGCTTCACTGGTGGGAGCGGTCTTTCCGGGGGGTGTCGCCGCACTAGCGGCCATTGTCATCAATGTGTGGGGTGTCTCTATATTTATGAGAGCAGGCATTACTGGTCCTGGCATCGTCATGAAGATACTCCGCTTTGGGAGACAGGTCCCTTCAAGGCCATGGCTTTGGATCACTTACTTGGTGGGAGGGTTTTACCCCACCCTTTTAGTGGCCTTTATCATGTTACTGCTTGTGCCCGCTGCGGGTGTCACTCGCCGTGCGTATGACAACCCTCGGTCTTACCTTTGCTTTACCTTGGTTATGGGCCTCCCATTCCACTGTTTCTTGATGAACGTTGAGGGCACGGAGGTCCTGGAGGGATCAGTGCTTGTCGACACAGAGATTGCACCGTATAGGTTAACCAAGACTAAGGTTACAGGGCCCGTCAAGTATGTTTTCCGGGTGCCAACTCTAATAGAGTTTGACGCCATTCGCGACTATGATCAGCCGTCGGCATCATATGGTTTCTTAACACATAATTGCAATACTGTCCTGTACAATGCAGTTTCTAATAAGAGTGGTTTTGGGTTGGGGGCGTCGATCCTCTACCTTTTTGTCGGCATTGCTTCGGTAATTGCCGTTTTTGGTATTGGTTTCGCCTTATTCGTTGGGCTTTCTAGCCTTATATTAGTCGCCACGTCCGCCATCACACCGTCTGTGTTCCAAAACACAATCAATGAGCTGATTTTTAAGTGGAGACGTGCCGCATTAATTGATCGGATTCGTGCCGACCGAACGGTACTTTCATCGCTCTTGATCATAGCGTTGCTCCTAACAGATTCCCAATCAGGGTTGAGTCAAGAGGAGAAAGAGGCTTTCGCAGCAGTGACTCAGTTCCGCCAGGATGTCGCGGCGATGAATGATCCGCCTACGCTAGCTGAGACAAGCGCCTTGATCAACAAGCACATAGTTCTTGGCCCGCTTTACCGTGGCAAGTGGCGCACTTTAGGGATTATGTTCGGCATGATCCCGCATGACTTTACCGACGCGGAGACTGCCGCAATCATGGGCGTACACCCGCTTGTCAGGCGCGCCCTTGAGATCCAGGCTCGTTACCATTCTGCGCTTAATGAGGCAAAAACGCACAAGATAACGTTTAGGCAATTCGTTGAGCTGCGGGAAGGCCTTTTGGATGAGGATCCCGATGTCTATCGCAACCTTGGAGTGCCCATCTTCTCCTCTTTTGCAGAAATAAATGATTTGCAAGTAGATTGTGATAGCCTGTCCGAGCCCTATGACGGGGAAGGCAACCCTGTCGAATCAGTTATTCAGGCTATTTTGCCTAACTTCCTGCTAGAAGAGGAGTTGTTGAAGGCTTATGAAGCCTTTCCCGTGCATGGGGATGGACCAGCTTCTCCAGATCAGTTTGCCTCCCTCGGGCTTGATTTCGATGAGGACGAGAAAACCGTCTATTCATTCATATTCTTTTCTGATCTTCATTCTCCAGAGCTTGACGGCTCTGCTGTGTTTTCTACCGTTAAGGAGCAGCCACTGACACAGCATGACATTGATTGTCTGTCTGTACTCACTGCGGCCGCTCGCTCTTATGGAGCCACAGAAGTGGAGGCCTTGAAAGAGGCAGTCCTTAATGCCAAGGACATACTTCATCCCAGGGGTACTGCAGTCCTGATCGGAGCTGATGCTCGCATCAATAATGCTATTGACGGGTTTGTGTCGTCCACAGCGGCAATACGGTCGAGTCTCCAATGGATCGGCACTGCAGTATTCAAAGCACGCCACTTTCTTCACTCGGATCCAGTCGCCAGTGCACTGATCAAACCTCTTGATGTCGTGTTTACCTTTGTTGGCTTTCTGGCCGCTACTATGCTTGCTATTGCACTGCGCCTGGTTAATGCCGTTTTGGCTACTCTGGCCAAGATGGGTGCACTTGACCACACCACGACCAAAGAGGTGTTAACAGCCCTTGGGGTTTTCTTATCCTCTATGGATCCTCGTCACCGGTTCAAGCCCAAGAGCGCATGGGCTTTGCTCTGGGAAAGATCGAGGTTGGTATTATCCAAAGGGGAAGCATTATTGTTTTCGCTGAAGGACTTCACCTACGAACACTCGCCTTCTTACCATGATTGGAGCAATAAAATGGTTGAGTTGCTCACTACAGAAAAGCTTGATGCGTCTGTACTCCATAGGTCCCCACCGAAGCGTGCGGTGTTCTTTCCCCAGCGTCCTGTTGGACTCAAGGAATACGAGGGCCTTGTTGCGGACGCCAGCCTGCATTTTGTTGAGATGGCTAATGCGCGGCGTAACATGGAGCAATCTCTTGCAGCAGGCAATGGCCTTGCGATCGATGGAGCATGGATGGCCAAGCCAGAACATGTCACCAGTTCTCTCTTGCGATACACTGTACCGCGCCCATCGTTGGACAGCGAAGCGAGAGCCATGTTAGGCTTTGCGGCAGACGCAATATTTGAGCATCATCCAGAGCTCTATACGAACCCAATGCCTATGGATGTGTCGCAGGTGCTGGCCAAGAGCAAATGGAAGTACTCCGCTGGGTTGCCATTTCTCCCAGTCGTGAAGAAACGCGAGACGCTGCGTTTCTCGGAGTGGTACAACTCGATACAAACGGCGGCTCAGCGCATCATTGATTCTGGTGTCATGCCCAGCGTTGGATTACACGCGTTCCCAAAGGCACAGGTTGTGTCCCTTGAGAAACTGCTTAACAACCCAGCGGCAGTGCGAACTGTCACAGCCGGCGATCGTATCACCGGCACAGTCTTCAACACTCTCTTGTTGGAACGCAACAAACGGGTCCCGCCTGCTTCATATGGGCATGTTAACATGCTCCGAAGAAGTGAAGGAGGCGTGGCCTTTTTAGAGCAGCATCTTTCCGAATTTCCTTACGTCTATACTGGAGACGGTAGGGCGTTCGACTCCACCGCTGCATCTGAAGTCTCGACAGTTGGCTCAGTTCGCCTCTATCAGCTTGGTCTTCAGGCAGGGTTCGCATTTAATGAAAAAGCTGCTGTATCCCTAGTTAAATCTTACTACGAGGGATTGACAAGAGGATTGATTGTCAACCTCCTTAACGGAGACGAGATCGTGAAGACCGGAGGTGGTGGCACTGGATCAGTAGCTACAACCCCTGACAACCGTGACTGGGTTGAACTAGTGTTTTTGGCGGCCTGGGGCCTTGTTACTGGGCAACATCCCAACACCTTTTACCAGCACTTCAGACTGGCACACGCGTCGGATGATGTTATCTTTTCAACCGACGAGTTTGGTAAGGAGAAGCTTCAGGAATGGGTTGACTGCATCAGGACAAGATATGGGCCTGACTTCACGTTTGTGCTTGAAGAGGCCGTTGACAATTTGATACACCTGAAGCGAGTGCCAATAACCGAGAAGGATGCCGTGTTGTACCAGCGTCTTGGCTTGCCTGTCCCCGAAATCGGGTTTCGGCATGACCCCCAAAGGCTGCTCTTTTCTAGATCTGCCTACAGATCGGACAGGGCCAAGGCAAACGCCGAGGTCATGGCTGCCCATGTTGCCGAGCGTAGTGCAGGGTTTCAACTGCTGACTGCTCACAGCCCACAAGAATACGATCTAGTCACGCGTGATCTCGTTGACGCCAATCTCGAGTATGCGCTGATGTTCTTCAAAGGCGCTCGCGCAAGCCTTGAGCTTGATCCCAATGGCAATGTGATCGACGGTCTAGTAGAGATAACAGACCGTCGTCCCGCCCGCCATGTCAAACGCCTTGCATCGCGCTTCGGCATCGGTGGCAATGCCCAGGCATGGATCGCGAACAGGCAGCGCATAGCCGAATCCCATCTGAGACAGCGTCGAGGGTCAAGCTATGCTAGGGTGTTTGAAACGTGGGTCAAACCGCCGCCAGCGCATAATGAGTCCAAGGCGTATAAGCGGTGGCTGTCTTATAGGGCCGCAGCCAACAGTCTAAATCCCATAATCGATGTATTGCGGCTAACAGCCAATCGTATAGATTTTGCTTTGAGCCTGGTGCCCACTTCACTGATCAAGAGCACAGCAGAGCCGATAGCTGCAAGATTCTCGAAGCCCTTCACCACTTTCGACTTTGTTATCGAGCAGTATATATATCGGAGAGAGTTTCTGCGCACTGGACAGCCAGTTTCTTTCGAGCGTGCGGGTGCCCTCATAAGAGAATCTCCCTACGCTGCTGCCACTGACTTGAGCGCGTTCTATTCGGTTTTATCTTATCCCACGGCTTTGAATGCGTTTCACACGTCCATTCGCGAGAACAGGGCAGTCTATGCTCCTGTCTTCTTTCCATCCAACGACACCAATATGGGCCAGGTTCTTGTATATATGGCCTGGTACACTGCTATTGATTTGGCCATTAATGCGACCAAGAAGCTTCCTCTCCTTGGGGCCGTCGTGTTGTTGACATTCTCAGCCTATCGGTGGCTGGATATCTTTTATTCGTTGCAGAGTCTGTTGTTTTGGCTGGCAACCGCATCTGCTTCCTTGCCTATCTCGAATTCTTCGCCAAAGGACAAGTATGCGCTGCAGAAAGTATTTGCCGCAATACTGACTGCAGGGACGCCGTCGGCCCTGACATGGTACATGCCTGGAATATTTAAGGCGATAGGGTCGTTAGGAGGTGTCATTGAACTGCAGGTCTGGATACCGAACTTGTTTCAAAGTGTGCACCAGACCGCTGTGCAGGAGTTACGTGACGTCCCTGCGGAAGCGTTTTCAGCTTTTCACTCTGTATGGAACAAAACAGTCCCGCGTATGCTCCTCAATGCGCCCACTGGTACTGGGAAAAGCACTAGCTTTCCAGCCAGTATTAAGTTGCGTTACCCGAACTGCACTGTCTTTCTCTTATTGCCCTTTAATCGTTTAGTCGAGGACTATTCCAACACCTTCTCTCTCAGATTGACTGTGGTTAAGGTTAATAGGGAGACTCAGCGTACCGAACCGATCGTGGGAGACATAATCGTGATGACTCCCGGGCAATTTACCAGGAGGCAAATGCAATTCGAGATATCCGATGCAGTTGTGCTGCTCGATGAGGCGCACATACCCAGCTCTGAAGCCCTTATGGCCTACTCGACTGCTCTGGCTCTTAAGATACCGATCATCTTGATGAGCGGCACTCCTGGGCACTTTCTTGCGGGTGGCTTGTTTGGCGACTTCCCGGCGTATGATGCCCCTCCTCGGGTGTCATATGCACGAGATATTCTGCCAGAGGCAGCCTCTTTTGAAGAGGCTGTCACCGCGTTGTCGCGTACAGGGACTCCGCTCCACGAAGTTTTGATCTTTGACCCTAGCGTCCAGACGCTTGATGCATGGTCTTCGCAATTCGAAGGGCTTCGTGTGCCGATCAAAAAGATAAATGCTGCTGTGGGCGATTCTGGGCGCCCTGCTGCCGCTTTCGCAACTGGCGTTATTGCGGTGGGTGCTAACGTGTCACCTGCCCCAACGGCTTTAGTGTGCTCTGTAGAGCATCTCGTTGCAGCTCCATCTAAGAAGCTGAAGGTGCTTGAGCCTGGCATTTACGAAGTGGAACAAGACACACTCGTGCATGCAGTCGTGTTGACGCGCAGACAGATGCCGTCACATGAAAAACATCAGCTGTTTTCCCGAGTTGGGCGCGTAAAGGACGGCTTCTGTATACCACTGAAGTCCGACCTGCCCGTGTCCAGCTCTACCTTTGTCACCCTTGCAGGCCTTTTGAACGCCGGGCCTGCAGGGCAAACTTGGCTGAAGTACTGTGGTGTCAGAGCATTGTCGCTGGACGCTAGCGCCAAGTGCAAGCTCTTTATTGAGAATAAAGACTCTTTTCTGACACCAGCGCAGGCTTTAATCGCGTACGGGTTCGCTGCCGCTGTAGTTAATATGACCTCATTTTCTTCACTGACACGCCTATATGGCACTGACGCTCACGAGGACACAGAAATGCAGATCATCGAAACCGTGGAGTCGACCTATGGGATATCTCCCACGGTCTTCGACGTACACTATCGTGTTGCGTTGGCCATGTTTTGCCTCCACTTTGTTCTCGATATCAATGGTAAGTTGTATCCAAATACCATACCCATGATACTTGAGTCAAAATTGGCACTTTTCAAAGTGTCATCTTTATTGTAAAATTTTGTCAGCCAGGGAGGAACGATCCCCCTCTATGTGATATGTGTGCTATGAGCAGTTACACACGCCCCCCACCTATCGGTGTTGCTCGCCGGTCCACGTTGTTTTTGGCTGTCTTCCCTTGATGGGAACTAGTGCTTATAGTCTATGCCGAGAGTTGGCACTAGGAAATGCTCGGACGTCGAAGTGTTTTATTTTACAATAAGCTCTGATGAATGGTTCATCTTGTTGTGTATGTGTGGAGCTACCATCGGCCTATGGGGCTAATTCGTAAACGAGAGTACGGTTAAAAATTTTCCGCTCGTCCAAGGTGCCTCCTCCACACTGCTGTGTAACAGCTTTCT

> SrHV5

TATTATAGCGTTTTGGCGTAAAATCTCTTGTTAACCATGTTGTTTTCTCTCACTCCCGCCGTTGGCATTGTCGTTGAGACTACCCGTTCTGGCGCTATTTGCGTCAGGACTAAGACAGGAGAGAAATGGACTGTTGGAGAACAGACCGTGCCCAACGTTTGTGACGTTGGAGTTTTTAAAATTGGTTTTATTAAGGCATCAGCCTATGTTGAAGAGTGTGATGGCGTGCATAGGTGCCGACACCATGGGTCCTCTTGCCCCGAGCTCTCAGCTGCAAGAAAGCGACGTGAGAGTGCCCTTGCTGAAATTCGTGCTCGGAAGGCTGCTCGAAAACAGGCAGAAGATGCCTATTGGGCGGAGGTTCGTGCCCTAAACCAAGAGCTTCGTAGGGCATTTGAGGAACGCCGCGAAGAGCTTTTCGAGGAGGTGAGAGATGCCCCCTTCGCGTGGCGCGAAGAGTACATTTGGGGCACTCTCGGCTGCGAGGCGATTGATGCGGGACTGGATTATAAGCGTGCTCCGTTTCTCTCCCATAAAGAGAGGAACAAAATGCAGCACGCCCTAAACGGCAATATTGAGCCTGGCGTTAGTTCGCGCAGGGGCTTCAAGGCAAAAATCGCCGATCATGCTGTGGTCTTAAAAGGGAGGTTTTCGTTGTCCTCTTCAAGCAGGAAGTTTTTCTTGATGAAGAAAAAGGACTCGACTGCTGCGACGGCACCTGTTACTGTTACTCCGCCGCCGTCAGCTGATCTTGGCCCATCAGTCGCCGTTGCGGCTGCAAACACTGTTCCAGCAGCTGCTCCTGGGCCTCGGTCGGCCAGCACTTCAAGCACGTCATCGATTGCCCAGTCGATGGTTACTGTTATGTCGCGTGTTGAGGAGAAGCATCAATCCGTTGCTGCGTCTACCCCGGCCGTCGTAAATGATCCTTTTGACGATGCACAGAAACGTGCGTACGTCAATTATCGTAAAGTTGCCAAGGAAAAGCTTGTGCAGCCCTACACATTCGTCAGGCCGCTGTGTTGCCCCGTTGACCCCAATTACATAGGGAGAACTGAATATGTGATTGGGCAAGGTGAGGGCTATGTCGTTCTGATGAGCTCCTTTAAAGATAAGCCTCTGGTCACAGTGTGGGCTGAGGCACATGGCGTTGTGCCCCTGATACCACACGATATTTCCTATGGGGCCCCTTGGCTGGCTACTTACCCCGATCATTTCCATGGGCACTTGATCAAGATGGTAGGTAATAATGTGAAAGGTGTGGCGGTGGTTTTACCTGCCAACACGCCTTTCCCAGACTTTGCAGCACAGGCTGAGGCCGGCTTAAAACTAAATTATCGTGCAGCTGTCTTTGGGCAAAAGCGAGCTGTGGTGGTCGCAGTTTGCCCCAAGTGTAAGAAGTTGCTGCCGATACACCCAGATTTGACGCAGTGTGAGCACTGTTGGGCACGTGCCGGCCCTGAGGTCGGCCGTTCAGTACCTGCTTATATAGAGAAGATACGCGACAGTGTCCCCGAGGCTTCTCTAGCGCAGAAATTGACTATGGCCCCCTCTAAGTTTAAGAAGCGCCGCCATCTCCGACGCCCTGGGCCTGTCGCATCTCCAGTGGTCGAGATTTCGAGTATACTTGAGTCAAGGACTGCCCCGTTGCAAGAAAAGGTGGCGGCGGTGCTCGATTTGATACCTGAAGTTTCTGACCATGTCGGGAAAAATTTTGCGCAGGTTGCCGAGCCCATAGTCGATAAGCTTTACGACAATGTGTTTGGCGCCACGCCTTCTAAGGTGGTCCAGGAAATTCTGAAACCCAAGAAGAAGGGCAGACTTCCTGTGCTTAATGACGAAGAGCTCAAAAGCCTCGTCATGTTAGTAGGAGCCAAAGAGGCCCTTACCCGCATCCCGACTGCAAAAGTGCAGAACGAGATGCTTGACGCTTATTTAAAGCAATGTGTTGTCAATCCTTCGGACCACACTCTCTTTATCGATGCGCTCTTGGCTTTGTCTCCGCATAGGAGAGAGCAGGCGATTAAGATGGCTCGTTTCCCTTCTAGTGTTAGGAAACAATTGACCGAGGAGCTTTTCAGGCTGGCACGATCGTCTGCTGATGCAGACATCGAGGTGAACCCCGGGCCGTCTTTCTCCATTAAGAAGTTAGTCGGCTTTGCAGGTGAGCACGTGTCCCTTCCGTTTGGATCGGGCCATGACATCCGCCTTAAGCTCGTTCCCAAGACCAAAGACGCCTCTTGGGTCCCTTTTGGGCCCTCTGTCAATCTCCACGAGGTGATGCCTCCGCGGTATTTGCCCCCCAGCCCACTAAGAACTCCGGAGTTTCTGAAGGCTTTCAAAAAGGCCACTACAGCGTGCACTGAAAGAGGGTGCACCAAGCGACGGTTTGCAAGCGCCGACCTCGATGAATACATCGATTCTCTCGGTGGCCACGACGGCATGATGAATTTGGTGTTTTATTGTGCCGACACTATGTGCGACCATCGCCTTGGCAAGTCTTGGCTGTGGTCGCTTATAGAGGAGTGGTTCGAAGCGGCGGATTTTGACTATTCTCTTGATGAGATGTACGCACTCTCTGACGGTGAAGCGCGCTGTCAAGTTGAAGAGCACGACAGTGGTGCAGTGATGTCGGCTTTGCGCGATCTGCCTCGCTTTTCAGAAATGCCTTTAACAGACATGGATGCTGAGCTGATCGACAGGATGGGCTTTGGCCAAACCCAATGCCCGCTCTGTTGGCCAGACAAGAAGTGTTGGGCCGATGTCTATGAACTGTTGGCCTCGCCCACTAATACTGACGACATCAGCCTAGCGCTGCCTTTCCTTCAGATCGGGATCGATTTTTGGAAGGACATCCCTGATGACTTGCCTGTGATTGTCTGCCTTGATCTCGGCGAGCTGCAGTCTGTACCCTCTGCGCTCGATAGTTTTCGCATTATACATGTGACGACTGATGAAGACAGGATAGATGAGCCCGGTTATTACGTCGTTGACGAGGAGAAACGTCCCTACCTGGCAACGATGGCGCGGGCAGCAGCGAACCTGCTCGGGCGCGTTCGTCCCGTTCTTTATTGTAATGAGCTGCAAATGCATCGGATTCCCCCTACGATCCTTGCACTTCCACCAGGTGCATATGGCTTATATGGCGTCGACGGGCAAAGTGCCATCGGCACCTTTGCTTGGCCGAGAGTCGATTACACATGGGAAAATGATGGGCTTATTCTGGCATGCCGTGGCGCCGGCAACTTTGCACTAGCGGGTCAGCTCAATCGTAGTCGTGTTGAAGGCAATTATGTCTTTTGCGACAATGTTGAGGTCTTCAATGCTTGGAAAGTCAAGCTTGAGATGATAGCCCCGCCTGTTGGGTTGTATACCCGAACGTGTATACGATTATTGTCCCGTCCTTCGTTTGTTGTTCGCGGGCCTGGACTTGGCGCTGCCATTGGTCGTTTGGCATACATTTCCTTGTCTGGGGCCAACCTGTGGTCCGGACTCTCAGCAGGAACGATGGCATCGATGTTTGCCGGGTCCGCCTTCAATCCTTCCCACGTCGGGACGTTTTATGAAATGTCCTTGGATGATGCACTGAGATTTAAGCCCAGTACCAGGGATGCTTGCCCGTGCACTGGGTGCCTCCTCGCTGCGTATTATGGCCCGATAGTGTTTTGCACTGCTGGTTCAGCAGGAGACATTGTTCCAGTGCGGGCTGTGGCCCGGGCACTGGTTCGAAAAGGCTTGGACACGATCGTCATCGACTGCCTCGACGGGCGCGGCTTCGACTTCCTTCGAGCCGTGCAGCGAGGAGAAGTTCTGTCTTTGGGGCCTTCTTACGCTTCTGCTGCTGTTGCATGCCGAAACCTTCCGTTCTTGACCATCGGACCCCCAGAGCTAGCGTGCGACATAACCTGGAGTCTGGCACCACCGCCGGTGGCCATTCGGCAGTTTAACTTCCGTCTGGGCCCGCTGTTTAATCCTTTGCTATCGTGGTTTGCAGGAGTGATGCAGCCTGTGATTCGGATTTCATCGTATCCCGGTCCGTGCCACCTGCCTAGATCTGCGGATGGCGTAAACTTTTTAAAGCGCACGTTGCCCACCGAGAAACCCAGGACCCACAAATTTCTTGTGGCCTTTGGGTCTTCGGGGTATCCTGTCCCCCCTGGCATGCAGGAAGTGCAGCCAGGTGACCATTATTCACAGTTTCTCGAAGCTGAGACAGTGGTGTGCCACGGCGGCGCTGGAACCGTTCAGACTGCTGCGATGGCAGGATGTCGTGTGATCTCTGTCGACAACACGTTAGACAGAGACTACCTCGACCCAACTAATTGTCAGTCCGGCATAGGCGCCGAGCACCATGAGGATAAAGTTCTCGGGCTTCTGCTCGGCGTTGATTTTATGCCGTTCTTCATTGCGGGCAAGCTGCCCGGTTTTGGTCTGCCAAAGATGTTGCATTGGTATGCGAGTGCTTACCTGCTCGCAGACTTAATGACGATATGGGCGCTTTTCCGAGCTGTAGGAGCCACATTGCTGGTGGTCCCTGGAGATTGGGTCTCGACCATGATCGCTTCTCTTGTGGGATCTGTGCTTCCTGGCGGGTTTGCTGGGTTGGCAGCCATAATCATAAACGTGTGGGGTGTGTCCATTTTTGCTAGGGCTGGTGTTACCGGCCCCGGCATCGTGATGAAAATCCTACGTTTTGGCCGTATGGTCCCTCAAAGACCTTGGCTTTGGCTGACTTACCTTGTCGGTGGTGTCTACCCCACCTTTATCGTTACGATACTGATGCTCTTCATCGTGCCCGCCGCTGGCGTGACGCGGAGAGCTTACGACCACCCCAAGTCATATTTATGTTTTTCTTTGGTGGTCGGCCTTCCGTTTCATTGTTTCTTGATGAACCTTGATGAGACGGAAGTCCTCGAAGGTTCAGTTTTGATTGATACGGAGAAAGCCCCGTATCGGTTGACAAGGACGAAATTGGCTGTCAAGCCCAATTATGTCTTTAGAGTACCCACATTGATTGATTTTGAATCCATCCAGGACTACAACCAGCCGTCCGCATCGTATGGGTTTTTGACACACAACTGCAACACGGTGCTGTACAACGCTGTGGCTAACAAAGGTGGCTTTGGGCTTGGAGCGTCGATTCTATATATCTTCGTTGCCATAGCATCTGTCATAGCGATCTTCGGGATCGGATTCGCTTTGTTTGTTGGCCTGTCGTCTTTGATATTGGTAGCTTCCTCGGCTATCACTCCGGCGATGTTCCAGACGACGATCAACGAGCTGATCTTCAAATGGCGACGGGCGGCCTTGATCGACCGTATTCGTGCCGATCGGACTGTTCTGTCGTCACTATTGATCATCGCGTTGCTTGTCACCGATTCGCAGGCGGGCTTGACAGACGAGGAGAAACTCGCCTTTGAAGCCATTACGCAGTTTCGTGTTGACGTGGCTAACATGCCTTCACCACCATCTTTGGCAGACACAAGTGCACTCATAAACAAGCACTTAGTGCTTGGGCCCCTTTTCCGGGGCAAGTGGAGAACTCTAGGGATCATGTTTGGCATGATCCCCCACGACTTCACAGATTCTGAGACAGCAGCCATTATCGGCATTCATCCTCTAATTAGGAGAGCTCTCGAATTGCGTGCTGAGTTCAACATTGCCTTGCAACAGGCGCAGTCTACCAAGATCACTTTTAGGGAATTTGCCGAGTTGAGAGAGATGCTTTTGGAACACGATGCTTATGTGTTTCAGAAATGCACCTCGCCAATCTTTGCAGTCATCGACAAGCTGAATGATCTCCAGGACGAGATCGAGGAATTGGATGATCCTTATGACGGAGAAGGGAACATGGTCGACGCCATCGTTGAAGGAATCACTCCTAATACACTTATTGACGTCGAACTTATTAAAGCCTTCGAGGCTTTTCCAATTCATGGGGATGGACCGACTAGCTCTGATGCTTTTAGCAAGATCGGTCTCGATCTAGATGAAGAAGAGTTGGCGATCTATTCATTTATCTTCTTCTCCGATTTGCACACCCCGGAGTTGGATGGCTCTGCGGTCTTTTCCACAGTCAAGGAGCAGCCATTGACCCAGGATGACCTTGATGGTCTCTCCGTGCTGGTTGCAGCCGCCAAGGCATATGGTGCTACAGAGCTCCAGGCTCTGACGGAAGCCGTCTTGAACGCGAAAGACCTGCTACACCCTCGTGGCAGGGCGGTTCTTGTTGGTGCCGATGCCCGGATTAATAGTGCTGTCGATGGCCTGATCGCGTCGACTGCCAGCATTAGATCCTCCTTACAGTGGATCTCTGTCGCGGTTTTTAAGGCACGACAGTTCATACATGCCGATCCCGTCGCTTCTGCTGTCCTAAAGCCCTTCGATGTGGTCTTCACTGTCGTTGGTTTCTTGATGGCGACCATTTTATCGGTCAGCATACGGGCGTTTTCAGCCATTCTTGGCATGTTTGCCAGCCTTGGTGTGTTACAAGCCACCACGGTCAAAGAGACCCTGACTGCTTTAGGCATATTTCTGTCATCCATGGACCCGCGGCATCGGTTCAAGCCCAAAAGCGCCTGGGCACTGTTGTGGGAGCGCAGCCGTTTGGTGCTATCAAAAGGAGAGTCTTTGTTGTTTTCTCTCCGGAATTTCACCTATGAGCACTCTCCATCATACCACGACTGGAGTGGCAAGATGATTGAGTTGCTCCGCACCAAAGACATCGATTCCAGTGTCCTGCATCGCAATCCTCCGAGGCGGGCGGTATTCTTTCCGTCCCGCCCAGTCGGTATCAAGGAATACGAAGGTCTCACGGCGGACATTAATCTGCACTTTGTTGAAATGGCAAACGCTCGGCGCAATATGGAACAGAGCTTGGCCGCTGGCAATGGGCTCGCTATAGATGGTGCCTGGATGGCTCGCCCCGAGCACGTCACGTCGAGTCTATTGCGGTATACGGTGCCGCGACCTGCTATAGACTCTGAAGCCCGTGCGATGTTGTCATTTGCAGCGGAGGCAATATTTGAGCATCACCCCGAGCTCTATGTGAACCCCATGCCAATGAGCGTCTCTCAAGTCCTCGCAAAGAGCAAGTGGAAATACTCAGCCGGGCTTCCTTTTCTCCCTGTCATTAAGAAGAGAGAAGCCCTGAGACACAGTGCCTGGTACCAGGCTATTGACACTGCTGCTCAACGCATCATTGACAGCGGCGTCATGCCTAGTGTTGGTTTGCATGCCTTTCCAAAGGCTCAAGTCGTGTCCTTGGAAAAACTTCTCAACAATCCTGCTGCAGTTAGGACTGTCACTGCCGGCGACCGCATAACCGGCACAGTCTTTAACACTCTGCTCCTGGAGAGAAACAAGCGGGTGCCTCCAGCCCATTACGGACATGTTAACATGCTTCGTAGGAGTGAAGGCGGCGTTGCTTTCCTCGAGGAACACCTTTCGGCGTTCCCGTACTTTTACACTGGGGACGGCAGGGCCTTCGATTCTACTGCTGCTTCAGAAGTATCCACAGTTGGTTCAGTGCGATTATATGAGCTTGGCCTTCAGGCTGGATTTGCGTTCAACGAGAAGGCAGCTGTGTCTTTGGTAAAGTCTTATTATGAAGGGCTGACTAAAGGCCTAATTGTTAATTTGCTTAACGGCGATGAAATCATAAAGACTGGTGGCGGTGGAACTGGTTCAGTTGCCACAACTCCTGACAATCGAGACTGGGTTGAGTTGGTTTTCCTAGCTGCATGGGGGCTAGTCACCAACTCCCACCCCTCCAGCTTTTACGACCATTTTAGGCTCGCGCATGCTTCCGACGACGTTATCTTCTCGACGGATGAGTATGGGCGTCAACATTTGCAGGAGTGGGTCGATTGTATTCGCAGCCGATACGGCCCAGACTTTTCTTTTGTCTTGGAAGACGGCGTCGACAACCTCATCCACTTAAAGAGAGTGCCTATCTCGAGCTTGGATGAGGCCCTTTATACAAGATTAGGTTTGCCCGTGCCTTCTGTAGGTTTTAGGCATGACCCTCAACGCTTGCTATTTTCTCGTTCAGCATACCGATCTGACAGGGCTAAGGCTAATGCCGAAGTGATGTCGTCTCATGTGGCTGAGCGTTCTGCAGGCTTCCAGCTTTTGACGGCTCATTCCCCAGCAGAGTACGACATGGTCACCCGCGACCTTGTCGACGCCAATTTGGAGTATGCCTTGTTGTTCTTCCGTGGCGCCCGCGCCTCGCTCGAGCTTGATGCCAATGGAAATGTGATTGATGGTCTTGTAGAAATAACAGATCGTCGTCCGGCCAGACATGTCAAAAGGTTAGCCAGCAAGTTTGGCGTCGGTTCCAATGCACAGGCTTGGATTGCAAACCGGCAGCGAATCGCGGAGTCGCACTTAAAACAGAGGCGAGGGGCCTCTTATCTGCGAGTATTTGAGACCTGGGTCAAGCCGCCCCCGCCTCAGGCTCAAAGCAAAGCTTACCGGAGGTGGTTGACTTATCGAGCAGCTGCCAACTCATTAAATCCACTCATAGATGTGTTGAGACTGACAGCCAATCGTATAGATTTTGCGCTCTCGCTTGTCCCTTCTTCCATTGTTAAGAGCTCTGCTGAACCTATAGCGTCGAGGTTCTCAAAGCCCTTTACCACCTTTGACTTCGTCATTGAGCAGTACATTTATCGCAGGGAGTTTTGTCGTACTGGGCAACCGGTTTCATTCGAGAGAATGGGCGTTCTCATTCGTGAATCCCCCTATGCCGCGGCGACCGACATATCAGCCTTCTTCTCTGTATTGTCGTACCCCACAGCACTTAGTGCTTTTCATAAGGCGATTCGGACGAATAAGGCCGTGTATGACCCCGTGTATTTCCCTTCGAATGATACCAACACTGGCCAAGTGGTTGTTTATCTTGCCTGGTATACTGCGATTGATCTCATAATCAATGCTACGAAGAATTTCCCATTGCTTGGGGCAGTAGTGTTATTGACTTTCGCCGCGTACCGGTGGTTGGATATATTTTACTCCATTCAGAGCTTGGTATTCTGGGTTGCAACAGCGACCGCATCTTTGCCCATTTCCAATTCTTCGCCGAAAGATAAGTACGCACTGCAAAAAGTGCTAGCAGCCATTTTGACTGCTGCGACTCCTTCAGCCTTGACTTGGTACTTGCCAGGGTTGTATTCGGCGGTTGCCAAACTCGGCCCTATTGTTGAGTTACAGGTGTGGATACCCAACCTGTTCCAGAGTGTCCACCAAACAGCGGTTTCTGAACTCCGTGAAGTGCCTGCAGCAGCATTCGCTGACTTTCACAAGGTCTGGAACATTTCCCTTCCGCGGGTTGTTTTGAACGCGCCCACAGGCACTGGCAAAAGCACAAGCTTGCCCGCGAGTATCAAGTCACGGTACCCTAATAGCACTGTGTACCTGCTCCTGCCCTTCAACCGCCTGGTTGAGGATTACTTTAATCCCTTTTCCTTACGCTTGAATGTGCAAAAGGTTACACGCACCACGCAACGCGACAATCAGTTGCTAGGTGACATATTGGTGATGACACCGGGTCAGTTTGTTCGTCGGATTCGACAGCTTCACCTTGTTGACGCTGTGGTGCTTCTCGATGAAGCGCACCTCCCGAGCAGTGAGGCTCTTGCTGCATATAACACTGCGCTCGAGCTTCAATTGTGTGTCGTTCTTATGACCGGCACACCCGGACACTTTTTATCGGGAGGCCTATTTGGTGACTGTCCGACATTTGACGCGCCCCCTCGACTGACATATCAACGAGAGATTTTCGAGCCTTGTGCCTCGTTTGATGAAGCCGTTCAGCTTCTCACGAGGCACGATGTCGAACTCAGAGACCTTTTGGTTTTCGATCCTAGCATCCAAACCTTGGATGTTTGGGCATCGCAATTCCAAGGCCTTAGAGTCCCGATAAAGAAGATTACAGCGTCGATTGGAGACAACATGCAGAGGGCCGCTGCCTTCGCTACCGGTGTGATAGCAGTGGGGGCGAACTTGACGCCCCCGCCTGTCGCGATGATTTGTTCCCCAGAACACCTGGTAGCCGCACCATCCAAGAAACTGAAACGGTCGCCTGAAGGGGTATACCTGGTCGAGCCGGCAGAACTAGTACATGCTGTCGTGTTGTCTCGGCGGCAAATGCCTCCTCATGAGAAGCATCAGTTATTTTCTCGCGTTGGTCGTTTGCAAGATGGTTTAGTCGTGCCTCTCTCGTCCGACTTGCCAGAATCTTTTTCTTCCTATATAACTATCGCTGGCCTGCTCGCTGCCGGTGCGACTGGTAAAGTCTGGGCAGCTTTTTGTGGGTTGCGTCACTTTTCTATCGTGCCGGGAGCTGCATGTAGTCTGTTTAAAGTGAAAGAGTCATCGTTTCTAACACCTGAACAGGCCGCAATAGCGTACGGCTTTGCAGCCGCAGTGGTTTCTATTTCTTCTTTCTCTTCTTTGGCAGCACTTTACACAACTTCAGAACGCGAAGAGTCAGAAGTGCAGCTAATCGAAAGTATGCACTCACAATTTTCTGTGGATGCATCTTTTTATGATGTGCATTTGAAGGTGGCTATTGCCATGTTTTGTTTGCACTATGAACTTGATGTCAATGGTGTTATTTATCCTAACACCATTCCTATCATTTTAGAGCAAAAGCTTGAATTGTTCAAGCTCTCTTCCTTATTGTGATTTGCAGCCAGGGAGGAACGATCCCCCTCTATGTGATTTGTGTGCTATGAGCAGTTACACACGCCC

> SrHV6

TCTCTTAGGTTTATGATTTTGTACTTACGCGATTTAACGCAACCTAATAACTTGTGTTTGTTCCAAATCGTGGTCTTAGACCTTGGAGTCGGCTTCTAGTGCAGCCGTTTCTGGCCGTGTGTTCTGCTACACGGACCTAAAGCAGGGGTTTATGGATTTGTCTCTCACAAATCGCGGGGAGGGCGACCTCCTTCCGGCACGTCGTAGACGGCGACCGGATTAATCAATACAGCTAGTACTTCTCAGCACGCTATAGCGCAGAGAACACCAGGGGACGTCAGGGAGAACGAAGCACGTCGCGACCTCGCGAGGTTTCTACCTCCAAGGGGGGCTGCAGGCTAAGGCCGTATGAACGTTGTACTCCCGCATTTTGGATAGATACTGATCACGTGCGTCCAGCGAATCACGAGATCCCTCGTCCCTGTAGACGTTAAATGGCAGCGCAGGCGGCGACACCGCCCTTTCGGTTCTTACTAATCACCGCATCCATAAAAGCGGAGATAGGCATGGGTGTCCAATTAATCAGTATGAGATGGGCAGTGCCGATCGCTAGCGGCCCACGGGTCTGACGATAGCGTTCCGAAAGGAAAAGGGCTGATATCAGATATCAGATATATAGGCCGGCCCACGTCCGTAGTTCCGGCTGTGTGCAAACCGGTGGCCTAGCTTGGGACTTCGATCACCCCAATGACGCCATGAAAACGATTCATCCAATTCTTGCTGTGTTAACGGTTTATCTTGGGCGATAGAGCCAACTAGCTAAGATCGTGGTGAAATGGTTCGCCTTCGTGGATCCTCCCGACACTGTGGTTTCGACACCACAGGTGCTTTCGAACTTGAACTTTTATGCAATTACCGGCGCGGGAGCGCCACATTTTGGAGTCGAGACTAAGATTACATCAGTTCTATATGACGTAGCCACAGAGAGCTTGCGCCTCTCGCGTTGGACAGCTTATGCCAGTGACTAACGATATCAGATGAAGCTCTGCGGGATCTTCGGTGAACCAACTTCTTTACCTGTACGAAATAAAGGGATCTGTACCTCGTCTTGCGTGTCTGAACGGGACCGAAAATCGACTTCTAATGTAGCAGTTTGCGTATGGAAGGGATGGCTTGATAGTGCGGTTTGGAGGTGGTAGCGTAGGTTGAAAGAGTTCTAGGGAACAAAACCTCCATACAATATGAATGTTGAAAACACTCATACACCACCCGGGGACGAAAGAAACGTCGGAGGACACGGCGCCACTGGTCTGGCGCCGTCTCAGGGGGAGCCGTATCCCACAAGAAACGGCATTTATTATTTCTCTTTCGACGATGAAAAAGGCGTCGGAAAGAAGCTCAAGGTGGAGGTGAGAGACGGGGACATCGTGTGGGAGACCCAAGCGCAGCGCCAGAAAGCGCTCGACCTCCTCTGGGCCGGCGGCTCGGTCGCGCAGTACAACGCGATGACACGCAGCCTCGTGCGGCCTCGAGGCATCCTTCGCCCCGACGTGTGCGGCTACTCCGAGCACGTCGACCGGCCGATCCGAGGCACCGTGGTCGCCAAGGATCGCGAGGACCAGCCTTCGCTCGAGATGCGGACGTGGTACGCTCGCATCAAGAGGGCGGACGGCCTGGTCCGCAATCTCGGCCGGGCTGAGGACCGAGCTCGACGGCAATCCCGCGCTGCCGATCGCGCCGAAGCGCGAAAGGCGGTCGAGGAGAAGTTGGCGCGAGTCCGAGAGGAGCGCAAGCGCGAGGCGTCGATCCGCGGGCAGTCTGTCGCGGCGTTGGCGAAGAAGCTCGACGAATTCCGGGGCGTAGTCCACCCTCGTCTCACTCTTGCCCGCAAGATGGCAGAGCAAGCCCGGCTGGCAGCCAAGCGCACGAGGATCGCACAGAGCTATGCCGCGTTCAAAGAACGGCGAGCTGCTCTGGAGCGTGAGCGCGCTGAGCTTGCCATCAGGCACGCTGAAGAGTGGAAGCGGGAGGGGCGGGCTGCGTACCTCGAGCACCTAGCGGCGTTCGGCCGCAAGCGCTACACTCGTGGCACTTCCATCTGGAAGGCGCGAGCCCGGGAGGCCGTCAAGGACGTCAAGCACGCTGCCCGATCGACGTTGGTGGCGAGCTTTGCGAACAGCTTCGCGGTCCTCGGAAGCTTGGCGAGCGAGGAGCCGGCCGCGACGGCCGTTGCTCATGCGTTTGGTGACGGGGAGGACTGCTCTCTGGTCACGGCGGACCCTGCTTACTCCCGGTCTGTCGTGCTCGACGCCTACGGCACTCTGGTTTCCGGCATCTACGAAGCCGCGAAGCAGGAGGCCCGCGAGGCGGTCAAAGAGAACGCTCTATACGCCCTTGCCGGCGCGTGGTCGCAACGCCTGCCTGGGCAAGCTGACCGCTTCGAGCTGACGGCCGCAGACCCTGCGCTGGCGGAGATCGCTGGACCCAACGTCCCTCGGTTGCACGGGTACGAGTTCGCTGCTATCATGCGGACGCCTGATGAGCTGCCTGGGCCCTGGACGCTGTCGACGTGCCCGATCGGTATTCGGCGCAAGTGGTTTGACTCACGCTTCGCCGGAATCGGCCTGAAGCAGTGGTTCGGCCGACACGTCGAATGGCGGGTCAAGGCTTGGGAGACGTCCGGCGACGAGCTCATCCCAGCTCTCGACGCCGTTTGGGGGCCTGTTACGGAGCGCGATCCTGAGGTCATCAAGGCGGATCTGGTCGACGCCGTGCGCCAGGCTGGGTTGAGTGGTGCGAAGGCATTCGCGTGGCTGGCTGCTCAGCCGGCTCACCGCTTCGCGACGCTCACTCAGCGCGTATGCGATGTCCTGGCGGACGCGTGGGAGGCGCTACCGGAGGTGCCCATTCAGGCTCTGCGGACCGTGGCCGATCTGATCGCGGAGTTCAAGCTCCTGACGCGCAAGCGGCCGAAGCCAACATGGGCGCCTCTTATACCCGTCAGCGGGCAAGAGGGTCGACGGCGATACGCTCACCGGTTCCAGCGGCTCCCGACAACCGGAGACCTGGCCCTTGGGCCTCGTAACTACGAGACAATAGTCGAGTGGTACAAGGTTCAAATGGAAGCCGCCGTGAGTGCCGGGTGTGTCCCGATCCCCATCAACGAGGACTTCGTCACCCAGATCAACCGTAGTCCGTACGTGGACTACCATCTGGAGGAGCTGAAGGGCCATGCGTTGTTCCAGGATCGCCACCTGACGTGGACGGATCGCTCGATCGAGGCCGACAGAGCTGCGCTCGCAGAGGTGACGGGGCGCTACTTCGTCCGGAACGAGCACAAGCTCCCGGAACGGAAGATCGCCGAGTTCGTCAAGGCCAAGTACTGGCAGCACCCGGAGCTCTATGCGCATGCGGACATCGCTTCGCCTGGCGAGATCTTGCACCGGCTCAACAAGAAGAGTTCGCCGGGCTGGCCTTTCATGGACAAAGCGTTCTCCAAGCGGAAGCTCATCCGGAACGGGCAGTGGGACACAATCATGTTCCTCGCCGACCGCGCCTTGGCTTCGGACGAGCACATCCGCGACGTCTTCCAGGTGAAGCCGAAGTCCCTGGTGTCATCGAAGCTGCGAACGATCTCGGCGTCCGGCCTCGTCGGCTTGGTGAACTCAGAGCGGTTCTCGATCTCGACGAAGAAGCGACATGATCCGTACGGCGCTGAAGGTGTCGCAGGCTTGCCACTGAACGGCTGGGGCTTGAACTTCTTGTACAAGCGTGCTGCCAATCGTCAGTACCATGTCAAGCTGGACGCGCGGGCGTACGACTCGACTGCTTCCGCCGTGCTCGATGTCCCTCGTAGGCTGCGCAAGCTCGGCTTCGACTGGCACCCGGATCGCGAGGCCATCCATCGCCTGATCGACGCCGTCTACGACTCCCTGGAGGACACCTACCTCGTGGACGTCATCGACGACGGAAGCGGGAAGCCGGCCGTGTACGAGAAGAACACGGGCATCGCGACAGGGCACGGGTCGGTCACCTTCGACAACAGCGAGTCAGAGCCCGCGAGCTGGATCATCCCGCTCTCCATCAAGTCTGGTTGGTCGATCGAATACATCATGACCAACTTCGATGAGGAGAACATGGGCGACGACAACTGGAAGCACCATGACATTCCTGAAGGGACTGTCACGCCGACCGGTTGGGTGTTCAGCTGGGACAATCTCTGCGTTGCAATCGAAGAGATGACCGGCATCCACTACATTGTGGAGGGCAAGAACACCAGCGTCGCTGGCATTGACTTCCTGTCGAAGATCGGGCTCGAGTGGACGGACGCGAAGCGTGCAGAGTGCTTGGCGGCGGGCGTGAACCCGGATGACTTCTTGTTTGCGGTGCACCACAACATGCAGTCATGGAGGGCCCGGTACGTCGGGCTAAAGCAGGACGGCTTCCACGTGGCTCAATATCGGCGACCCACGGATCGATGCGCGGCACAGCTCCAGAAGATCAAGGGCATGGCCTCGTTGCTCGCTCATGAGCGCGAGATGTACGACTTCCTGGTTGAGGATCGCGAGTACTACCTCAACAAGCTCGAGCGTTACAACCCGGCGGCCGCTAGGCAGCTGCGTGGACGCAAGGACTTGCTGATGCCATCGTACAATCGGGTCATCAGGGAGTGGTACAGCGAGCCCAAGAAGGACGCGCGCCGCCACAATCAGACGCGGCGACAGGCCAGGCTCGACGCCATCAACTGGAACTTCTACGCTGCGGACCGCGCGCTCCGGAGGGCCAGGCACGCTGTAGGGCGAATCGATCCTGAGCGCTACGATGTCCCGGATCCGATCCACGAGATCCTACCGCCTGATGCGTTCGATCCCACAGGTGAAGTGGAAACGTTCGCGTACTACGCCTGGATCAACGAAACGTGGAAGACATCCGACCCGGACTTCCTGCCGCGACCGACCTTGAGCGAGCTGACAGTTGTCCTGCGAGAGAGTCCGTTCTTCGGCCAGACATCGGCGACGTGGTTCATGACGTACGTCGAGCCAAGGCTGCACGCGGAGCTGCTTGCACACCCGGAGCCCAAGACTCGAGTCCGGCACCTGGCAGGCAAGCACAGGATGCGAATGGCTCTGGGATCTGCGATCTACACAGCGCTCACAATCGGCTTCTACAACACGCCGGCAGGCATGCTGAGCTTCGCGCCGATCCTGTTCGACATCTACCGCAATGGTGTCAGGCGGCTATTCAGTTACCTGAGCTACGCGTACTGGCTGGATCAAGGCCGGGCAAGCATCGAGATCTCGAACATGGTGCCGAAGGACTTGTACGCCCCATTCAAAGCGTGGGCTCTGAAGTTCCTGCACATGATCCCGCAGTCGACGCCGATGCCGGACTACCAGCGCGTCCCAGGCTTGAAGAACTTGTCCCTCGCGCCGGTCTACGAAGCCATTGCTACCGCGGTGAACCTGGGTCAGGGCATTGCCATTCCTCGGCGTGACGGTGAATCCTCCGGTTTCAACGAACGGGAACAGACGGTCGACCCGTGGGTGGCGGTGACGCAACAGGTGCTCGAGAAGCTCTACGCCCAGAAGGGTCAGCTAGCGGTCGCGCTAAGCTCGCCGACGGGAACAGGCAAGACCACTAGGTTCTCGCGGCAGTTACTGCAAGGCGTCCACTGGATCCCGGGCTCGATCATGCCGGTGAGCTTCCAGCGTGTGATCATCCTGGTGCCAACGCGCACACTGGTGGCCGAGACGCGTATCGTCGTCAATGGCGTGGACATCGTCAAGAGAGTCACGCGGCAGGACGAGGCCTTCCGCATCCCTGCTGGGGTCGTCGTCATGACCTACGGGTTATACCGCGCGCATTTCGCACAAGCCCAGAGGTACAGCGTCGGTTCGATCGTGCTTCTGGACGAGTTTGCCCTCGGCGAGCCTGACATGCTCTGGGTTTCAGAGAGCTTGCGCCGCGTCGGCATTCCTCGCGTGGTCATGAGCGCGACCCCGGACTTCTCGTTCACGACTGAGCGCTTCGAGATGGTTCGCTCCCGGATCCCGCCGCGCTTCAACGTCACGAAGGTCGAAGTGGCCGACTCCAACTGCCTGGATATCGCCGTTGGTTTGTTGTGTAGTCGTGTCGCGCGCGACGCGGGTGTCTGCGAGAAGATCCTGATCGTTCACCCCTCAATCGCGGAGGGCGAATACATCAGGAACTCCTTGCTGCATCTGCTCCCTCGGATTCAGATATTGCACCCCGCCTTCAAGGTGGGGCTAATGAATTCGAAGGCGCGCGCTGTCCCGGATACCCAAGTCATCGTCGCCTCGTCCATAGCGAGGATCGGTCTGACCATTCCAGGGGTAACCTGCGTCATAGACTCCGATCTCGTCAGAGGGCAGCACTGGGGCACGTTGGCTACGGTGGACCAAGACGTCGACGCAAGCATCCAGCTCGCAGGCCGCACCGGCCGCACGTGTGATGGGTGGTACTTCCGATGCTCGCAAAAGGCTTTCGGTCCGCGAATGGTACAGACACCCTCGGCGCTTGATTACCTCGAACACCGCGAGCTGTACGATAGCACCGCGACCTGGAAGCCCGTCTGTGCGTTCATCCCCTGGACGCGGCAAGACGGCTTTCGCGCGCTCCGCCATCCGTACCTCGCTGTCGACGAAGCAGCAGTGGCGCCCGAACACGATGAGCAGCTGGGATGGTTCCTGAGCCTCGTATGTCAACAGCCGAACAACGTGGGCTGGGACGAACGTCTGCGAGTCGCATCTGACCAGTGGGCGTTGGCGCAGTGCAACGCCCCCGCCGAAGAAGTGCAGCACCTGGCTAAACTTCGGGATCGTTTGACCGCCAGCGCGTCCGTCCTCGAGCAGTACGTCGCTAAGGGACTGATCGTCATGCGTCACGCAGATGGGCAGGTGTTCCGAGGGCTCCCGTGGTTCTACGAGGGCGCCATCCGAGAATCGTGCGAGGTCAAGCCAATGCCTCGAAACCAGTACCAGCGTGGGTTCACAGACCACATCCCGGCGTTGGAAAAGGCGATTCGGTCCGCACGCGTTCCCGTGGCGGTCGCTCCGCAGGCAGAGAGTCCTGTCCCGTCTCGGAATCCAACCGAGACTGCCAACGGGCGGCTCCTTGCCGCACAGCTCTATCCACCTATCGGGGTTAAGCTGCCACGCACGACTGAAGAGGAGGCTGAAGAGGATGCCGTCCGCCGAGCGTTAGCCTTGAAGTTCGCACCAAAGAGCCAGAGGGCGCTAGTCGTTGCCAAAAAGAGTGCCTCGTTCGCGTACAACCTGCTGTTCCATGCAGATCTGTCACCGGTGAGGAAGGCCTTGAGCGGAGGAGCTCGGGGTAAGTAGAGTGAGTGTGGTGTGTTTGATAGGTTTTCACTGTCTTTACGTATATAAGTATAAAAGTTAGTGCCCGCCRGGTATGCGGGA

> SrHV7

ATAGCGTTTTGGCGTGAAATATCATTTAACCTCCATGTTGTTTTCTATTACCCCTTGTGCAGGAGCTCATGTCTTTATCGACGGCTCCCGAGACGCAGCACTTGTGGTGCGTCTAGATGGAAAAGAAAAGAAGATTGGAGAATCTTCGAGACCCGGATTTGCGCGGGTCGATAAAATATTCGCAATAAAATTTACTGCTGCCTCCCAGTATAAGGAGGTTTGTGACGGCACTGGCCCGTGTCGCCACCATGGGCGTGCATGCCCCGAGTTCGTCGCTGCTCGTAGGAGGGCAGAGACTGCTCGCCGCGTGCATGCGGAGCGCCTGGCCTCGGCGCTAAAAAATAAGATGAGGCTACAGCAGTTGCGCTGGGAGCTCGGGCGCTCCCAGCGTGTACTCTTCGAAAAGCGTCGGGATGAGCTCTTCGAGGAGTATAAAGATGCTCCATATGCTTTCCGTGAGGAAGCCATTTGGGGCACCCTTGCCGCGATTGTCGGTGACGCTGGGGTGAGGGTCTCCCAGCTGTGGTACTTGTCCGCGAAAGAGCGGAACAAGTTGCAACACGCATTGAATGGCAATATTCATGCGTGGTTTAAGTCGCTTAGTTCTTTTTCGCGGCGCGATAAGAACCCGGCGGCCCCAGACAGGATAGCGCCTCGGCCTGTCCCCCCTATTCCAGCGGCATCTGCCACTGCTCTTCCAGCTGGTGACAGGTTTGTTGCTCCAAAGGTCGAAGCGCATGCTTTTGACAACAAATCGTCACACTCCTCTATTGCAGAGACCTTCCAGTCAATTGTCAGTAGAGTTTCGACTGTTCGTCATGAAGGGCTGACACCTGAGCACCATATGCCTTCGGTACCTCTTGCTACTCTGACTGACAAGCAGCGTGAGTACTATCAATTGTATTCGCGCTCTGCGAAAGAGGAAGTTGTGCCTCCTCGCCCCTTTGTTCGACCTGGCTGCTGTGCTCCAAATCCCTTGCGTGAGGGCAACGAGTATGTTATCGGCCATGGCGCTGGGTACCTGGTGTTGTTGAATGACTTTAAGGGGACGCCCTTGGTGTGCGTGTGGGCAAATGTCCACAAGCACTGCCCAACATTCCCTTACGATATTTCATTCGGAGCACCATTTTTAGCGACTTACCCCGAACATTTCCATGGGCACATTCTTGATTGGAGATGGAATGGTATTAAAGGGGTTGCATGCCAGTTGCCCGCTGGCATGCGTATTCTCGATTATGCTTCATGGACATTCCCGTCCATAAAGCACAATTATCGGCAAGCATCTCTTGGGGTTACGCTGAAGGTGCGAGTTGCGGTTTGTCCTAAGTGTGACAAATTGCTTCCAATTCATCCTGATGCCACCCAATGTGAAGTTTGTTGGTACAAAGAAAATCTGCCTGAAGATGTCACCATGCCCCCGCGTTGTGTTGCGCGTTGGGACAGGGCGCCTACTGCCACCTTTGGGCAGAAGCTTATATTAGTGGCGCGGCCAGTGGCTAAGAAACGTCATCATAAAAGGGTCCTTCCATCGCCCCCTGATGTTTCTCCAGTCTTGTCTATTCAAGACACACTGAGGTCGCCCCATGTTTCGATCACCGACAAGATGCACTCGCTTCTTGAGTTGGTGCCCGATATTGCGGATAGGGCCACAGGGCAGGCGTTTGTTAGCTTGGCTGATTCAACAGCTAAGAACGTTGCCGAGAAGTTTGTTGGGCCTACGGCAAATAAGCTTCTTTCTGCCCCTGTTAAACACAACAAAAGGCTGCCCTACATGAGCGACGAGGAGATTGATGCTCTCGTCGCTCTAGTTTCTGCTGCTGAGGCTCTCAAGAGAATCCCTCTGGCTCGCGTCCAGAGTGACTCCTTGAGAACCCTGTTGTCTGCATGCGTCGACAGGGTGGATGACTCCACCTTGTTCGTCGATGTAATAATGGCGTTGTCCCCTCGTCAGCGTAAGACAGCTGTCAAGATGGCTAACTTCCCACCTGGCTCAAGGAAGGCCTTATGTGAGGAACTGTTCAAGCTGGCCAGGTCTTCTGCAGATGCTGACATAGAGGTGAATCCTGGCCCGAGCATTTGGAGCCGCCGGTTGGCAAAGTTTGTTGGCGAGCACGTCGTCATTCCGGTAGGACCTGGCCAACACTTGCGGGTTAAGCTTGAGCCCTCCAGCGATTCACCTTCATGGGTGCCTTTGGGCTCAGCTGGACCAGCTTATGAAAAATTGCCTGAGAGGTGGTCCCCTGCCTCGCCGCTAAGGACGGAGGAGTTCTTAAAGATCGTTAAGAAGCACTCCAGTAAGTGTAAAGTGCCTCTTTGTGGGAAAAGGACTCTCTCCTCGAATGACCCTGACGGGCTATTCGATGATCTTGGTGGTTGGTCCAGGTTCTTCGATGTCTGCTTTTACCTTGGGCAAAATTTTTGCGGACATCAGTGGGGAGTTAAGACGGTCTGGGACATTGGCGTTGATGCTTATGGTGAAATACCACTCACTCATGCACTGACGTGGGCCCGAGCCTTAAGTGGAGGCCGAACAAGGTGCGTGGTCCCTGACCATGATGAGGCTGCCGTCATGTCCTACCTAAGGACGTTGCCCAGATATGGCGAAACACCATTAAGCGATATGGAGAGCGAACTGGCGCATCGCTTAGGCTATGCCCAAGTGCAATGCCCACTTTGCTCTCCAGACGATCCATATTGGTGTTCCAATTATGAAAGATTGGTTTCGCCTGCCGATGAGGACGACTTTGAAGCCATCATACCATGCCTCAAAGTCGGTTTCAAAATATGGCGAGACGTGCCTGATGACATGCCTTGTGCTGTCGTCGATGACTTGGGCGAATTTCAATCTGACCCTGTCAATCTTTCATTCTTTCGAACTATCCACATCACGTTGGATGAGGACAGGCGTGACACGCCAGGCTATTTTGTATGCACGCCCGAGGAGTACCAGGTGGCTGGTTTGATGGCTCGTGCTGCCGCTGTTTTCACTGGGCGCACTCATCCGGTATTGTACGGAAATGAATTGCGCTTGCAGATGGTGCCTCCTTCGGTGTTGTCCCTGCCTCCTGGCTCGTATGCTTTAATGACTCCGGACGGCCAATCTTCCGTCGGAACATTGGCTTGGCCAGGTTACGATTACCTTTGGGAATCTGGTGGGCTCCACTTAGCTGTGAAAGGGTTCGGTAATGTGAATATGGCGGGTGCCCTTATGCGGTCCGTTATCGAAGGGAACACTGTTTTCTTGGACAACGTTGAAGCTTTCAACCCATGGAAAGTAAAGGCAGAAATGGTTGCACCGCCAGCTGCGCGTTATACTCGGAACACATTCCGTGTCTTGTCTCGTCCTCAGACGGCTATTCGCACACCAGGACTTGGGACAGCTGTTGGGCGTCTTGCCTATATAGCATTGGCTTCGTCGACAGTATGGACTCGTTCTAGCTTTTCAACTTTCGTCCATATGGTGTCCTCTTCTTCTTTTATCGGGCACGAGGGTCAATGTGTTGAGTTGTCCCTAGACGAGGCAATGTTGTATAAGGCGCCGCCGCGCGACAGTTGTGCGTGCTCTGGGTGCTTGCTTGCCGCGTCCTTTGGGCCCATAGCTTTTTGCACCGCAGGTAGTCGTGGCGATCTGGTTCCAGTTCTCGCAATCGCACGCCAACTCGCCAAGCTAGGGCTGGACACCATAGTCATTGACTGCCTTGATGGCAAGGGAGGCGAGTTTTTGCTTGCTGTCCAGCAAGGCGAGGTGTTGAGTTTGGGGCCAAAGTATGCCATGGCCGCAATTGCATGTCGCAACCTCCCGTTTGTGACTGTGGGGCCCCCTGAATTGGGCTGCACGATCACATGGAGTTTGGCGCCTCCACCTTCTGCCATCAGGTCTTTCAACTTTCGGCTTGGGCCTTTGCTGAACCCTTTGCTCTCGTGGTTTGCCGGCGTCACTCAGCCGGTCATTCGCATTGCGTCATATGATGGTGGCTGCCACTTGCCTCGTTCTGCTGATGGTGTCACGTTTCTCAAACGTGCGCCACCATCTGATAGGCCTCGTAAGCATAAGTACCTTGCGGCAATGGGGTCTTCTGGGTTCCCCCCCCCACCAGGGGTTCCCCTCGTGGAGCCAGGCGATCATTATGCACAGTTCCTTGATGCACAAGAGGTCGTGTGCCATGGTGGTGCAGGCACCGTTCAGACTGCTGCCATGGCAGGATGCAAGGTTATCTCAATTGACAACACGTTGGATCGAGACTACCATGATCCAGCCAATTGCCAGATAGGGATTGGAGCTGAGCATCATGCTAATAAGGTGCTGGGGTTGTTGCTCGCTATAGATTTCACCCCAATGTTACTTGCCAACAAAATTGAAGGTTGGTCTTTGTGGACCGCCTTGCATTTTTATGTGACTGGCTTCTTGTTGGCAGATGTCATGACGTTGTTCGCTCTTTTCAGGCTCGTTTCTGCCTCTGCAGTTATCGTGCCTGGAGATTGGATATCCACGTTGGTGGCTTCTTTAGCCCATTCCATCCTGTCGGGATCAGCTGCTGGTATAATTGCTGTTGCCATCAACGTCTGGGGCACTGCTATCTTCGCTCGTGCCGGCTTGACTGGCCCAGGAATCATCATGCGCATTCTGCGATTTGGTAGACTGACGCCGTATCGACCATGGTTGTGGCTAACTTTCATGGTTGGTGGATTTTATCCCACTGTCATCGTTGCCTTTATCATGATGGTGGTTGTTCCTGCCATCGGTGTCACACGACGAGCGTATGATAAGCCACGGTCATATCTCGGTTTTTCTGGGGCTGCAGGATTACCCTTTCATGCTTTCCTGATTAACAAGGATAAGACCCGCATTTTGGAGGGTAGTTACCTCACGAGCGAGCCCGGAGCCCCTTATCGATTAGCTGCGATGCCTTACCGGCCCGAAGATCAGGCCTGGTTCATGGTACCCACGCTGATAGAGTTCGACGACATCGCCGACTACGACCAACCTAGTGCGCAGTATGGCTTTCTCACCCACAATTGTAACACAGTATTGTACAATGCCGTACGTACAAAAGGGGCCTTCGGTGCTGCATCGTTGATACTGTATGCGTTTATTGGCATCGCATCGTTAATTGCGATAGTGGGCCTTGGCATTGCACTATTAGTCGGGTTGTCCTCTTTGATCCTGGTGTCAGTCAGTGCGATAACGCCATCGACTTTCCAAGCCACGATTAATGAAGTGGTCTTCAAGTGGAGACGAGCTGCTTTGATAGATCGTATTCGGCAAGACAAGACGGTTCTCTCAAGCTTGCTTGTTTTCGCGCTGGTTATGACCGATTCTCAATCGGGACTGACTCCAGACGAACAAGTTGCCTATGAAGCTGTCACTCAATTCCGACTTGACCTTGAGGCCCGACCTAAGCCAATGTCATTGGCTGAGACAAGTGCATTGATCAATCAGCATCTTGTCCTTGGCCCACTCTTTCATGGAAAGTGGTCAGCTCTTGGGGTCATGTTCGGGATGATCCCTCAAGATTTTGGACAGGATGGGCGCCATGCGATCGTCCATCCGTCGCCATTCGTCAAACGGGCGCTTGTGATCAGGGAATCTTTTGACAAGATGGTTGATTCGTTTGCCCTCTCGAAGGCTACGCCCCGCCAATTTGATGAATGGAAACAGTCAGCGGATGATGAAGAGTACACCGAAGACTTGTCGTTTTATTCTTCGGCTGCATTTTTATATCAGCAGGTCGAGGAATTGTACTCCGACGTGTCAAAGGGCACATACTCAACAGCTGATGAGCAGGCTACCGTCACCTCTATAGTCGAAGCGTTGCTGCCGAATGTCCCTCAATTGGGTGAATTGGCGACCGTTTTCGAAGCTTACCCGATCCACGGCCAAGGCCCTCTGAAGGACACCTGGGTTGCCGATCTCGGGCTAGACTTGTTCGAGGACGAATTGCCCTTTTACAGGTGGCTATTCTTTGTCGAGACCGATCATCCCGACCATGATGGCACCGCCTTGCTTGGCGAAGCACACACGGCCAATCTGCAGCCAGACGACATGGACGCTATTGCAGTGCTGATGGCTTATGCAAAGAAAGCTGGAGGGACATCCTTGGAGGCTATTACTGAGGCAGTTCTGAATTCAAAGGACATCCTGCACCCTCGTAGCAGGCCCGTTCTATTGGGGTCTGATAAGCGGTTGACGGCTATGGTCGAAGGGTTTGTTTCGGCAACCCAGCCTATACGATCTTCTGTACAGTACTTGGCACGAGGTGTGTACTATGCTCGCCTATGGCTGCATAGTGACCCCGTGGCCTCTGCCATCCTCAAGCCCTTCGATGCGATCTTTGCGCTGATCGGGTTCCTTGCTGCAATGTTTATCAATGTGGCATTGCGACTTATCACCTTTGCGATTGCTAGCTTGGGCGGCTCTAAATTACTTCCAGTCGGGACTGCCAAAGAGGTCCTTACTGCCATTGGTGTGTTTTTGACGGCAATGGATCCCCGCCATAGATACAAGCCCAAGTCTGCTTGGGCCTTGCTTTGGCAACGGTCCAGGCTGTTGTTGACCAAAGGCGAGGCATTGCTATTCAACCTGTCTAATTTCTCACATGAGCACAGTGCGAATTATCATGATTGGGCTGCGAAGATGATCGAACTGCTTGATTCTTCAGGGGTGGATTCTAGCAAGTTGTCATTGTTTCCTCCTACTCGTGCTGTATTTTACCCACGGCGCCCAGTCGGCATTCGTGAGTATAATGATATTGTGAAGGTGGCAGATGTCAAGTTTACTGAGATGGCAAATGCACGACGCAATATGATGCAAAGCTTGGAGAGAGGAAACCCTCTGGCCATCGATGGAGCCTGGCTTGCCCGCCCCGAAGACATCACTGCCTCATTGTTGAGATATACGGTGCCTCGCCCTAGCATGTCTTCGCATGCACGGACAGCTCTTACCCATGCTGCAGAGGCTATCTTCGAGCATCACCCAGAGCTCTATCGCAACCCTCAGCCCATGGACGTTAAGTCTGTATTGCTGAAATCTAAATGGAAGTATTCGGCCGGGTTGCCATTCTTACCATTGATCAAGAAACGTGAGACGCTTAGGCATTCTGCTTGGTTCAACGCAATTGAAGTTGCTTGCAATCGCATTATTGAGTCAGGCAAGATGCCTTCTGTTGCGCTACATGGGTTTCCAAAGGCTCAGGTCAATGACATGGAGAAATTATTGGCTAATCCCGCTAAGGTGCGTAGCGTCACGGCTGGCGACCGTATTACGGGCACTGTGTTCAACACGCTTCTTTTGGAGCGCAATAAGAGAGTTCCCCCTGCTCATTATGGACACATTAATATGCTCCGTCGTTCGGAAGGAGGAGTCAAATTCCTTGAGGAACATTTATCCACCCATCCTTACGTGTATTCCGGTGATGGCCGCGCTTTTGACTCTACTGCAGCATCCGAGGTTAGCACTGTTGGCTCAACAGAGCTTTATCGTCTCGGGTTAGCAGGAGGTTTCACGTTCAACGAGAAGGCTGCGACGTCTTTGGTCAGATCCTATTATGAAGGGCTGACAACTGGCATCATCGTCAACCTGCTCAATGGGGATGAAATTGTTAAGACTGGCGGTGGCGGCACTGGTTCTGTCGCGACCACTCCTGACAATCGTGATTGGGTCGAATTGGTGTTTTTGGCTGCCTGGGGCATGGTGTCTAATAGCGATCCCAAGACATTCTACGATCATTTCGTATTGGCACATGCCTCTGACGATGTTCTGTTTTCTGTGTCGGAATACGGCAACTCCATGATGCAGCAATGGGTTGATTGCATTCGTACCGAATTCGGCCCTGACTTCACGTTTGCGTTAGAAAAAGACGTCGACAATTTGATCCATTTGAAACGTGTGTCAATCTCCGACCAGGACGCGAAGTTGTATGAACGCCTGTCAATGCAGGTTCCTGAAATCGGTTTTAGGCATGATCCTAATCGATTGATGTTCATGCGTTCGGCCTATCGCAGTGACAGAGCTAGAGCCGACAAAGAGGTCATGGCTGCACATGTCGCTGAGAGGTCATGTGGGTTCCAACTGTTATGCGCACATTCACCCACCGAGTATGATATTGTCACTCGAGACTTGGCAGATGCAAATCTGGAGTATGCCCTTCTCTTTTTCAAGGGCGCACGTGCTAATCTGCATCTGGATGCCAATGGCCTTGCTATCGATTGTACGGTCGAAGTGACTGATTTTCGACCAGCAAACCACGTCAAGCGTCTAGCCTCTCGCTACGGTGTTGGCAGCAATGCTGCCGAGTGGATCTCAAAACGTCAAAAAGCTGCACTTCGGTCCTTGAAACAACGCCGTGGGTCGTCGTATGCTCGAGTTTTTGAGACATGGGTGAAACCACCTCCGCCAACCAATGAGTCCAAGGCGTATCGGCGGTGGTTGTCTTATCGATCTGCAGCTAATGCGTTCAACCCTTTGTTGGACTTATTGCGTTTGACAGCGAACAAGATCGACTTTGCGCTATCTTTTGTTCCATCATCATTAATCAAGTCGACCCCTGAACCTATAGCGCCTAGGTTTAGTCGCCCTTTCTCTACGTTCGACTTCATTATTGAACAATACATTTATCGACGGGAATTCATAAGGACAGGTGAAACTGTTTCTTTTGAGAGGATGGGCACACTGATTCGGGAATCCCCATATGCTGCAGCAACCGACATATCTGCTTTTTACTCCGTGTTGTCATATCCGGGGGCGCTTGATGCCCTCCATCGCTCGATTAAGACAAACGCGCATGTTTATAGCCCTGTTTATTGGCCTAGCAATGATGCTCTTCAGATGTCGGTGCTTGTTTATATGGCATGGTACACGGCTATTGACATTGCAATTAATGCAACCAAGGCAGTACCCCTTATTGGAGTGTTTGTCTTGCTTGCATTTTCGGCGTACAGATGGCTTGATGTCATTTATTCTTTGCAATCGCTTGCTTTTTGGCTGTGCACTGGGTCTGCCTCACTTGCCATATCCAATAGTGCACCTAAAGACAAGTATGCACTGCAAAAAGTCTTTGCTGCGATCTTGACAGCGGCCACTCCCTCCGCAATTACCTGGTATGTGCCAGGTATTTTCAAAGCTATAGGTGGTCTGGCAGGCCTGATAGAGTTACAGACCTTCATTCCTAACCTAGTGCAGTCTGTGCATGCAACTGCTGTTGCGGAGTTGAAAGCGGTACCACCAGAGGCATTTGAGCATTTTCATCGAGTGTGGGATTCAACAACTCCAATGATAATTCTCGATGCTCCTACTGGCACAGGGAAAAGCACCAGTCTACCAGCAAGCATCTTGGCTCGCTTCCCACGAAGTCAGCTGGTGTTGCTCTTGCCATTCAATCGCTTAGTCTCCGATTATGAGAATGTTTTCTCATTAGGTTTTTCCATATCTCGAGTGTTGCGCGAGACTCCTCGAGATGTGGTTTTTACTGAACGATTAGTTGTCATGACACCTGGCCAGTTCAATAGGCGGCAACATCAATTTCGATCTCCGTCAACCATAGTTATCTTAGATGAAGCTCACTTGTGGTCGACCGAAGCTGCTGCTGCCGCCGATGCCGCACAGAAGGCCGGGATGCCAATTATCTTTTGCACCGGGACTCCAGGGCACTTCCTAGCGAGCCCTCTGTTCGGGGAATTGCCGATTTTGAAGGCTCCTCGCAGGGTATCCTTTGAACGCAAGATACTCCCAGCCTTGCCCTCATTTGAGCAGGCGGTGTCTGAGGTTGTGGCTCGTGGTTACCTGGGGTCAGAACTATTAGTCTACGACCCGGCTATATCGGTGCTTGAGGCTTATGTTTCGCAATATGAAGGCCTTGGCGTCACTTGCAAATTGATAACTTCTTCAAAGCCTGACACCCGAAGACCTGCTGCTGCTTTTGCTACTGGTGTCATACAAGTTGGGGCAAATATTAGTCCTCCTCCATCTGTCATGATTGCAAGCGTTGAGCATCTCGTGCCAGTCGTAAACAATTCTTTGCATATTCGTGACACTGGTGTGGTGCAAGTCCCTGTGCATGAGATCACTACAGCTATAGTGATGTCTAGGGCCAAGATGCCTGCCCACGAACAGCACCAATTGTTCTCTCGCGTTGGGCGCGAGAAGGATGGCCTTGTCATTCCATTAGCTTCCAAGGCACCTGAGCAAGCATCGTCTTTTATGACTATCTCTGGAATATTGGCGTCCGGCAGCTACAAGGATGCTTTGTCGGTTTATATTGGCATAAGTGAACTTTCCTCCTTGCCTAACTTGGTTGGACAGCTCTTTCTTGAGGATCCACATAGCCCTCTGGGAGAACATCAGAAACGGGTTGCGCATGCCTTCGCGTTACTTGTTCTGAGACTCAATACGTTCAGTGGGCTTCCTGGCATCTATATGCTAGGGCAACGGACAGAAGATGCGCAAGCCTTAGTCGATGACATACTTGCTGTCACCGGATTTTCTAGCGATGTTTTTAGCGGGCATTACATCAATGCTGCTGCATCGTTTGCCGCTAATTTCGATCTGCTGATCAATGGTCGCCGTATTAGGGCCGGGATACCTGTGATTATTAATGCCAAGTTGGTTATTTTGGCTCTTGTATAATTTGTCGCCTGGGAGGAACGATCCCCCTCTATGTGATATGTGCGCTATGAGCAGTCACGCACGCCCCCCTGCTTTCGCAGTTGCTCGCCGGTCCACGTTGTTTTAGGCGCCATCAATTTATATTGATCATGTACTTATATGCTAGCCGAGAGTTGGTACATGGAAATGCTCGGACGTCGGTGTGTTGTTATACAGAGCTCTAATGATTGGTTTAACTCTGGTACTAGTGATTACTACCAATGGCCTATGGGGCTAATTTGATGATAGACTAGGTCAGAGATTTCTTTTTCTTTCTCTGTTCCGCTTACCTGATGCGCCCCTCATTCACTAGACACACCGC

> SrHV8

TATAGGTGCTGTAGGTCTCCCCGTCGCTCACCATTACGGTGTTAGCGTACGAGCTCGTGCGTTGTATGCACGAGTACCCTACAGACTTCTTAGGTCTTGTCGACCCTGCTGGTTGCGCCGTCGTCGTTAGACGGACTCTATTCGGACGTCATTTGTTTTCGTCCAACCCCCCTTCCAAAAGACTGCGTGGAGCGGTCAATCTAGTTACGGGCTGACAGTAGTGGTGCTGTCACTAAGGAACACCAGGCTGGCCTCAGAGGAAAAGCTGAGTAATAACAAACCCGTTAAGTGATCACATAGCCTCGCTAGCATGTGACACAGCGCTATGACTCTGATGCGTCAGGATGCACTAGCATCCCAGGAAAGAGTACGTGAGACAATATGAACACAGTGCGTGACCTTTGTTCACCTGTGCGATTTTGGCTCCCTAGCTGTATGCCGCAGCGACTTGTCTTGGGTTGATGTCGAATAACCCACGTAGAATCTTATCCGAAACCTCTCCACACACAACCGTCGACGCAGCGACGTTAAATACCCGGCTATGCGGAAGTACCGAAAGTCGGGCGGGCGTCAAATCTACGCCTTAAGGTCAACCAAAACTTTGCAATTGCATCGTGTGTCAGATGACACGACCATGAGTCCTCCTGGACACTTCTCTAGCTTGCTAGAGTTGCCCGCCACCACAGCTGTTATAGGCCCTTCGGGGATGGTGCGACTAGTGTTAGCACCCGTGTGAAAGAGAGGGCGCGTTACGCCCTATAAACGCTTGTTAGGATAGCTTTCCCCTGGAGAGGAGGGACTAGTGTCGATGAAAACCTCACAACTCGAAAGAGTATTGAAGAGCCTCGGCGCGTCAATCTTAAACCAAACACCCAGTGCCATTTAAAACCCGAAAAGGGTGGATGGTATAACCAAAGTTCTCAAAAGGAGCCACTGTGTGGAGTGCGACGTATAGTTCACCAGGTCAGCCATAGGGTTGTTTACAAAACAACAAATGTCTTCCTCAAATCAAACCCCTTCGCGCGCCGGCAGGCGGAAAAATGCAACCCGTCGGAGGACCGACGTTAATGAGTTCTCCCTCGGGGACTTAGGACTTGGTTCGTCCAGGTCAAACCCGGTGGTCGTTGTCGACGCCACTATTCCGGAGCTTGAGGTCGCTCCCATTGAACATGACCTCCCTCGTGCACTAGTTCGTCAGATCGAGACGAACATGGGATACGAAACCCAACACAGAATCCCGGCTATATGCCGTGGAGGCTGTGATGCAGCGTCGCCCTGCTTACTGGCGACAAACTTCCGTGGGGAGTTCATCTCCTCCTTCGCGTCCTTCTTGGACGTATTTTTCGGTAAGAAGACGGGTCACACCGTCTCTTACCCAGGGCACGGCACCTTTAAAGCCGTAGAATACAAAGACCCGCTTCAACGGAAGCACATCAAGTGGGTCACCACAGCCGCCAGGCGTGCGGCTAAGGAGAAAAAGTCAGCGGTTTTCGCTGGCATCGCCAAGGGTTGCCCCCCGATTCAGGGCATAAAGTCGCGTCCCAAGGACATGACGCGCCGCGAGTACCGTGCAGCTCGCGAAGCAGAGGCCGAAGAGGCCATGTACGAGGAGTGCTTCGGCTCCATGACAACCGAAGACTTTATCTGTGCGGACCGGCCAGATAACTTAACACTGCCTGCACCTGAGGTAGTTGTTCCCCCTGCGACCACCTCAGAAGTGGAAGCAGTTTCTACGGTTTGGGTGGCCGAACACACCCCAAGATGGTGGAGGGTTCTGAATTGGCCCCCTTTCCATCTCATTCCTCGGCCACAGAAGGTCGAGCGCACGATGGCGGCAACATGCGCGCCATTCAGACCCAAAAAGGAGTGCTCCGCACTCAAGGAGTTTGTCGCTGCGTATAGCAGCCATGCTGACAAGCCTTCGCTCGAGGACACCACCCCCGAGAAGGAAGATTTCGACGCGTGGATGCCTGTGCGAGGTGATTACCTCTTTGATGGCAACTCCGCCGAAGCGCAGGGTGAATTGCGCTACATCCTTGAGCGGTGGGAGCAGCTCAAGATGAAGATGGATGACGAGGAGGGGAGAGACGATGACGAGCTCTTAACTGAGTTCCGTCGTGCCCGCAAGGTGGCCACAGTAGAGGAGGAATTGAAGAGGATAGAACAAGCGAAAGCTTGCTCCACCCCTCTAGGGTTCATCTCTGCTGCGGACGCAGCATGGGCTCGTTCCACCCCTGTGGTGGCACGTACTGGTACACCGACCGTACCCCAAGACAGAGGTCGTTACCCTTGGGTGACGTACCTCCTTGGTAGAGGAGACCTCAACTCTGCCGTGTACAAGACGGTTGCATCAGCACGTGAACACTACGTGCGTGGTGAGTGGGAGAGTTGGCGCTTTGGTGCCAACAAACACAACCCGGAATCGGATCCCCGGCCTCATAAGAAGAGGAAAGAGCGACCACTTCCAGACTTGGGCATGTACCCTCGTCTTACGTACTTCTTCCGTCGCTGGAAGACCCAGGACCAACACGCCAAGTGGCGTGACCAGAGGGTTCAACAGAAGGAGCTAGAAAAGAAAAAGCTCATTCCCGTCGAACCTGTCACCCCTGGTTATGGAATCCTGGCGGTGGAAGGACAGTGGGAGTATGCGATCGGTGAAATTCCGTCGATTACGTATGGCGTTACTGATCAGGTTAGACCAGATCGTCCAGTGTACTCGAAACTCAGAGAGTACTTTACTAGAGGGCGCACCGGTTGCGCTCTCCACATTAACGAACCGGGCTCTGCGTTTTTACGCAACCCCAAAATCCACACAACCTCCGGTCCAAAGCTAAGACGGCGCGGCCTGATAGCGCTCGCTCGTACTAACGGGTACTGCTATCTGCGCGGTTTTGAGGCGAAGTTCTGGAGAAGCGCTGTCCTCTTCTTTGGCGCAAATCCTACTATGAGCAGCCTGCTTCAAAAAGGCTGGCATTATAGTTATGACGTGTCAGGCGACACCGCTCACGCTCACCGGTTCGGTCAGTATGTCACTGTCCATCCTGCGCACCACTCTCTAAGAGTCGGTTCTGCACAGTGGTGGCAGAAGCAGGCCCATGACTGGCAAGCAATAGCGGAGCGCGCGAACACCAACGCGGCTTTAATGTCCGATCAATTGACCGAAGCACGTCAGGAGATAGCTCGTCTTAGAGCGGAGAAAAAGGACGGTGATGAAAACACCCACGTCTTGCTTGACTTAAAAGAATCACTGGACAAGGCATTCCCCGAGATAAAATCTCACGCCACCACGAAAGACACCATTGACTTTTTCAATGCCATGAGGGCGGAAGTCACGGAGTCGCGAGGGAAACTCGGGTATGCAGACGCGGTGAGGAAAGGTTTCAGCCATCAGACTGGATTTGGTGGCCAAACTACTCTTCCCCCTGCCCAGCACAAATCTTGCGAGTCGGTAGACGCGGCACATAAAAAGGAGGTCGCAGACCTGAACGCCACGTGCGCGGCACAAGACGCACAACTAAAAGCAGCTGAGCTTACCGCTGCTTCGGCTACTGTGTATGCCAAAACCCAACGCGAACAGCTCCAACTGTATCGTGATAAGCTCGCTTCCAAAGCGAAAACACTGTCTTTACGAGAGAAAATATGTCTCCCAGTTGAACGGCCATCTTTCTTTTGGCGTTCCATAATGCGCTATAAAGTAGCCATCAACATCCCAGGTGGGTGGTTTGGGCATACTGGCGGCTGGACAGTTTCCGGAGTGCCTGCATTTAGGTCGGGCTATAAATATTACCAAAATCTGTCACGTGAGCAGCAGAAGAGGGGAGGGCGACTCACGGTCCAAGGCTTTGAGAGGTTGTTGAGTGTATGTAACCCAAAGAAAAGGAGGCAGATACTAAAAAATTTAAATATTACAGAAACAGAAATAGCCGAGGAGTTCAGGTTGGCGCGAAACTCCTCGATGGCAGATATAGAGCTGAACCCTGGGCCGCCCAACTCGGTCCACAATTCACCACGTTCATTAACAGAAAAAGTGCTTCGCAGAATGGCCAACGATTACGCTGATAGAGACTGGAATCTCCAGAGTCATGAGGGTAGGGCATCCCAATATTGGAAGACAGACTTCATTTTCGAAGGAATCTATCAATTCGGTGATGGCACGATCCACTCCCATGAGTATGGCAGGTCTAGAGAGTCTAAGCAAGTCTACCAGGTAGACATTGTCCCCAACCCCCAGTTGTTGGACGATCTCTCACAAGACATAACTCTCTTCTCGACGCAATTCGTCGTGGCCACAAAAGACTACCCCGCTGGTACGTGCTTCTGCGTGTCAACCTTCCCTGGTGTGCAAGTTCCCGGCATGGTCTCGCTGCAACTATGGTGGCCTAGAGATGAGCTCAATGAGTACGGTGTGGTTGTACCTTTACCACCTTATCTCCGCTCCAAACTAGGGTCCGCCAAAGCTAGGCTCTTACCGTTCGCCCACGGCAACCCTGACCACATGTTCTACGTCACTAACGGACGTCACGAGGACGAAAATTGGCCTGATCTACGGCCCCTTGTGACCAAGGAACATCCAATTTGGTCAGTGTGCCCAGGGTTTGAGCACCCAGGCATGGAGAACTTGTGTGGTTATGGCGGTTTAGTGCCCATCCAGCACCTGTGGATGTGTTACTGCTTAACACACGAGGCATACGGCGCGGACGAAGTCATCATGCTCTCCATCCTTCGTGTGCACACCTCATCCCCGAAAGGGATAGGTAAGCGCACTGTGTGGCATAATATCACGCCTTTCACCAATATGGCAGTGGATTTTTATGACCACCACAACGGAGAAGTGGAGCATGTGGACAGGATGTCGATGCATTCCATACTCGAGCGGAACGCCCGCCTGAAAGACGTGTACGAACCTCTGCCCGCTCTGACTACCCTAAAGCCATTAGGGATACTCATATCGGGTGGAGGCCGGCCGCTCTTGAAGAGGGCCCAGCCGTCTCTCACACGCTTACAGGCCGTCGACGTGGCATTCAAAGAAGCGACAGAAAAATCCAAAAACAAATTATCGTCCCTCTTTACCAAATCTTTTACTTTTACTAATTTGGACCATTTGTCCGGGAGGCGTTCCCTCAAAGACCAAAGCATTTCTTTAAATTTAAAAATAAAGGAAAACATCGGGCGTTTCAGTGATGCTACGGCAGGTAGAACACAGTCTTTCGTTCCTTTCGGGGATAGCGGCAGAGAGACTTCGTCCGACAAATCACAAACAACATATGACCGCACCATATATTACGTGAAAGAGAGATGGCTAGGCTATTACCTCTGCATCGCACTCGTGTTAACGACATCATGGCAACGTAGGGCCCTCTGGCTCTGCCTGAGAATGTTAGCACCAGTGATCCTCCACTTTGGCACCCACATTTACGCCTCATTTGGGTGGATTTGGACCTTGATCTTTGGTCTGTACATCTTGTCCCTAGGCTTACCCCGTACGTGCCGGGTGATCTCAACCTTCGCTGCGAACCGCGTCCCGAATGACGTACTCCCGCGCTGCGAAGGATTCGCATATACCGAGGGCACTACACCACATTCCGAACTCTACAAGCCCTCGGAAACCTTCCTCATTGCTTCGATAGGCACATGGGGTGATTTGATGCCCGTTCGTTACTACGCTAGGCTAGCAACCGCCTTAGGCGTAAACACGCACATAGCACACCAACCCGTCACCAAGGACGAATTGGCATGTCTAGATAACGGCCATATCACCTTTTCGTTATTGCTGAAGTATGCTTTCCTCTTCGTGAGTGAGACGTATGGATACAAGAAAGTCTTAACGACACAGATGACGCCGGTTCTTGGCGGTGACTCCATCTCCTTTTCGTTCACAAAGAGATACAGGAACCGCATGAACTTCGGCCCAAGCCTTATAGGCCGCGTCCAAAGTTACGCTACTGACTTGTTCCGCCCCACTTTCGTGGTTAGCTGCTTCGGTGATGGCAATTTGCCACGACCCGTTTCGGAGCACGCTTTACTGGTGAAGCGCAAAAACAACAACACCGGAAAAACAGGCCGCGCTATCGGCTCAGCGCCCGCTAATTGGGAACAATGGCAACACATCGAAAATGTCCCGAATGGTGACCACCAAGAGATCATGCGTGATTACAGTCGTATTGTAGTCAACGGCCACGGAGCCCTCTCCGTGGCAGTAGCATGTGGTGCTGAACCAATCGTGGCAAACCCGACTCTGGATAGAGACATACACACTATGCCGAAGCTAACTGACTTTAGTCAGCCGACTGCCGCACCTTTCGTGGGCATGTTAGTTGCTTCAGGGTTTAACGTGCAATTGCCACTGTATTACAAAGTATGGGCTGCATTATCGTATTATGCACGCCACGCTGAGACCACACTCTCATCGTTGCCCCGACTCACCCTCGGGTTCATTCTTTTATACAGTCACTTTGCTAGTTACACTTGGTTGATCCTGGCATTCACTGTGTCAATACCTGGAGGTTTCTCCCTTCTCTCCCGCGTGCCACATGTGCGCGCATTCCGCTCGTTCATAGTCCTACTCTCCCACATGCCTTTAGTGCTTGTGGTTTCTGGCTGGTTCCAGCCCCTCTTGTTTTTCACCCTTCAACTGTACCTTGCGCCTTACATCGTTCGTGATGTTCTCACATTCTGTCGCAAGGAGGGTACTTGGCTCGTCATAGAGAAGCAGCCAGGTGTGCCATGGCCATGGGGACACACCCTGCTATACTCTGAGACATATGACGAAGTTTATGAAGGCTTATTTGAGTTGGAACAGGGACAAGCCCAGCCATTCCGTTTCCAGAAATCCCCCAAGGATTTCTCGAAGGAACAGGCCCGGCTCTGGATCCCCCTCCCTTTCAATGATGCCGTTTTACATCATGAGCTGAAGTCTAAGTCTGCTGCCTATGGGGCGACCCATAACTGCAACACAGTGCTCCTTCCCCCTTTACTTTCTACAGGCCTGGTTGCCTATTTCATTTTCGCGGTCGAGATATTGGGTTCAGCCTCCTTATTAGGCTTCGCCACTGCCGCTCTTTCATTACTCGACCGTATTTCTCCTTCTACCCGTGCTGAGATGAAAATAGCTCTTAGGTTCGCTGACAGAGCAGAAAACCCGGAGAAAAATACCACCAACGACACTGAGGGGGGTACTGATCAGACCAGAAAGGTTGAAGAGGAACAGATCAATAATGACTTTGATGACGATGACGAATTTATGAGAAATCCTGACTTTGACGCCAAATCTGCATGGTCGGTCACCGTCGACTTATGCACTGAAGCGCTCATCCGCTCTGGCCTCTCCGAGGCTGAGGCGGTGGAGCTAGTAGCAAGTGCAGTCGACAGACAACTGGAGGAATGTGTGGAGCTGCAAGTAGCTCAGCCTCCAAGGACCAATGATGACCCGGCGCTCCGTCCATACTGGAAAATCACGGACAAGATAAGGAAATGGCTCAGGCATGCAAATGTGCCACCCGGCACTATTGATCTCGTCAATTGGTTGTTAACAAGGGCTGAAAAGCACTACAACCTATGGAGGCCTATATTATCTTTACTGTTCGTTATAGCACAGCACATCAAGACCGCCACAGAGTTCTGTTTCAAGAAACTCTCTAAATGGATTAGGCGCTTGATTTCCTACTGGTATGACGACCTGCCTACCCGTGACCCCAAGGCCGTCTGGCTTATCCAAGAATGGATGGACCCAGTCGACCTCTCACCCTTAGGGAGGTGGGTCCAGACTATGTCGACAGTCAAACGACAGGAACGATTAAGTTATATGGAAGATTTTGACTTCTACGCCGCTCTTTTAAAACATTATGGAAAGAAAAACGATGTGCCACAGGCTGTTCTAGACAAAATTGGCAATCCAAGCTTCACCCGCACAGTACGCGGAAAGCCTGTCATGTCTCGTGAAGAAGCCGCTTACAACGGCTTTTCTGAGGATGACTATGTGTGGATCGCCGAATGGCAAGAACGAGTGGATTGGCTTGTCGGCAAAGAGGGTATACCTCCTGCATCTGATAGTTTCTGGCGTGGTTACTTACACCCAGAAGAAGTCCAGAGAAGCTGCGATAGATACACTCAGCCCCTGTATAAAATGGATTCTGAACAAAGAATCCTGGCTGACCAGATCATTGAAGAGATGATCGCTCGCTTCCCTGGTATTTTCTTAAACAAACAGATCGTAGACCCTGATGCTGCAATGCGCTACATGATGCGTTCCTCCGTCAAAGGCACCGCCTTAGGGGTGGGCATGACGGAGTATGGCCTCAAGACGAAGGAAGACATGCGCACAACTGGCATGAGTAAAGCTATCGTCGAGGCCGTACGGGGAGCGTACACTAATGGCACTGCCATCCCAATGTTCTTCCATATGAAGAGTAAAGAACAGCCGGTGAAACAAAACATCGACAAAATGATCCGAACTATATCAGCCCAATCTGTTCCGTCGATCGTGTATGACCTTGTGGTCTCAGTTGACAACATGAAGACCTCCGCGTTCGCCTTGGGGAACCTCGGTGGAGTACCTCTAGACTTCCGCTTCCGCAAGGTCTTCAATGACTTGCTCATACAAGCTGGTGGCCCCCGTGGTAAGGGTGGCTATATCTTAAAGGATGCGCATGCATACGATTCGACGATACCAGAGTTAGGTGCCCATGTCGCCACTGGTCTGCGTAAAGCTGGGTTTGATTGGCACCCAAACAAAGGGGCAATCCATTCCCACATCGAGACGCACAAGGACAACCTGAACAACTCATGGTTGGTTAAAGTGAGTTTGGATCAAACAAAAACAAAAACCTACGTGGTCGTAGATAACGACTCTGACGCTCATTACCTTTCAACGCAATATCCTATGCATTTCACCACTAATACAGAGGAACCGTCGCAGAAAAGTCAGAAAATTATACTATTAACGCCAGAAAACCATAAAACCTTACAAAAACAGGATCCATGGCTGAATTACGCGGGTTCGTTCTTCACCTCCAACGTAGACCACAAAGGCCTACAAAACACTCCTTTCCTTTCTAAAACTTCGTCGCGTGAGCTCCGTGACCATTACTTACTGAAAATTGCGAGATACCCGCGTGCTGCCGAGCATCTCTCCAACATCGTGGAGAAGAACCGAGGAGGAGGTACTGGTTCATCTGACACATCCTGGTGGAATGGCATGACCTACCTAGGCATATGCATTGCGTCATACTGCATAGCCTTTGGTAAGACCCCAAAAGATTACTTCGAGGAGGTCAAGCAATTCAATATGGGTGACGATAGCGGAGAGGCAACCAAGACGGCGTGGACCGCAGAAAAGCGAATGGCATTTAAGCAAGCCGCCGCCTCACTGGGCGTGGAGCTTGACGTCGATTTCACTCGGAACATCGAGGAAGTCCAAATCATTGGCATGATGGCAAGATACCCAACTTTGCAAGATTCTCAGACCATACGAGACTGGCAAAGGTGGACTCAAGCCCGCACTGAGATAGACCCTGAGACCAAGCAAGTCATCAAGTATGCAGGGGAACTTCACAAGTCACCTGAACTGGTCATATACAATCAAGGTGACCAAGCAGTGATGAGGCGCACAGCTCTCACGGCATTCAAATCCACTCAAGAAAGGTGGAGGTGGACTTCTATCAACGGTGCCCTAGGCATCGCCAATCTATTCGCATTCCAACCAAGGTACTATAACATCGCCCTGAGTATGTACCTTGAAGATGCAGCAGCACTGTGTGCCATGCATGGGATCCAAAGTCGACCAGTCGTTAGGACTGATGAAGACGGCTATCCGTATGTCCACTGGGTGGGCTTCTCGGAGACCAATCACCAGATTGCCGCGACTAAGGATCTCGCAAAAACACTGGGGCTACCTCACTTCCTCAATTTGGAGACTCGTGACCTTGATCGTCAAATGTATGACCGCAAACTGACAATGCACCAAAAATTGACACTGCGGCAAGCACACCTGTACAATAGGTTGACGAAAACAGGCTTACAGTTTCCATCTTACCGCCAGGTAGTTACTAACTGGATGAAGACCGGTTCCATCAAGCAAAAGAACCTTGAGACTTCAACTTTCGGGACTTACCTACACAAATGGCACTCAGATTTACCATGGGACCAAAAGGCCATGGAACTTCTGCAGTACGCCACCAAAGTAACCGACACAATTATACCCAACGACCTAAAGCGCTTCATTCCTTCAGTCCCAGGACTGTCACCTTCTTTGCCCTGGTCGCCACCTAAAGACATTCTTTCTTGGGCTCTGGCAGGTGCACTCGATGTGTACGATGCTAAAACGCTCTCCGCATATAGTCGTGAGAGTCCCTTCGCCGATTGCATGAGCCCCGGAGACTGGGCTACTACCGAAGGACAAGACCCTGCCATCTTAGAGCAGTTTAAGGAATATCCAAAGCAATTTTACAAAGCCATATTCCTATGGAGTTGCGTAGGCTACATGCTGGTGGGGTTCGTCCTCGAACCCCTCATATATCGCATGCCTATATTAGGCCTCATATACGCCTTCTTCCTGTTCACCCAATTCAAGATGACCAAGTGGTATTCACTACTCAACTGGCTCTACTTCTGCGGCCGCATGCGCAGTAGTCGCGAGATTAGTTCCTTACAACTACGAGATCCTTTCGTTGCATTGAAGCGCGCGTCTGTATGGCTAGCATGGATCACGCTAGAATACACAGACTATGCGCCCGCATGGATTTTGATGCCCTTTGTGTGGGTATTCGAACTATTCGCAGACCTGGTTGAAAGCTGTGCCCGGTTAGTGCGTCAATACTTGCTGGTCAAGTGGGTGCAAGCCCGATACGGTGATAACAGCGCCGTGTCACAGAACAACCCTTGGGACCAATACGTCATGGCCATCCACGAGGCTATCGAAGACAAAAATGATGTGATAATAACTGCCGGTACAGGAACAGGTAAATCCACTTTCCTGCAGCTTTCAATCCAAGCCGCAAGGAATGATGGAATATGCCCACGTTTCGGCGATCGCACCGTTATCTGTCAACCACGCCACGTTTTGGTCGACAACCTCAAACTCCCCGAGGGTTGCACTTCCCAAATATTGGAGGGCAACACACCAATTGACAACTCAGTCACTCATTATATTGGCACTTACAAACACTTATGTAACCGCCTAACCGCGGGACAATTTGATGACCCCGAAAACACGTTGATCGTGTGCGACGAATTTCACGAGGCATCAGTCGAGATGATCGATCTGATAGACGAAATAATGAAAGTACGAAATGGCACGAGCGTAGGTTTTAAAAATCAGAAAATCCCAATGGCATTCCCTATTTTATTCTTATCGGCTACGCCGGTCCCACTCTCATTAATTTCTCACGCACCCATAATCAGGCCCGACATTCCAATGCGTAACAGGCGGACAACTCACATTCGTGATGGTGCCGATGTCATGGACAACATCTTATGGGCAAGGGAAAATTTCCCCGAATATGCAGACTCAATTCTAGCTGTCGTTCCTACAGTAGAAGAGTGCATGAATATTTCCCAGCACATGGCTTCCCTTGGCATCACTACGGATGTGTGCCACCGTAATGCACGAAATCCCGATCCGACCGTGTCCATCATAGTCGCTACCTCAATTGTCAACGCTGGCATAACGCTCCCAGGCCGCGACCTCGTGGTCAGCTCAGGCAAGCGTATAGCCATACATAGGGGCCAGTTTGTCCATCCGTTCCCGGACACAGACCCTGACTTTGAGACGCAACTAGATGGTAGGGTTGGTCGAGGCAACCGCGCGGGCATAATTGTTAAGCCCTCTAATGCGGGCATGGGCGAGAAGCCGATCGAATATGGAGCAGGTTTATTCTACCAAAAGAAAGTGATTGCTGACTTCCACCGTCTGCCCCAATTGGAGACGGTGCAAGATGCCTACTTTAAAGAAGAACCATGTTTCAGTGTAGATCGGTCACTACCCAACCCTATCCGCAAAGGCTTAGCATTTATCCACCTATGCCAACTGATGGGAATCAGCCAGACCACACACGGTGATAACCCAACATGGCGCCAAGTATATCGCATGAAGTTCATCAAGAAAGAACCTTTGCCAGAAGAGTATGAGTACATAGACAGGGTGATGTCAAACAAGGACACATCCCCCATAGCAGGGTTTGACCTAATCTTGCAGTATCTTGGGTCTGGTCACGTACATTACAAAATCAATGGTAAACGCGATAGCAGAGGTGTGATAGTACCGCTAGGTGGCACTTGGCAAACTCCTACGCCGCCGGAGCACAAATATGTCATTCATGAGACTACGACGGAAGCACAAGGCTACGAAGAGTTGATGAAGGCTAAAGAGCGCAAAATCAAAAGCCATTTAGCTAAGATCCTCAACGCCGCTATGAATAGCGTCAAAAGCGACCTGAAGCCCCACCAAGCGCGCAAGGCATTGGAGAGGGTAACGAAGGAGATAGGACGAGCTAAACAAGAGCTCGTCACTCCAAGCGACGCAAGCTTAGATGCTAGAGAGCACCACGGCAAACCTCATGAATTCAAGGCAAAACAAAAACCAAAAACAGTTGCAGAAAGCCCCAAAACTCAGGAATCACTAGGTGGTTCGATAGGCTCGAGATCTCTTTCCCCTCTCGCGGAACCATTCACTCCTTCAACGCCGACAGGTTCCAATTACGAAGTAACGACTCCCATGATCACTGAGGCAAACCTCCTTAATGACTCTCCTTTCACTTTGGATTCCTATTACTAAAACACAAAATGCCGTCCATGGA

> SrMYV1

TGCTGCAACAATTTTCAACACACTGGACTTACCAGTGTGAACCTATAAAAGGCTGGGGATTCCTTAATCCAGAACCGTCCAGAGAACTTTTCGTTTGAGGTACGAAAGACGATGCGAGCCGAAAGCGTCACTTAGAACCTTGTCCGTTAGATTGATTTTGATAACCCAGAAAAAACGTGCCACGGGTAACAACACGCTAAACAACTGGAATCTGCTTTACAGTCAAAGCAGCAAAGACCAGCGACCTAACACCTTTAAGAAAGACCGACTTGTGCTGGGGAACCGCCTACTCAGCTTTTCACCGGAACCCGAAGCCTTAGCGATTTTACGAATCGAACGCGCTACTGAACGATAGTGCTCTTTTCCAACTGCGTATGAGCATAATGACGCATGCCCCAAAGCGTCAATCTCGTATCGAAACAAATGACTCGAGACGGGTTATCACCAAAGTGACACTACCACTTTTGCCTTAGATGTCCCAACAGAGAAAGATAGCGTGTAAGGGGCGGCTACCACCACAGGATATAGCAGCCGCAGGTGTTCGTCGCCGGGAGACGCGCCTTGCTGAGGACCGAACGACGAAATAAAGTAGGACCTGACTCGTCTCGCAATGACTGTTTTCAGATGCCGTGGACTTTTGACGAGTCTCGCGCGTATGGTCCGAACGCGGGGATCGAAAGGAGCCACACGTATCTTGCATTGGTCTTACGCATACACGTAATACTACCATCTATTGGTCTTACGCATGGAGATCACACGTAACACCGCATGTTGCCGAGGCGTAACTCTCGGTGCAAGGCGTGGGTGAGTGGCCGGCCGAAGCCTCATCAGTCTCGCTGCGCTAAGGCAAACGACGGACTGGTGTGAGAAAGGGAGGAGAGAGGGATCCTGAGTAACGACAGACTACGGTAGGAAGGAATGTAAGGGTGGGGCTTAGGAACGAATCGAAAGCCCGCGGTGTGGAAACTTGGAGGAAAGAATAGGACTTAGGACGAATCGAAAGCCCAGGGTGTGGAGTTACTCACTACCTCCCCGCTACTACACCGCCAAGGATGTTGGATCGTTCCTGCCTCAGGCAAGGATCGGTATGGCATCTCCTAGCGGTCGCGGTACATCGGTCAGAGCATGGCTCGTCTCGGATACCTCCGTTCTCGTCGCTACTTACGGACCCGAAGGCAGTAATCCCGAATTCCGGAATTACGTCGGAGGGATCGGTGGTAAGTGCGAGACTGGAGAATCCTTAACGGATGCTCTCGTCCGCGAAGTCAAGGAAGAGGTCGGAATTGACGTCTCATCATGCAATGTTGAGATATCGTCAGTCGAATCTCATCCCGATTTCACCGTCGTCTGGGCCATTGTGTACTGCGGAGAAGAGAAAGATATCTCAGTTCCCGAGTCAGAGAAGTCGAAAGTAGTCGACCCTCACTGGGTTCCTTTCTCAGAGATATCAGCACTCGAAGTTCCTGGCATCATGCATCCTGATACGTTGACAGCCTTCCAGGCTGCTAAACGTCTCTTTATGCAGGGTGCGTTGTACACTGCGACACGTGGTTCAGTGCCTTCGGCAACCTGGTTGCGCCGTGACGACCCGGATCAACTACCTGGCCCCGAGTGGAACCTGATCATGATTAACAACAAGGATGGCCTCTGTGTACCATACGCTATCCAAGCTGTGTCGGGTCAAACGTTCGACTTCAACGCGTTGGCAGCTGCTTTGCCTGATGCCGGATACGACTACCGTACACTCGTCGACGACCAAAGCCTTTGGGCAATGTGGTCTGCTGACGAACGTGCGTGGGTTGTCGGTAACCCGACGACGGCAAAATATTTCCTGTACCACAAGCGCAATGGCCATACTGCTCACGTAGATGGTTTGATACGCAGTACGGACCCCGCACAACAGTACGACCGCGTGTTCTATCCACAGGGCCGCAACTCTGAATTCTACCCTGTCCTCGAAGAGTTTAATGCATACGCGATGAGCGCAATCAAGGGTCTCACCGACCCAGGCGACCGTCAGCGTATCTACGGACAAGAGGTTGAAAAACTAATGGCCATGCCTGACCAAGACCGTCACAACTGGTTGGAGCGATGGTACAAAGTTGCCGTTCCTAGTGACTCAGCCGAACACACACCGGAACCTGTGCCTGCCACTTCTGTCCAGCAGCCAGCCGAGATTAATGGAGTCACCTCGCCACCTCAACAGTCCCAAGCTGCTCCTAGTTTCGGACTTACACTAACGCAGCCGGTCGTCTCCAGCCTTAACTATTCGGGTATGGCTGCACAAATAGGTAATGACGGCCCTCTGCGCCTCACAGGTTCATTGGACACCGTGGCAATTACTGCCGACCGTCGTGCCCAGGTCATTGATATAAATGACCCTGAGCTTATATTGCGGGCACCTGAGATTCAGCATGCCGTGTCCTTTGCAAGCCCACTCGATGGCCTGAACAACATGGGAAGACTGCTCATGCCTGACACTCTCCCGTATGGTTCGAACTATACGTATGACAAAGGATCCGGTGCGGGTACAATCTCGTTTGCTTTCAACGACGACAGCACCAGTTTCCGCGGTGTCGAGTATTGGGCCGCAACGTCAGGATCCGGTAATAAACATTCCCAGGTCCTTGAACCGAACACGATACCCATGGACCACTCTGACCGGTTCCACACTACCGTTAAAGAGTTCGGTGACATATACATGGGATCGCTCGCAACGCCAATGGCACGACAACGGTTTGCGCAGTGGGTGACTACCACCGATGACGCGCCTGCAGCTAGTATGGCTGAACACTGGGAACGTCTGTGGTCAGGATTGTTTGCAGCCGCTAGCGGCGCAACCGTACGACAAGGCCGCGTCCGGAATGTCCAAGGCCATGGTGGAGGCATCGTTGATCAACACTTCAATGCTCTCTTCCCTAATAGTACAGGCTGGGCCGATATTACTAACACCCTCCCCCCGGCTTCGAACCCAGTACCAGGCTGGCCTCATTTCTCGGTAGACTACGGGTATTCCGCAATGACCGTGGTGCCTCTTAGAGCATTCGGTACTGCGAAAAGACCTAGAGTCTACAACCGGACAGGTCTACACCGGGACTATCGAGTCGTCCTTTACCACTTGGGTAGCGAATATCTCGATATGGACTCCAACGTAGATATGCTCGCGCCTGGACTCGCTCGTACAAGGGCGAACTTGAGCCAAGGTTCCTCATTCTTGGCACGGTTTGACGCTGAGCTCCCGCCGCTGGTCTTCCTGACCGAGACATTGGACATATCGCGTAACAACGAATCTCGTCCAGGAGCCGATGATGGTGTTGGGTACAACATCTTAGCCCTGGAAAATGCCGATAGCTGGCGAGACGCGATTACTTTCTTGCTCCAGTTTATCGGTGGGTCAGCGACTTGCGTCCTGGGCTTAGAGCGTACTGTCAGGCGCTCGTTCTACTTCTTCCCAAGTGAATCGAGCCACCTGCCTACTACCCTAGCCGAACGTTTTTCCTATGGGCAAGAGTTCCACGAAGTATTGGCTAAACGTTTGCTTTACCTCAGGATTGTGCGTCCTAACTACTCCAACCAGGATGATCAAGCCGTACGTGAAAGTCGCCCAACAGCGATTACCACACGGTTGCTCAACTCGGCAACATATGCTGGTTCCAACCTCACCCGTGCTACACTCGCTGCTGGGCCAAATACGGCTGGCTGGGCGACATTCCAACACATCACGGGACTCGCCGCGCAACCCGCCAACTTACCTGCCGCAACCTGGTCGAACTTGCTACTCGATGATGAATCGCTGTTCGTACACGCAGGCTGGTCACAATATTTCGCTGGAGTTGCCCTAGAAGGTGATGGACAACCTGAGCTTACCAAATTCGTCGCTCAGATGCCCGAAACCGATCAACAGAGCTTGCTGGAATGGTTGGCTTCAAGTTTCGGCGGATTACCTGCGACAGTCCCTTTTCCGGCTGCTTGGGGTTCCGATCATGTGACGGTCCGGAACAAGGCAAATTCGCGGAATATGACAGAAGGTGATATCGCTGAAATCATCGTTCGCGGAGGCCTTCTCAGACAAGACCGCCTGCTGCCTCTCACTAACTTGACAGAGCAGTATTCAGTCATTGTCCCTTCCGGTGTGAGTGCCGTACGTGCTACCAAGATTAACATCAACACGTACCACTCTGACACTGTTGCTCTCGAACTGGCTCGCCGCCTCGCTGATACTGATGCACTCCCCATAACCTCACTTTTCCATCGTGCTCTCTTGATGGCGGTGCAGATGCGTGCAGGAGTTGATGCTGTCGCAGCAGAAACTAAGCTAACTTCTTCGGCATTGCATCTGCGACAAGGACAAGCAATGACTGGGGTTACGCAAATCGACAACGTACTCCACGATACTAATGAGTACGTCCAGTTTGGAGACATGACTTCGTACCTCGAGGCCTATATCCAAGGTGTCGGGCAATCAATGCTCAATGCAGGTTACCCTTCTACCTTCAAAACAGAAGTCGGTGTCACTATCCGCGTCAACTGGCCCAACCTGTCCACTTGGCAGTACACGCGCGAGTACAGCCCGAGGCGCGGTCAAGAGCTCAATGCACCTGATGCGACGTTACTGGTATGGCGCTCATTCCATTACTTGACGGTTAATTTCTTTAGACCTGACGTCAGTGTACCCGGTTCTTTAGTCGCGAAACGTCTAAATATATTTGGGTACTTCACACCTGGTGCGACTACGCCTTGGGATGACTGGTATATCGATACGTCAAGGGTGCGGCCTCTCGACGCTTACGGGGCAATGCGTATCCTCGCTTCCCTTAATGGTATCGTCTTCACTGCTACGCCGACTATTAAGTTTGTTAATACGCGTGGTAACAGGAGGCCGACTGATTACCCAATCCTCCAGTCGGTTGCTAGCCGAGTGCCTCGTTACGGTGTATTTGGCAAGTTGACTCCTGCGTATAAGGTTGAGCCTCTCTGGTATTACGACTCGTCTCTTGCGCCGCGTTTACAAGTCACTGAGGACGCGGTCAACCAGACCACATTCATGGCAGAAGCACCAAATTTCGAGTACTTGACCAACGGTATCGTTGACGATTCACCACCAATGTTTGCGTACCCGATCGGCCAAGTGACGCGCTTCGTCGGCAGCGACGCAGTAAATGAAAAACCAGTCGCAGGTTTCGGTGTGGGAGGATATTTAAGAAATGTGCGCTCGATTGTCCCAGGACAAGATTATGCTGACGAGACCACTCAGACCCTTGCCTCAACTGGTCTGCCCACGCCGTATGCCGATCACACCTATACATTCTTCAGCAGACAGCAGAGTGCGAACATCAGCTATCAGAGAAGCTACCCGTCCGCTGTTAATAGTACGGAACGCTATCGCAAATTCCGAGGACCGGTCACTGGTGCTGATACTAAAGAAATCCCCACGGGATACTACTCTGCCGGTACGCACACGGCTGATTTCTCTACGATGCGTGAAGATGCGCTCTTCCACTATGTTTCCTTCGGCGACATCAAACTGTCCGCTGCAAATGACTCGCAGCTCAAATATTTCTATAGTGTTGTGAACCGTTGGCTCGTACGGTTAGAGCAATGGTATTTGAAGCCTAGCATATACATGCCACCCGTTTATTTGAGTGGTAAGTATGAGGCTCCAATATACCGTATTGCTGACCTTGAAGGGACTATCTCTGATATGCGTGCACGCTTCGTCCCAAGTGAGGGGCTATCCAGGGCAATCGGTGGTTTCAACGCTGAATTTGCGGCTCCTAAAACATCAGAGTCGACAATCCTCAATTTTGACCCTGAGATAATTTCTGTCCCCAAGCGTCACGGCGACCCGGGCCACTTCTTGACGGCATTCGGCGCAACGCTCGGACCACTCGATGCAGGGGGAGAGGAATATAACAGAAAAATAAGAGAGAATGATTACCGTCGTGCTCTCAAAAAATTGACCGACGAGATGGCCGAACAGGGTTTTTCCTAAGGCCCGATAGTTACCAAGCGCGCGGTAATAGACTTTACCGCTTCAGCAATTCACGGGTGCCTACCCCTAGTGTGGATGAAGGTGCTCGCATTTATAATATAGTACATAAATATTTGCCTAACCACAAAGTTTTAACTGGAGGTCTTCCTTCAAGTAGACGAGCTGGTCTCGATTATTTATTGCCACCTGCCCTGAGGAGCGGCAACGAAACGGCTGGTCCGTTGGCAGGTAGTACGCTGGACGTACGTGCTCGAGGCGGTCCTCGACTCACGGTCACGAAGACTGAGGTCGGTGTTACTGAGGTAGGAGAAAATGTTCACGGTCCCAAGAATGAGGATAGAGCCCTGTCCCCGCATGAGACTATTACACGTTCTCTTTCCGGTGTTTCGCTGATCGGAAGTAGTCGTGGTAGTGATGACGGTTCTGCTTCGTCCCCACGGCCGGGTGGTGTTACCACCTGCCAACCCCCCTCCCCTACTGGATTATCGAGGCAGGCAGTCAAAGATAACTATGTGGGCGGTGTCGATGAAACTACCCAAGCTGCGGATGTGACATCGGCACGTATCGGCAAGCAAGCTGTGACACCGGAAGTATCTGATAACGAACAACCTCCAAGTGGTATCAGATGGGAACCCTTACGCAGACAAGGGGCAAAGTTCGTCCTGCTCATCCCGAGTGGAGAAGGTAAAACATATTTGGCCGAGACGTATCCTGAGATCTTCACCGATCACGATGCCCTCGAGGATTACACAATTGAGGTCCAACCGTACATTGAGCAGGCACGCGAAAGTGGCGACTGGTCATACATCGATGCGGTCCACCGCCGGAGTAGCGCCAAGTGCCAAACACCTGTACTACTATCGTGGAACGTTTCGACATACGATCATACCCGCTTCGTGAGTCTAGGTGCGTATTTATTGCGCCAACCTACTAACATTCGTGAAAACGAGAATAACCGTGCCGCTCTGATCGCAACCCACGATTATTGTATGTCGGACAGCTTCTATGCTCGCGATTTACTGATTGCGAAGGCACTGAAGACGGGGAGGCCAGCATATCAAACACACACAGATCTTTTGCCAGTCGCCGGAGTATTTTCAGTAAAGGCACAATCCCTATTCGAACCTCTGCTGCGCGTCGCGACTACGCTGGGACAACAACCACAGCTCCTCAATCGCTACGCATACGGGGTACTTCGCGATTACTACCCACAGTTCTTAAATGCAGCATTACGTCAGCAAGTCCCTGTACAAGATGTCCACCATTTGCACCAACTTGCATTTTCAGGCCAGATACCTGACGCTGTCTACAAGCTATACAACCGCGTCAACGACGCATACATACCGGAATGCGTCGAGTTGCGCGATCAGCTGCACAAACAGCACCATAATGGCGGAGCATACCTCGGTCTACATATCTACGATGAAGAGACCGAATTCAGACCGAATCAAGGCAAGAGGCGGACGCACGAATACTCTTCAATATCATTCGCGACCTTGGCCGAAATGTCGAGACATGAACCGGTCGTGCTCGTAGGCCCTCCAGCCTGTGCGCAATGGGTCATGGGTGCTCCAGTCCCTAGATATCGACCTGGATCAGCCCAGAATGGTGTCTTCTGTACACCAGAAATACCCCTTGATGAAGAGAAGAAAGCTGTAGTAGGTTTCACTTTCTACCTGCTTCGCTCGTTCCATCGACGCGAAGGTGTCCCTGGAGCACGCGCAACGAGGATGGCTAAGGCAGAGATAGCTTCCCAAGGTAAACTGCCCCGCTTTGGTATGCAAAAGGAAACAACAGGTATACCTTATAAGTACCGGAAATGGGGATTCGCACAACGGCAGCGACGCCAGGTTAAGCGTGAACTAGGGAGTATTCCTGGCCTATCAGCTTTAGCCGATGAAATCACCGACGATCAAACACATCCGCGTCTGAAACGTACAGAACGACTCCAACAACTTCGTGACATGGACAAGCACCATATTCGAAATTGGCGAGTAGGCGAGGAACAAGCCCGTTGGCGTTCGGACTCGGTCCGCTACCTCGTGTTGTGCCTTGAACAGGGTGACCTCGCTACCGCGCTAGATCCATTCAACTTCCTGTTCCCATTCCAAGACCAAGAATTGTTGGACAGGACTCGGATGCAGCCAGACTTGGTCGTTGCCGTTCTCTCCAAAGTTCAGCAGACGTACGGTTCCGAATATCTTCATGACTTGGTTATCGGGACTGGAGGATTAGGCTTACAAGGTATCGCAGGTTGGGCTCTCTACTGCTTAGCTAGCTATCACGCTGCAACTTGGACTCTGTCGTTATGCCGGAAGGGTATTCTGTGTAATGGAGGCAAAAGTGCTTCTAAGATCCTGAAAAATATCCACACGGTCGTTCGCAGGATAGATGTGATACCCGCCGCTTACGGTGACTTGTGCTTACCAAGCTACTGGAATAAGAAGGACTTCGACAAAGTGCCTAAAGATTTCACCCTCTACACTAATCTACTCATCGGACGTGCGCAGGATGAGATTCTAGGCTATGACGGTTACGTGGAAAAGAGAAACACCGCGCAACCAGTACAACTAGCTGCGTCGCAGAAGCCAGATCCTAAAGAAAGGATGACAGAGTGGGACAGTTACATGGATAGGGCATTGCAACGCTTGGCCGAACCTGTTGCTGAAGCGCTAGCTTCGACGCGCGACACAACGGGTACTGGTGATTTATCACCTTCCGGCTTCCTGGCTCACTTTGTTAACATCGCCCCTCGCGGGTCAATAGGTATAGGGAAGTTCGACTTGAAACGGTTTGGAATACCTCTACACAATGCACACAAGCGTTTGTGGTTGGATGCCCTGACGCCAGACGACTTGACTTCTGTACTCGACAGACCAGCCGAGGTCTTTACAAATGCTCAGGTGAAGACGGAAAGCGGTCTACGCTTACGTCAGATTATCCCTGGAGAGATCCATCAGTGGCTGATCGAGTCTATAGCTATGTTCAAGACCGAAGGAGCTATCTTTAGGGCAATCCCCGACTTTTCACTTGGGACATCCGTTGGCAACACTATGCTTGCAGATCTCAAGAGATGGTGGCGTACGCGTACCGGGAGATGGACAATGGCTACTGATTACGCCGATTTCAATTACTTACATACCATCAAGGATATGAAAAAGTTTTGGAAAATAGTGATACTCGAACCCGCACTCACGCTGGCTGGACCAGGGGACTGGGATGGATCCAACTACGCAGGGTTTGTTGCACGCTGTGCTGAATGGCAGATTAGAGCGCTCGACGCCCTGTATGTCCGCGAAGTAGGATCGGACGGTAAATATAAGCATGTGACTCGGTCATTGTGGTCTGGTTGGCGGACGACGACGATGATCAACAATGCAATGAACTACTGTTATAACCAGATAAATCGTGAGATATTCGCAAATGAATGCGGGTTCGACCCAATCAAATCTGGGAATGTAAACGGTGATGATGGTGATTTCGAAGTATTGGGTGTTGTTGAAGGTCTCTTATACTTGAGGCATCTCGACATCGAGGAATTAGATGTCCAATCGTCCAAACAGATGCTATCACAAGAGAAGGCCGAGTATCTACGCATAACTTACCGAGAGGGAGGTATTATCGGTAGTCTGGCTAGATCTGTCGCTTCGTTCATCGGGGGTGATCTGCAAGAGCCTGTGATCGATGCGGGCCCAGATTATGTACGCGGTACCTCATCTGCGATCAACGTACTGATACGCAGAGGTTACGACTTAGGCACCGCCGAATGGCTCAGAGACACAGTGTGTGGCTTCTACGCAACTATGAAGACCCAACTGCTTGACGGTTCGACTCGAATTGTTAAACTACAGGATACTCGGAAGTTATATGTACCGTACGCCGAAGGTGGTTATGGATTATCTAGATATAACAACTACTCTTCAGTGAAATTAGCGACCACAAAGAAATGGCCGGTAATGTCAGACAAGTGGTCGCTAGACAAGTTCCCACATCACGGTGCAGCTGCCGCTGTGCGAGCCGCTGTGGCCCGCTTCGACCGTTACGGGATGAAGCTCAGCGACCCAAGTACCCTATACCATGCGTACACTTCCCTTGCTTCTCAAGGTGTAGACACAGCGTTTAACAAATGGTGGCAGGACATAGACCGTACACGATTATGCGACCATATCGAGTGGCTGAATAAACAGACAGTCGCTGCAAGACCGTCCGAACCAATAATAGTCGACAAACAGATCCAAATTGAGGTTGAGAGGACCATCGACTCTTTCCTTGAACGAGATGCGCACCTGTTCAATAGTAACAAAGATCTAGCTCCGAGTTTAATGCAAGCACAGACGGAATTCGTCAGTCGTATTCTTGGGCTGGCTTCAGTGTCACCGCGCTCGCTACACCTGTTGGTGGATAGTGCAACAGGCGAACGGTTGTCGATCGAGGAGATCTCGACCAGGATGGGGGACACTTCTTCAGAAAGATCTAAGTTCATGGGATTCTTCCCTCACGACCTGATCGAGTTAGTATTGGATTATAACTTCGAACTACCTAAACCCACATCAGGCGTAATACCCGACTATCTCCTTCCGTTGGCAGACAAAGTACTCTCCGATGTGACTAGGTTACATCCTTGCCAATACGATGGGATACAGTCTAAGCGTGAATATTACGCTGGACTAACACTATGTACAATTACACATATGCAACGTTACATGTATGCAAAATACAGA

> SrOLV1

GCCCCTCCTTCCCTCTCCTCTGCCTCACCAACCTGGTTGGGTGCTTTATGGGCTTTGGACGACACCGGACCCTGGCTATGATCAAAGCCGGGGTCATCAAGATTAACGGTGATGATATCGTCTTTCGCTGCACCGAAACGGAACGGCAACAATGGCTCTCAATTCTGCCACAGTGCGGACTGGTACTTGAGCACCAGAAGACCCTTGTTCACAGGTCTGTGTTCACGTTGAATTCAACCTTCTTTATGGCTCGCGCGTCACGCTTGCCTAGGTGGGTTTGGTTCTTCCGTGCCTCCTCCGTTCTTGCCGCCGCGGTCCCTCAGCGCAAGGTCCCCCTCCCGATCCGGTCCGGCCGGCACACCGCCTCCTTCCTCTCGACCCTCGCGGACAACACTCGAAACCTCACAACTCCAGGGCGTGCACGTTTGTGCTAAGCCCCGGGA

> SrRV1

CTGGACCGAAGCTGATCCATTCACTGATGAGATCAATCCGGATGCTGTACCACCCGCTAATGTCGGGACGGTTGCCGCCGTGCCTCACGCCTTCGAAGTCATCCATTTATCAGCTGTCTCGCACCAGTTGGCCAACGTTTACCAGGACCCTGAAAGTCCGTTATGGAGTAATCAAGCTGCAGCAGTCGATGAGATTGCGAATGCTCAGGCTACATTCGTGGATGCTGACGGTCTCTCGGAGGATGAGATTGCTTTGGTCATTAAGGCCTTTGCACCCCAGGCTAAGGAATACATGCCCGCCGTTACTGTTGGTCAAGGCGAAGGTGCTGTTAAGCACTACGCCTGTCCTGCCGCTTACGGCTCGCTGGCTTCACGGCTTGGTGAGACCCCTGGCACTACTAAGATGTACTTACATCACGGTGGTTCGCCAGTCCCTCTTAACCAGGTTAATCCTAACCCTGCTCCGGCCAATCCTTTCTCTGACTGGGAGGCACGCGTGCTGGGAAGGGGCGCCGGTAACTGGGACAACTCGCCTTGGAAGCGAACGTTTAGGTCCAGTACCATCAAGAAGGTTATCAACCACTTCATCGCCAAGCATCACGCTGGCGCCGACGCTGAGGCCGCTCTTGATGCATTACTGTACAGAACGTTCTTGTTCAAGATGAGTCAATTACCCAACGCTAGGGAGAATCAACCCTGGCCTTGGGCAACTCCTATGGGACATTCAAGATTACAGCTCCCTCAGGATTACACGTTGTCAGCATACTATGACGCCTTCCGTAACGCTGACACTCCATCTACTACTGCGCCGAGATTGAGCACGTTCATGAATTCAACTAACCGCCACATCATCTGGACTGGTTACCAAGCGTGCTTCTCTATGGCGGCGTCAATTACGTGGCCTGCTCACTTGTTATCAATGACAGGTCAGGTAATCGCAGCAATCAATTCTGGTCTTGGCAACCAGAATAATCAGTTCATTGCCAACCTCGGCTATGCCTTAACAGAAGTACTCCGGTCTACTGACCTCTCTGTTTGGCAAATTTACCATAGGTATGCATGTGCACTAATGTATGATTTCGCTCCCACTGAGGAAACCATGCTGACTCATGCACCGCAGCCGATGAACGGTTGGAATGATAATACAGCCCCGTTCTTCACTAACGCCTACCATGAATTATGGTCGGGCCAAATCTTCCCACGCTTGTTTGAGTTGCCCTCGACTCAGCAGGGAATACTCTGGCCAGAAGGTGAAGATCGCCCGGTCACTACGGTCGAACCTCCAACCAATGGAGTTCGAGTTGCTCGTGACCTCCCGGCATTTACGGGACGCGCCTTCCTTCAAGACGGCGGATCTACAGCAAATCTACAGTATTACTATGCTGTAGGTTTGGATGGGCACTACAGGTATGAGCCTGCTAGTACTAGAGGCGATATCCATGAGCACCTCCAATTGGGCACATGGAATACGCCATTCCAACAGGAGTGGCCAGCTAATCCCTCGACTTTCGCTTCCGTCGCGATGGGCGCTCCAGGTACTCCTTGGGCTGATTTCCTGCTGCCAGGTTCCCTAATGACCTATAATCTAGGTCAAAACAGGATCAGGGCACTAGGTACTCGCCTGAATCCAGAGAGTACTCTTACCGCGAGCCAACAACGCGCACTGGGGCGTGATTGGTATGAATTGTCTGCATCCGGTGGTGACAAGAATGTTGTGAGATCGCTGTGTATATCATATATACCACCGTTCCCCGTCAAATTCGAGACCAACTACCCCTCGGATTACTCAATGGCAGTCTGGGCTGATGTGAATACCAATGCCTTCACCGGCGTTAGTTTCTGGACAGCCAAGCCTAAGGAGAACATGTTCCCTAATACTCCTACAGCTACCGATTCGGCTGCGCCATACCTCGCTAGCCGCCCGAGCTTCCCGTCCTCCTTCATGAATGCCAACCCACCTGCGGTTCATCAGAAACCAGCTCACCCAATCCCTCCAGCTAGTTCTGTTGGCCAGCGTAACAAGCCACGCACTGGGCCACAAATGCAGATGCTTAAGAACCGGAAGCACGAGGCACTCATAGAAGCGCAGAAGAGTCAATGGCTGGCGAGCCTCCAAGAACAGGCTCGCATTAAGGCTGCGGCACAGGAGCAAGCGAACCAAGAACTACAGAGGATGCTTGCTCAACAACAATTTACTGATGCCATGCCAAGCTATGACGCTGTGCGTTATGAGAGGCCAGCGCCCAATCGGCCCCATGTATTCCCCGCCCACAATCCCGATCCTCGTGGCGATACGCAAGAATCGGTTATTCCTGTTTTCGATGCTAACAATGGCAACGCCTTTGCCGCATTAGCTGATGAAACCAGGAGTGCTGCTAGTACCACTCGAGACTCTAGTGGCGGACCCACCCCTCGGTCTGCTTCAACTGGGCCATCCGGTAACAAACACAAGAAAGTTAGTTTCCAAGATGGACCGCGCAAGAAGCTTGAGGTTTCTAGAGATGGTGTTACGACCATGATTGATTCCAAGAACCCCGAGAGTGTCGGTGACATCGCCAAGGCTTATCGGTTACATGAAGGAAGAGTTGACGTTAAACACGACGACTCGACCGTTGAGCTACAGCCTGGCCCCTTTGTCGAACCAACCTTTGGGATGTCTCGTCCAATGCCTGGGCTCTATACTGGACCTGTTCCTCCAGTTGGGCCTGTAGAGGTTAATGACGCAGCCGCTTCCAAGGTACAGGTTGGTTTCGAAAGTTTCGTTGACACAGGTCACGGAGCTAGTGATCCGCAGAGTCGGATACACGATCTACGAGTAACTCCAATAGGAGATACTCGACCCACTTCTTCCGCTAGAAAGCCGGAAAATTAATGTGGAGTGCTGCTTATGGGGGAACAGCCACCCAGCGCTCCGCTAACATCATAAGACTTATCTACGCTCAAAAGTATCAGTTCTCAGAGCTTGGACGGAATTTCCTCCACTCTACGAGACACCCGAGTCCAATGGACTACTTACTGATTTTGATTAATCAGTATTTCTCTATTAATTTGAGCGTAGAAGATGTCTTGACTGAACGCATCCCCGATCGACGATTTGGAGACTTTGTTGCTGCTAACGCCGAGTATCGTTTCGAAGAAAAACCCACAACGGTGGAGTCCGGGTTGATGCGTTTCATATGGTTAGAACCTACACCTAGCGAAACTTTCGAATTTATACCCCAAGAACTATTTATTTTATCATACTTTAAGAAAACAACCGCTCTGTATAAAGCATTTGAACGTGGCGAGCTTCGCGAAGCTGCTCAAAAATTCAAGGAATTAATTAAGTTACCAGTAACTCCAACCTCTAGGTGGGAGTATGCTGCTTTGAGCCTGTCCCTGGAAGCTAAATACCCACTGGTCCCAGGCCGTGCGGGAGCTAAGTCATTCCTAGGGCTTAGAAACGCTATCGAAATCGCCAAAACTAACGCATACTCTAGACAGATCGTTAATAAGCTTAATACTGTCAATAATGACTGGGAAGCTACTTTCGCTACAGCCTTAATCTTATTACCTATGGCTTCAGCCCAGGGCGGGAGAATCGCTTCCGTACTGTTATCTTTATCTGACGCTGCCTTTTGGGAAGCCGACTTTTTAGATGTTGTCAATATCTTGAAGGACATACACACTATGTCCCGTGTCGCCAGATATTTATTGTTAGACGATGAGTTTAAGACAGCGGAACAAGCAAGACACATCTACGGTTTGGATAGTATTGTCGGCCGCAATGAACGCTTCAATAATGACATAGATGAGGAAATAATCATGCGTATTGGTGATCCAGTGCACCATGCTGTGCCCGATCTTATAAACCCAACTCCTGGACATTTGAACTACGATGATGTCCGGAGCGGAGTATTATTGCGTGAAGGACAGCGCGATGCTCTTGAAACTGCCTTGAGACCACAAGTGCGTATACTGACATTTTCGGATTGGTATGCCGACCGCATGGCTTGGGCCGCTTCGGGAGGCGCCCCTGGAGCCAAAGTCCAGTGGGAGCCTCAAGCCAAGGCCGACAGATTGAATAAACGAGGTGCGTTGCTATTAATTCCAGAGAGCCACATACTAAATATCCTACGCCAAACGTTAAAGCCTTCACTATTTAGCAAAGCAGCCAAGAAATTCGAGAACGGTAAAATACGTAGCATTTGGAATACTAGTATATCGTTGTATGTTATCCAAGCTTACGTATTACATCATTTCGAGCGCGCTTGCGCACCCGGTACTTGGAATACATCGGCAGATAACACGTTCTCAAAGATGAGGGGAGACATCAAACGTTTGCTTGGTCTAAGCAAGCATGGCAACCATGGGTTGATGTGGGATTATTCTGACTTCAACATCAATCACACAGCTGAGTCGATGGTCTCGCTCTGGGAAGAGACGTTGAACGTTATGCTACCGAGGCTTGTCGCCGACACACCTGATGAACTTGAACAATGTCGTTTGGACTTAGAACTCTGCGTCGCGTATATCGTGAATGCCAAGAGCAACACTTTCTTGTATAACCCGGAAACAGAATTTGGCAGCTATGTTTGTCGCAGTCTACAGAGTGGAGAACGCGCAACTTCTTTCGTTAATACTTTCAATTCACGCGCTTATGCTTACATACACGATCGGGTATCAATGCGGTTATTCAATAGGACACTATTACTTCCACAACTAGCTGCGCATCAGGGAGATGACGTCTTCAGAATCGTGGCTTCAATTAGTGATGGTGTCCTGGCGTGTGCCTTATTTAATTTGTTGGGATATGCTGGGCAAGTATTTAAGATCATGTTAGAATACAACCCTCGCGGTGAATTCCTGCGCCTCCACTACGACGGAGTAGCCAACATAATCGCTGGTTACCCAGTTAGATCGTACATGGGTTTAATAACTGGAGAGTTTTTCCGCGAGAACGTCATCGATCCAGGTGACCGTGCCATGGCATTTTGCGACCAGTTTGGGAAAGTACTTCGCCGTGGGGCAGTGTTGCCACAGCGAGTGTTAGATGTATTGCTCCGCGATCACACCGCCTTAACTTATACTCATGATGGAGTCCAAAGACGTGTTACACCTGATTTGACGATGTTGTTCACTCCTTCAGCACTTGGGGGATACGGAGCTAACGCTATTACAGCGCAAGGCACGGTCGCACTAGGTAGCAGCCTCCAAGCCGTACCAGATGTTTCGGGCGACGCTTTGCAACGAGAGACGGCTAGTGTTGGTTACGCGATAGCTATACCGTCGGGTCATGGCAAAACCACTCTCAAGAATAGGTACCCTGAACTGTTTTACGATCAAGATGATTTTACTAACCAACCCAAGGTTGCTAGTTTGATTGCTGATGCTAAACGCAGGGCCAGAGGCGGTGATGCCGGAGGATGGGAAGATTTAGACGCCTATCATAGAACAGCCTCCATCCCTCGTGACAAGGTATTGTTAACCTGGCATGCTGGGACCGTTCCTACCGGTTTCCGTTTGATTGCAGCGTTAATGGTGGACGCCAACCGCGAGGGAGTCGTCTTAAAACGTACTGATGAATATTTCTTCGTTAACATACACCGCCACTCAACATTGAGTATTAATTTGTTTAGCAACCATAATGAATTGGCTAACGAAGCTGTTCGCCTTGCCAAGATCGCCAAGAACAAGGAGTTTGTTCCAGGTTACTCAGTCAGGAGATTCTTAACTTATAACACTCGAGTGAAGGAGCGTCCGGAATACCACTTCCCACCGGTACCTGTTCGCGCCTTTTACGCCGGCACTGCTGGACAGATACCTGATGTAGGAACAGCGATCAGGTATCAACTCGATTTTAGTACTGTGACCAGTATTAAGAGGAGTATTCTTAATTCAGCCTTACCAGGTGCTTATCCAGCTGCCAAGCTGTCTAACACTCTGGCTAAATACGCTAGTGAGTTAGATAAGTGGATGCGGATTAATAAGCCTAGTACCACTATTCATGTTACAGCAATGCCACCTCAACAGGTTTTAGATGAATTCAGCTCCTATGCTGCTATGTTCACTGGAAGTTTATATAGTTTCCAAGGTGATGTTTCTACAGCACAAGGCTTTGCACATGCTGTTAATCTGTATTTCTCCACAATCGCTGACGATACCAATATTTCGTCGGCATATTTATTTGCGCGCCCCAACTACGGTGCATTCCAACATCTTGTCCGGCCAACTGGATGCTCGTCATTCGAGACTCTGGTCAACATTGTTGATGCATCCCCTCTCTTAGCATCTGATACTGGACATGCTGGGAGGTGGCTCAATTTAATTAACTCAACGACGCGCGCCAAAGAATCCAGGGTAGGTATCAGTTATGCTAAATTACATAAACGGATATCGGCTTCAACGTTTACCGCTGAAGAGAAAAGTAATGCTTACAACTTCCTTGTAGATTACTTTAGAGGCAACTTGAGTCTCTACCCACCAACAAAGCGAGTATTATCTCCAGTAATAATCTCACTATCAAGAATTTGTACGCTAATGTTTGTTGAATCGAACTTCATGTACTTTGCACGATTAGATATAATCACTAGATATGATTACCTATGTGTGTTAGAGGAACATGCGATCAGTAGATCTCAACAGCGCTTGTCTAGTATACCTAGTCAAGTTCATAGCACCCCTATTCAGTACATTGATTAGGGGCTTATAGAAGAGCAGTGCAAGGGTTATTTACCCCCT

> SrRV2

ACGTAGCGGCTCCCGACCCCAATGACCCTGAATCATATCTTGATGAGCCAGGCGTACGCGACGCTATACGCGACGGTACCTATGCCCTCCTCGATGTGTCTGGCATGCGTCGTGAACAAGTACGCGAATACGTCAACGCCTTCTGGTTGCAATCCCAGGAGAATAAGTGGGCCGTCGCTATTCGTAACAGTAATAACGACGATGTCCGTCCCATGGTTTCCCCGCTTGGCGTAGAAACCATCCAGGGCGCTCGCCGCCTATACATCCATTGGGGTAGTACGCCAGCTAGCCAGATCGTCCCCCTTCACCAACTCTGTGGTAATCCTGCTCCCGGTGAACCTAACTACAATTTCCGCAATACTCCGCTAAACACTAGCGTTATTGCCGGCACGTTAGAGTCACTGATAATTAGGCACCATGCTGGATCGGACGTAGCTGTGGCGCTAGAACTAGCAATGTATCGATCCAACTTTTTCTTGACTCTCGACATGCCGGCTGGTATGAAGAGAGCCGGACGTGGATCAGAATCGTTCTTGTGCACCGGCGGTCGTGAGCTAAGATTACCGGTTGATTACACGGCAATGCAGTACTTTGATTTCATGTTTTTGGAGTCACCTGTTCCTGATTATGTCCGAGCATGGCTTCAAACCACTTTACAACCCAATGTTATGAAGTGGAACATGTATTATGCGTGCATGCACATGGCACAATCGATCAATTGGCCCTCTGTCACGTTCTCCTTAAAAGGCGAACTTATCCACGCTCTGCATGAACGTGCGAACGGACGCCTCCCGGCCAATGTCTCACCTTACATCGCCTCCCATATTGATCGTTTCGATGCTAATTTCTACATTAAACGCGACGACGTTCTTACAATGTGGCAGGTTGCTCACAAGAACGCTTGTGCTCATCGATTCGGTTACTCCCCGTCCACGACCTTCTTTGAGCTTAGTGCTCCGTACCCACTGGACCTGTGGGCTGACAATCGTCCTGTGTACATCACCCATCCATACTACAACCTTTGGATGGCGTCATCAATCCCGTATCACGCGGTTCTGCCAACGACGAAATCAGTACCACTATGGAGTAAGGAAGAGTTCACCCCCGACGAGGGCATGTACACTCAGTTACTTGATGCTCGTTTCGGTGGGTTCCTAGAGACTCACGAACAAGCTAAGTATCTTAGTGACGATGGGTCCGATTTCTCTTTCGCTCATTTCTTGGGTACCCCTAACTACGTGGCTCAAGTTGGTCACCAAACTCGAGCTGAGCACGCCCAGTGGCAGCCCCAGGTTCAGGGAGTGGTTTGGAATAAACCACGTGACTACGAGACCCCTTCCCCCGTTAATCCTGCATCTACCGTTATTTCATTCTCTTATAACGGTCCCGCAGGTAATGGTAACCCTCTCAGCAATTTTGCTGTTCCTGGAGCGTTCACTAACATTGACTGGGTCAATTGGCGTGCTAGAGCTGGTGGTATGTTTGCGACTAATGCCGTTCACAATAATGCTCCCTTGGCTCAACAGCTCTCGAACGCATGGAAGCGGTTCTACCAGGAAAACCCGGAGCCCCTCATGTCGTTGGGCCTAAAGGTACGTCCCCCAGTATGGAGCGCATCCTTTGTTATGCAGCCAGCGGAGTATTTGATGTACCCACTATTTGAGAAAGCAACTGATCCGTTTGGTGGCTATGCTCTCTTCGATAACGCTGACGGAAGCCAGATCTCTGCCATACGCTCAATGAATATTGCTATGCTCGCACCTGGCCCGATGGCTAACGTTCTACCGTCCGTGCTGCAGAACCACAACCCGAATAGTCAGAGGCGTCCGTACCAACTTAAGGGCCCTGAAGTGTTTGATGCGGTTAAGCCTTTTGTAAGCGAACAACGTAATTCGCACAAGACATACCGTGCACCGAAGATCACTGACCTGCCCCCACCAGCGATGAGTAAGCCTTCATTCTCGTCAATGGAAGTCAGTCTTGACGAACCCGATGTGCGGCACCGAGCACTACCTGGAGCGGCAGAACCTAGAGATTTCAATCGTCGTGCTCCGTCCCCCCTTCCTAGTAGGCCCACACTTCAGTACGACGACTCGAAGAACCCGTACGACGCTTCCATGGTACCTGACGTCCGTGAAACCACTGCCAACTTGATTGGTAGTTCTCAAGAACCGGCTGTCAGGCTAGATCCCCCGAGTTCTAATACTAAACCTTACGCATGGACTGCCCCTCCTGATATGCAGAAGCCAACACGTCCCACCGTCAAGCGATCCAGTATTACGCCCGATGAGAATGGAGTTATTAATCTAACTCAACCACTCAAAGGAGCGGAATGGGCTCGGATGGGTTTAGTTGATGACGAATACCGTTTGAAGTTCCCTGTTATAATCTCGAACAAAAACGGTTACGGTCAGTACAACGTAGGAGATATCCCGTCAAAAGCCCCCTTCTTCGGCGATGCTTACGTTAATGCTCCAAAGCCAAAGGCACGCCAACCTAAGGCCTTCAAGACAGAACCGTACCGCGAATTCGATACTGTTTCAGCGGTTGACCTAGGCGAAAGCAAGCCTAAGAAAGTCGACAAGGCCAACGTTCGGTTTGAACTCCCTGAAGCTAAGCCCAACCGTGCTCCTAACCCTCCGGAACCATATATGAGTTTCGAGGAGTGGAGCACCACTAATACACAACCACAGCCAGGCAGTTCTTTCACGGAAGCTGACTGGCCGAAGGTCAAACTCCCCAATCCCGGCTTACCAGTACCTGATCCCCAATCTGTCCAGGTTATTGGTGGAGCCGAAAATGGCCTGGAGTCCAAGCCCATCAATGATGCCGCTGCATACGCAACCCAAGAAGCGTACGACCAAATGCGTGCTAAGTTCTTTGGCGCTGGACCCCCAGGCTTCCCTGATTCAGGCTTCACCCCTAGTCAGGCAAAAAACTCCTCTGGAGCGTAGGTCTAAGTAGCTCTTTAGTCCCACGTTCCTGTCGCGTAGCTGATGATATCCTGAACGGAGTATTTTCGCGTCACATCTCACGGGTGAATAACCTCATGAGGTGGATTAGAAATAAAATCAGCCACGCCGAAGAGAGCCAGACGCCGTACGTCTACCTTCCAATGGATGAACTCCCGACCGATATACTGAACATAGTCAACCAGGCGCCTTCTACTCAAGAAGAAGCCATTATTACATGGTTGTTAACTTGCCCCACGCCATCTGAAGTATTCTCGTTCTTGCCGAACGCTTATGCCGAGATGACCGCTCTTGTGGACCTGAAGTGGGGCGAATCGCAGTTAAACGGTCTTGCAGAGGATCCTCTGAACGAAAGGGAACGGCAGGCTCTCATAGACAAGTACCCTGCTAACAAGGGCCGTCCTGGAGGTAAACTACACGTCGAATTAGGAGGATTGACCATTGCATTCCGACATAGCGCTTTGAGCAATCAGATCGCTCGCTGGTTGATTAAAATTCCCGTTACCGATTGGGAACTGACACACGCTGCAGCTCTTATAATGTTGCCCCACACGAGTAGGACCGCTGAAAAGGTAACTAAAGAAATCTTTAAGTTACAAGAGATGGGTACGATGTCAACCAAAGATACTACAAAGTTCTACAAAGGTATACATACTCTGATCCGAACAACGCACTCTCTAGGTAATCTGCGCTTGAGCATAGATGAAGCTAACGATGTTTATGGCATTGATGCTCTAGTGGGCCCTAGTGAGCACTTAATCACTAACGCTTACGATGAGATAGTGATGAGAACCCACACTGCTGCCAAGCTCGCTGTCCCAGTCCCTAGTCAAAATGGTGCCCTATCTGAGGAAGCTTATGGCCATTACTTCGACGTAGCCCTTCTAGCCACGATCCGCGAAACACTACCTACCACTGTTAAATGGAAAACCTTTGATGAATGGTATGCTGATCGCTTCGGTTGGGCTGCATCAGGCGGTGCGCCGGGTGCTACGTTGCAGTGGGTTGAAGGAAAAGAACGCATGAATAAGAGAGGTGCTCTATTAGTACTGCAACAACAAGAGGTTCGTCGAGTTCTCATGGAAACTAAAAGGCCAGTTAATTTCTCAAAGGCTGCAGTTAAGTTTGAGAAGGGCAAGACCAGGATGATCTGGAACACTTCTTTATATCACTATTTGTTCCAAGCATACGTTTTAGACTGTTTGGATACTCACACTGTGACTGAACCCACGGCTTTAGAAGGTCCCGTCCCAGCGAGTTGGAATTCTGCAACCCATTCCAATTCGGAACGCCTGGCCGCTCAAGCTTCTCGCTTAGCGCAACTAGGTACTAATGGACAACACGGTCTGATGTGGGACTTCTCAGATTTTAATATCAACCATGATCTCTATAGCATGCTTAAATTGTTCGCTAGATTTGGAAGCGCTCTTGCCGCTAAGCTTGTCAGCAGTGGATCAGACACCGCCCAGATACACCGCGACTTCGCAGCTTGTGTTGAACATATTAGTGGTGCTCGCATGAATACCATCTTATCGAGTTCTACGGTGGATGATGATCTGCTAGTGGTGGACATTAAACGTTCACTACAGTCAGGAGAACGTGCTACGTCCTTCACAAACACGTATCTAAACCGCGTTTACATCAAAATAGCGCAATTAGTATCGAATGACCTTTTAGGCTATGAACTCATTAAACCATCTCTTAGTCAACAGGGCGATGACGTTTTCTCATTGGTGGGCACGATCACGGACGGAGTTCTCGCTTGCACGCTCATTAATCTCATTGGTGCAGCTGGTCAGACTTATAAAATCACTCTCGATTATAATGGTAAGGGCGAGTTCTTGCGCTATGGTTACGATGCCATTAACCGCGTAGTACGAGGCTATCCTCTACGCACCTCACTAGGTTTATTAAGCGGCGAGTTCTTCATGGACCCGATCGTCGACCCAGATGGCCGGGCGGTTGCTTACCTTGAGGCTTGGCAGAAAGCTAATTTGCGTGGAGCTATCCTACCGAGATCTTTCCTTGATTACTTAATCCGCAATAACTGTTTCGTCACCTTCACTGATAATAATGGTGTCAAACATAGAGTAGTGGGTGATTTAGAGTTCGGCTTAACCCCCAAAGTTTACGGTGGTTTGGGCTCAGCTGGATATGTCGGCCACAACTTTGGAGGCAATCCCGAAGTTACAGTAGGCTACTCCAATCCTCGACCTCGTCCGTTGTACGAGCCGCCTAAGTTTGACTCTACCTATGTATTTAGGCACGTCAACCTCGTTGATCGTGCAGCATTAGTCAAATTAGGCATGGCGGAATCACTAACTGGCACTTTGTCGAAAGCTTTGGCTGCGAGCACTCTAACTGGTTCATACCCTGTTTCGAAGGCTTCTGAAGCGTTAGCATCTTATGCACGTAGGTTGTATGACTGGAGGATTAGGCTCGTTCGATCAAGACATGATCGCGTTAAAGCTGTTAGAGCTAATGTACCTGAACGGAACGTTGAAAAACTATTCCTACAAGCCTTAGGTCTCCACTCCCCCACTAACCAACCACATGCAATTGATTTCTTGGGCGCTGCTGTATGTATACCAAGCGGGCATGGAAAGACTGAACTAGCCAAAGAGTTCCCACATCTCTTTATTGACCATGATGATTACGTAGATCATACGTACGTCGCCGATTTAGTGGCCGCCAAAGAGTGGGATACACTTAACCTATATCACAGGACGGCTCGGTTACCACGTGATGGCAGAGTCTTATTGACCTGGTCACCCGAAACATGTCCCAGTGGACGGCCCGTCGTATGTGGAACAATTGTCCAGTCAGCGAGCGACAAACCACTGACGAAAGATAATGTGGCAGTTCTTGCACGGACACTCTCAACAAAGTTGAAAGTGTTCCAAGATTTCACTACGCGCAACAGCTTCTTATGTGAATTGGCTCGCGATACGATAGCAAAGGCACGCGCAGTTCCAATTCACGGTAACCCGATGTCCTTCAAGCCCGCGAGAAAGGTCTACCATGCCTTTAATGGAATTACTCGCTATGCCGCTGCTTGTGGCTTCTCGCTCACAGAAGTCTTCACGGCTGTGATCGCGGACTACCCCTCATCCTCTGGAGAAGGTGACTTGGGCAGGTTCTTCGCTATTTGTGAGAAACTAATACGCCCGCTTTCAAAACCACCCAAACATGAGGAAACCACTAAGTGGT

> SrURV1

CCGAGGATGTCCGCAGCAAGCGACGACGCACAAATGTCTCGATGGATGAGGAATGTCGCGCTCAATTTAAAGCGCGCCCGCTCGTATGAAGACGATCTGCATTCAATCGACACCACGGTGCCGAGCTCACCCGCGTCAAGCAGTGGAACCGCTAGGCGCCGCAATAAACGACATCGACTAAACTCGCAATCCTCGAGGACAGCCGAAGAGGATTCTATCACCCGCGCGTTGGAAGGATTGGAGTATTTAGGGAGAAATGATTCGGGGAGCCCCCTCCATATGGAATTGGAGTACGCCGAGCCGTGCATGCACTTCCGGCAGTTTGTCGAAGAGAATCCGGGGTTGTTCGAAGACACGCGTTTCGAGGATTGGAGGTTTGTGGAGGCAAATCACCTTGCGGAAATGAACCACCTCAAGCGCTTCTGCCGCGAGTATCCTGAAATCCAACCCCAACACGAGTGGGCGATTTCACGGGCGATCGAAGTAGTGTCAGCACTCATGGAGCTGCCTGAGAAGATACCCTTCCCAACGAAGGACCAGTTGGACGACGTACGCTGGATTGGCTCGAAGTACCCTGGCATCGAATACTTTCGCATGGGTTTTAAGACAAGGGCCGAGGCCAATCCCGTTGCACTCGAGCACGCACGCGCCGCATATGAGGACCTCATGAGCGGGCGTCGGGTGGAGCCGCAGTTGAATAAAATGGGGGGACGAGGGAAGTTGGTGAAGGAGGAGAAAATACGAGCGCAAGGGAAGGATACCACCGCGGGGCGTTTGATCCTCATGGCGAGTCAAAGAGACGTCTTATTAGCGGGGTTGCTGGAGCAGCCGCTTACGGCTGCGCTACTCGGGCCCGAGTATCCGGTCTCCGTAGGGACGAGTTGGTGGAAGGGTGGTGTCAACGACTTTATCACGCGCTTCCACAAATTTGAACGCTTCTTCTGCTTGGATGCGGAGAAGTATGACTCATCCTTACCGCCATGGTTGATAGAGATCGCGCTCCACATCCTCCGGGACCAATACGAAGATGGGCAAGATGAGAAGTATGACACCTACTGGGATTTCATACGCGACGGTTTGATCTACGCGCCGATCTACCTCCCAAATGGTATGATGTTCCGCAGAAAGGGCGGTTCCACGAGCGGTCATAACTTCAACACACTCATGCAATCCGTTGCGACCCTGGTGATGGTTTACACGGGGTTCTTTCTCCTTGTTGGTGAGGGGGAGGCAGATAGGGTTGAAGAAGAAGTGTACGCGGAATCACTCGGCGATGATCAACACACGGGTACACGAGGTGCCTGCGCTAAGTACGAGGTGGAGGATATTGCTGAGGTTGTAATGGAGGTGTTTCAGGTGTCCTTGGGGGGGGATAAGTCATTCGCCACAACGCGGCTGTTCGACGACATCGAGGGCGAGTTCCAAGGGACACAATATTTAGGTAAGTTTTGGAGGTGGTTCGATGAGGTTGTGGACGACATCCACTTCGAGGGTTACATCCCCTACCGGCCATTCGTCGAAACCGCACTACGGTTGTACTATCCCGAGAGGGGCGAGCAGGGAGAGCTCCCCGCTTGGCAACGGGCGGTGGGGCACTACGCCGACGCCGCCGGGTTGACGCGCACCAGGGCGATGTTGGAGCAATACCTGGATTGGTTGGAACCGCGGGTGGATGGGCAGCAGTTCTTTTGGACGGAGAGGCAATTGAAACGGTTGGGCGATAAAGTGAATAGACTACCCCCACCCCTACCCGCACGTCGTTGCGGCTTCGTAGAGTGGTTGCAGGCTGTATTAACGGACATCCCAGTGGAGTCCGCAAACTAAACGGGTTTTAACTGCCGCATCGGGTAGCGTGGGGTCGATTGGGAAAGTTGGATCCCTTTCAATGCCACGTTACCGGACGCCGCCGG

> SrURV2

AGGGATTACAGGTCAACTTCGAGGCTAGAGAATCCCTACAGTAGAAGGGCACGAATGGCCAACGCACGAATCAAGAGCTGGCAGGACGAAATGTCCAGGGGACTTAAACGGTCTCGTGAGGATGACCTCTTTGAGGAATTTGGACTCTCAACCGAAGTCCCATCTTCACCGGCCTCATCAAGAAGCTCGTCGCGCAGGAGAATACGCCGAAAACGCAGTAAACGAGCGGATCTCTCATCCGCAGAAGTTGAACTCACTCAGCGGTTCAATAAATTGAAGTATCTGGGACAAACGGAGTCCTACCGCGCATTGGATAGGGAACTACGCCACGCCGAACCCTGCTCTACTTTCCGGGCGTGGGTGGAGGATAACCCTGGCCTTTTCAGCGATATCGATTTCGCGCAATGGACGATGGTCGAAGCGAACTCAGTGGTGGAATTAAATCACCTTGAGCGCTTCTCAAGAGACTATCCTCTTCCTCCAGAAGCCGACACACCTGCCCTCGCCGCCGCAATCAGCTATGTGGCGGAACTGCTCAAGTTGGATGAGCAGATACCGTTTCCATCACGCGAGGACCTGGATGATGTGCGCTTCATTGGGAGCAAATTCCCTGGAGTAGAGTACGCGTGGCGCGGCTACAAAGATCGAAGCGGTGCTCACAGTGAAGCGTTGGACGACGCCAGAGCCGCTTGGGACCAACTCTTAGCAGGGGAGAGGGTTGAGCCGCACTACAATCGAATTGGAGGGCGAGGGAAGATGGTGACGAGGAAAAAGATAAAGGAGCAAGGTGACAAGACTACGGCAGGCCGCTTAATCTTCATGACGAGCCACCGCGACTTGCTGCTGAATGGGGTCCTTGAGCAACGTCTGACTAACGCCTACAAGGATCCCCACTTTCCAATATCAGTGGGCAAGACGTGGTGGCACGGGGGTACGGAGGAGTTCCTGGCTCGCTTTGGTGGGCGCGACATGTATTCATGTTTCGACGCAGCCAAATACGACAGCTCTCTTCCTCCCTGGTTGATTAAGGTGGCTGTGAACATTCTGCGTCAGCAGTTTGTGGATGGCGGGGACCCTGTCTACGATGCTTATTGGCGTTTCATCGAAGATGGTCTCATTTATGCTCCGGTGTACCTGGATAACGGTATGCTGTTCCAAAGGAGTGGCGGATCGACCAGTGGTCATTCGTTTAACACTTTGATGCAGAGTATATGCACGCTCATCATCGCCTACACCTGTTACTTGACGCTGGAAGGGCATGAGAACGTCGAGAGAGTAGCGCGTGAAGTGTGGTGTGAATCGTTGGGTGATGATCAACACACAGGTGCCTCCGGCCTGTGCGCAAGATGGAAGATTGAAGACACCGCGCCCATCGCAATGAGAATATTCGGAGTGGATTGGAGTGGCGACAAGTCGTTCAACACGAGACGCTTAGTAGACGATAGGGAGGGCGACTTTCAAGGCACGCAATACCTTGGAAAGTTCTTCCGTTGGTTTGATGAGGATGTTGAGGGGGTGCCCATTCAGGCCAACCTGCCTTATCGGCCATTCGTTGAAACGGCGTTACGCCTATACTACCCAGAGAGGGGCGAGCAGGGAATTCTCCCAGCTTGGCAACGCGCCATAGGCCATTACGCCGATGGTGCAGGCAACACGAGGACTCGCGCGATGTTGGAAGCGTATCTTGATTGGCTTGAGCCTCAGGTGGGTGAGGGAGAGTTCGTTTGGTCTGAGAAGTGGCTTCGTAGGTTTGAAAGAGCGGGGGAAGAGTTTCAGGCGCCTGTGCCGCCACGCCGCATAGGGTTCTCGGCGTGGTTAGCTCTTGTAATTACAGATCGTGATCCATTCAGTTAAACTCTCGGGTCCTACAACCACTGAGCGGCGATTTGGCTGCCCACCGGCGTTTGGTTGACTCCGGTGGGACCGCCATTTCGCGGTTCAAAGCGTGCGGACAGCCG
